# Determination of the absolute configuration of conformationally flexible molecules by simulation of chiro-optical spectra: a case study†

**DOI:** 10.1039/c9ra03526e

**Published:** 2019-06-10

**Authors:** Michele Mancinelli, Roberta Franzini, Andrea Renzetti, Emanuela Marotta, Claudio Villani, Andrea Mazzanti

**Affiliations:** Department of Industrial Chemistry “Toso Montanari”, University of Bologna Viale Risorgimento 4 40136-Bologna Italy andrea.mazzanti@unibo.it; Dipartimento di Chimica e Tecnologie Del Farmaco, Sapienza Università di Roma P.le A. Moro 5 00185 Roma Italy Claudio.villani@uniroma1.it; Faculty of Education, Room 426, University of the Ryukyus 1 Senbaru, Nishihara Okinawa 903-0213 Japan

## Abstract

The assignment of the absolute configuration (AC) of two conformational flexible organic molecules by means of TD-DFT simulation of the electronic circular dichroism (ECD) spectra is presented. The factors leading to a reliable assignment were evaluated in the various steps of the process. The effects of different functionals and basis sets in the geometry optimization step is very limited in terms of the resulting optimized geometries, whereas the inclusion of the solvent in the calculations has a much larger effect on the correct evaluation of the conformational ratio. B3LYP and M06-2x were found to be the most accurate functionals for geometry optimization. CAM-B3LYP and ωB97X-D provided the best results in the TD-DFT simulations.

## Abstract 2

The absolute configuration (AC) assignment of two very flexible organic molecules by TD-DFT simulation of ECD spectra is evaluated by modifying the calculation level in the various steps of the AC assignment process.

## Graphical abstract

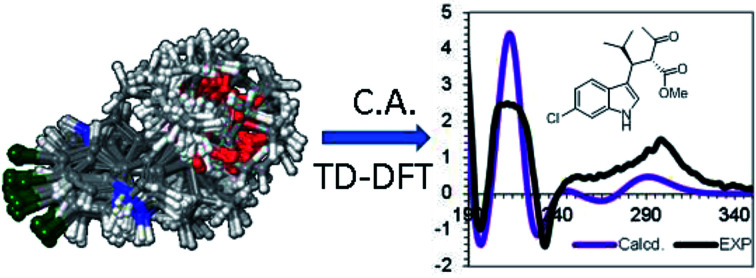

## Introduction

The determination of the absolute configuration (AC) of synthetic molecules and natural products is still one of the most challenging tasks in organic chemistry. Since the first experimental determination from Bijovet,^1^ the anomalous dispersion X-ray technique has been the most widely used approach to determine the AC of organic molecules. This methodology, although based on experimental data, is not free from pitfalls and drawbacks.^2^ Over the decades, the availability of this technique has been widened by the use of Cu-Kα X-ray sources, that allow a reliable determination of AC also in those molecules missing heavy atoms,^3^ such as many natural compounds. However, the primary obstacle to the use of X-ray diffraction is still the preparation of good single crystals, which in many cases is impossible or unfeasible due to the limited amount of compound.

Thanks to the development of computational methods, the assignment of the AC by the quantum—mechanical simulation of chiro-optical spectra has become a “classical” alternative to the X-ray methodology. In the past few years, the simulation of optical rotatory dispersion (ORD), electronic circular dichroism (ECD) and vibrational circular dichroism (VCD) spectra have allowed the determination of the AC of a wide typology of organic molecules.^4^ Some operative protocols have been formalized^5^ and methods have been devised to evaluate the reliability of the various simulations.^6^

Within the time-dependent DFT (TD-DFT) method for the simulation of the ECD spectra, many combinations of DFT functionals and basis set have been used for the various steps of the AC assignment. Some benchmark studies have been performed on the TD-DFT simulation of the UV spectra of small molecules,^7^ organic dyes^8^ and metal complexes.^9^ Here we present a case study on two diastereomeric, highly flexible, organic molecules with few UV-chromophoric groups, in which we evaluate in details the performance of various combination of functionals and basis sets in the conformational analysis step, by comparison with experimental NMR data. The performance of TD-DFT with different functionals and basis sets is also performed to investigate the key factors that lead to a reliable interpretation of the chiro-optical spectra.

## Results and discussion

**Scheme 1:**
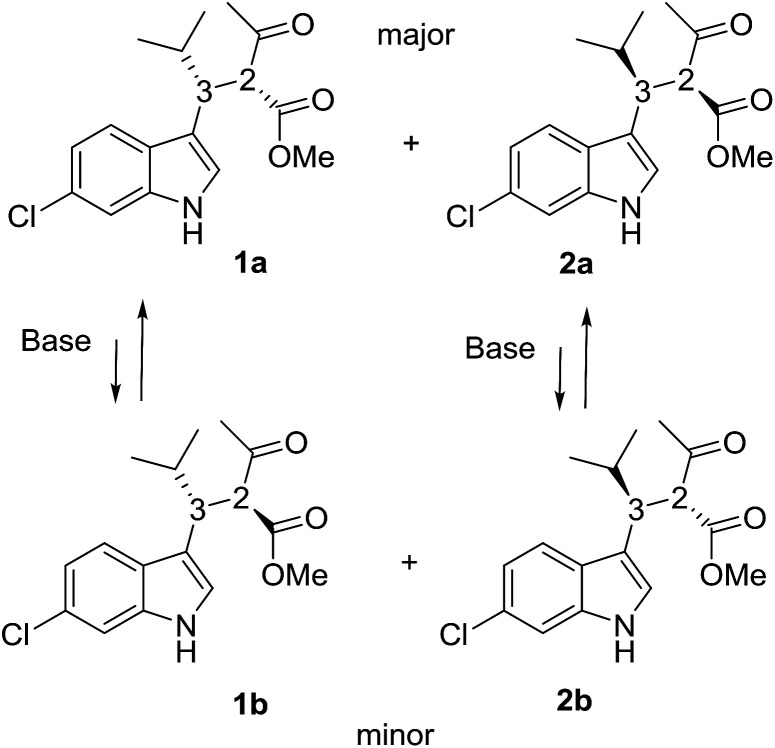
Stereoisomers of the compound investigated in this study.

**Fig. 1:**
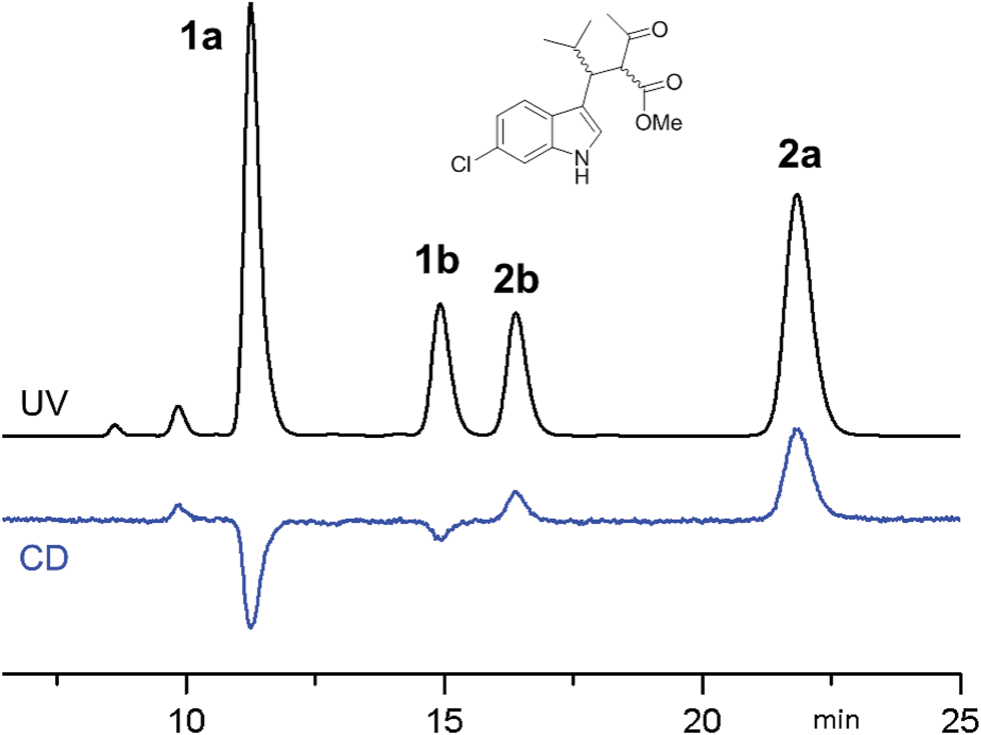
CSP-HPLC separation of compounds 1a, 1b, 2a and 2b (Daicel Chiralpak IA, hexane/2-propanol 93 : 7, 1 mL min^−1^, +25 °C). Top: UV detection at 280 nm. Bottom: ECD detection at 280 nm.

Compounds 1a + 2a and 1b + 2b (Scheme 1) were prepared by a single reaction according to a reported procedure.^10^ Due to the reaction conditions employed, the presence of the two stereocenters forged during the reaction entails the formation of four stereoisomers. The two diastereoisomeric pairs can be easily converted into each other by treatment with a base, due to the high acidity of the acetoacetate proton,^11^ yielding an equilibrium ratio of 70 : 30 (in ethanol at +25 °C). The four stereoisomers resulting from the reaction were resolved by chiral stationary phase HPLC (CSP-HPLC) on Chiralpak IA column (Fig. 1).

ECD detection allowed to confirm that the two major peaks are enantiomers (1a and 2a), as well are the minor ones (1b and 2b). A chemical correlation followed by CSP-HPLC in the presence of Et_3_N as base allowed to determine that the second eluted peak 1b converts into the first eluted 1a (Fig. S1 in ESI†). Thus the first and the second peak are diastereoisomers (1a*vs.*1b) with the same AC at the C3 carbon, and the same happens for the third and fourth eluted peaks (2b and 2a, respectively).

Thanks to the presence of the chlorine, the absolute configuration of stereoisomers 1a and 2b (*i.e.*, the minor stereoisomer of 2a, third eluted peak on CSP-HPLC) was determined by anomalous dispersion X-ray crystallography.^12^

**Fig. 2:**
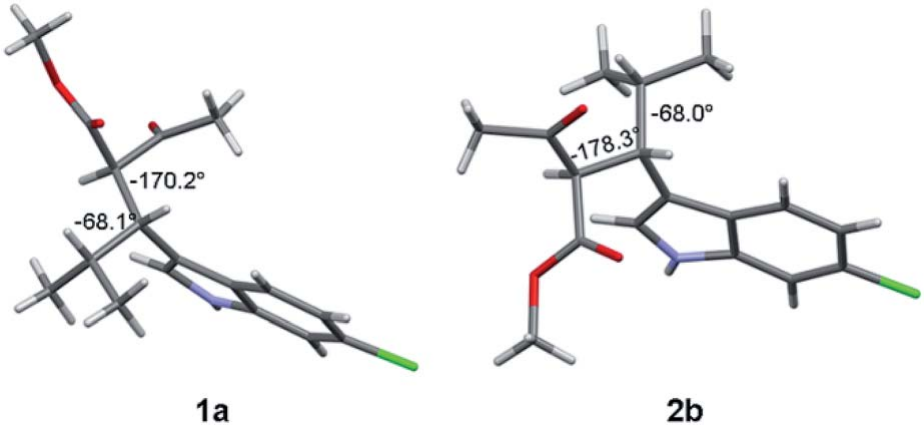
X-ray structure of compounds 1a (2*R*, 3*R*) and 2b (2*R*, 3*S*). The numbers indicate the dihedral angles between the H2 and H3 hydrogens, and between H3 and H4.

Compound 1a (Fig. 2, left) crystallized by evaporation (24 h) from a chloroform solution in the *P*2_1_2_1_2_1_ chiral space group, and the 2*R*,3*R* absolute configuration was assigned with Flack parameter = 0.03.^10^ After many attempts, compound 2b (Fig. 2, right) crystallized by slow evaporation (10 days) from an acetonitrile solution in the same *P*2_1_2_1_2_1_ space group, and the 2*R*,3*S* AC was assigned (Flack parameter = 0.04).‡‡CCDC 1897062 and 1897063 contains the crystallographic data, see ESI for details. Being 2b the enantiomer of 1b, the absolute configuration of 1b is therefore 2*S*,3*R*, in agreement with the base-catalyzed mechanism of diastereomerization. An analogous reasoning applies to 2a (enantiomer of 1a), whose AC is (2*S*,3*S*).

### NMR conformational analysis and dynamic behaviour

**Scheme 2:**
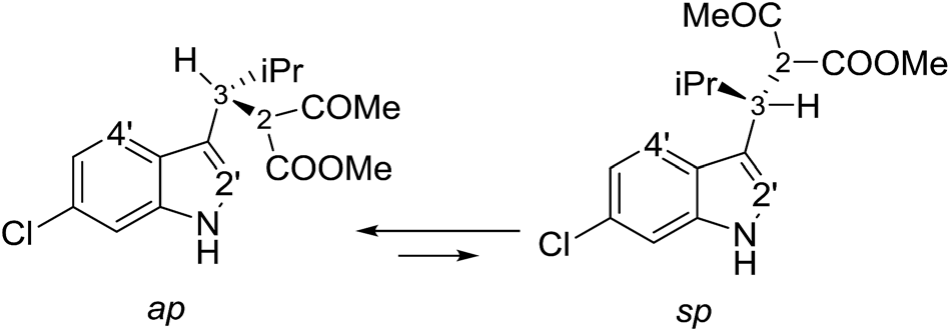
The two available conformations of 1a due to indole–CH rotation.

Compounds 1a and 1b showed similar ^1^H-NMR spectra in terms of coupling constants. The H2–H3 ^3^*J* coupling constant was 11.9 Hz for 1a and 11.3 Hz for 1b (in CD_3_CN), whereas the H3–CH_i-Pr_^3^*J* coupling constants were 3.9 and 4.7 Hz, respectively. The very large values for the H2–H3 coupling suggest that most of the populated conformations have these hydrogens in an *anti*-relationship, with the dihedral angle close to 180°,^13^ whereas the small coupling constant between H3 and CH_i-Pr_ suggests a predominant *gauche* arrangement. This outcome is in full agreement with the solid state structures (Fig. 2). Due to the steric hindrance of the isopropyl group and of the acetoacetate moiety, compounds 1a and 1b can be conformationally correlated to 1-naphthyl aryl-carbinols, which are known to generate two conformational diastereoisomers due to the restricted rotation of the sp^2^–sp^3^ bond (Scheme 2).^14^ This conformational preference can be conveniently described by the *anti*-periplanar (*ap*) and *syn*-periplanar descriptors (*sp*).^15^

Variable temperature NMR experiments on 1a allowed to freeze the rotational motion around the indole–C3 bond, and to determine that the *ap* and *sp* conformations are populated in a 90 : 10 ratio at −100 °C (in CDFCl_2_, Fig. S2 in ESI†), with an experimental Δ*G*° = 0.75 kcal mol^−1^, corresponding to a 78 : 22 ratio at +25 °C. NOE spectra recorded at ambient temperature in CD_3_CN showed that saturation of the H3 signal yielded strong NOEs on the H4′ and H2′ signals of the indole (Fig. S3 in ESI†), it was confirmed that the *ap* and *sp* conformations are both populated also in a polar solvent. Saturation of the COMe signal yielded similar NOEs on H2 and H3, thus suggesting that the two conformations obtained by rotation of the COMe moiety are both populated.§§Although the rotation of the COMe is fast in the NMR timescale, the observed enhancements are the result of the weighted average of the NOEs of the populated conformations. In the case of 1b, low temperature NMR spectra showed that the *ap*/*sp* ratio was 94 : 6 at −110 °C (Fig. S2 in ESI†).

### Conformational analysis

**Fig. 3:**
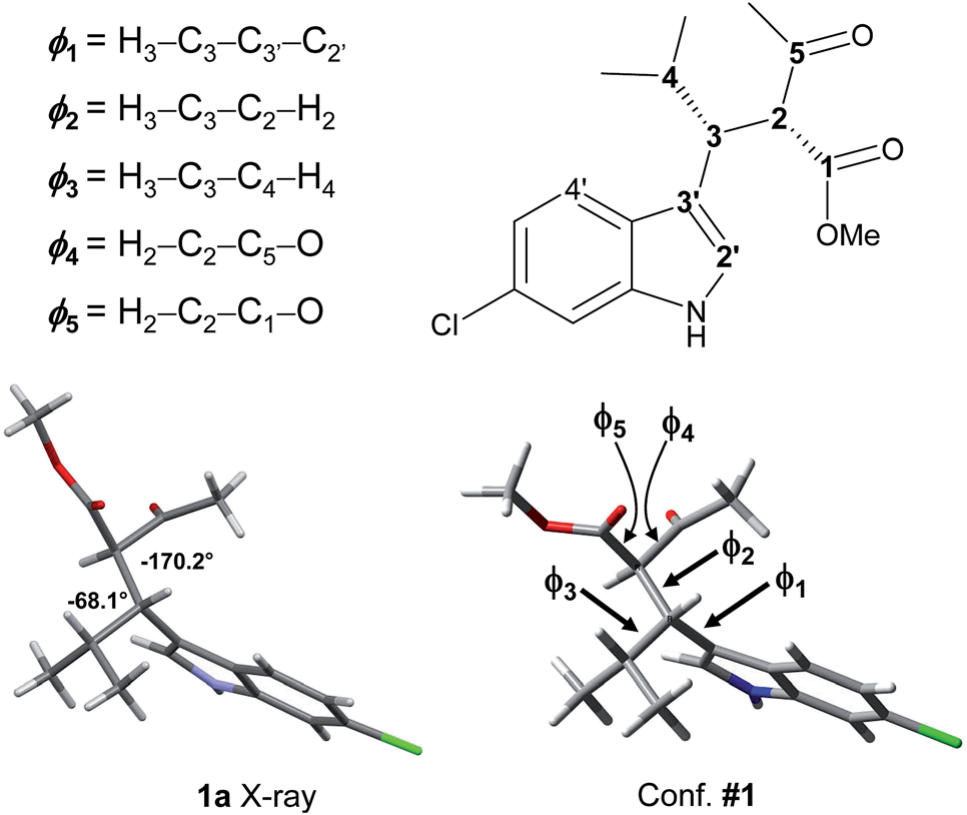
The X-ray structure of 1a and geometry of conformation #1, with dihedrals definition as from Tables 1 and 2.

Because 1a and 1b are very flexible molecules, the conformational analysis step must consider a wide conformational space. The full conformational space was explored by molecular mechanics (MM) using the GMMX algorithm included in the Compute-VOA™ software,^6*b*^ as well as the MMFF force field, and retaining all the structures enclosed within a 10 kcal mol^−1^ range. A total of 66 energy minima were found for compound 1a. These conformations were then optimized using B3LYP^16^ and the 6-31G(d) basis set with the Gaussian 16 suite of programs.^17^ After DFT optimization some conformations were proved to be redundant, with only 50 geometries being really unique. All were checked to be true energy minima by frequency analysis (no imaginary frequencies were observed). Among these, 12 conformations were enclosed in 2 kcal mol^−1^ (Table 1 and Fig. S4 in ESI†). Each conformation can be conveniently described by three main descriptors: (1) the H3–C2′ dihedral angle, *i.e.*, the angle between the benzylic CH and indole, that yields the *ap*/*sp* conformations (*ϕ*_1_ in Fig. 3 and Table 1); (2) the H2–H3 dihedral angle, *i.e.*, the dihedral between the benzylic CH and the CH of the acetoacetate moiety (*ϕ*_2_); and (3) the H3–H_i-Pr_ dihedral angle between the benzylic CH and the isopropyl CH (*ϕ*_3_; 3D structures are reported in Fig. S4 of ESI†).

**Table 1:** Summary of the optimized conformations of 1a after MM conformational search and optimization by DFT at the B3LYP/6-31G(d) level. Relative energies in kcal mol^−1^

Conf. #^a^	Rel. E	*ϕ*_1_ *sp*/*ap*	*ϕ*_2_ H_2_–H_3_	*ϕ*_3_ H_3_–H_4_	*ϕ*_4_ CH–COMe	*ϕ*_5_ CH–COOMe
1^b^	0.00	*ap*	−175	59	5	174
4	0.28	*ap*	−47	−175	−133	−124
8	0.89	*ap*	−52	−173	−128	43
9	1.00	*ap*	−171	−71	5	177
19	1.29	*sp*	−179	58	−168	150
35	1.31	*ap*	178	57	−165	152
59	1.33	*ap*	−159	−176	−1	162
16	1.34	*ap*	171	56	−152	−8
7	1.56	*sp*	171	47	−153	−7
6	1.68	*ap*	−179	58	13	2
46	1.90	*sp*	−175	59	5	174
33	1.94	*ap*	69	−171	−78	156
99	3.74	*sp*	−178	62	10	−8

a The conformation labels refer to the energy order obtained from MM conformational search.

b Conformation observed in the solid state.

The calculations results can be checked by the experimental NMR coupling constants and by low temperature spectra. Analysis of the data of Table 1 show that the most preferred conformation is the *ap*, where H3 is close to the H4′ of indole (conf. #1); this is the conformation observed in the solid state. Only three conformations have the *sp* geometry, and the most stable one (#19) is 1.29 kcal mol^−1^ above the global minimum, in agreement with low temperature NMR data. In nine conformations the H2–H3 dihedral angle is close to |180|°, and the H3–H4 dihedral is close to |60|°. Again this geometric preference is in agreement with the experimental ^3^*J* coupling constants in the ^1^H NMR spectrum. However, conformations #4, #8, and #33 are calculated at very low energy with respect to the global minimum and they seem to disagree with the observed coupling constant values. To investigate this point, we calculated the NMR coupling constants H2–H3 and H2–H4 (corresponding to *ϕ*_1_ and *ϕ*_2_) at the GIAO-B3LYP/6-311++G(2d,p) level and including the Fermi contact term.^18^ The Boltzmann averaged values were 10.8 Hz and 6.1 for *ϕ*_1_ and *ϕ*_2_, in good agreement with the experimental values (see Table S1 in ESI†). Once the three main descriptors are fixed, the optimized conformations differ by the relative disposition of the two carbonyl groups (*ϕ*_4_ and *ϕ*_5_). It is worth to note that the conformation calculated as the most stable (#1) is that observed in the X-ray structure (Fig. 3).

We also noted that only seven out of the eight available dispositions of the two carbonyl groups were present in the optimized geometries. For the sake of completeness, this last conformation (labelled as #99) was manually built and optimized. Its energy was calculated to be 3.74 kcal mol^−1^ above the global minimum.

The same conformational analysis was performed for compound 1b. For this compound the MM conformational search found 107 conformations, which were reduced to 16 after optimization at the B3LYP/6-31G(d) level and elimination of the duplicates (Table 2 and Fig. S6 of ESI†). As in 1a, the *ap* conformation is predominant (ten out of sixteen conformations), with only two *sp* conformations enclosed among the first ten. Conformation #21 corresponds to the experimental X-ray structure of 2b (actually its enantiomer), so in this case the calculated global minimum is not in agreement with the X-ray structure. However, 2b was crystallized from a polar solvent, whereas the calculations have been hitherto made in the gas phase.

**Table 2:** Summary of the relative energies (Rel. E) and geometric parameters of the sixteen conformations of 1b after MM conformational search and optimization by DFT at the B3LYP/6-31G(d) level. Energies in kcal mol^−1^

Conf. #^a^	Rel. E	*ϕ*_1_ *sp*/*ap*	*ϕ*_2_ H_2_–H_3_	*ϕ*_3_ H_3_–H_4_	*ϕ*_4_ CH–COMe	*ϕ*_5_ CH–COOMe
37	0.00	*ap*	44	178	122	151
2	0.12	*ap*	173	−62	−156	−10
14	0.46	*ap*	49	175	−42	141
5	0.52	*ap*	−179	−62	2	169
21^b^	1.09	*ap*	176	−63	−160	169
17	1.14	*sp*	73	177	−108	37
3	1.24	*ap*	−66	170	−150	70
7	1.28	*sp*	−177	−62	−1	172
23	1.33	*ap*	156	175	−150	−1
1	1.37	*ap*	−65	170	−140	−111
15	1.48	*sp*	79	−177	−107	−146
22	1.64	*sp*	80	−178	−108	−139
9	1.66	*ap*	−178	−61	−1	−14
8	1.72	*sp*	173	−63	−157	−6
20	1.95	*ap*	179	62	153	4
44	1.96	*sp*	158	176	−152	−173

a The conformation label refers to the energy order obtained from MM conformational search.

b Conformation observed in the solid state.

To investigate the effect of various optimization parameters, the thirteen conformations of 1a were optimized using B3LYP and the larger 6-311++G(2d,p) basis set, and for each basis set the optimization was carried out both in the gas phase and in acetonitrile using the IEF-PCM formalism.^19^ Acetonitrile was used because it was solvent used to record the NMR and ECD spectra at ambient temperature (see below). Calculations yielded the four sets of data shown in Tables 3 and S2 of ESI.† The relative energies are very different when the solvent is included in the calculations, whereas the effect of the different basis set is very small.^20^ When solvent is included, seven out of the eight most stable conformations have identical dihedral parameters, with the H2–H3 dihedral being *anti* and the H3–H_i-Pr_*gauche*, in full agreement with experimental NMR data. Conformation #1 is the most stable conformation in the gas-phase calculations, but the most stable conformation when the solvent is included is only the 35th in the MM search, with a relative energy of 3.6 kcal mol^−1^*vs.* the global minimum, and 1.47 kcal mol^−1^*vs.* the gas-phase DFT calculations.¶¶This occurrence is not in contrast with the X-ray data because the crystals were grown from a low polarity solvent such as chloroform. Unfortunately, chloroform cannot be used for ECD because its absorbance limits the range of the ECD spectrum to 230 nm. This result confirms once more that conformational analysis has to take into account a broad energy range (at least 10 kcal mol^−1^) to guarantee that all the low energy conformations are included in the following DFT step.

**Table 3:** Relative energies of the best thirteen conformations of 1a calculated with B3LYP using two different basis sets, with or without the solvent (IEF-PCM). Relative energies in kcal mol^−1^, as ZPE-corrected enthalpies

Conf #^a^	6-31G(d)	6-311++G(2d,p)	PCM-6-31G(d)	PCM-6-311++G(2d,p)
35	1.16	1.47	**0.00**	**0.00**
1^b^	**0.00**	**0.00**	0.51	0.30
16	1.21	1.40	0.55	0.41
6	1.68	1.70	1.59	1.07
19	1.25	1.68	1.21	1.29
59	1.22	1.65	1.85	1.45
46	1.90	2.03	1.68	1.62
7	1.56	1.49	1.72	1.74
4	0.11	1.09	1.17	1.97
9	1.04	1.42	1.95	2.09
8	0.76	0.97	1.55	2.20
99	3.75	3.90	2.84	2.52
33	1.76	2.38	2.44	2.95

a The conformation label refers to the energy order obtained from MM conformational search.

b Conformation observed in the solid state.

**Fig. 4:**
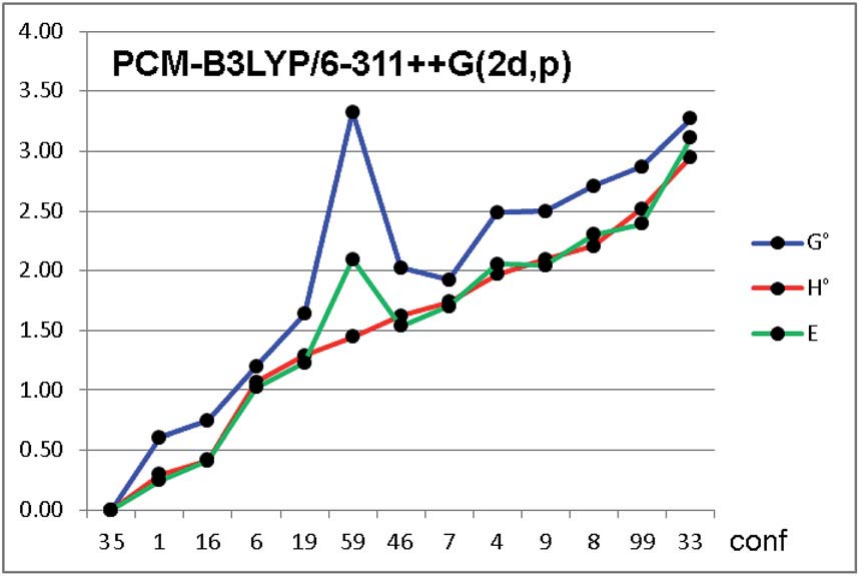
Relative energies of the thirteen conformations of compound 1a, optimized by PCM-B3LYP/6-311++G(2d,p). Green line = relative energy as uncorrected internal energy (*E*); red line = ZPE-corrected enthalpy (*H*°); blue line = ZPE-corrected Gibbs free energy (*G*°). Conformations on abscissa are arranged in increasing order of *H*°. Energies in kcal mol^−1^.

Among the thermochemistry data obtained after frequency analysis, we choose to use the ZPE-corrected enthalpies, rather than the Gibbs free energies, to derive the relative energies. As a matter of fact, the use of uncorrected internal energies, ZPE-corrected enthalpies or Gibbs free energies did not modify the results to a great extent, except for a few conformations, in which the entropic correction yielded very different relative energies (*e.g.* conf. #59 in Fig. 4, see also Fig. S5 in ESI†).

Frequency analysis revealed that about 10% of the values were below 200 cm^−1^, and about 25% were below 500 cm^−1^. Although some methods have been proposed to cushion this issue,^21^ they inevitably hampers the correct evaluation of the entropic factor in the thermochemistry corrections to the internal energies. In the conformational analysis realm, the ground state entropic factors between different conformations are expected to be very similar for different conformations, and the different entropic corrections obtained for the various conformations are mainly due to the contributions of the very low frequencies modes, all ascribable to internal conformational motions.

**Fig. 5:**
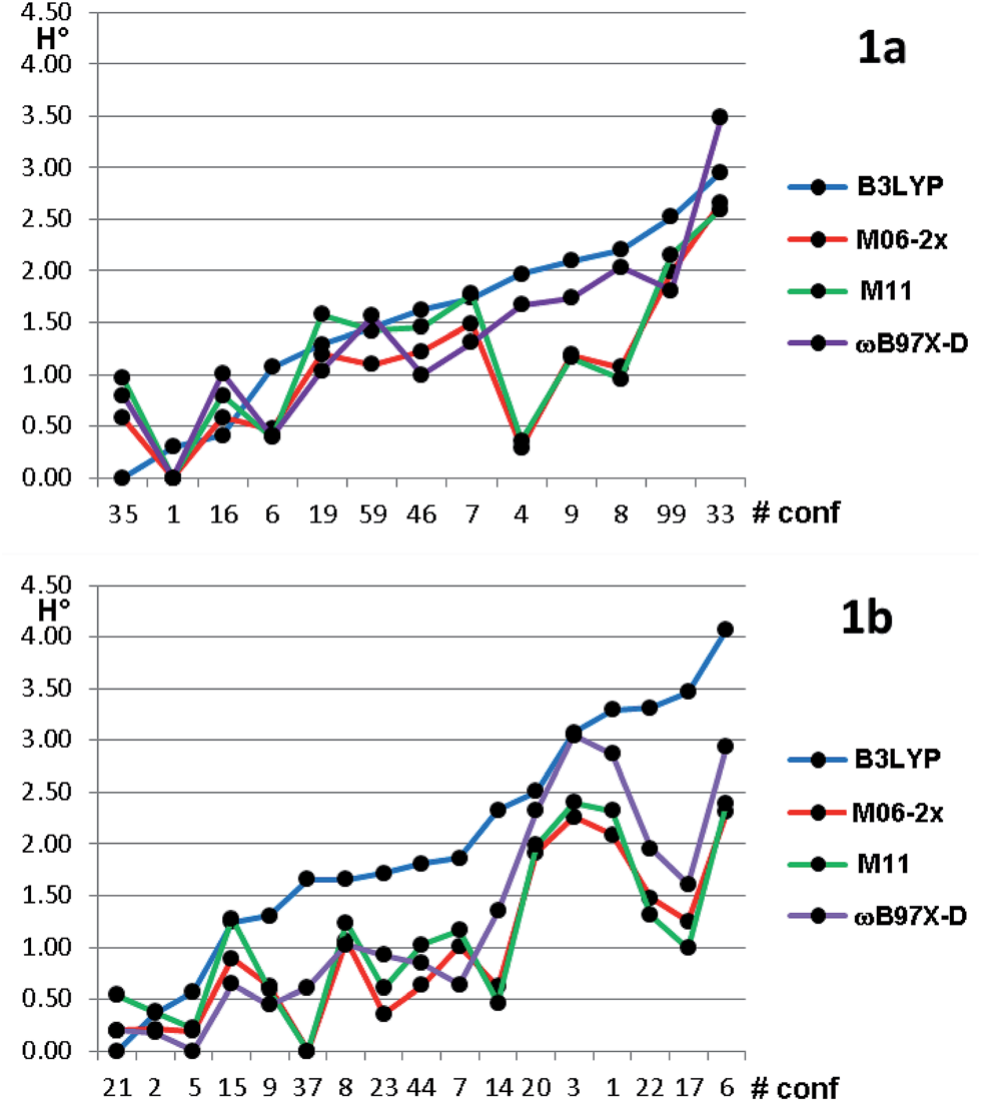
Relative ZPE-corrected enthalpies for the thirteen conformations of 1a and the sixteen conformations of 1b. The abscissa reports the conformations numbers arranged in increasing energy order, based on B3LYP optimization. Energies in kcal mol^−1^.

Based on the results of Table 3, we fixed the largest basis set and the inclusion of solvent, and we investigated the effects due to the functional, since B3LYP has been recently considered not fully appropriate for the relative energy evaluation.^22^ Among the plethora of functionals developed so far, we selected the global hybrid M06-2x^23^ and the range-separated hybrids M11 (ref. 24) and ωB97X-D.^25^ The thirteen conformations of 1a and the sixteen of 1b were fully optimized again in acetonitrile (IEF-PCM) using the three functionals and the 6-311++G(2d,p) basis set (Table 4 and Fig. 5).

**Table 4:** Relative ZPE-corrected enthalpies for the conformations of 1a and 1b, calculated in acetonitrile with various functionals and the 6-311++G(2d,p) basis set. Relative energies are in kcal mol^−1^. Bold numbers indicate the conformations within the 1 kcal mol^−1^ limit *vs.* the global minimum (bold and underlined)

Compd. 1a					Compd. 1b				
Conf #	B3LYP	M06-2x	M11	ωB97X-D	Conf #	B3LYP	M06-2x	M11	ωB97X-D
35	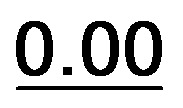	**0.58**	**0.97**	**0.80**	21^a^	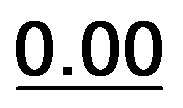	**0.20**	**0.54**	**0.20**
1^a^	**0.30**	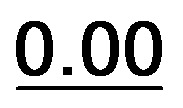	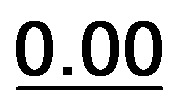	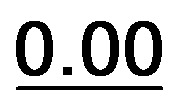	2	**0.36**	**0.21**	**0.38**	**0.18**
16	**0.41**	**0.58**	**0.80**	1.01	5	**0.57**	**0.19**	**0.22**	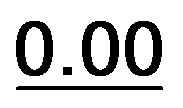
6	1.07	**0.47**	**0.39**	**0.41**	15	1.24	**0.89**	1.28	**0.64**
19	1.29	1.20	1.57	1.04	9	1.30	**0.62**	**0.59**	**0.44**
59	1.45	1.10	1.42	1.57	37	1.65	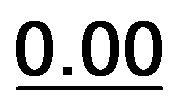	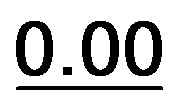	**0.61**
46	1.62	1.23	1.46	1.00	8	1.66	1.06	1.23	1.03
7	1.74	1.49	1.78	1.31	23	1.72	**0.36**	**0.61**	**0.93**
4	1.97	**0.29**	**0.36**	1.67	44	1.81	**0.63**	1.02	**0.84**
9	2.09	1.19	1.17	1.74	7	1.86	1.01	1.17	**0.64**
8	2.20	1.07	**0.96**	2.04	14	2.33	**0.62**	**0.46**	1.35
99	2.52	1.99	2.16	1.81	20	2.51	1.92	1.99	2.32
33	2.95	2.66	2.60	3.48	3	3.07	2.26	2.40	3.05
					1	3.30	2.09	2.32	2.87
					22	3.31	1.48	1.31	1.95
					17	3.47	1.25	0.99	1.61

a Conformation observed in the solid state.

For each functional, the conformations of 1a within 1 kcal mol^−1^ from the global minimum were optimized also using the SMD model^26^ for the solvent, without appreciable variations (see Table S3 of ESI†). Three functionals (M06-2X, M11 and ωB97X-D) found the same global minimum for 1a (#1). B3LYP and ωB97X-D performed similarly, whereas the two Minnesota functionals showed different trends, because conformations #8, #9 and #4 were calculated as much more stable with respect to the other functionals. In all the cases the Boltzmann-averaged NMR coupling constants corresponding to *ϕ*_2_ and *ϕ*_3_ were in fair to good agreement with the experimental values (see Table S1 of ESI†). Only three conformations were found by B3LYP and ωB97X-D within the 0–1 kcal mol^−1^ range, whereas M06-2x and M11 found five and six conformations in the same range, respectively. It appears that B3LYP and ωB97X-D results are in better agreement with the experimental data, while M06-2x and M11 slightly overestimate the stability of two conformations, whose population is experimentally small on the basis of NMR coupling constant analysis. The same computational process applied to compound 1b gave even more complex results. Three different conformations were calculated as the global minima from B3LYP, ωB97X-D, and by the two Minnesota functionals. B3LYP found only three conformations within 1.0 kcal mol^−1^ from the global minimum; ωB97X-D found nine conformations within the same energy range. The relative energy of conformation #21 (that found in the solid state) is always very low, but it corresponds to the global minimum only in the B3LYP optimization. It should be also noted that the conformation found as global minimum by both the Minnesota functionals is #37, whose energy is 1.65 kcal mol^−1^ in the B3LYP calculations. From the structural point of view, conformation #37 has the isopropyl CH in *anti*-disposition with H-3, whereas the small experimental NMR coupling constant value suggests that the most populated conformations have a *gauche* relationship.

### Absolute configuration

**Fig. 6:**
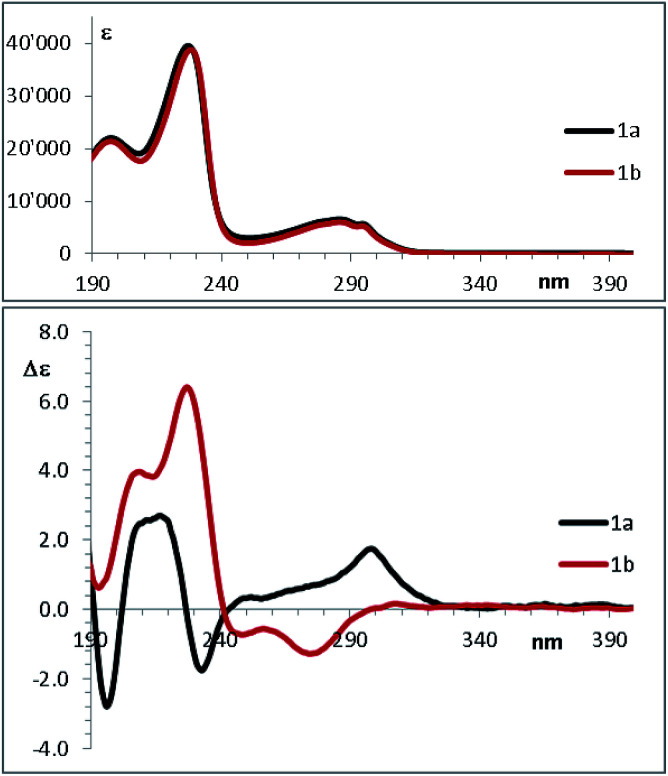
UV (top) and ECD spectra (bottom) of 1a and 1b in acetonitrile.

In the past few years, the computational methods based on DFT and TD-DFT calculations for the simulation of chiro-optical spectra have known a widespread use. It has often underlined that the simulations are heavily influenced by the geometries employed in the calculation.^27,28^ Compounds 1a and 1b have a single strong UV-chromophoric group (*i.e.* the indole ring), and the two stereogenic carbons do not bear any additional UV moiety that could interact with the indole to produce exciton couplings.^29^ As shown by calculations above, the conformational flexibility of these compounds is reflected by the existence of many low-energy conformations. The large conformational freedom and the absence of multiple chromophores make the simulation of chiro-optical spectra particularly challenging. The UV and ECD spectra of 1a and 1b were obtained in acetonitrile as the average of 16 spectra taken in the 185–400 nm region (Fig. 6). The UV spectra are dominated by the absorption bands of the indole^30^ and, as conceivable, the ECD spectra are rather weak. Compound 1a shows a weak and structured positive band between 295 and 305 nm, two negative bands at 235 and 195 nm, and a broad positive band in the 230–200 nm region. Compound 1b has two broad negative bands in the 290–240 nm region, and positive bands in the high-energy region (230 and 205 nm).

**Fig. 7:**
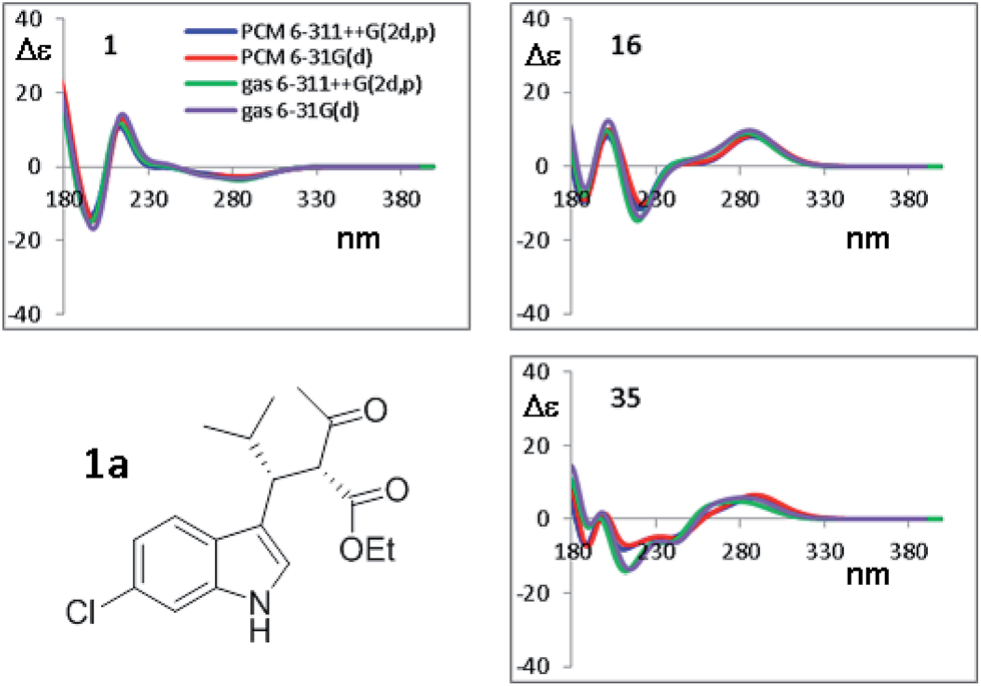
Selected TD-DFT simulations obtained at the CAM-B3LYP/6-311++G(2d,p) level using the four geometries optimized by B3LYP, using the 6-31G(d) and 6-311++G(2d,p) basis sets, in both the gas phase and including the solvent acetonitrile. Only the spectra for three most stable conformations from Table 3 are shown. All the simulations are reported in Fig. S8 of ESI.†

To simulate the experimental ECD spectrum, the spectra of all conformations have to be simulated and then mixed using the Boltzmann distribution. Therefore, two main factors influence the shape of the final simulated spectrum: (1) the combination of functional and basis set used for the calculation of the ECD spectrum by TD-DFT; (2) the combination used for the geometry optimization. The latter step also affords the conformational ratio to be applied in the Boltzmann averaged spectrum. TD-DFT calculations were performed in two steps. Firstly, we simulated the ECD spectrum of 1a (2*R*,3*R* absolute configuration) at the CAM-B3LYP/6-311++G(2d,p) level using the geometries obtained with the B3LYP functional combined with two different basis set [6-31G(d) and 6-311++G(2d,p)], both the in the gas-phase and with the solvent. For each conformation, the first 50 excited states were calculated, and the spectra were obtained using a 0.25 eV linewidth at half height. As shown in Fig. 7, the differences among the four ECD spectra calculated for the same conformation are extremely small. For some conformations the simulations in the gas phase and in acetonitrile, obtained with the same basis set, are even superimposable (see Fig. S8 of ESI†). This result suggests that the optimized geometries are very similar, and in many cases almost identical.

On the other hand, the calculated spectra are very different among the thirteen conformations (an example of superimposition of the spectra is shown in Fig. S7 of the ESI†), so the conformational ratio employed in the Boltzmann-averaged simulation of the experimental ECD spectrum will play a key role to correctly simulate the experimental spectra. This situation is due to the presence of a single UV chromophoric group, which makes the spectra very weak and strongly dependent on the whole molecular geometry. For example, conformation #1 and #6 are both *ap* conformations differentiated by the rotation of COOMe moiety, and the pairs #6/#16 and #1/#35 have different conformations due to the rotation of the COMe group.

As the second step, the PCM-B3LYP/6-311++G(2d,p) optimized geometries were used for the TD-DFT calculations using CAM-B3LYP^31^ with four different basis sets [6-311++G(2d,p), 6-311G(d,p), 6-31+G(d,p) and 6-31G(d)]. The results (Fig. S9 of ESI†) show that the four simulations are always very similar in the low-energy region, but quite different in the high-energy region where charge transfer (CT) and Rydberg transitions take place. Careful inspection revealed indeed that the pair of basis sets including diffuse functions yield very similar spectra, as does the pair without diffuse functions.

The ECD spectra of the thirteen conformations of 1a were then calculated using the four sets of optimized geometries obtained by PCM-B3LYP, PCM-M06-2x, PCM-M11 and PCM-ωB97X-D and the same 6-311++G(2d,p) basis set. Based on previous experience,^32^ TD-DFT calculations were performed with BH&HLYP,^33^ M06-2x, ωB97X-D and CAM-B3LYP. All the ECD spectra were calculated using the 6-311++G(2d,p) basis set, eventually yielding a 4 × 4 data matrix for each conformation. With these data sets in hand, first we checked the effect of the different optimized geometries on the simulated spectra, calculated with the same TD-DFT functional.

**Fig. 8:**
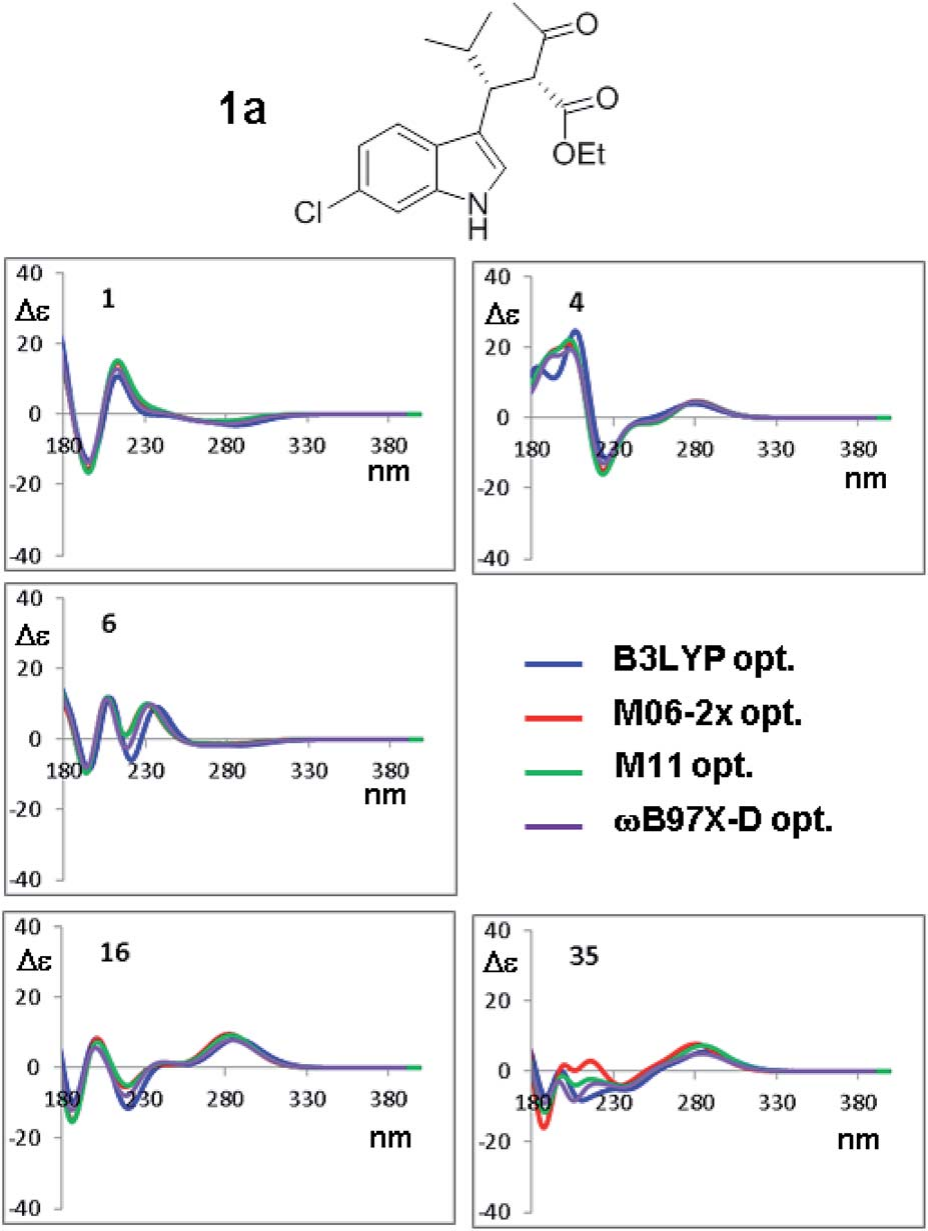
Selected ECD simulations for the conformations of 1a. The number in each quadrant is the conformation label, the four colored lines are the ECD spectra obtained with TD-DFT using CAM-B3LYP/6-311++G(2d,p) and the four geometry sets obtained with the four different functionals, including the solvent (PCM) and with the same 6-311++G(2d,p) basis set. The simulations for all the conformations are reported in ESI.†

The results shown in Fig. 8 for CAM-B3LYP suggest that the input geometries optimized with four different functionals yield very similar ECD spectra, with tiny differences mainly located in the high-energy region of the spectrum (*e.g.* conformations #7 and #35; the remaining spectra of 1a and those of 1b (2*S*,3*R* AC) are reported in Fig. S10–S17 of ESI†). Both the optimization functionals, and the optimization basis set as well (see Fig. 7), have a very little influence on the final result. This also suggests that it is not necessary to recalculate the ECD spectra using geometries coming from additional optimizations, as this will not lead to more accurate results.

**Fig. 9:**
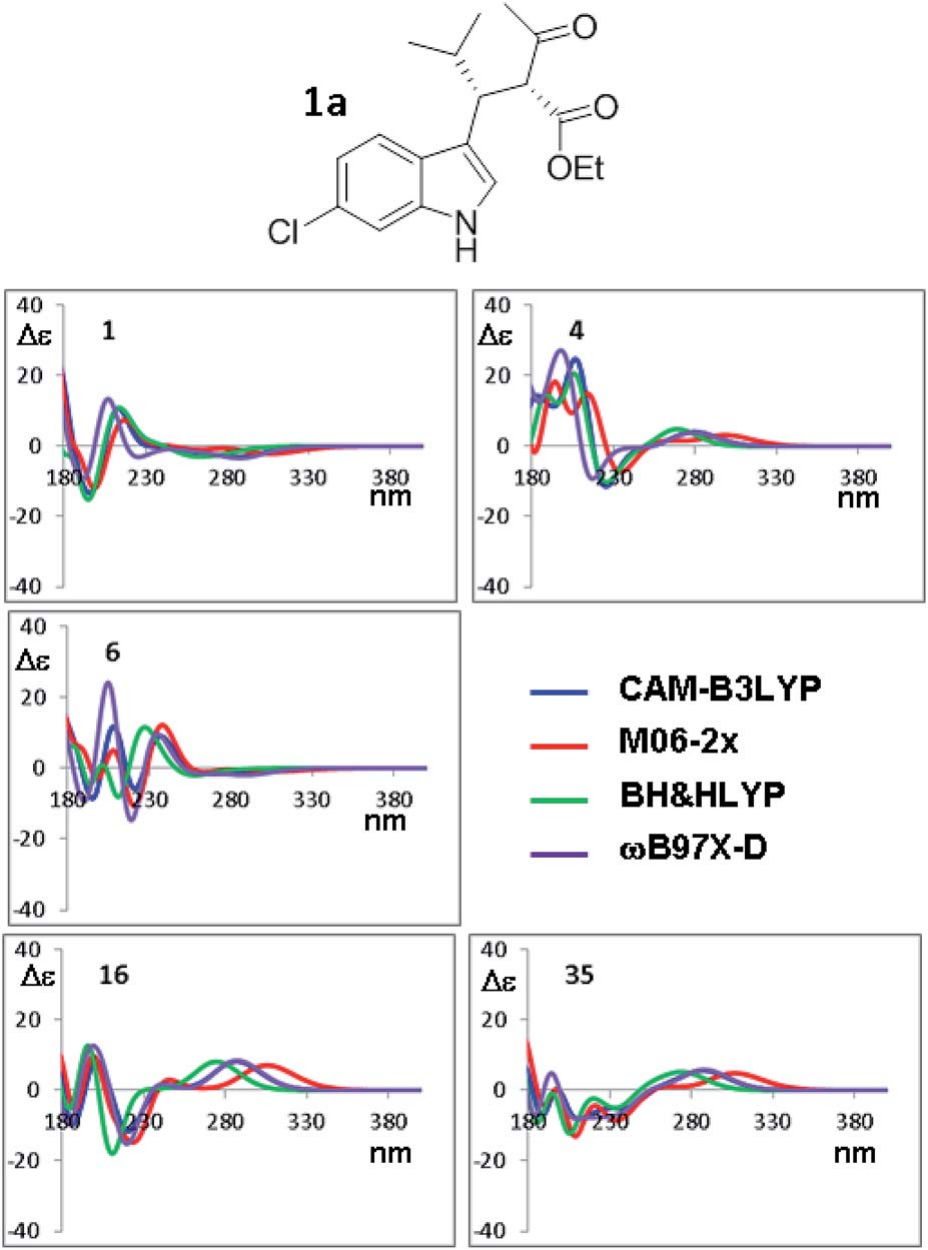
Selected ECD simulations for the conformations of 1a. The number in each quadrant indicates the conformation label, the four colored lines are the ECD spectra obtained with the four different functionals using the geometries obtained with PCM-B3LYP/6-311++G(2d,p). The simulations for all the conformations are reported in ESI.†

The orthogonal test compares the results when different TD-DFT functionals are used with the same input geometry (Fig. 9 shows the case of B3LYP optimization of compound 1a, the remaining simulations are reported in Fig. S18–S21 of ESI†). In some conformations, such as #1, the four simulations are almost superimposable, whereas in others cases (#8, #9, #16, #35) the low energy Cotton effect is calculated at very different wavelength. In some cases (#6 and #19) the shape is similar but the relative intensities of the Cotton effects are different. The same situation occurs for compound 1b (Fig. S22–S24 in ESI†). The largest effect is determined by the type of functional used in TD-DFT calculations, due to the peculiar features of each single functional. As pointed out many times,^34^ the use of multiple methods is a convenient and recommended way to achieve data redundancy, and to enhance calculations reliability.

The last factor for the successful simulation of the experimental ECD spectra is the conformational ratio employed when mixing the spectra of each conformation.^35^ The ECD simulations of the experimental spectra were obtained using the conformational ratios derived from Table 4 by Boltzmann weighted average at +25 °C (Fig. 10 of ESI†).

**Fig. 10:**
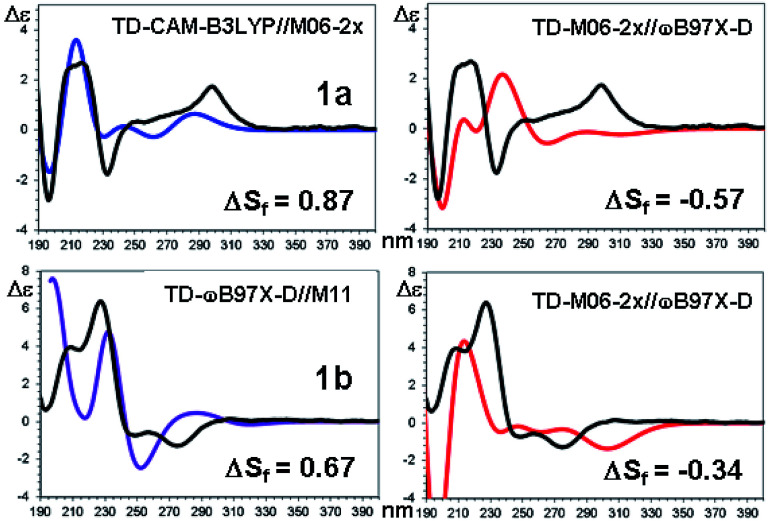
TD-DFT simulations (colored traces) of the experimental ECD spectrum (black trace) of 1a (top) and 1b (bottom). For each compound the best and the worse simulation are reported (see text for the definition of Δ*S*_f_ and ESI† for the full set of simulations with the related red shifts and scaling factors).

**Fig. 11:**
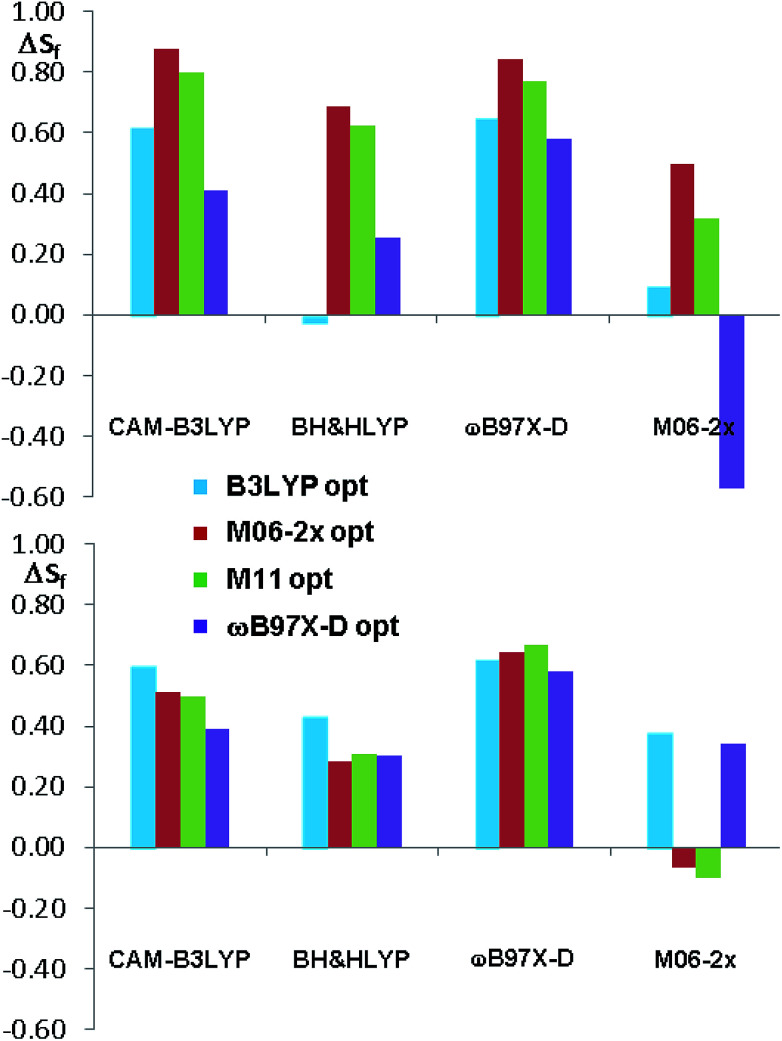
Summary of the Δ*S*_f_ values for the sixteen ECD simulations of compounds 1a and 1b. The different colors indicate the different geometry optimization methods.

The comparison of the simulated UV spectra with the experimental ones showed a very good agreement in all the simulations (Fig. S26–S29 of ESI†). The goodness of the fit between the simulated and the experimental ECD spectra was evaluated with SpecDis software,^5*e*,6*a*,36^ using the difference between the two similarity factors calculated for the correct enantiomer and that for the wrong one (Δ*S*_f_).||||This parameter has the same meaning of that defined as Δ in the SpecDis manual, p. 15.Fig. 10 reports the best and the worse simulations obtained for compounds 1a and 1b, whereas Fig. 11 reports the Δ*S*_f_ values for all the sixteen simulations (all the spectra comparison and the raw data provided by SpecDis are reported in Fig. S30 and S31 of ESI†).**The simulations of the UV spectra required a red-shift (Δ*λ*) of the calculated spectra between 20 and 25 nm, whereas the ECD spectra of 1a and 1b were better simulated by a smaller correction, with a significant improvement of the similarity factor.

As a whole, the TD-DFT simulations obtained by BH&HLYP and M06-2x are always the worse (as mean value of Δ*S*_f_), regardless of the optimization method used to evaluate the conformational ratio. In two cases of 1a and in three of 1b, the simulations for the opposite enantiomer had a better *S*_f_ score (right section of Fig. 10). On the other side, the TD-DFT simulations obtained by the two range-separated functionals CAM-B3LYP and ωB97X-D are in a much better agreement with the experimental spectra.^37^ In these two series the Δ*S*_f_ is always quite good (Δ*S*_f_ > 0.58 for both 1a and 1b) and the difference among the four available conformational ratios is small, being the ratio suggested by ωB97X-D optimization the worse. The influence of the different conformational ratio is particularly evident in the simulation of the broad positive band centered at 300 nm, that is calculated to be opposite in sign on changing the conformation. In this region, where the TD-DFT calculations should be more reliable, the conformational ratio suggested by B3LYP provides the best results. On the contrary, the B3LYP conformational ratio does not simulate well the high-energy region.

## Conclusions

The assignment of the AC of very flexible organic molecules by means of chiro-optical spectra supported by quantum-mechanical simulation is a challenging task because of the presence of many populated conformations. The results obtained in this study show that different functionals yield very similar geometries, that provide very similar TD-DFT simulations.

On the other hand, the relative energies derived from the optimization step are of primary importance to determine the final result, and the inclusion of solvent in the calculations plays a very large effect on the conformational ratio. B3LYP and M06-2x functionals provided the best results in terms of the conformational ratio. In the TD-DFT simulations the two range-separated CAM-B3LYP and ωB97X-D functionals provided the best results. This study confirms once more the importance of using many combinations of functionals and basis set for TD-DFT, and more than a single functional for the evaluation of the conformational ratio.

## Experimental section

### NMR spectra

NMR spectra were recorded using a spectrometer operating at 400 MHz for ^1^H and 100.6 for ^13^C. Chemical shifts are reported in ppm relative to TMS as the internal standard. The assignment of the ^13^C signals was obtained by means of DEPT, gs-HSQC and gs-HMBC spectra. The ^13^C spectra were acquired under proton decoupling conditions with 5.5 μs (60° tip angle) pulse width, 1 s acquisition time and 3 s delay time. NOE spectra were obtained at 600 MHz using the DPFGSE sequence and a 50 Hz wide selective pulse with a R-SNOB shape. The low temperature ^1^H NMR spectra were obtained at 600 MHz by using a flow of dry nitrogen through an heat exchanger immersed in liquid nitrogen and connected to the NMR probe by a vacuum-insulated transfer line. The ^1^H spectra were acquired using a 5 mm direct probe with a 9000 Hz spectral width, 2.0 μs (20° tip angle) pulse width, 3 s acquisition time and 1 s delay time. A shifted sine bell weighting function equal to the acquisition time (*i.e.*, 3 s) was applied before the Fourier transformation. Temperature calibrations were performed before the experiments, using a Cu/Ni thermocouple placed in an NMR tube filled with isopentane.

### UV and ECD spectra

UV and ECD spectra were recorded at +25 °C in far-UV HPLC-grade acetonitrile solutions. The concentrations of the samples were calibrated by dilution of a mother solution (1 × 10^−3^ M) to obtain a maximum absorbance of about 1.0 in the UV spectrum using a 0.2 cm path length. Final concentrations were 9.9 × 10^−5^ M for 1a and 9.3 × 10^−5^ M for 1b. The spectra were recorded on a Jasco J-810 spectropolarimeter in the 185–400 nm range at 50 nm min^−1^ as the average of 16 spectra. Δ*ε* is expressed as L mol^−1^ cm^−1^.

### DFT calculations

Ground state optimizations were obtained by DFT with the Gaussian 16 rev A.03 series of programs,^17^ using standard convergence parameters. The analysis of the vibrational frequencies for the optimized structures showed the absence of imaginary frequencies. The ECD spectra were calculated with TD-DFT using BH&HLYP, M06-2x, ωB97XD, CAM-B3LYP and the 6-311++G(2d,p) basis set. 50 discrete transitions were calculated for each conformation (lowest calculated wavelength about 160 nm). The ECD spectra were generated by convolution of Gaussian shaped lines (0.25 eV line width).^38^ The simulated spectra resulting from the Boltzmann averaged sum of the conformations were vertically scaled and red-shifted using SpecDis rev. 1.71 (ref. 36) to get the direct comparison with the experimental spectra.

### Materials

Compound 1a + 2a and 1b + 2b were prepared following a reported procedure.^7^

#### 1a + 2a

^1^H-NMR (400 MHz, CD_3_CN). 0.78 (d, 3H, *J* = 6.9 Hz), 0.84 (d, 3H, *J* = 6.9 Hz), 1.93 (s, 3H), 2.01 (m, 1H), 3.75 (s, 3H), 3.80 (dd, 1H, *J* = 11.9, 3.9 Hz), 4.19 (d, 1H, *J* = 11.9 Hz), 7.07 (dd, 1H, *J* = 8.5, 1.9 Hz), 7.10 (d, 1H, *J* = 2.5 Hz), 7.43 (d, 1H, *J* = 1.9 Hz), 7.63 (d, 1H, *J* = 8.5 Hz), 9.34 (bs, 1H). ^13^C-NMR (100.6 MHz, CD_3_CN). 16.9 (CH_3_), 21.6 (CH_3_), 28.6 (CH), 30.5 (CH_3_), 41.7 (CH), 52.4 (CH_3_), 64.0 (CH_3_), 111.3 (CH), 112.4 (C), 119.7 (CH), 120.7 (CH), 124.9 (CH), 127.1 (C), 127.5 (C), 136.4 (C), 169.7 (COOMe), 202.4 (COMe). HRMS (ESI-Orbitrap), calcd. for [C_17_H_21_NO_3_Cl]^+^ 322.12045; found 322.1209. Crystals suitable for X-ray diffraction analysis were obtained from a chloroform solution by evaporation (about 24 h).

#### 1b + 2b

^1^H-NMR (400 MHz, CD_3_CN). 0.80 (d, 3H, *J* = 6.7 Hz), 0.81 (d, 3H, *J* = 6.7 Hz), 1.93 (m, 1H), 2.27 (s, 3H), 3.35 (s, 3H), 3.79 (dd, 1H, *J* = 11.3, 4.7 Hz), 4.19 (d, 1H, *J* = 11.3 Hz), 7.06 (dd, 1H, *J* = 8.6, 2.0 Hz), 7.13 (d, 1H, *J* = 2.5 Hz), 7.42 (d, 1H, *J* = 2.0 Hz), 7.63 (d, 1H, *J* = 8.6 Hz), 9.30 (bs, 1H). ^13^C-NMR (100.6 MHz, CD_3_CN). 17.2 (CH_3_), 21.7 (CH_3_), 28.7 (CH), 30.4 (CH_3_), 41.4 (CH), 51.8 (CH_3_), 64.2 (CH_3_), 111.2 (CH), 113.1 (C), 119.5 (CH), 120.7 (CH), 126.9 (CH), 127.8 (C), 127.8 (C), 136.4 (C), 169.2 (COOMe), 203.2 (COMe). HRMS (ESI-Orbitrap), calcd. for [C_17_H_21_NO_3_Cl]^+^ 322.12045; found 322.1201. Crystals suitable for X-ray diffraction analysis were obtained from an acetonitrile solution by slow evaporation (about 10 days).

## Conflicts of interest

There are no conflicts to declare.

## Supplementary Material

RA-009-C9RA03526E-s001

RA-009-C9RA03526E-s002
