# Supplementary material for: Determination of the absolute configuration of conformationally flexible molecules by simulation of chiro-optical spectra: a case study
Source: RSC Adv. 2019 Jun 10;9(32):18165–75. doi: 10.1039/c9ra03526e (PMC9064610; doi:10.1039/c9ra03526e)
Supplement: RA-009-C9RA03526E-s001 [file RA-009-C9RA03526E-s001.pdf]

Electronic Supplementary Material (ESI) for RSC Advances.
This journal is © The Royal Society of Chemistry 2019

Determination of the Absolute Configuration of Conformationally Flexible Molecules by Simulation of Chiro-optical Spectra: a Case Study.

Michele Mancinelli[a], Roberta Franzini[b], Andrea Renzetti[c], Emanuela Marotta[a],
Claudio Villlani*[b] and Andrea Mazzanti*[a]

SUPPORTING INFORMATION

Summary:

Pages 2-35: Figures S1-S29 and Tables S1-S3

Pages 36-48: X-ray data of **1a** and **2b**

Pages 49-219 XYZ coordinates for all the optimized structures.

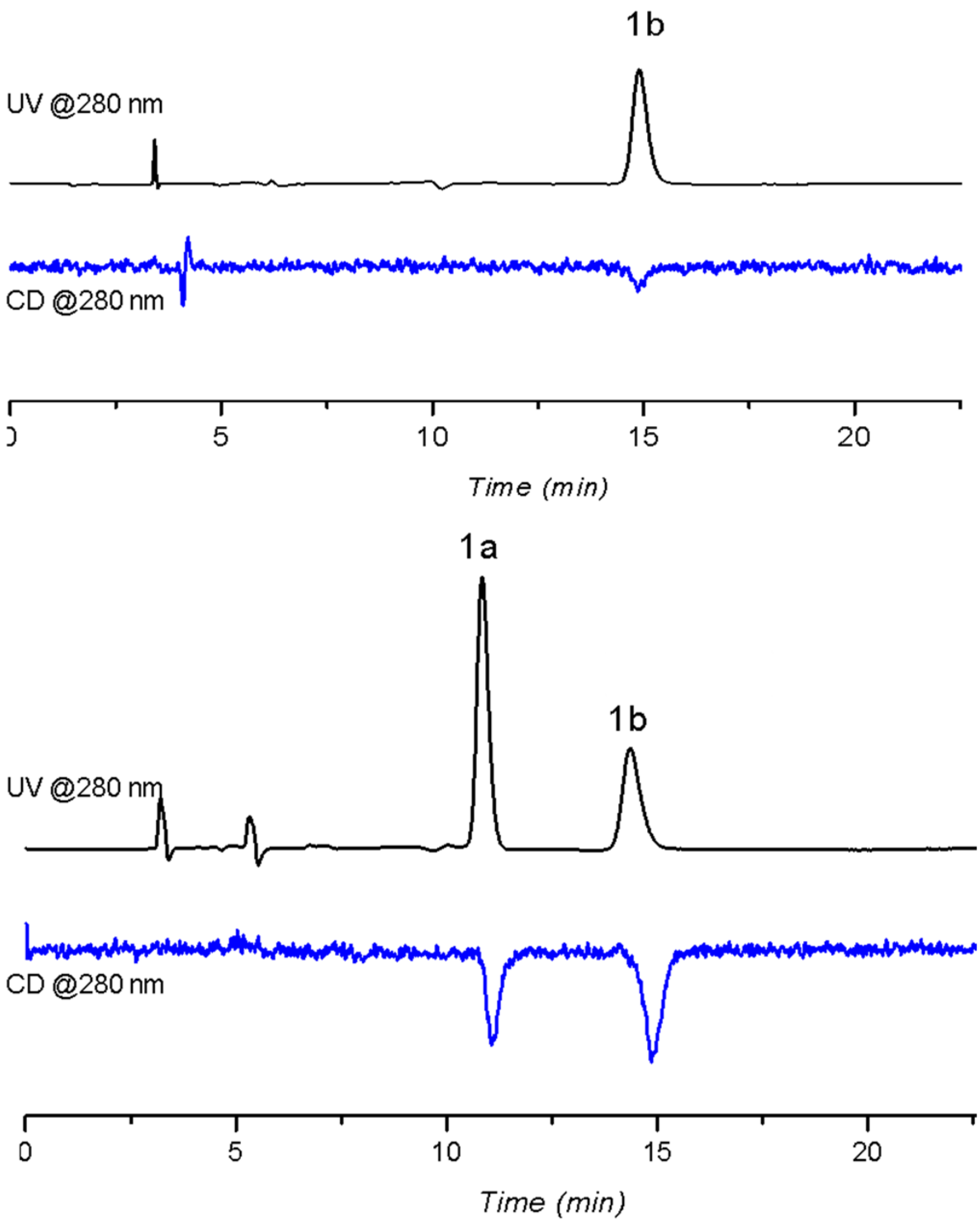

Figure S1. Top: HPLC chromatogram (Daicel Chiralpak IA, hexane/2-propanol 93:7, 1 mL/min, 25°C) of compound **1b** after semi-preparative HPLC separation. Bottom: HPLC chromatogram of the same sample after 18 h stirring in a solution of Et_3N 0.1 M in ethanol.

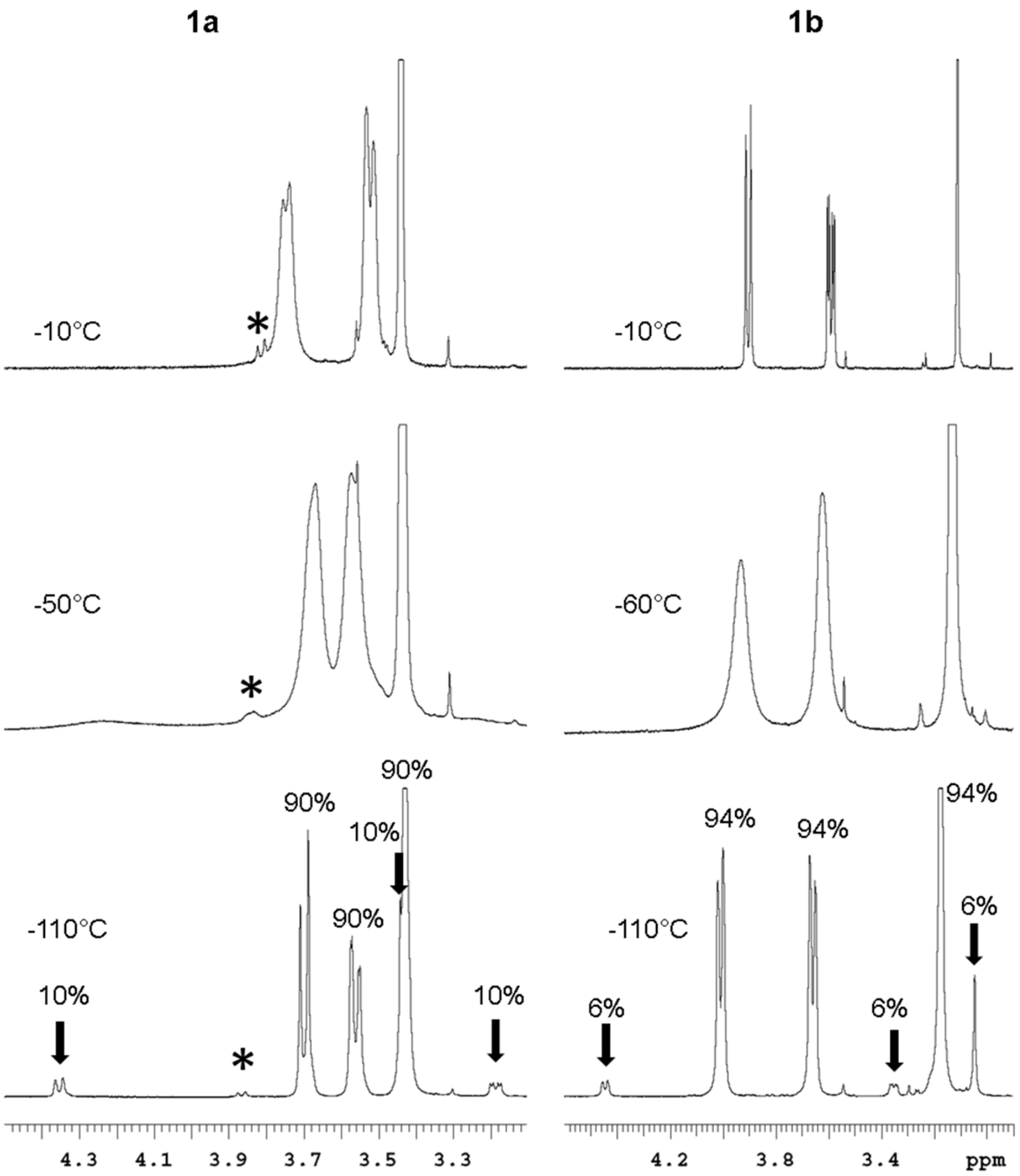

Figure S2. Variable temperature ^{1}H-NMR spectra of compounds **1a/2a** and **1b/2b** (600 MHz in $CDFCl_2$). The two spectra at –110°C show the formation of the *sp*/*ap* conformers due to the frozen rotation around the indole—CH bond.

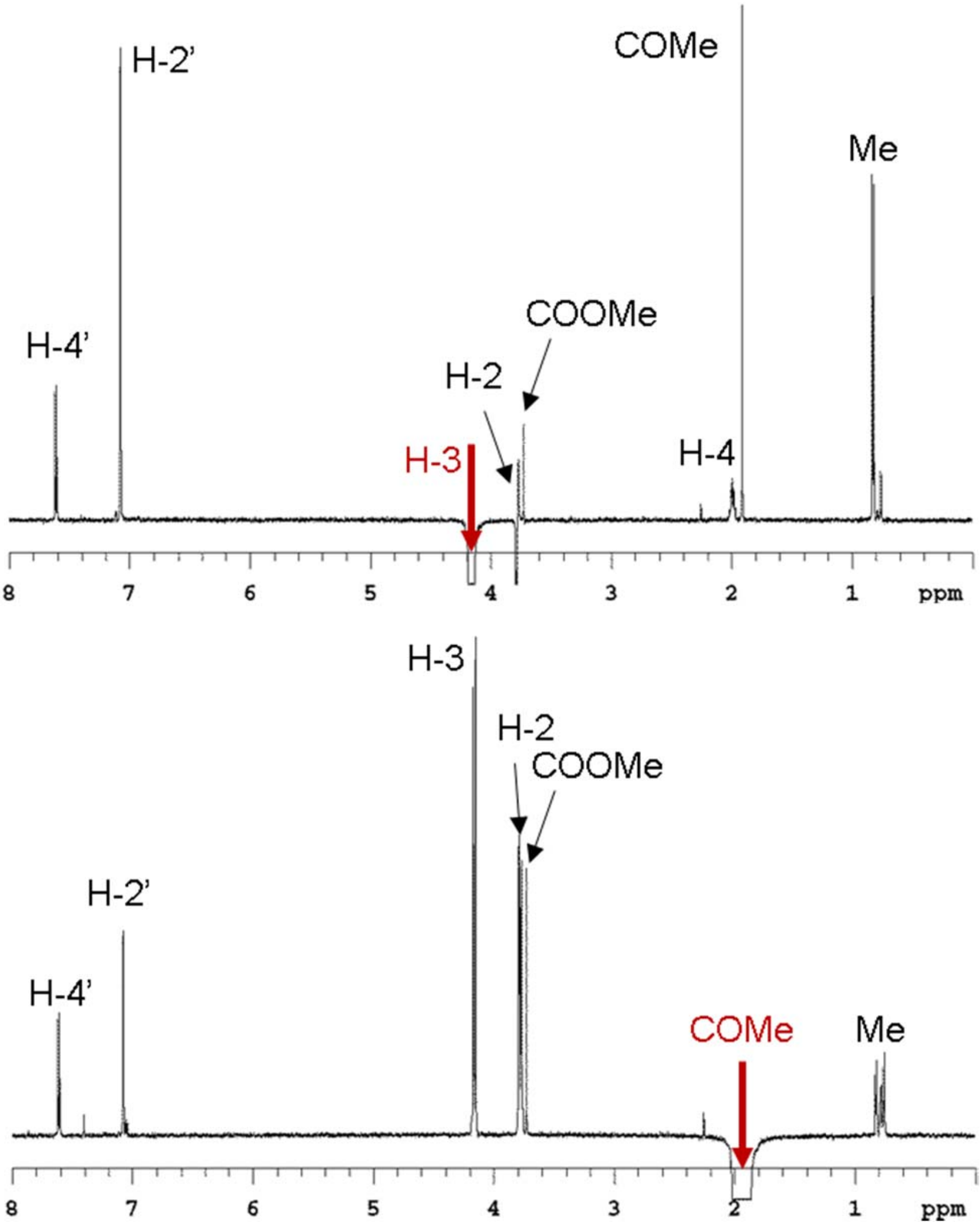

Figure S3. NOE spectra of **1a/2a** (600 MHz in CD_3CN, +25 °C). Top: saturation of the benzylic H3 signal; bottom: saturation of the COMe signal.

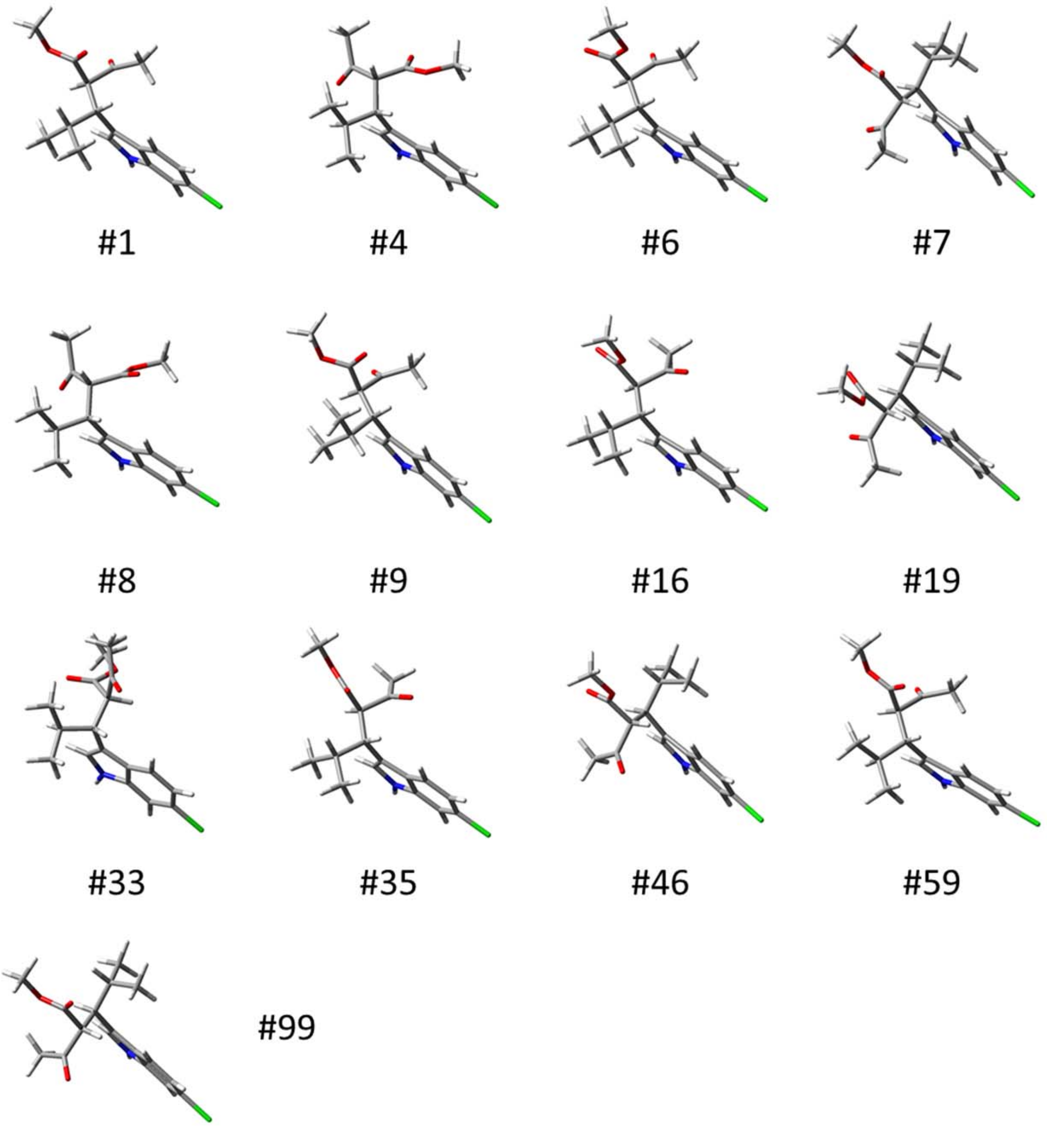

Figure S4. 3D geometries of the thirteen conformations of compound **1a** after optimization at the B3LYP/6-31G(d) level of theory. The geometric parameters and relative energies are reported in Table 1 of the main text.

Table S1. NMR coupling constants calculations for **1a**. The populations for each functional were derived from the data of Table 4. The ^3J calculations were obtained at the GIAO-B3LYP/6-311++G(2d,p)//B3LYP/6-31G(d). level. Experimental values were 3.9 and 11.9 Hz.

Conf #	Calcd. H2-H_{iPr} ^3J (Hz)	Calcd. H2-H3 ^3J (Hz)	B3LYP			M06-2x			M11			ωB97X-D		
			Pop %	J (Hz)[a]	J (Hz)[a]	Pop %	J (Hz)[a]	J (Hz)[a]	Pop %	J (Hz)[a]	J (Hz)[a]	Pop %	J (Hz)[a]	J (Hz)[a]
1	3.1	13.7	22.4	0.69	3.06	27.4	0.84	3.75	31.4	0.97	4.30	37.4	1.15	5.13
4	11.2	6.0	1.3	0.15	0.08	16.8	1.88	1.00	17.1	1.92	1.02	2.2	0.25	0.13
6	3.3	14.1	6.1	0.20	0.86	12.4	0.41	1.74	16.2	0.54	2.28	18.7	0.62	2.63
7	3.6	13.2	2.0	0.07	0.26	2.2	0.08	0.29	1.6	0.06	0.21	4.1	0.15	0.54
8	11.4	5.1	0.9	0.10	0.05	4.5	0.51	0.23	6.2	0.71	0.32	1.2	0.14	0.06
9	2.7	13.3	1.1	0.03	0.15	3.7	0.10	0.49	4.4	0.12	0.58	2.0	0.05	0.26
16	3.7	13.2	18.6	0.68	2.45	10.3	0.38	1.36	8.1	0.30	1.07	6.8	0.25	0.90
19	3.6	13.2	4.2	0.15	0.55	3.6	0.13	0.47	2.2	0.08	0.29	6.5	0.23	0.85
33	11.6	3.1	0.3	0.03	0.01	0.3	0.04	0.01	0.4	0.05	0.01	0.1	0.01	0.00
35	3.7	13.1	37.1	1.38	4.87	10.3	0.38	1.35	6.1	0.23	0.80	9.7	0.36	1.27
46	3.1	13.8	2.4	0.07	0.33	3.4	0.11	0.47	2.7	0.08	0.37	6.9	0.21	0.95
59	10.6	11.6	3.2	0.34	0.37	4.3	0.45	0.49	2.9	0.30	0.33	2.6	0.28	0.31
99	4.4	0.1	0.5	0.02	0.00	0.9	0.04	0.00	0.8	0.04	0.00	1.8	0.08	0.00
Boltzmann weighted calcd. ^3J values				**3.9**	**13.0**		**5.4**	**11.7**		**5.4**	**11.6**		**3.8**	**13.0**
Experimental values				**3.9**	**11.9**		**3.9**	**11.9**		**3.9**	**11.9**		**3.9**	**11.9**

[a] These values are the contributions to the weighted *J* value of each conformation, i.e. the calculated *J* value multiplied for the population.

Table S2 Relative energies of the best thirteen conformations of **1a** calculated with B3LYP using two different basis sets, with or without the solvent (IEF-PCM). Relative energies in kcal/mol. The columns reporting the enthalpies (H°) are those of table 3 of the main text.

B3LYP/6-31G(d)			
**Conf. #**	**E**	**H°**	**G°**
1	0.00	0.00	0.00
4	0.28	0.11	0.25
8	0.89	0.76	0.90
9	1.00	1.04	1.53
35	1.31	1.16	0.47
16	1.34	1.21	0.69
59	1.33	1.22	1.57
19	1.29	1.25	1.29
7	1.56	1.56	1.50
6	1.68	1.68	1.75
33	1.94	1.76	1.89
46	1.90	1.90	1.93
99	3.74	3.75	3.89

B3LYP/6-311++G(2d,p)			
**Conf. #**	**E**	**H°**	**G°**
1	0.00	0.00	0.00
8	1.76	0.97	1.52
4	1.27	1.09	1.17
16	1.52	1.40	0.87
9	1.41	1.42	1.65
35	1.63	1.47	0.92
7	1.84	1.49	1.66
59	1.77	1.65	1.59
19	1.73	1.68	1.60
6	1.71	1.70	1.63
46	2.04	2.03	2.06
33	2.55	2.38	2.40
99	3.93	3.90	3.80

PCM-B3LYP/6-31G(d)			
**Conf. #**	**E**	**H°**	**G°**
35	0.00	0.00	0.00
1	0.39	0.51	1.27
16	0.47	0.55	1.12
4	1.22	1.17	1.79
19	1.05	1.21	1.73
8	1.56	1.55	2.30
6	1.41	1.59	2.47
46	1.51	1.68	2.49
7	1.59	1.72	2.58
59	1.83	1.85	2.74
9	1.80	1.95	3.03
33	2.45	2.44	2.23
99	2.63	2.84	3.64

PCM-B3LYP/6-311++G(2d,p)			
**Conf. #**	**E**	**H°**	**G°**
35	0.00	0.00	0.00
1	0.24	0.30	0.61
16	0.42	0.41	0.74
6	1.02	1.07	1.20
19	1.23	1.29	1.64
59	2.09	1.45	3.32
46	1.54	1.62	2.02
7	1.70	1.74	1.92
4	2.06	1.97	2.49
9	2.05	2.09	2.50
8	2.30	2.20	2.71
99	2.39	2.52	2.87
33	3.11	2.95	3.27

Table S3. Comparison between two different solvation model in the case of **1a**. The optimizations using the SMD model were performed for the conformations that were found within 1 kcal/mol from the global minimum of the PCM calculations. Relative energies in kcal/mol as ZPE corrected enthalpies, using the 6-311++G(2d,p) basis set.

	PCM model				SMD model			
conf #	B3-LYP	M06-2x	M11	ωB97XD	B3LYP	M06-2x	M11	ωB97XD
35	**0.00**	**0.58**	**0.97**	**0.80**	**0.00**	**0.22**	**0.69**	**0.40**
1	**0.30**	**0.00**	**0.00**	**0.00**	**0.41**	**0.00**	**0.00**	**0.00**
16	**0.41**	**0.58**	**0.80**	1.01	**0.29**	**0.25**	**0.39**	**0.45**
6	1.07	**0.47**	**0.39**	**0.41**		**0.57**	**0.40**	
19	1.29	1.20	1.57	1.04				
59	1.45	1.10	1.42	1.57				
46	1.62	1.23	1.46	1.00				
7	1.74	1.49	1.78	1.31				
4	1.97	**0.29**	**0.36**	1.67		**0.41**	**0.31**	
9	2.09	1.19	1.17	1.74				
8	2.20	1.07	0.96	2.04				
99	2.52	1.99	2.16	1.81				
33	2.95	2.66	2.60	3.48				

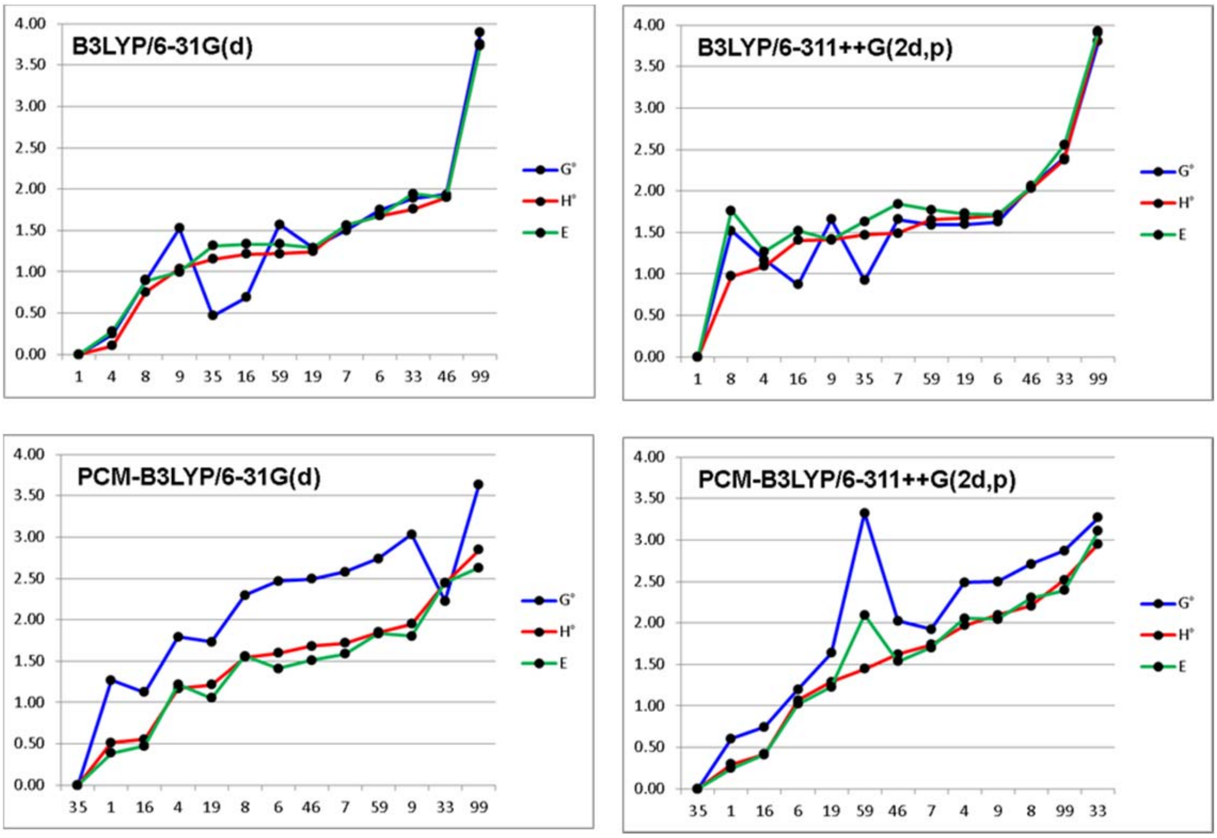

Figure S5. Relative energies (in kcal/mol) of the thirteen conformations of compound **1a** calculated with various optimization methods. Green line = relative energy in terms of uncorrected internal energy (E); red line = ZPE-corrected enthalpy (H°); blue line = ZPE-corrected Gibbs free energy in standard conditions (G°). Conformations are arranged on abscissa in increasing order of H°. The bottom-right quadrant is also reported in the main text.

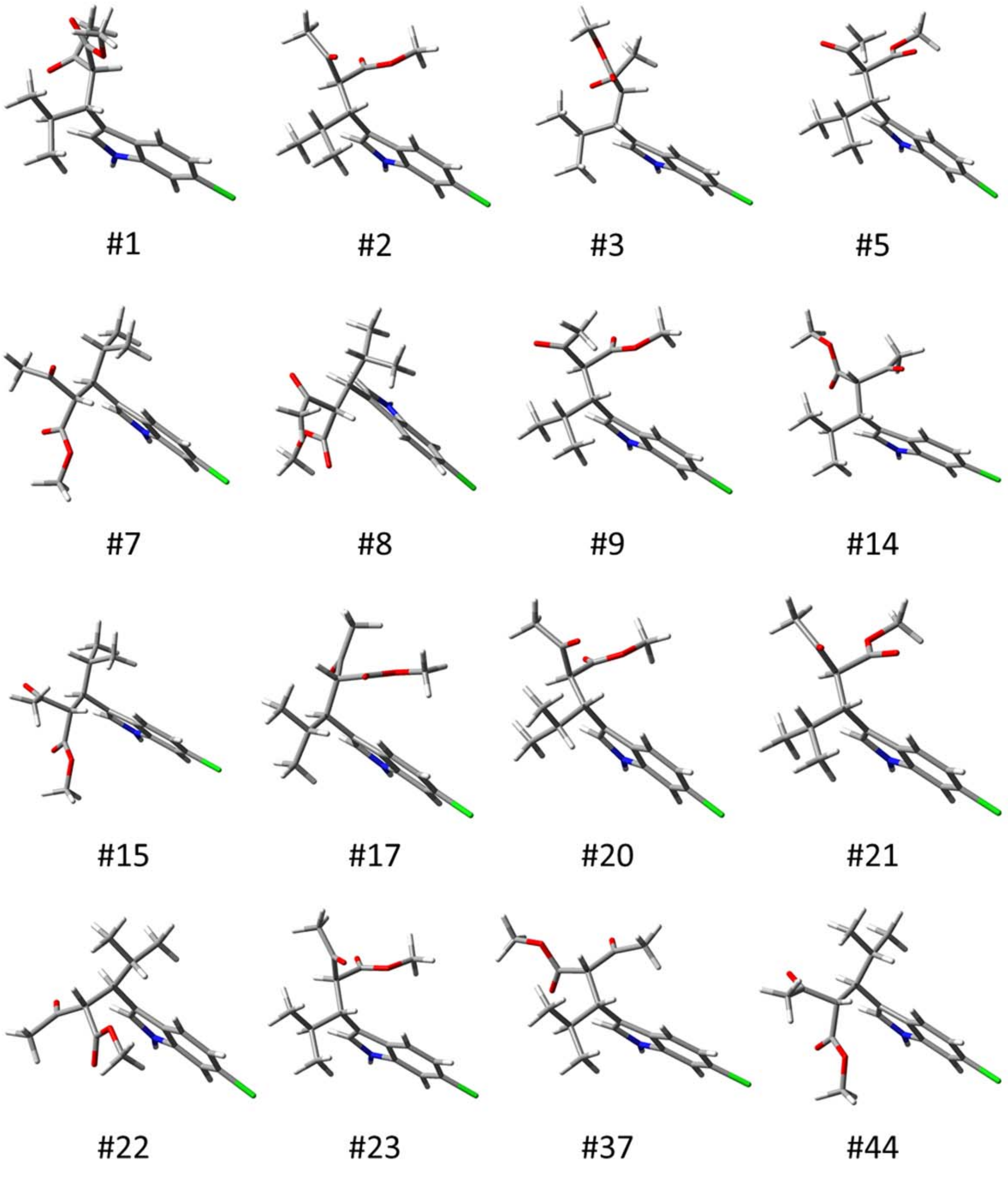

Figure S6. 3D geometries of the sixteen conformations of compound **1b** after optimization at the B3LYP/6-31G(d) level of theory. The geometric parameters and relative energies are reported in Table 2 of the main text.

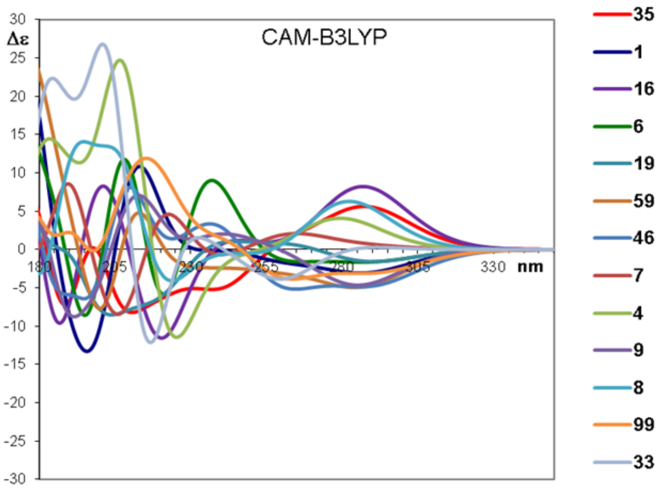

Figure S7. Example of ECD simulations of the thirteen conformations of **1a**, obtained at the TD-CAM-B3LYP/6-311++G(2d,p)//PCM-B3LYP/6-311++G(2d,p) level.

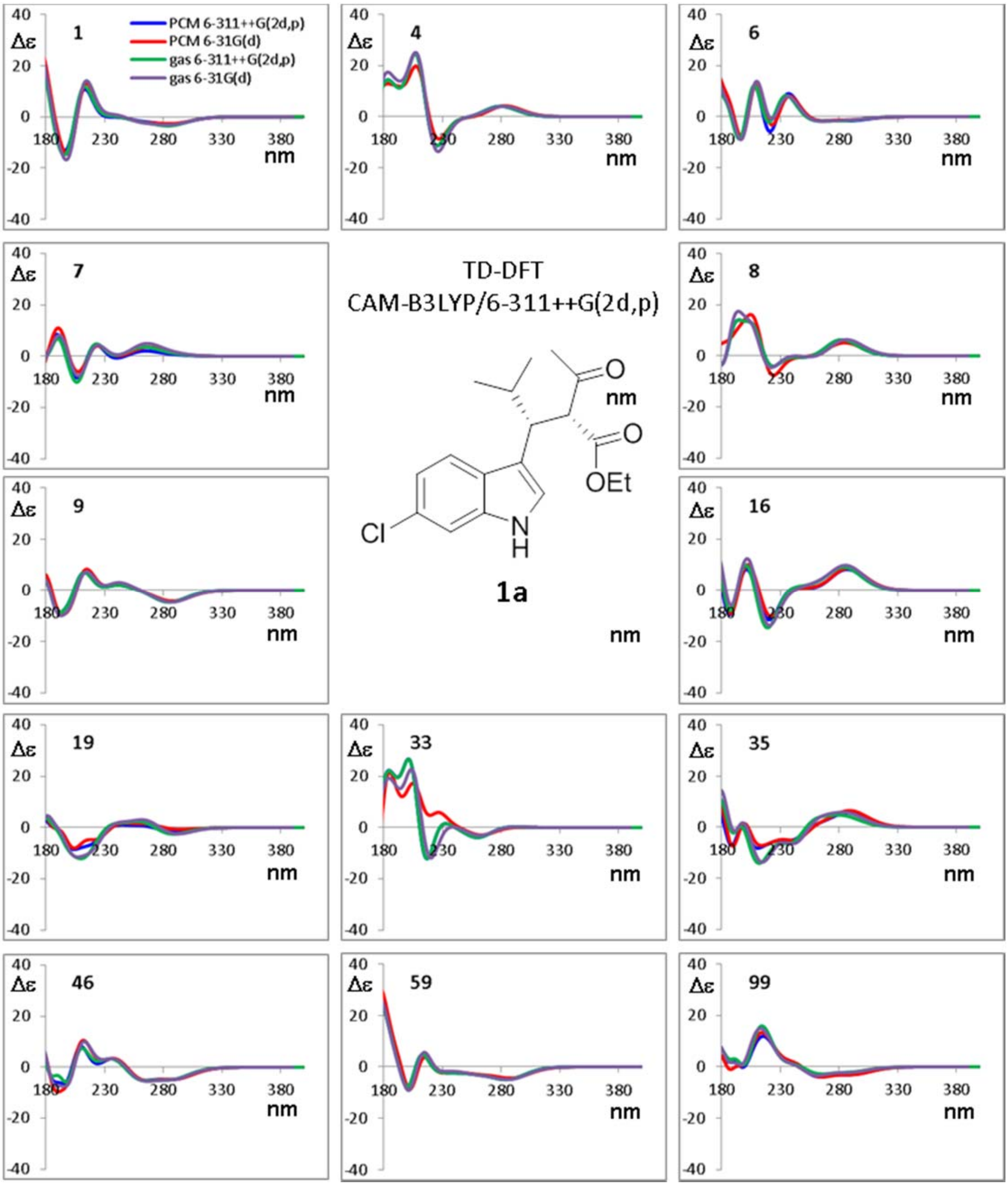

Figure S8. TD-DFT simulations obtained at the CAM-B3LYP/6-311++G(2d,p) level using the four geometries optimized by B3LYP, using the 6-31G(d) and 6-311++G(2d,p) basis sets, in both the gas phase and including the solvent acetonitrile.

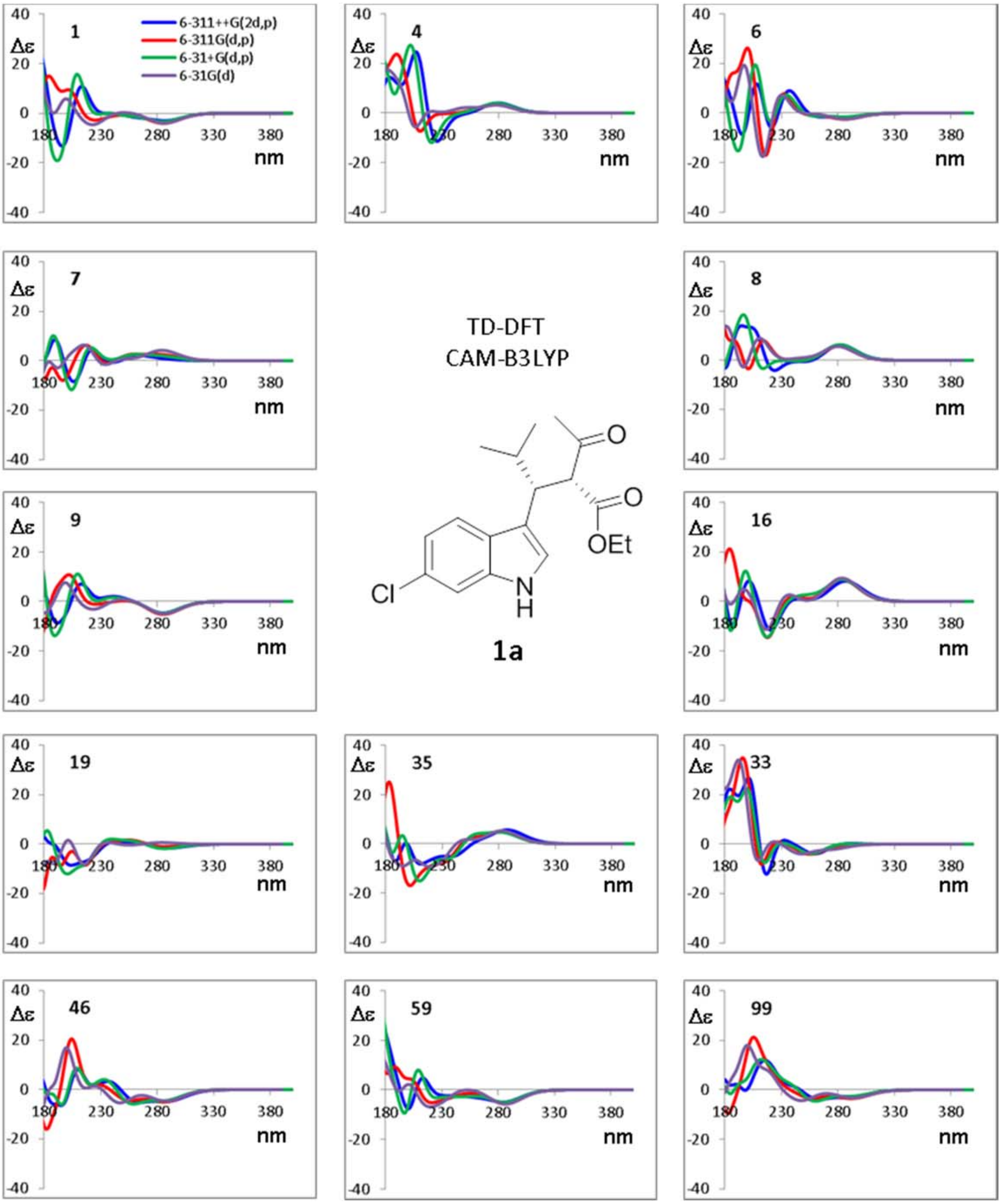

Figure S9. ECD simulation for the conformations of **1a**. The number in each quadrant is the conformation label; the four colored lines are the ECD spectra obtained by TD-DFT at the CAM-B3LYP level of theory using four basis sets (top-left quadrant). Optimized geometries at the PCM-B3LYP/6-311++G(2d,p) level.

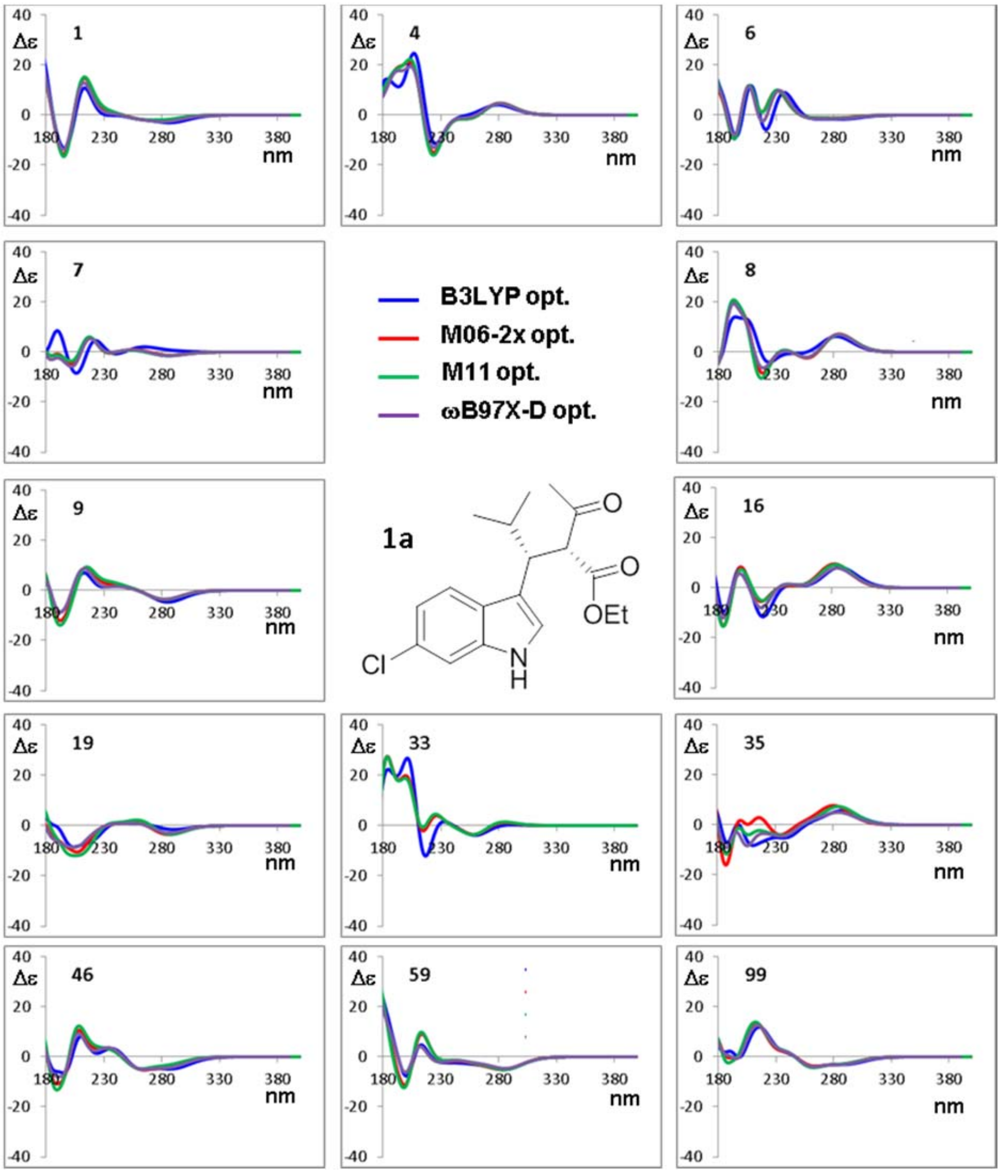

Figure S10. ECD simulation for the conformations of **1a**. The number in each quadrant is the conformation label; the four colored lines are the ECD spectra obtained by TD-DFT at the B3LYP/6-311++G(2d,p) level with the four different optimized geometries.

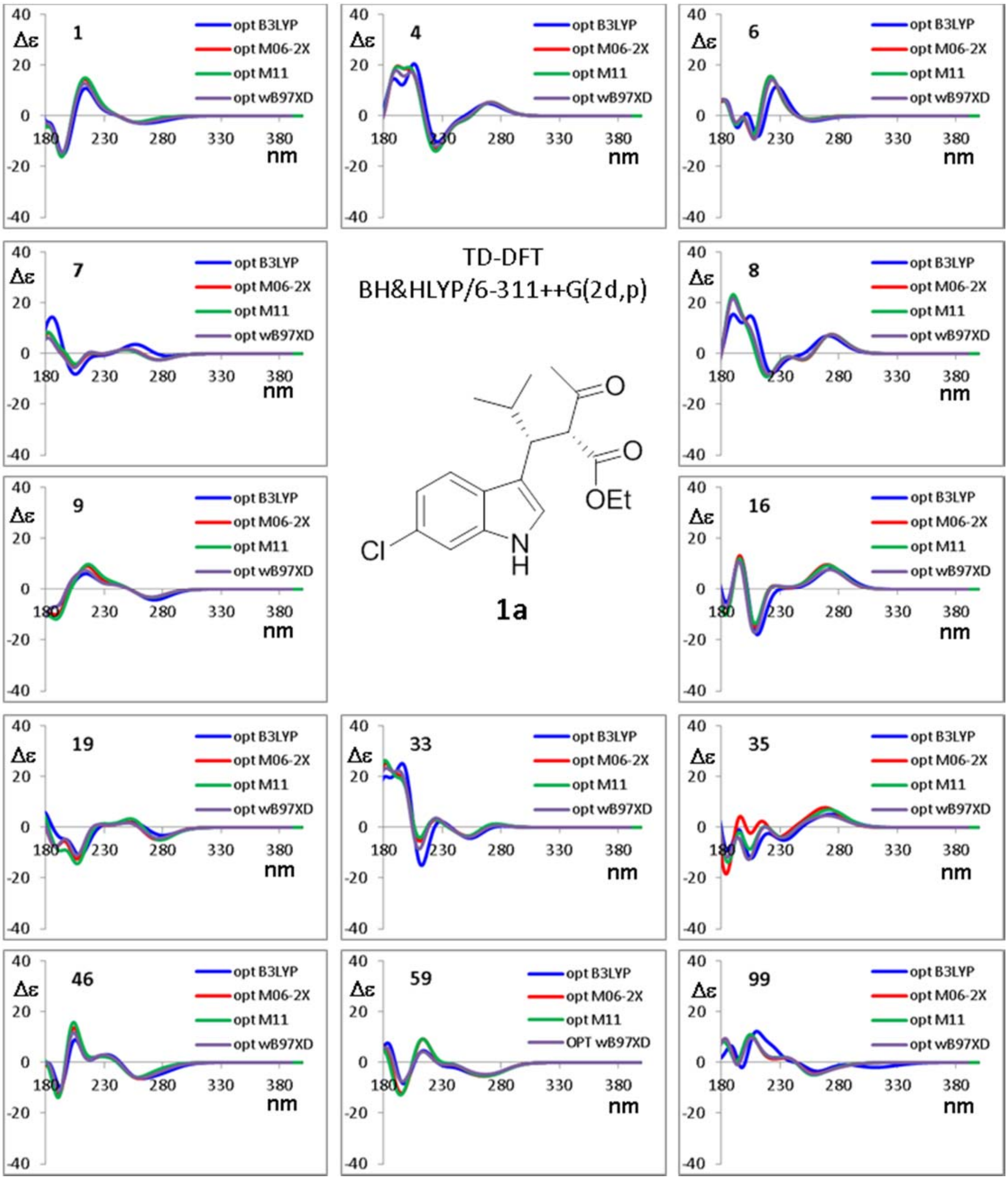

Figure S11. ECD simulation for the conformations of **1a**. The number in each quadrant is the conformation label; the four colored lines are the ECD spectra obtained by TD-DFT at the BH&HLYP/6-311++G(2d,p) level with the four different optimized geometries.

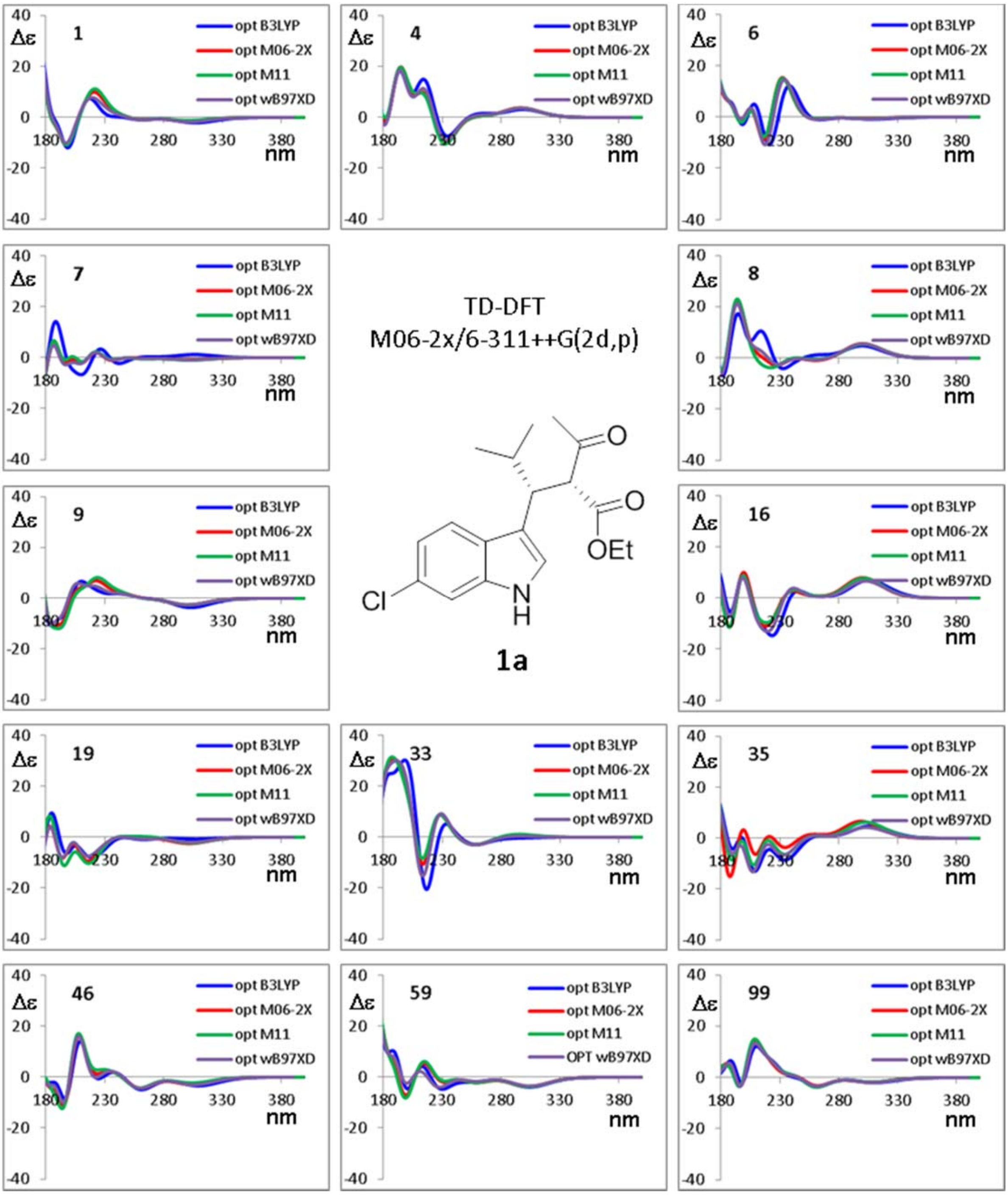

Figure S12. ECD simulation for the four conformations of **1a**. The number in each quadrant is the conformation label; the four colored lines are the ECD spectra obtained by TD-DFT at the M06-2x/6-311++G(2d,p) level with the four different optimized geometries..

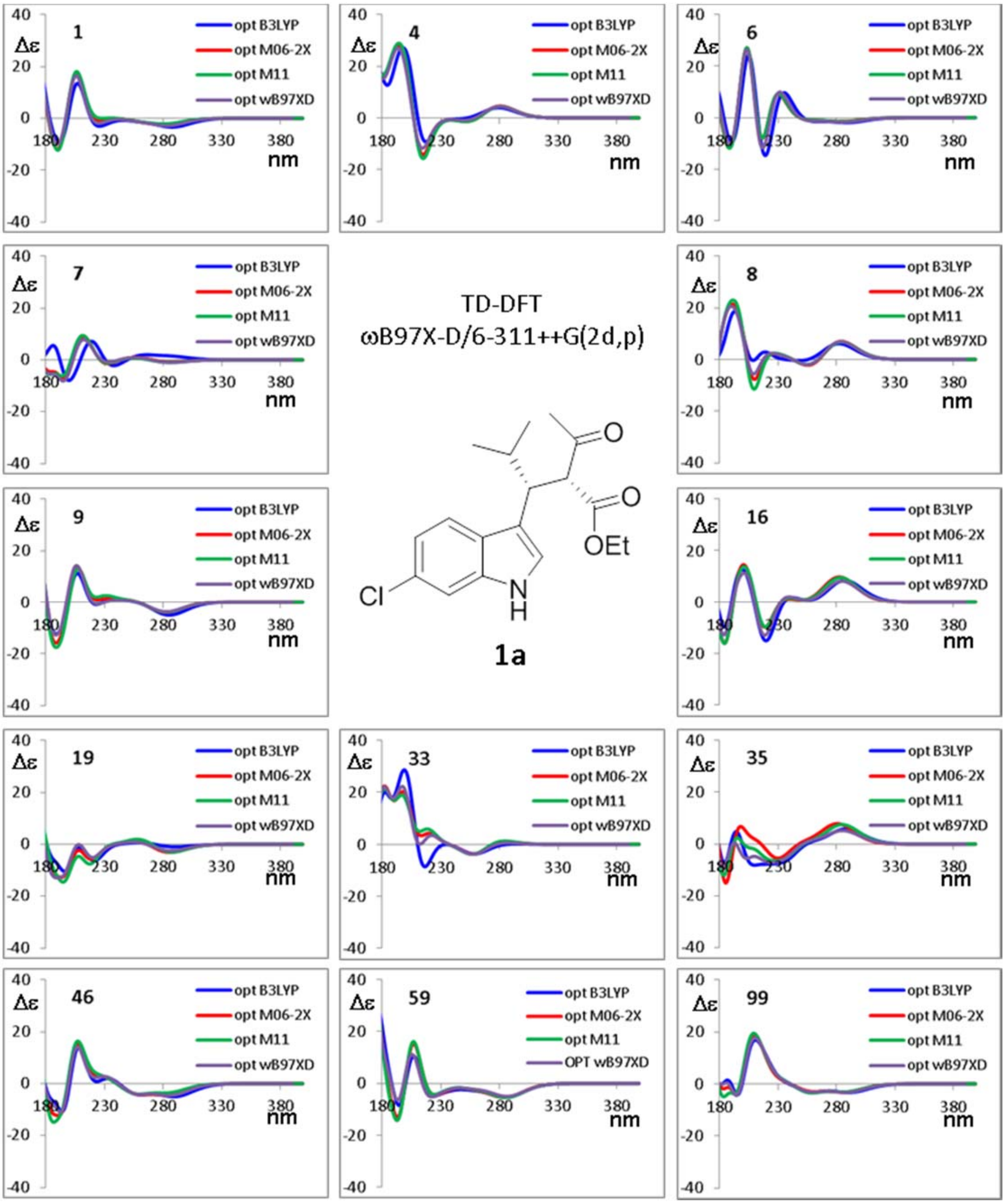

Figure S13. ECD simulation for the four conformations of **1a**. The number in each quadrant is the conformation label; the four colored lines are the ECD spectra obtained by TD-DFT at the ωB97X-D/6-311++G(2d,p) level with the four different optimized geometries.

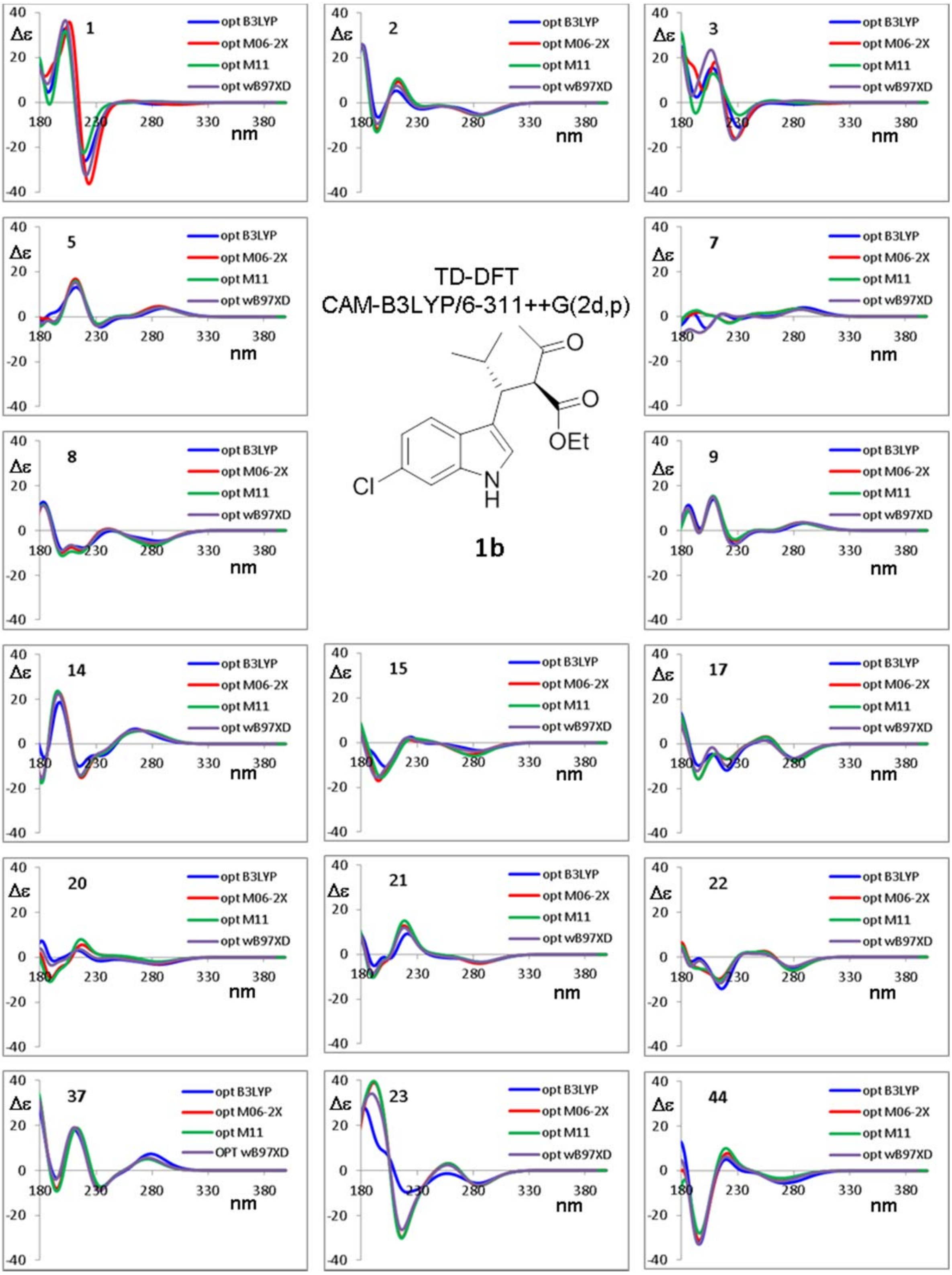

Figure S14. ECD simulation for the conformations of **1b**. The number in each quadrant is the conformation label; the four colored lines are the ECD spectra obtained by TD-DFT at the CAM-B3LYP/6-311++G(2d,p) level with the four different optimized geometries.

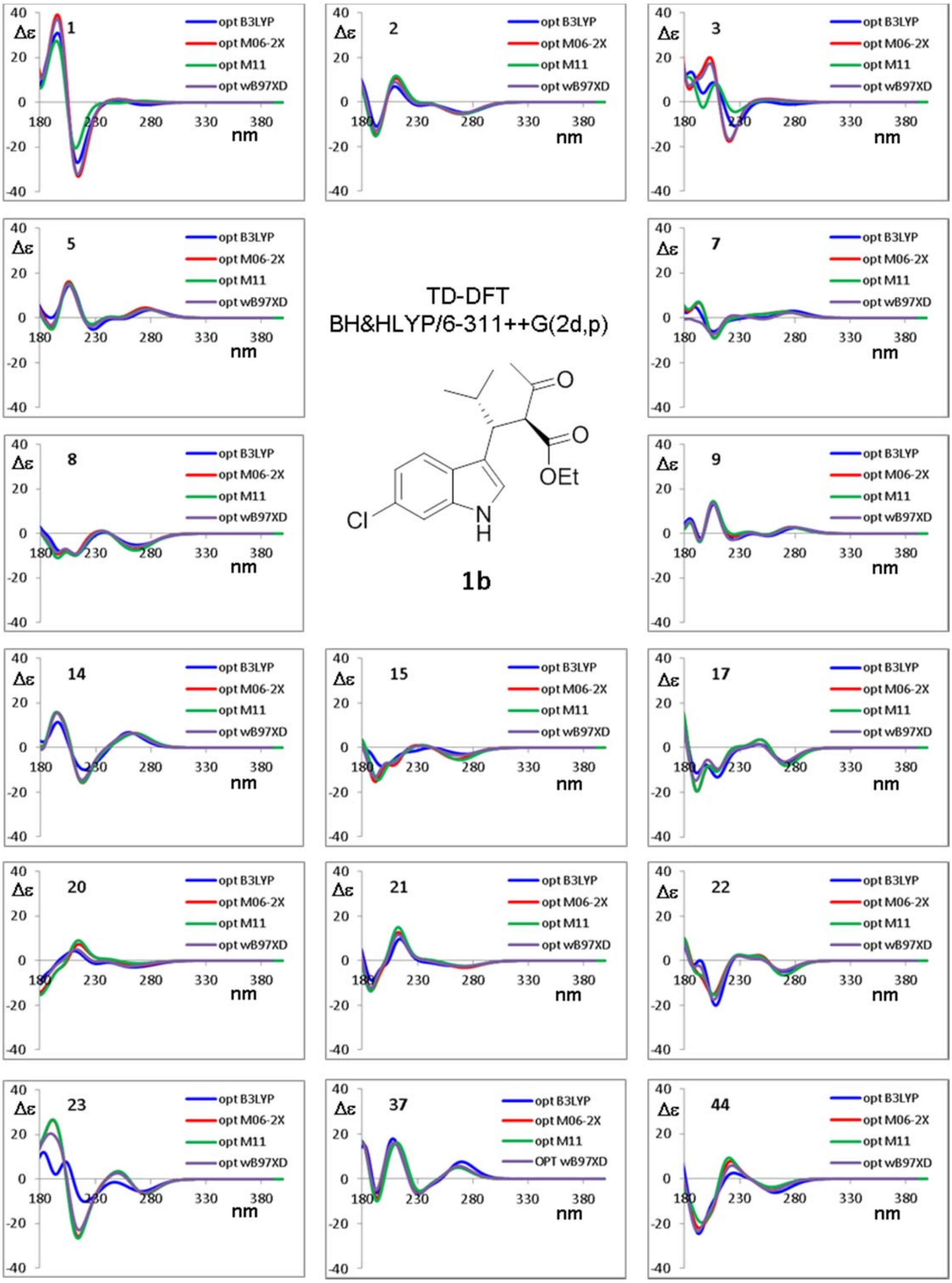

Figure S15. ECD simulation for the conformations of **1b**. The number in each quadrant is the conformation label; the four colored lines are the ECD spectra obtained by TD-DFT at the BH&HLYP/6-311++G(2d,p) level with the four different optimized geometries.

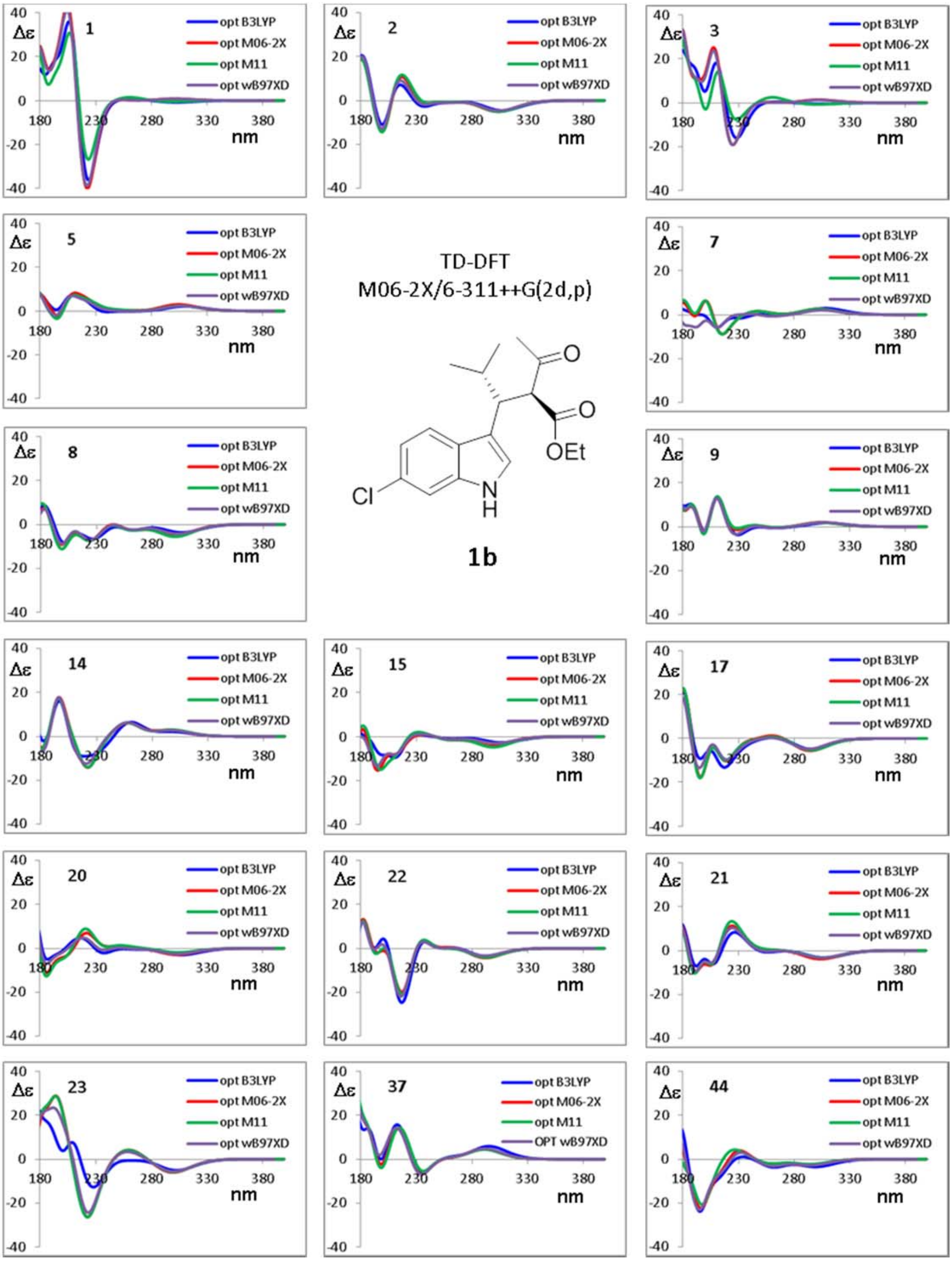

Figure S16. ECD simulation for the conformations of **1b**. The number in each quadrant is the conformation label; the four colored lines are the ECD spectra obtained by TD-DFT at the M06-2x/6-311++G(2d,p) level with the four different optimized geometries..

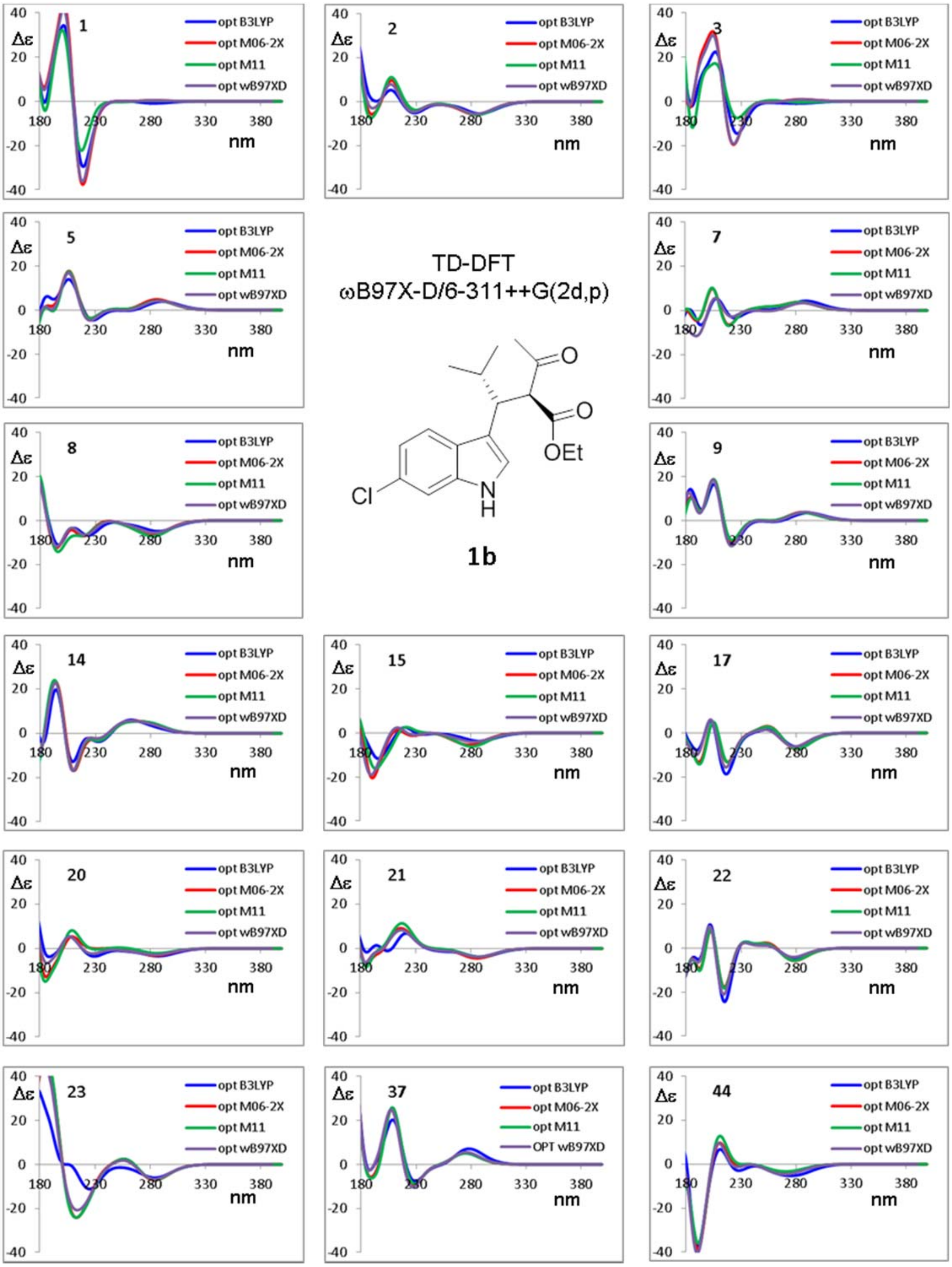

Figure S17. ECD simulation for the conformations of **1b**. The number in each quadrant is the conformation label; the four colored lines are the ECD spectra obtained by TD-DFT at the ωB97X-D/6-311++G(2d,p) level with the four different optimized geometries..

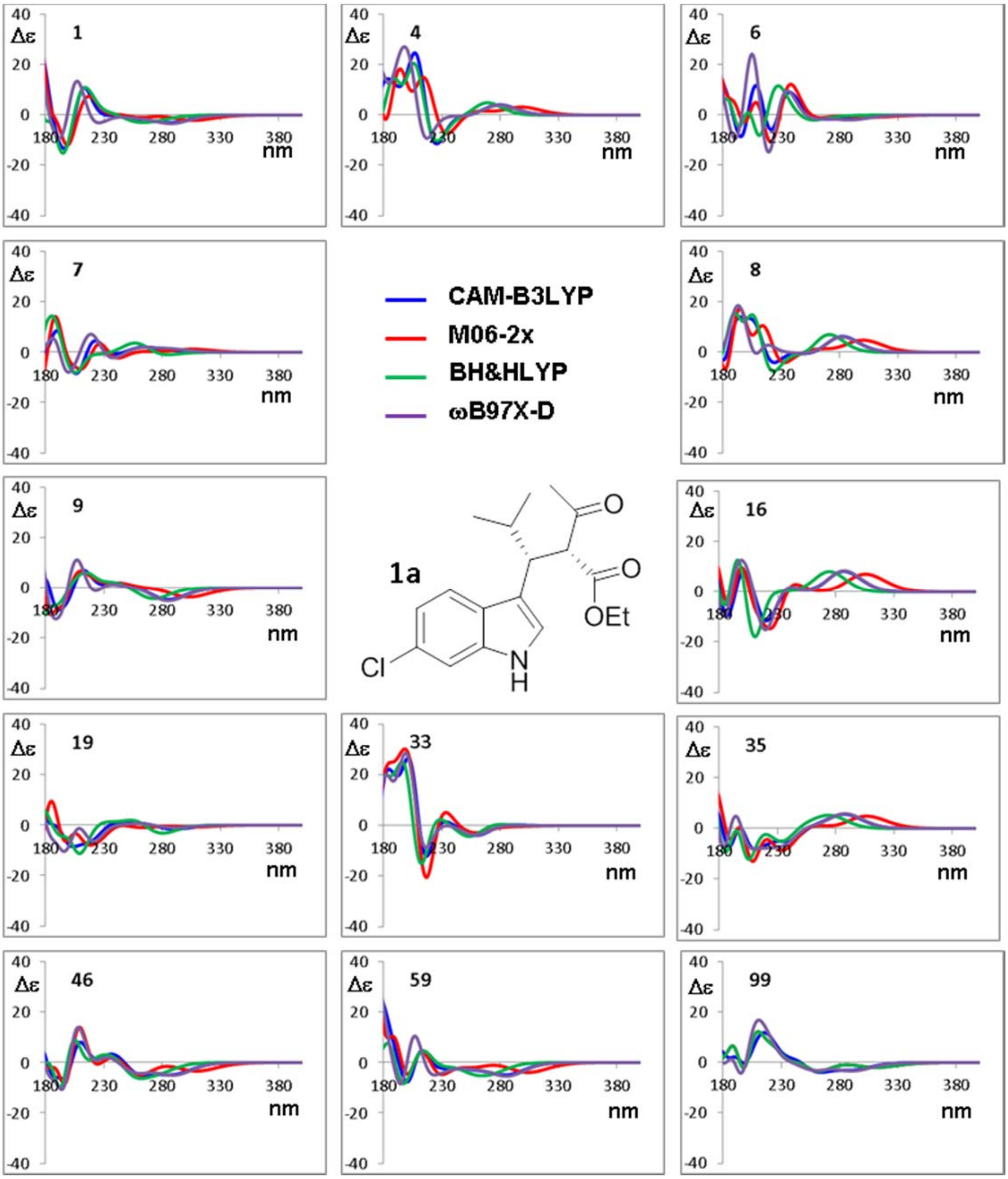

Figure S18. ECD simulation for the conformations of **1a**. The number in each quadrant indicates the conformation label; the four colored lines are the ECD spectra calculated with the four different functionals using the geometries obtained by PCM-B3LYP/6-311++G(2d,p).

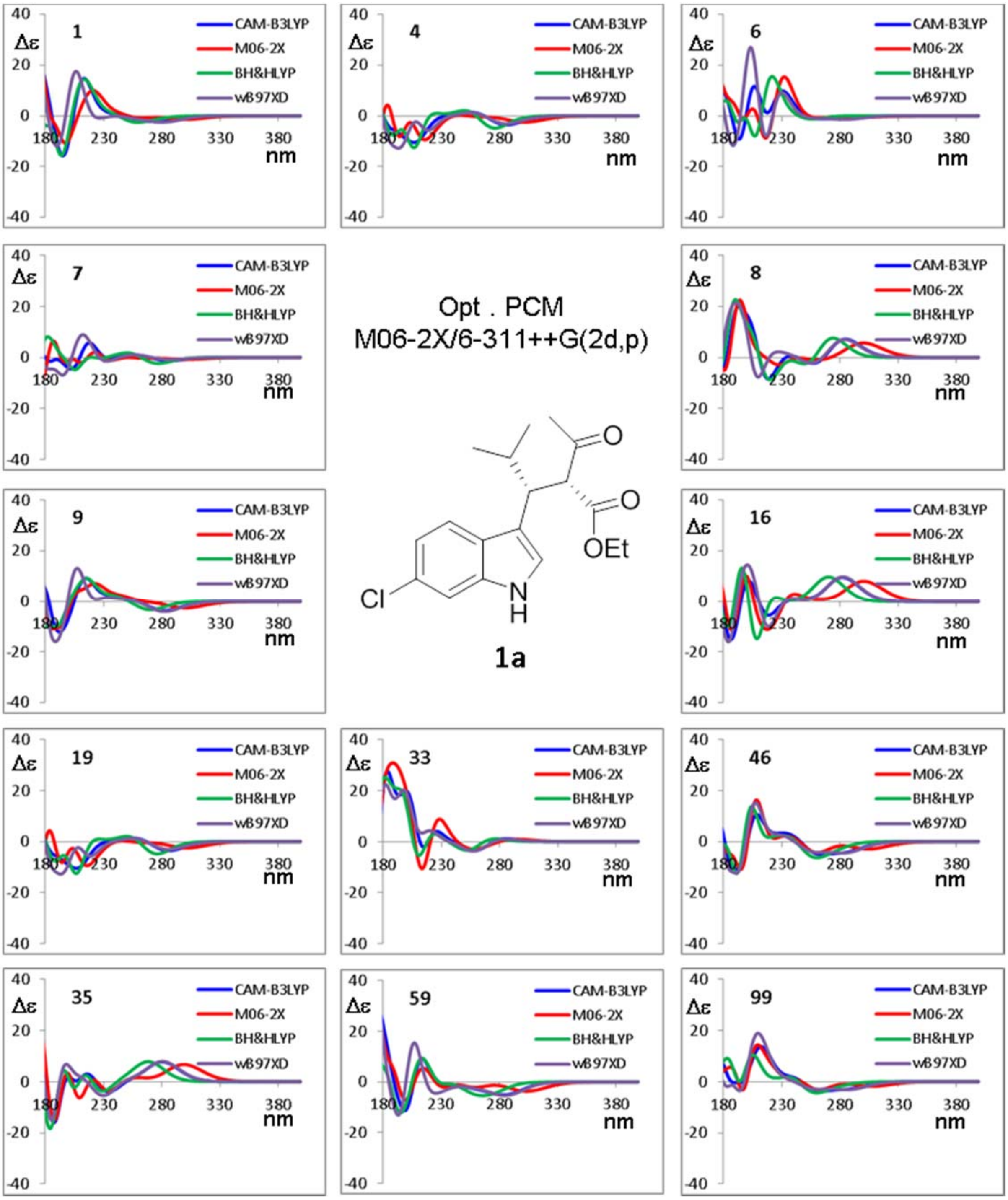

Figure S19. ECD simulation for the conformations of **1a**. The number in each quadrant indicates the conformation label; the four colored lines are the ECD spectra calculated with the four different functionals using the geometries obtained by PCM-M06-2x/6-311++G(2d,p).

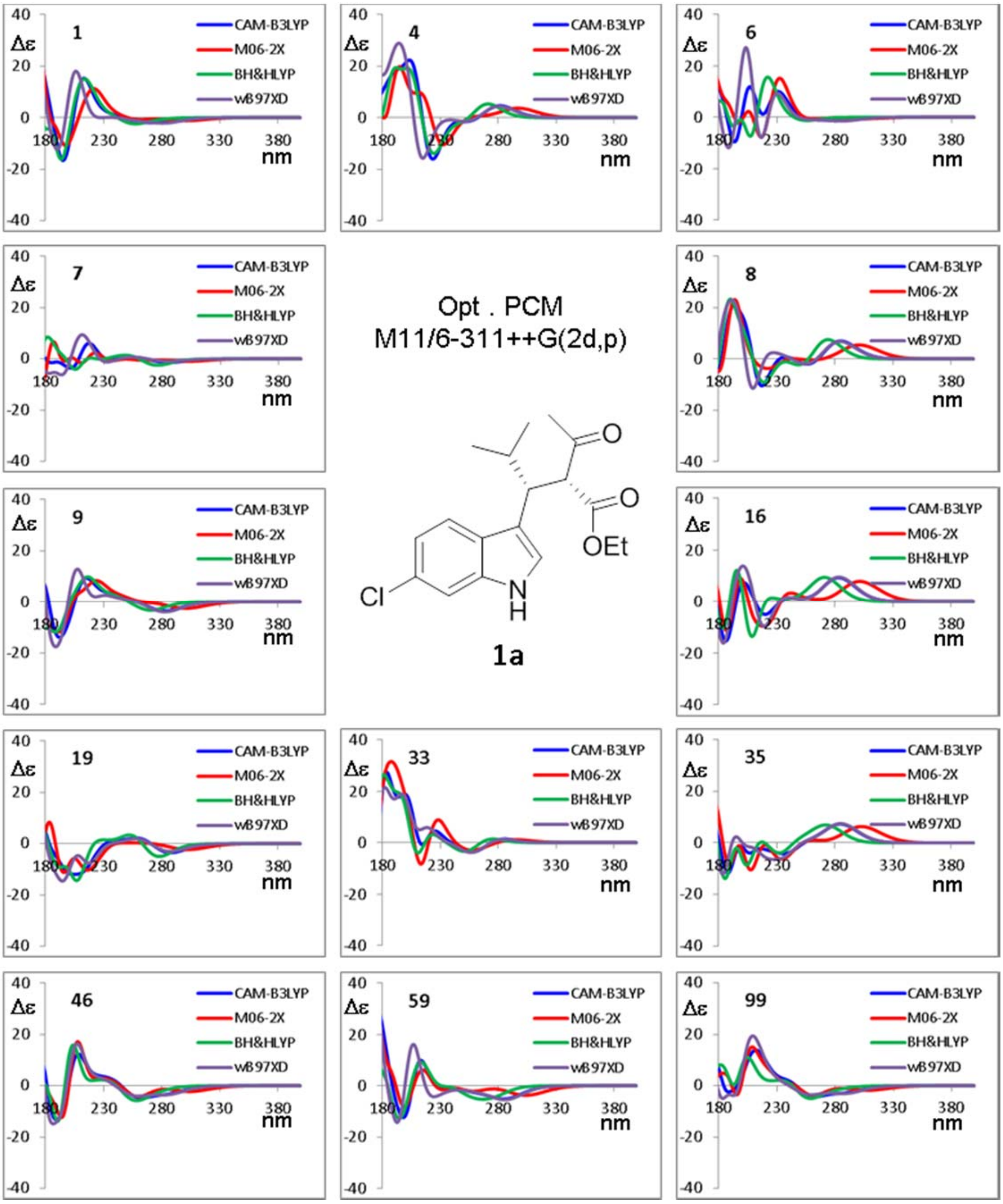

Figure S20. ECD simulation for the conformations of **1a**. The number in each quadrant indicates the conformation label; the four colored lines are the ECD spectra calculated with four different functionals using the geometries obtained by PCM-M11/6-311++G(2d,p).

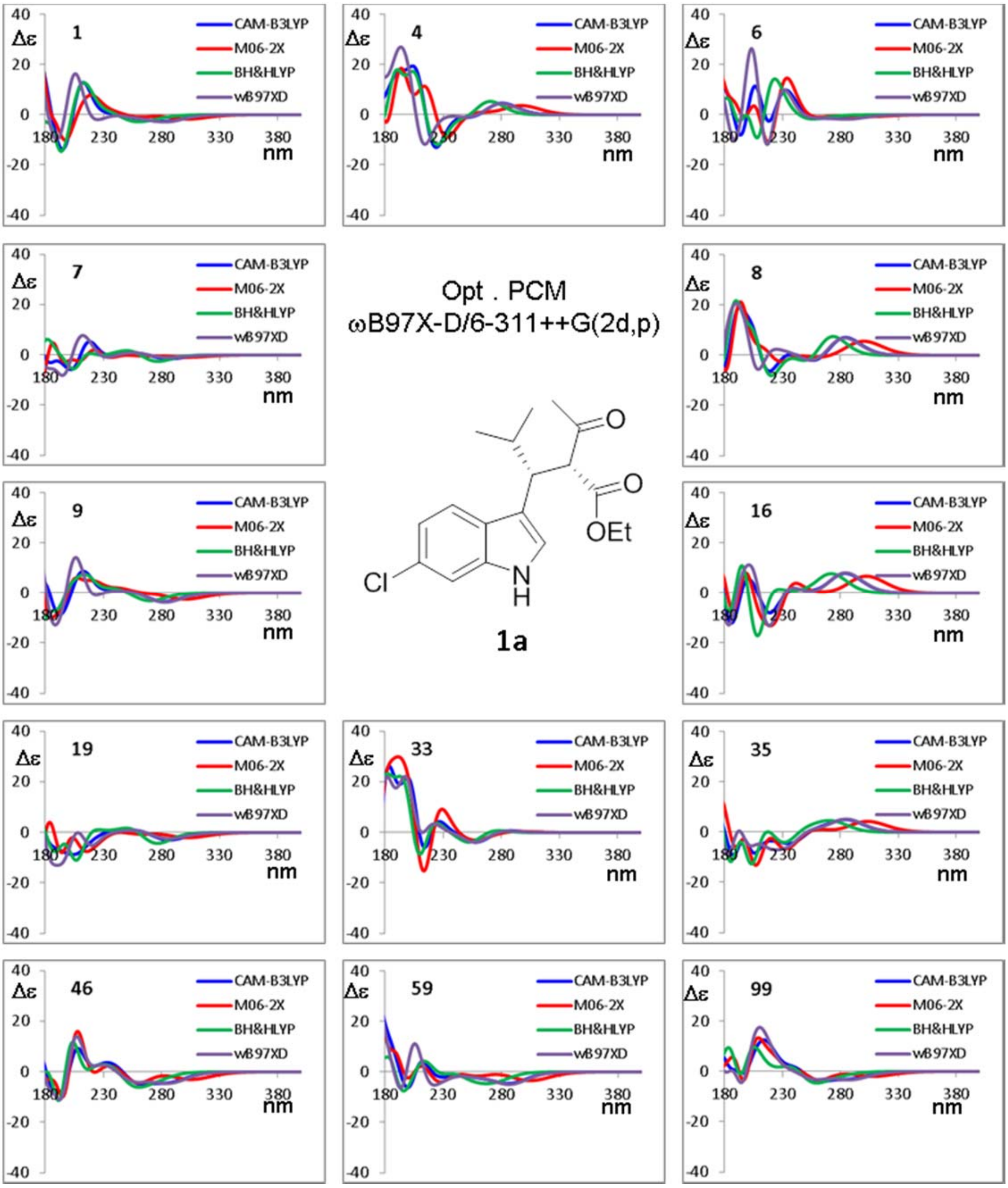

Figure S21. ECD simulation for the conformations of **1a**. The number in each quadrant indicates the conformation label; the four colored lines are the ECD spectra calculated with four different functionals using the geometries obtained by PCM-ωB97X-D/6-311++G(2d,p).

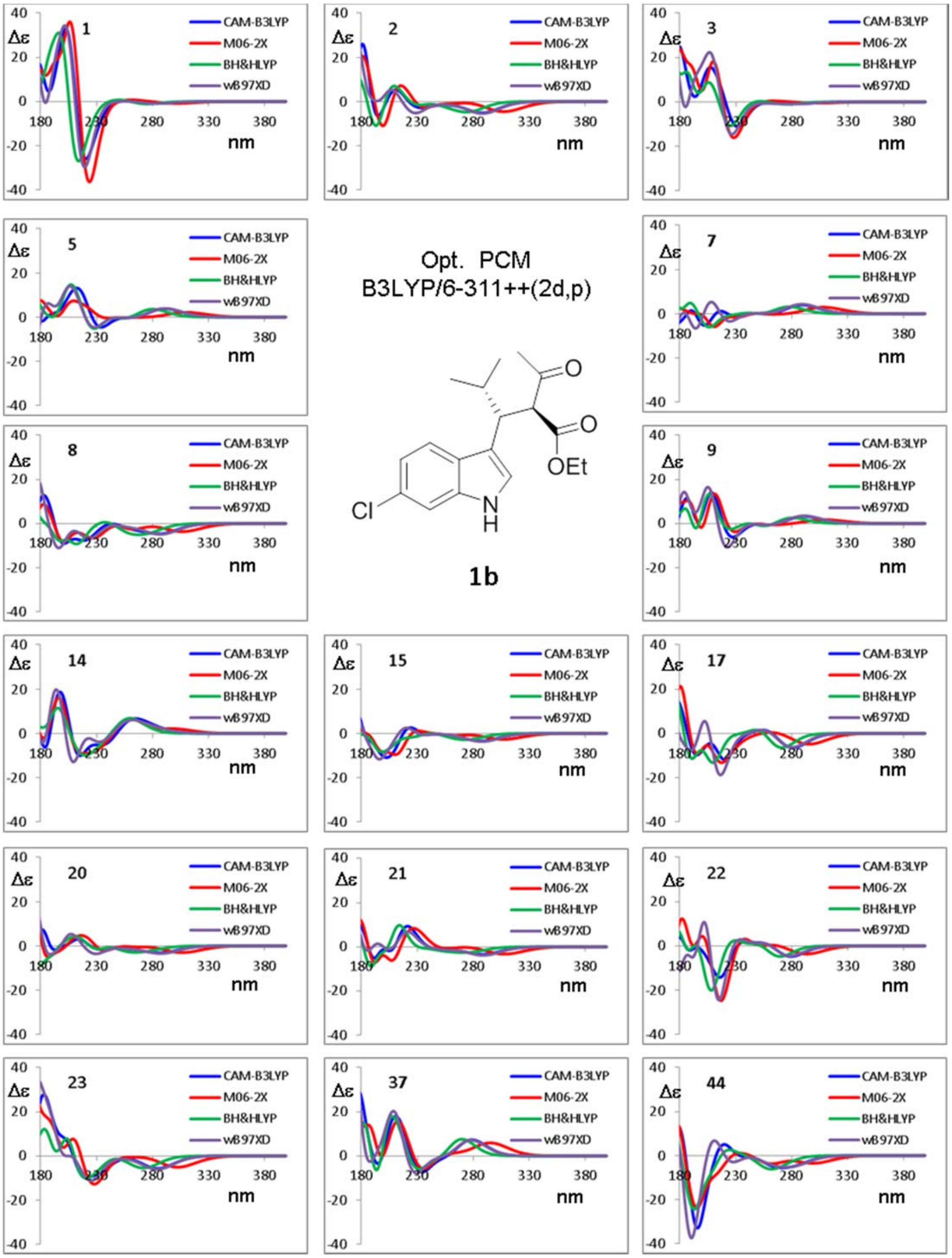

Figure S22. ECD simulation for the conformations of **1b**. The number in each quadrant indicates the conformation label; the four colored lines are the ECD spectra calculated with four different functionals using the geometries obtained by PCM-B3LYP/6-311++G(2d,p).

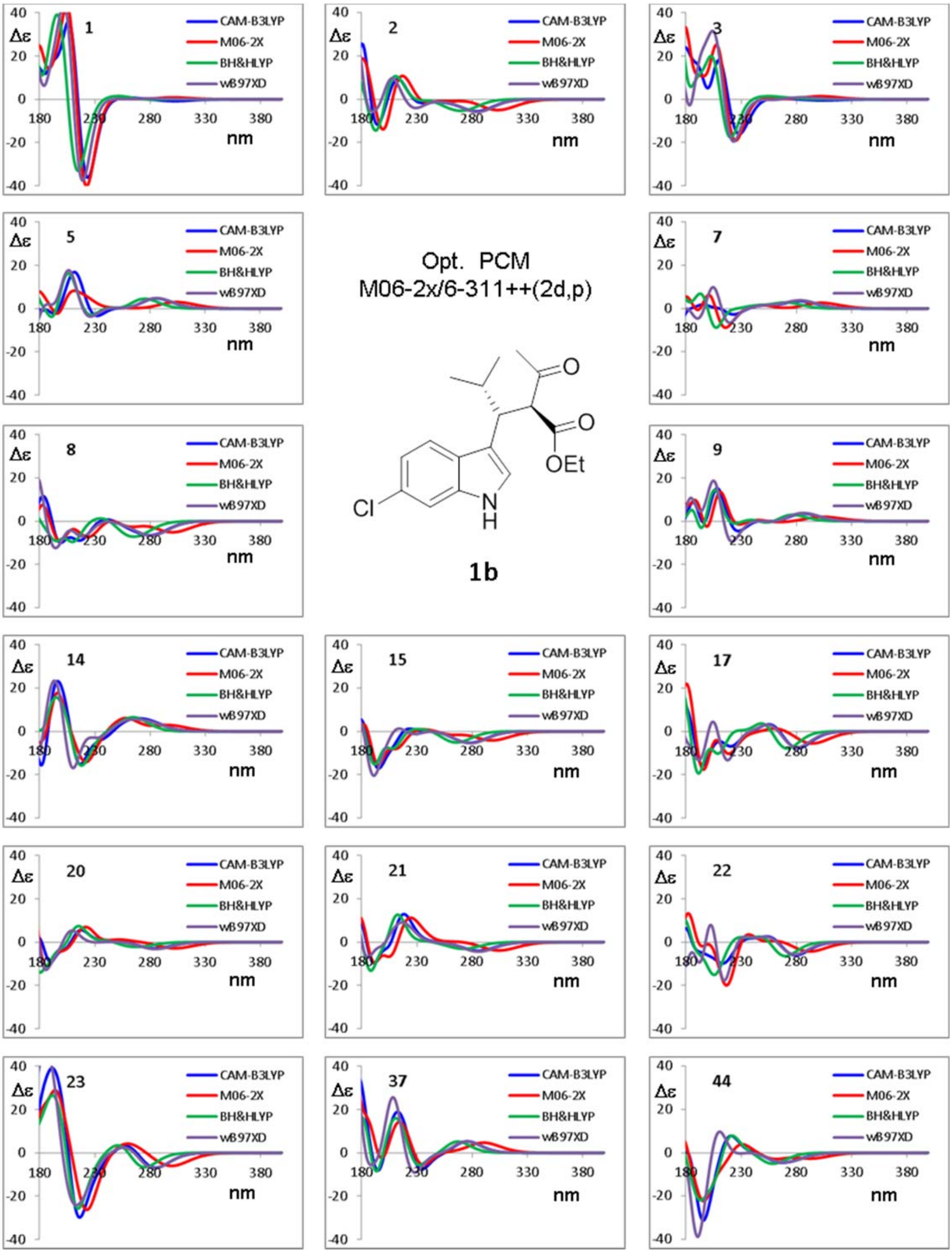

Figure S23. ECD simulation for the conformations of **1b**. The number in each quadrant indicates the conformation label; the four colored lines are the ECD spectra calculated with four different functionals using the geometries obtained by PCM-M06-2x/6-311++G(2d,p).

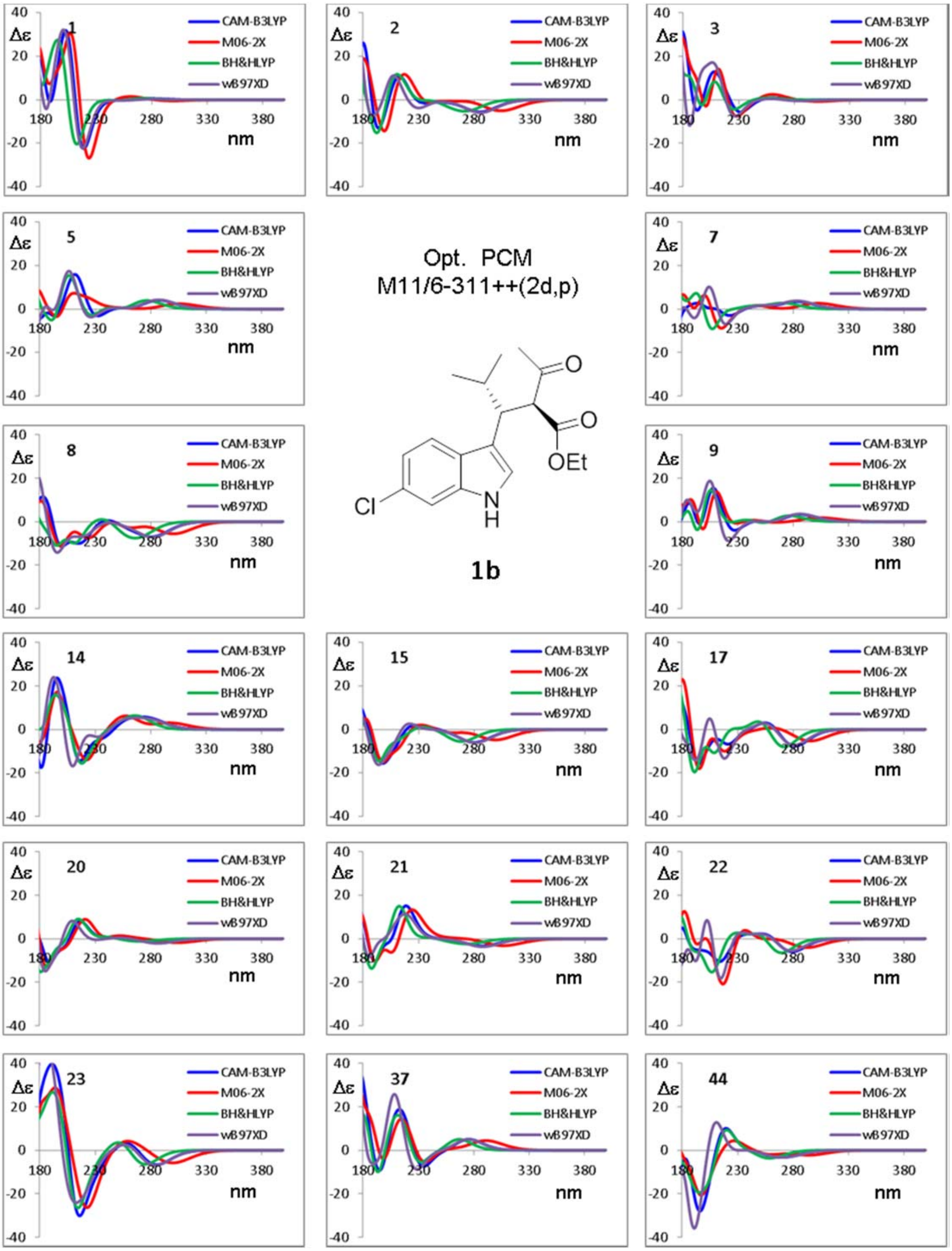

Figure S24. ECD simulation for the conformations of **1b**. The number in each quadrant indicates the conformation label; the four colored lines are the ECD spectra calculated by four different functionals using the geometries obtained by PCM-M11/6-311++G(2d,p).

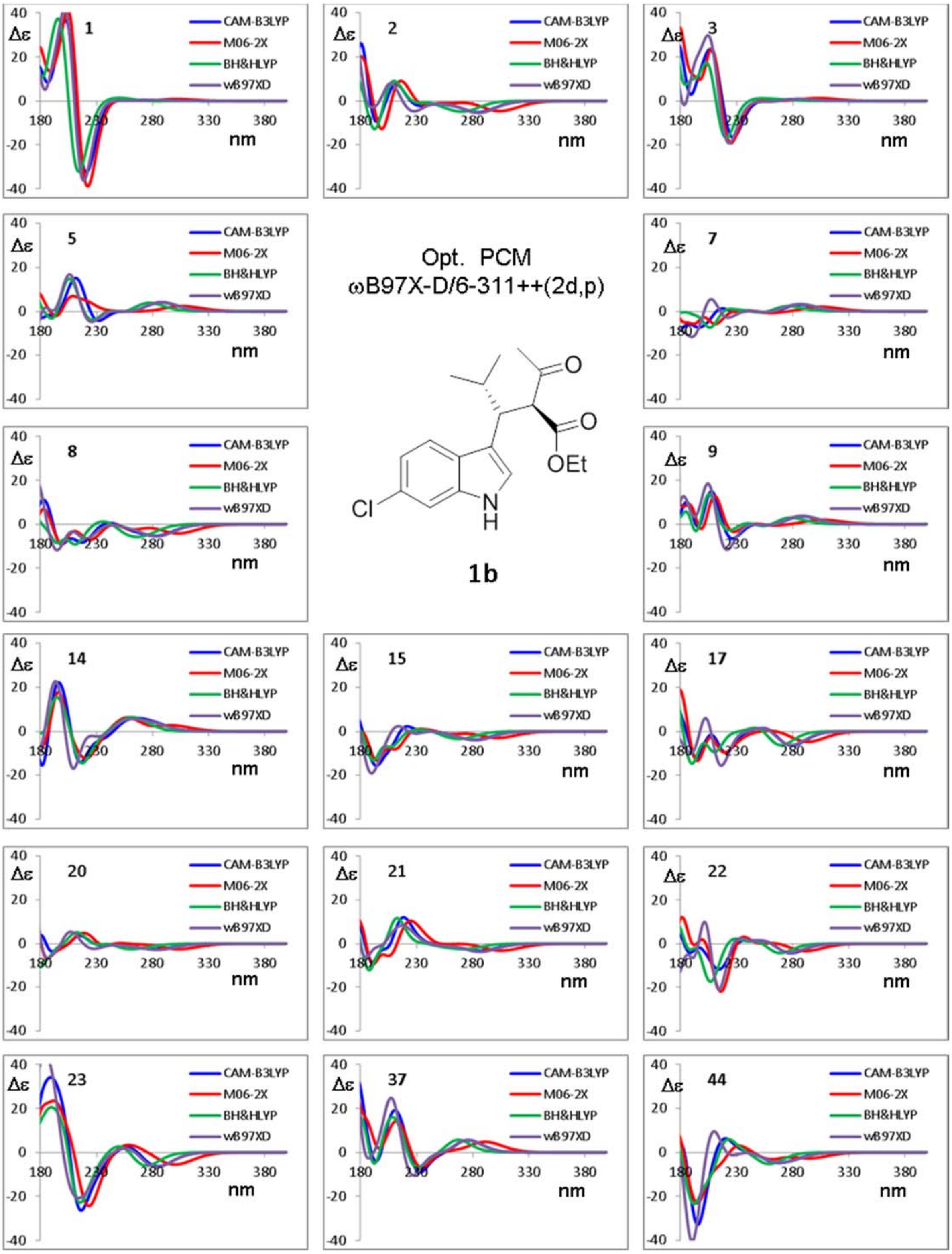

Figure S25. ECD simulation for the conformations of **1b**. The number in each quadrant indicates the conformation label; the four colored lines are the ECD spectra calculated by four different functionals using the geometries obtained by PCM-ωB97X-D/6-311++G(2d,p).

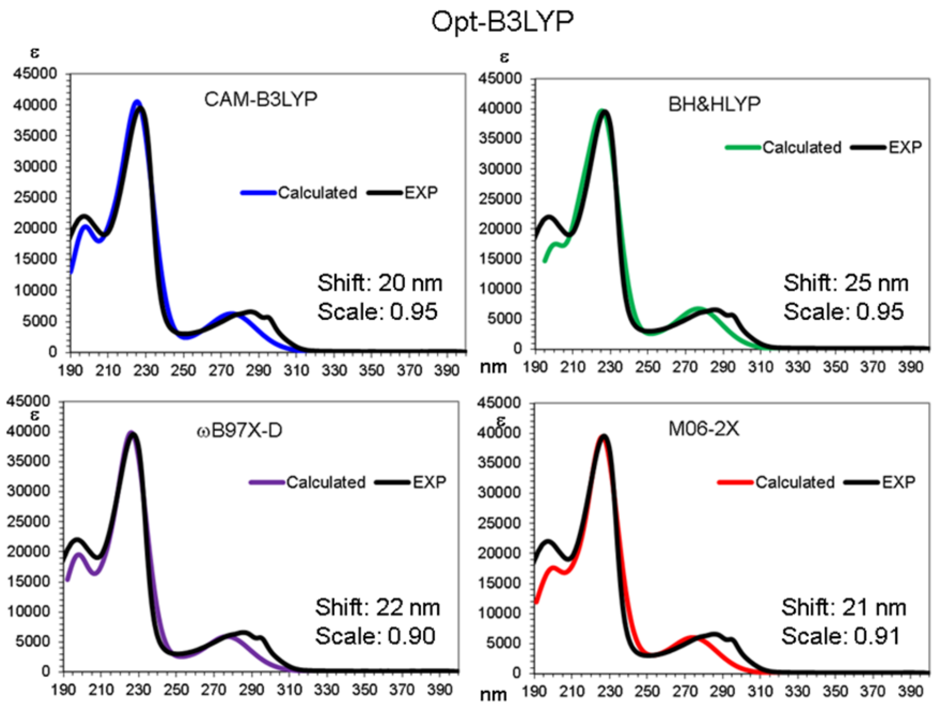

1a

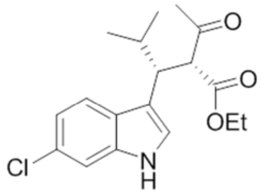

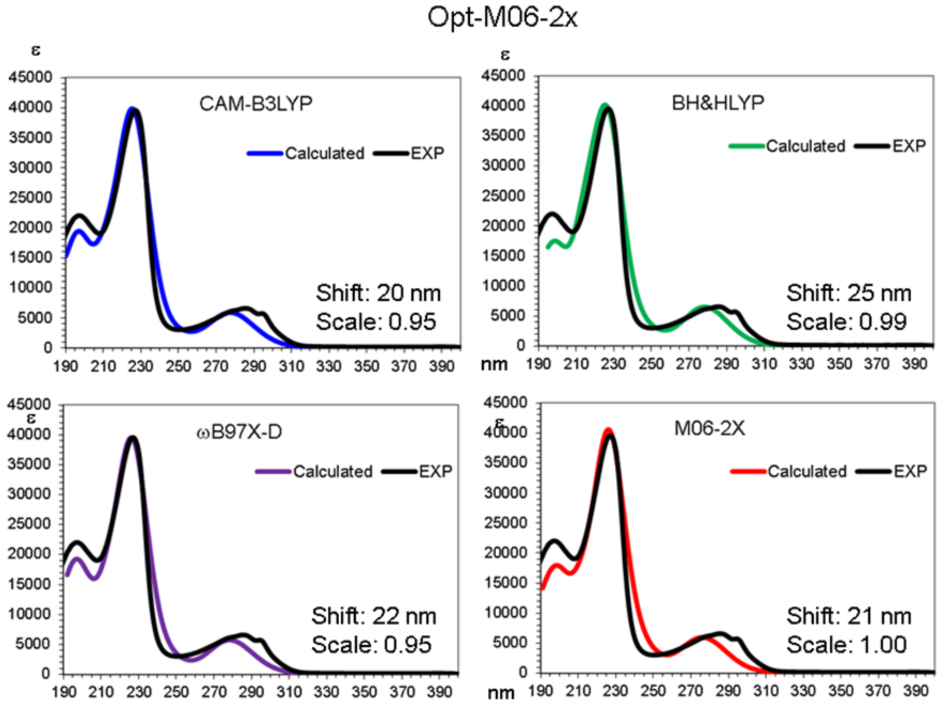

Figure S26. UV simulations for **1a** using the geometries obtained by the reported functional and the 6-311++G(2d,p) basis set. The simulated spectrum was obtained by using the conformational ratio derived from the energies of Table 4.

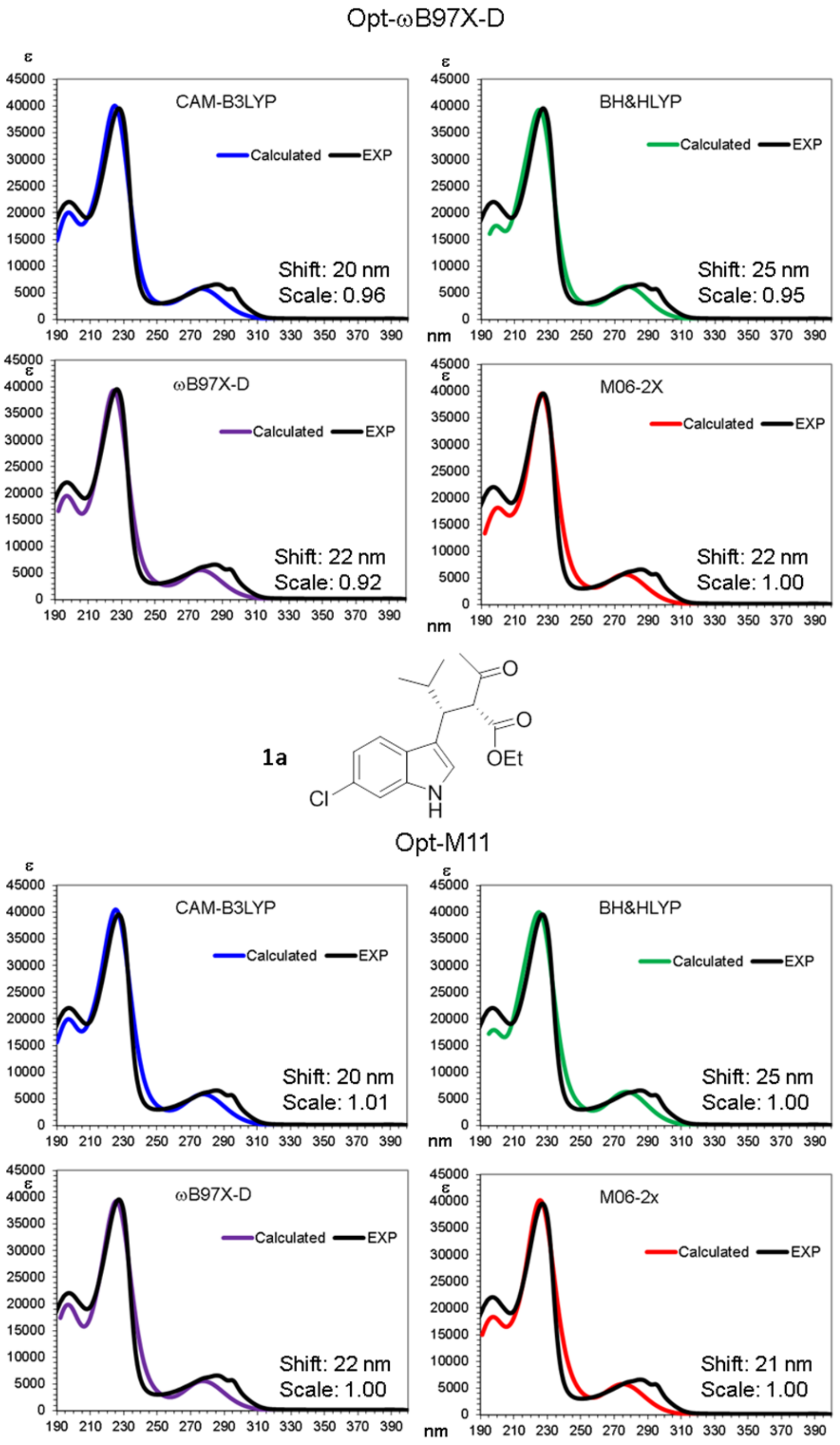

Figure S27. UV simulations for **1a** using the geometries obtained by the reported functional and the 6-311++G(2d,p) basis set. The simulated spectrum was obtained by using the conformational ratio derived from the energies of Table 4.

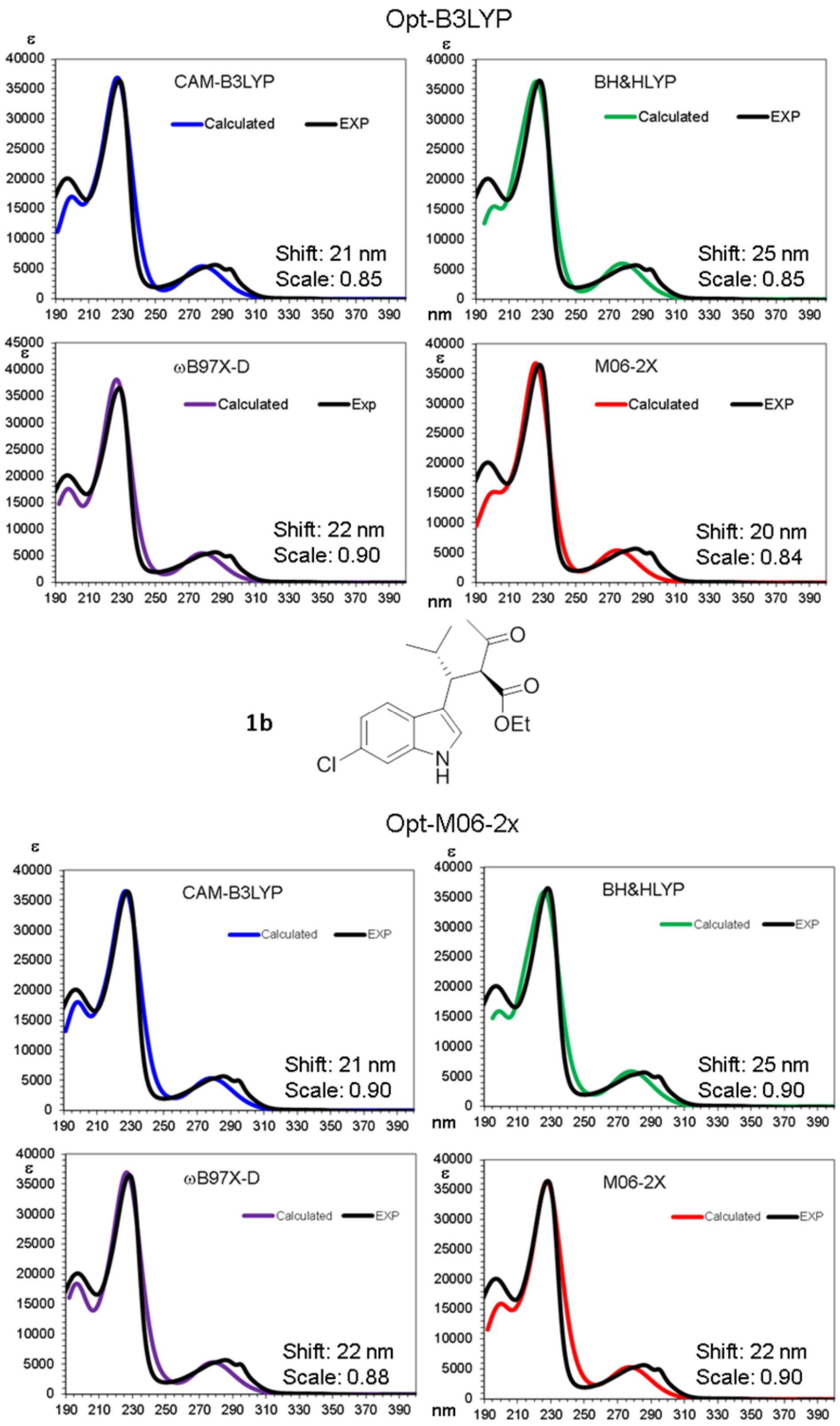

Figure S28. UV simulations for **1b** using the geometries obtained by the reported functional and the 6-311++G(2d,p) basis set. The simulated spectrum was obtained by using the conformational ratio derived from the energies of Table 4.

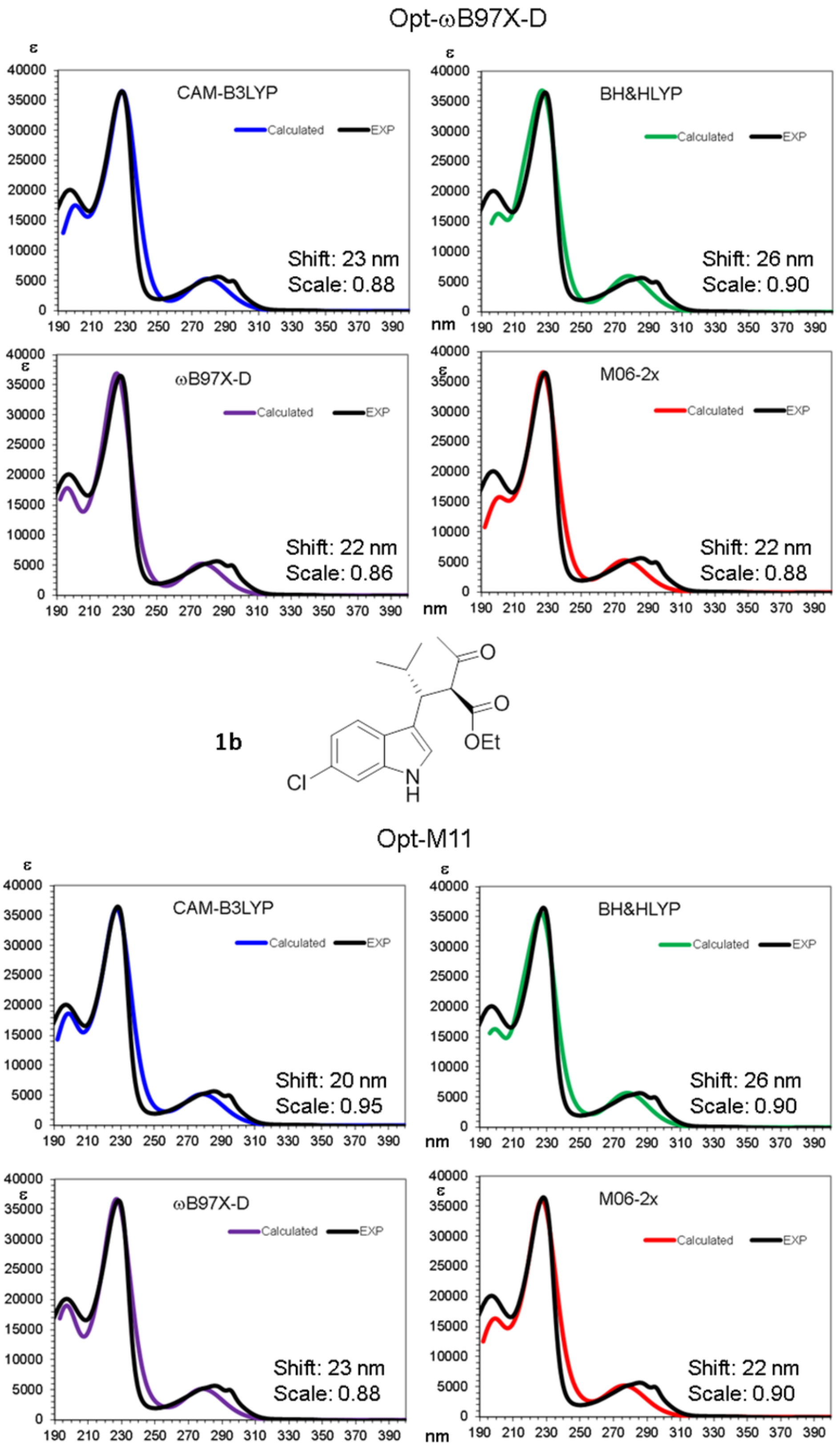

Figure S29. UV simulations for **1b** using the geometries obtained by the reported functional and the 6-311++G(2d,p) basis set. The simulated spectrum was obtained by using the conformational ratio derived from the energies of Table 4.

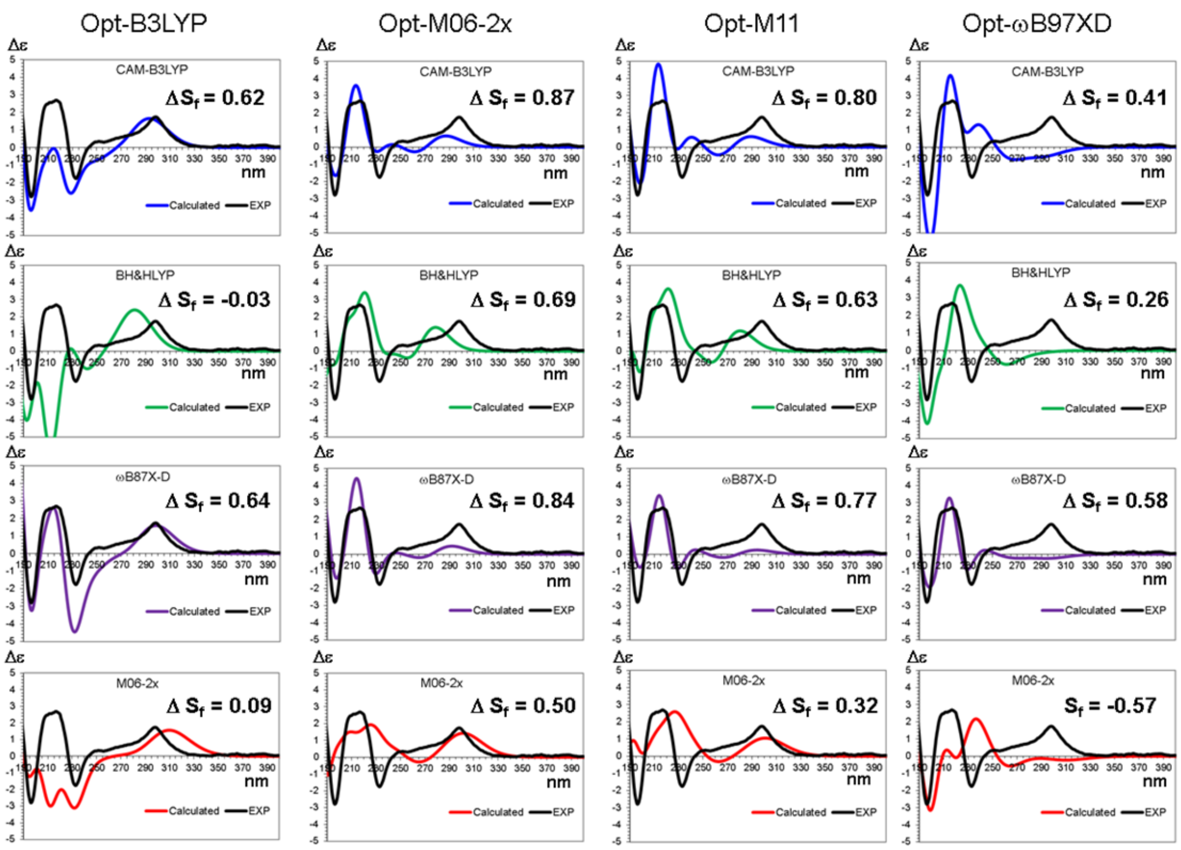

Geometry Optimization Functional	TD-DFT Functional	Similarity Factor	Similarity factor Enant.	ΔS_f	Red shift (nm)	Scaling factor
B3LYP	CAM-B3LYP	0.654	0.039	0.62	6	0.65
B3LYP	BH&HLYP	0.438	0.466	-0.03	6	0.95
B3LYP	ωB97X-D	0.798	0.154	0.64	11	0.62
B3LYP	M06-2x	0.449	0.354	0.09	4	0.65
M06-2x	CAM-B3LYP	0.893	0.018	0.87	5	0.45
M06-2x	BH&HLYP	0.772	0.085	0.69	6	0.78
M06-2x	ωB97X-D	0.866	0.022	0.84	11	0.35
M06-2x	M06-2x	0.699	0.200	0.50	4	0.95
M11	CAM-B3LYP	0.848	0.048	0.80	5	0.54
M11	BH&HLYP	0.749	0.124	0.63	6	0.74
M11	ωB97X-D	0.813	0.043	0.77	11	0.25
M11	M06-2x	0.601	0.285	0.32	2	0.90
ωB97X-D	CAM-B3LYP	0.701	0.290	0.41	2	0.73
ωB97X-D	BH&HLYP	0.495	0.239	0.26	4	0.65
ωB97X-D	ωB97X-D	0.753	0.172	0.58	11	0.26
ωB97X-D	M06-2x	0.061	0.634	-0.57	3	0.72

Figure S30. TD-DFT simulations (colored traces) of the experimental ECD spectrum (black trace) of **1a.** Simulated spectra were red-shifted and scaled to have the best match with the experimental spectrum, as from the table. Similarity factor were obtained with SpecDis software.

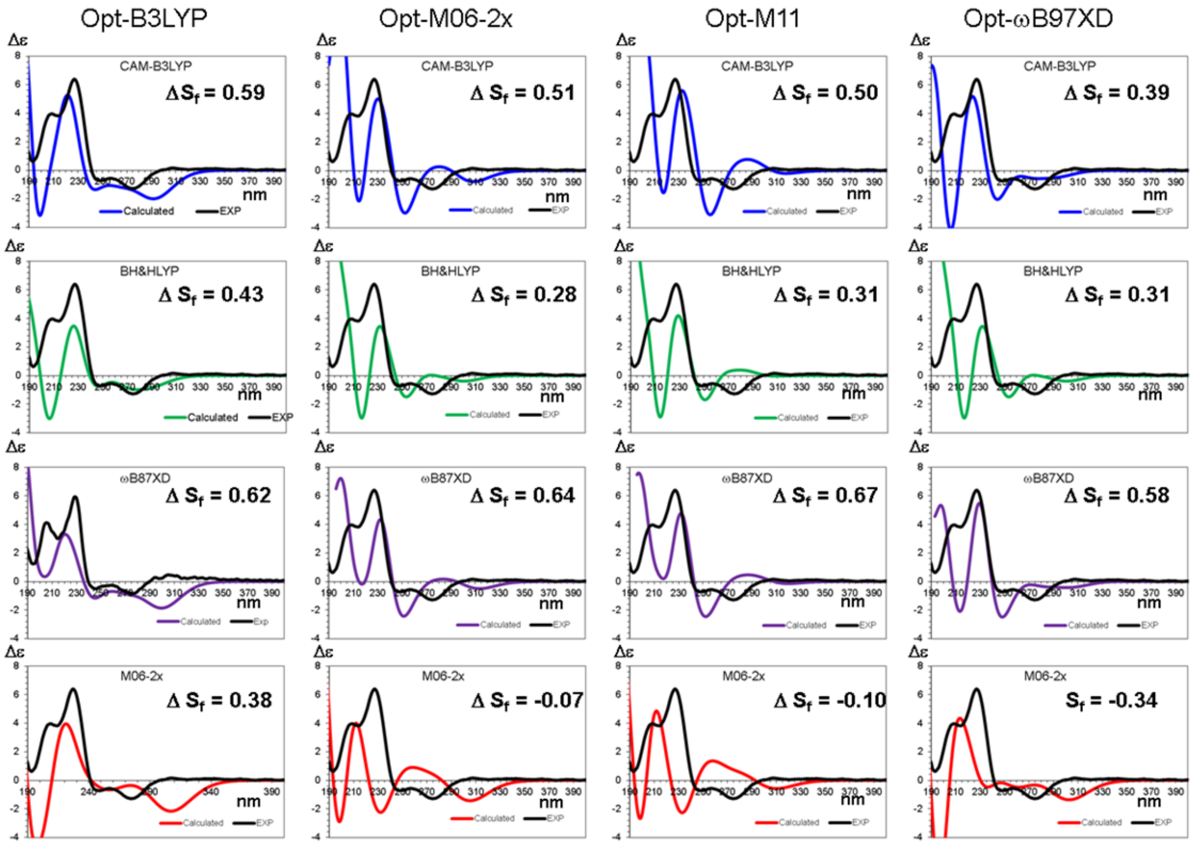

Geometry Optimization Functional	TD-DFT Functional	Similarity Factor	Similarity factor Enant.	ΔS_f	Red shift (nm)	Scaling factor
B3LYP	CAM-B3LYP	0.704	0.110	0.59	5	0.95
B3LYP	BH&HLYP	0.669	0.239	0.43	15	0.51
B3LYP	ωB97X-D	0.630	0.012	0.62	12	0.81
B3LYP	M06-2x	0.521	0.146	0.38	0	1.02
M06-2x	CAM-B3LYP	0.605	0.092	0.51	20	0.98
M06-2x	BH&HLYP	0.484	0.201	0.28	25	0.75
M06-2x	ωB97X-D	0.654	0.010	0.64	26	0.55
M06-2x	M06-2x	0.305	0.372	-0.07	0	1.5
M11	CAM-B3LYP	0.573	0.075	0.50	23	0.95
M11	BH&HLYP	0.506	0.198	0.31	22	0.75
M11	ωB97X-D	0.691	0.023	0.67	26	0.55
M11	M06-2x	0.363	0.464	-0.10	0	1.41
ωB97X-D	CAM-B3LYP	0.610	0.219	0.39	12	0.85
ωB97X-D	BH&HLYP	0.578	0.273	0.31	15	0.75
ωB97X-D	ωB97X-D	0.687	0.104	0.58	23	0.65
ωB97X-D	M06-2x	0.478	0.136	0.34	0	1.6

Figure S31. TD-DFT simulations (colored traces) of the experimental ECD spectrum (black trace) of **1b.** Simulated spectra were red-shifted and scaled to have the best match with the experimental spectrum, as from the table. Similarity factor were obtained with SpecDis software.

Crystal data for compound **1a**

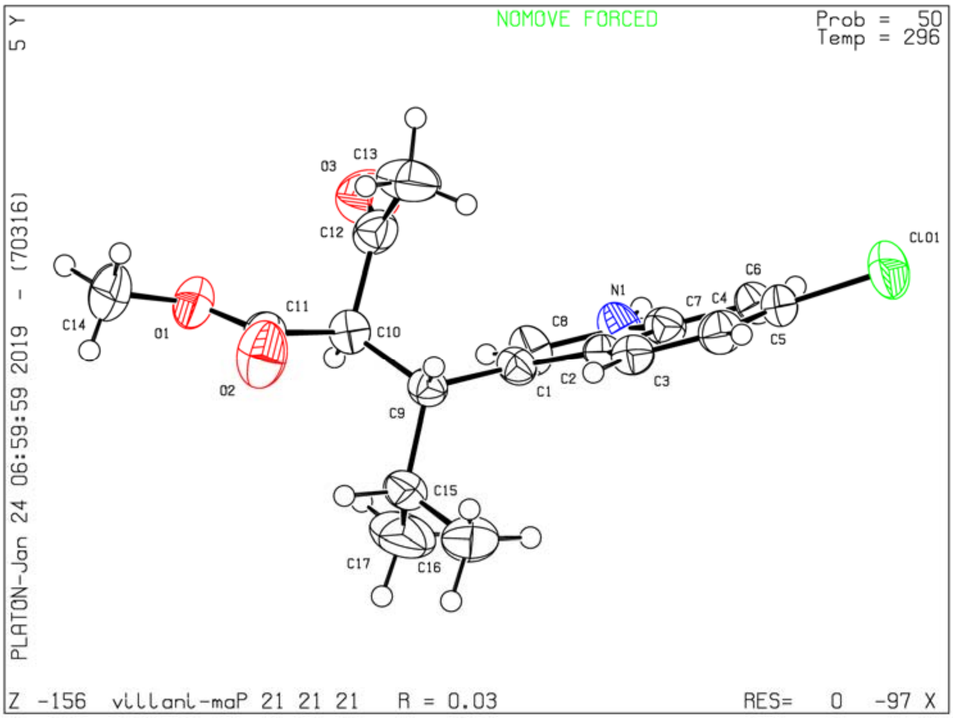

A specimen of $C_{17}H_{20}ClNO_3$, approximate dimensions 0.250 mm x 0.400 mm x 0.400 mm, was used for the X-ray crystallographic analysis. The X-ray intensity data were measured. The total exposure time was 16.55 hours. The frames were integrated with the Bruker SAINT software package using a narrow-frame algorithm. The integration of the data using an orthorhombic unit cell yielded a total of 5581 reflections to a maximum θ angle of 26.00° (0.81 Å resolution), of which 3184 were independent (average redundancy 1.753, completeness = 97.9%, R_{int} = 1.70%, R_{sig} = 2.79%) and 2977 (93.50%) were greater than $2\sigma(F^2)$. The final cell constants of a = 10.0517(8) Å, b = 11.1214(9) Å, c = 15.3258(14) Å, volume = 1713.3(2) $Å^3$, are based upon the refinement of the XYZ-centroids of 9921 reflections above 20 σ(I) with 4.530° < 2θ < 70.17°. Data were corrected for absorption effects using the Multi-Scan method (SADABS). The ratio of minimum to maximum apparent transmission was 0.903. The calculated minimum and maximum transmission coefficients (based on crystal size) are 0.9120 and 0.9440. The structure was solved and refined using the Bruker SHELXTL Software Package, using the space group P 21 21 21, with Z = 4 for the formula unit, $C_{17}H_{20}ClNO_3$. The final anisotropic full-matrix least-squares refinement on F^2 with 208 variables converged at R1 = 3.32%, for the observed data and wR2 = 8.39% for all data. The goodness-of-fit was 1.036. The largest peak in the final difference electron density synthesis was 0.167 $e^-/Å^3$ and the largest hole was -0.191 $e^-/Å^3$ with an RMS deviation of 0.027 $e^-/Å^3$. On the basis of the final model, the calculated density was 1.248 g/cm^3 and F(000), 680 e^-. Flack parameter for the 9*R*,10*R* absolute configuration: 0.03(2). CCDC 1897062 contains the full data.

Table 1. Sample and crystal data for mazza1802.

**Identification code**	mazza1802	
**Chemical formula**	$C_{17}H_{20}ClNO_3$	
**Formula weight**	321.79 g/mol	
**Temperature**	296(2) K	
**Wavelength**	0.71073 Å	
**Crystal size**	0.250 x 0.400 x 0.400 mm	
**Crystal system**	orthorhombic	
**Space group**	P 21 21 21	
**Unit cell dimensions**	a = 10.0517(8) Å	α = 90°
	b = 11.1214(9) Å	β = 90°
	c = 15.3258(14) Å	γ = 90°
**Volume**	1713.3(2) $Å^3$	
**Z**	4	
**Density (calculated)**	1.248 g/cm^3	
**Absorption coefficient**	0.234 mm^{-1}	
**F(000)**	680	

Table 2. Data collection and structure refinement for mazza1802.

**Theta range for data collection**	2.26 to 26.00°	
**Index ranges**	-10<=h<=12, -13<=k<=13, -18<=l<=8	
**Reflections collected**	5581	
**Independent reflections**	3184 [R(int) = 0.0170]	
**Coverage of independent reflections**	97.9%	
**Absorption correction**	Multi-Scan	
**Max. and min. transmission**	0.9440 and 0.9120	
**Structure solution technique**	direct methods	
**Structure solution program**	XT, VERSION 2014/5	
**Refinement method**	Full-matrix least-squares on F^2	
**Refinement program**	SHELXL-2014/7 (Sheldrick, 2014)	
**Function minimized**	Σ w(F_o^2 - F_c^2)^2	
**Data / restraints / parameters**	3184 / 0 / 208	
**Goodness-of-fit on F^2**	1.036	
**Final R indices**	2977 data; I>2σ(I)	R1 = 0.0332, wR2 = 0.0820
	all data	R1 = 0.0364, wR2 = 0.0839
**Weighting scheme**	$w=1/[\sigma^2(F_o^2)+(0.0397P)^2+0.2876P]$<br>where $P=(F_o^2+2F_c^2)/3$	
**Absolute structure parameter**	0.03(2)	
**Extinction coefficient**	0.0670(90)	
**Largest diff. peak and hole**	0.167 and -0.191 $eÅ^{-3}$	
**R.M.S. deviation from mean**	0.027 $eÅ^{-3}$	

Table 3. Atomic coordinates and equivalent isotropic atomic displacement parameters (Å^2) for mazza1802.

U(eq) is defined as one third of the trace of the orthogonalized U_{ij} tensor.

	**x/a**	**y/b**	**z/c**	**U(eq)**
Cl01	0.82987(7)	0.65765(7)	0.54100(5)	0.0673(3)
O1	0.72731(17)	0.40851(19)	0.26475(13)	0.0586(5)
O2	0.6217(2)	0.57758(19)	0.23033(16)	0.0715(6)
N1	0.1257(2)	0.31808(19)	0.41484(14)	0.0472(5)
O3	0.5419(2)	0.3181(2)	0.43352(14)	0.0777(7)
C3	0.1250(2)	0.63079(19)	0.36867(14)	0.0381(5)
C1	0.2558(2)	0.43484(19)	0.33085(13)	0.0350(5)
C9	0.3682(2)	0.47821(19)	0.27364(13)	0.0349(5)
C7	0.0776(2)	0.4322(2)	0.42395(14)	0.0376(5)
C2	0.1557(2)	0.50763(19)	0.37098(12)	0.0325(4)
C4	0.0231(2)	0.6738(2)	0.41944(15)	0.0448(5)
C6	0.9739(2)	0.4747(2)	0.47586(15)	0.0431(5)
C8	0.2340(2)	0.3208(2)	0.36017(15)	0.0445(5)
C10	0.4997(2)	0.4157(2)	0.29862(14)	0.0386(5)
C5	0.9512(2)	0.5957(2)	0.47261(16)	0.0439(6)
C11	0.6201(2)	0.4781(2)	0.25944(15)	0.0436(5)
C12	0.5217(2)	0.4120(2)	0.39720(16)	0.0476(6)
C15	0.3387(3)	0.4701(3)	0.17431(15)	0.0492(6)
C13	0.5180(4)	0.5285(3)	0.44583(18)	0.0703(9)
C16	0.2322(3)	0.5604(4)	0.14832(19)	0.0768(10)
C17	0.3042(4)	0.3449(3)	0.1448(2)	0.0820(10)
C14	0.8516(3)	0.4608(4)	0.2370(2)	0.0778(10)

Table 4. Bond lengths (Å) for mazza1802.

Cl01-C5	1.750(2)	O1-C11	1.330(3)
O1-C14	1.442(3)	O2-C11	1.193(3)
N1-C7	1.365(3)	N1-C8	1.374(3)
N1-H1N	0.84(3)	O3-C12	1.200(3)
C3-C4	1.372(3)	C3-C2	1.405(3)
C3-H3	0.93	C1-C8	1.363(3)
C1-C2	1.431(3)	C1-C9	1.509(3)
C9-C10	1.542(3)	C9-C15	1.554(3)
C9-H9	0.98	C7-C6	1.394(3)

C7-C2	1.406(3)	C4-C5	1.393(4)
C4-H4	0.93	C6-C5	1.366(4)
C6-H6	0.93	C8-H8	0.93
C10-C11	1.518(3)	C10-C12	1.527(3)
C10-H10	0.98	C12-C13	1.496(4)
C15-C17	1.504(4)	C15-C16	1.521(4)
C15-H15	0.98	C13-H13A	0.96
C13-H13B	0.96	C13-H13C	0.96
C16-H16A	0.96	C16-H16B	0.96
C16-H16C	0.96	C17-H17A	0.96
C17-H17B	0.96	C17-H17C	0.96
C14-H14A	0.96	C14-H14B	0.96
C14-H14C	0.96		

Table 5. Bond angles (°) for mazza1802.

C11-O1-C14	116.7(2)	C7-N1-C8	108.8(2)
C7-N1-H1N	124.4(18)	C8-N1-H1N	125.6(18)
C4-C3-C2	119.3(2)	C4-C3-H3	120.3
C2-C3-H3	120.3	C8-C1-C2	105.74(18)
C8-C1-C9	127.5(2)	C2-C1-C9	126.56(18)
C1-C9-C10	110.69(17)	C1-C9-C15	114.06(18)
C10-C9-C15	112.38(18)	C1-C9-H9	106.4
C10-C9-H9	106.4	C15-C9-H9	106.4
N1-C7-C6	129.6(2)	N1-C7-C2	107.3(2)
C6-C7-C2	123.0(2)	C3-C2-C7	118.3(2)
C3-C2-C1	134.1(2)	C7-C2-C1	107.65(19)
C3-C4-C5	120.1(2)	C3-C4-H4	119.9
C5-C4-H4	119.9	C5-C6-C7	116.0(2)
C5-C6-H6	122.0	C7-C6-H6	122.0
C1-C8-N1	110.4(2)	C1-C8-H8	124.8
N1-C8-H8	124.8	C11-C10-C12	106.74(19)
C11-C10-C9	112.22(18)	C12-C10-C9	112.44(19)
C11-C10-H10	108.4	C12-C10-H10	108.4
C9-C10-H10	108.4	C6-C5-C4	123.3(2)
C6-C5-Cl01	118.85(19)	C4-C5-Cl01	117.81(19)
O2-C11-O1	123.5(2)	O2-C11-C10	125.7(2)
O1-C11-C10	110.8(2)	O3-C12-C13	121.8(2)

O3-C12-C10	120.4(3)	C13-C12-C10	117.8(2)
C17-C15-C16	111.7(3)	C17-C15-C9	113.1(2)
C16-C15-C9	110.6(2)	C17-C15-H15	107.0
C16-C15-H15	107.0	C9-C15-H15	107.0
C12-C13-H13A	109.5	C12-C13-H13B	109.5
H13A-C13-H13B	109.5	C12-C13-H13C	109.5
H13A-C13-H13C	109.5	H13B-C13-H13C	109.5
C15-C16-H16A	109.5	C15-C16-H16B	109.5
H16A-C16-H16B	109.5	C15-C16-H16C	109.5
H16A-C16-H16C	109.5	H16B-C16-H16C	109.5
C15-C17-H17A	109.5	C15-C17-H17B	109.5
H17A-C17-H17B	109.5	C15-C17-H17C	109.5
H17A-C17-H17C	109.5	H17B-C17-H17C	109.5
O1-C14-H14A	109.5	O1-C14-H14B	109.5
H14A-C14-H14B	109.5	O1-C14-H14C	109.5
H14A-C14-H14C	109.5	H14B-C14-H14C	109.5

Table 6. Torsion angles (°) for mazza1802.

C8-C1-C9-C10	-38.7(3)	C2-C1-C9-C10	136.1(2)
C8-C1-C9-C15	89.2(3)	C2-C1-C9-C15	-96.0(3)
C8-N1-C7-C6	-176.5(2)	C8-N1-C7-C2	2.0(3)
C4-C3-C2-C7	1.7(3)	C4-C3-C2-C1	-176.9(2)
N1-C7-C2-C3	179.6(2)	C6-C7-C2-C3	-1.8(3)
N1-C7-C2-C1	-1.5(2)	C6-C7-C2-C1	177.1(2)
C8-C1-C2-C3	179.1(2)	C9-C1-C2-C3	3.4(4)
C8-C1-C2-C7	0.4(2)	C9-C1-C2-C7	-175.33(19)
C2-C3-C4-C5	0.0(3)	N1-C7-C6-C5	178.4(2)
C2-C7-C6-C5	0.1(3)	C2-C1-C8-N1	0.9(3)
C9-C1-C8-N1	176.5(2)	C7-N1-C8-C1	-1.8(3)
C1-C9-C10-C11	-165.52(18)	C15-C9-C10-C11	65.7(2)
C1-C9-C10-C12	-45.2(2)	C15-C9-C10-C12	-174.0(2)
C7-C6-C5-C4	1.8(3)	C7-C6-C5-Cl01	-175.96(17)
C3-C4-C5-C6	-1.9(4)	C3-C4-C5-Cl01	175.92(18)
C14-O1-C11-O2	3.0(4)	C14-O1-C11-C10	-174.6(2)
C12-C10-C11-O2	-106.8(3)	C9-C10-C11-O2	16.8(3)
C12-C10-C11-O1	70.7(2)	C9-C10-C11-O1	-165.69(19)
C11-C10-C12-O3	-111.4(3)	C9-C10-C12-O3	125.1(3)

C11-C10-C12-C13	68.3(3)	C9-C10-C12-C13	-55.2(3)
C1-C9-C15-C17	-57.3(3)	C10-C9-C15-C17	69.7(3)
C1-C9-C15-C16	68.9(3)	C10-C9-C15-C16	-164.0(2)

Table 7. Anisotropic atomic displacement parameters (Å^2) for mazza1802.

The anisotropic atomic displacement factor exponent takes the form: -2π^2[h^2 a^{*2} U_{11} + ... + 2 h k a* b* U_{12}]

	U_{11}	U_{22}	U_{33}	U_{23}	U_{13}	U_{12}
Cl01	0.0552(4)	0.0829(5)	0.0638(4)	-0.0080(4)	0.0184(3)	0.0190(4)
O1	0.0349(9)	0.0762(13)	0.0646(11)	0.0027(10)	0.0026(8)	0.0006(9)
O2	0.0570(11)	0.0573(12)	0.1001(16)	0.0220(11)	0.0146(12)	-0.0111(10)
N1	0.0579(12)	0.0361(10)	0.0477(11)	0.0080(9)	0.0163(10)	-0.0042(10)
O3	0.0917(16)	0.0730(14)	0.0684(13)	0.0329(11)	-0.0105(11)	0.0114(12)
C3	0.0400(11)	0.0360(11)	0.0384(11)	0.0010(9)	-0.0020(10)	-0.0028(9)
C1	0.0379(11)	0.0365(11)	0.0305(10)	0.0011(8)	0.0030(9)	-0.0024(9)
C9	0.0368(11)	0.0353(10)	0.0327(10)	0.0018(8)	0.0014(9)	-0.0024(9)
C7	0.0404(11)	0.0401(12)	0.0322(10)	0.0026(9)	-0.0004(9)	-0.0017(10)
C2	0.0322(10)	0.0380(11)	0.0273(9)	-0.0004(8)	-0.0027(8)	-0.0020(9)
C4	0.0460(12)	0.0417(12)	0.0469(12)	-0.0038(11)	-0.0036(10)	0.0066(11)
C6	0.0398(11)	0.0544(13)	0.0352(11)	0.0038(10)	0.0055(9)	-0.0038(11)
C8	0.0512(13)	0.0346(11)	0.0477(13)	0.0029(10)	0.0120(11)	0.0017(10)
C10	0.0399(12)	0.0335(11)	0.0424(12)	0.0007(9)	0.0027(9)	-0.0010(10)
C5	0.0339(11)	0.0593(15)	0.0386(12)	-0.0071(11)	0.0031(10)	0.0074(10)
C11	0.0398(12)	0.0494(13)	0.0417(12)	-0.0010(11)	0.0002(10)	-0.0045(11)
C12	0.0403(12)	0.0559(14)	0.0466(13)	0.0137(11)	-0.0032(10)	-0.0011(11)
C15	0.0451(13)	0.0712(16)	0.0315(11)	0.0033(11)	0.0028(10)	-0.0067(13)
C13	0.090(2)	0.079(2)	0.0422(14)	-0.0040(13)	-0.0113(15)	-0.0165(18)
C16	0.0707(19)	0.114(3)	0.0458(15)	0.0192(17)	-0.0098(15)	0.010(2)
C17	0.102(3)	0.096(2)	0.0477(15)	-0.0247(17)	-0.0050(16)	-0.019(2)
C14	0.0367(14)	0.118(3)	0.079(2)	-0.011(2)	0.0054(14)	-0.0107(16)

Crystal data for compound **2b**

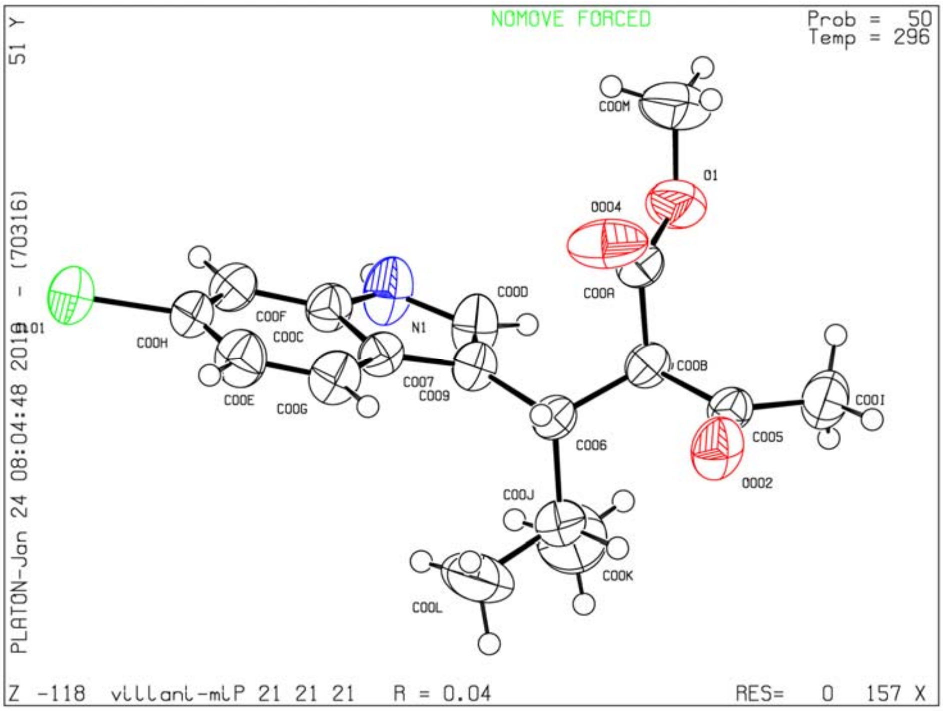

A specimen of $C_{17}H_{20}ClNO_3$, approximate dimensions 0.100 mm x 0.100 mm x 0.300 mm, was used for the X-ray crystallographic analysis. The X-ray intensity data were measured. The total exposure time was 13.33 hours. The frames were integrated with the Bruker SAINT software package using a narrow-frame algorithm. The integration of the data using an orthorhombic unit cell yielded a total of 24496 reflections to a maximum θ angle of 27.25° (0.78 Å resolution), of which 3897 were independent (average redundancy 6.286, completeness = 99.5%, R_{int} = 3.31%, R_{sig} = 2.23%) and 3445 (88.40%) were greater than $2\sigma(F^2)$. The final cell constants of a = 9.9020(12) Å, b = 19.022(2) Å, c = 9.2904(11) Å, volume = 1749.9(4) $Å^3$, are based upon the refinement of the XYZ-centroids of 9886 reflections above 20 σ(I) with 4.384° < 2θ < 53.59°. Data were corrected for absorption effects using the Multi-Scan method (SADABS). The ratio of minimum to maximum apparent transmission was 0.866. The calculated minimum and maximum transmission coefficients (based on crystal size) are 0.9340 and 0.9770. The structure was solved and refined using the Bruker SHELXTL Software Package, using the space group P 21 21 21, with Z = 4 for the formula unit, $C_{17}H_{20}ClNO_3$. The final anisotropic full-matrix least-squares refinement on F^2 with 207 variables converged at R1 = 4.31%, for the observed data and wR2 = 11.45% for all data. The goodness-of-fit was 1.063. The largest peak in the final difference electron density synthesis was 0.162 $e^-/Å^3$ and the largest hole was -0.223 $e^-/Å^3$ with an RMS deviation of 0.035 $e^-/Å^3$. On the basis of the final model, the calculated density was 1.221 g/cm^3 and F(000), 680 e^-. Flack parameter for the C006*S*,C00B*R* absolute configuration: 0.04(2). CCDC 1897063 contains the full data.

Table 1. Sample and crystal data for mazza1801.

**Identification code**	mazza1801	
**Chemical formula**	$C_{17}H_{20}ClNO_3$	
**Formula weight**	321.79 g/mol	
**Temperature**	296(2) K	
**Wavelength**	0.71073 Å	
**Crystal size**	0.100 x 0.100 x 0.300 mm	
**Crystal system**	orthorhombic	
**Space group**	P 21 21 21	
**Unit cell dimensions**	a = 9.9020(12) Å	α = 90°
	b = 19.022(2) Å	β = 90°
	c = 9.2904(11) Å	γ = 90°
**Volume**	1749.9(4) $Å^3$	
**Z**	4	
**Density (calculated)**	1.221 g/cm^3	
**Absorption coefficient**	0.229 mm^{-1}	
**F(000)**	680	

Table 2. Data collection and structure refinement for mazza1801.

**Theta range for data collection**	2.32 to 27.25°	
**Index ranges**	-12<=h<=12, -24<=k<=24, -11<=l<=11	
**Reflections collected**	24496	
**Independent reflections**	3897 [R(int) = 0.0331]	
**Coverage of independent reflections**	99.5%	
**Absorption correction**	Multi-Scan	
**Max. and min. transmission**	0.9770 and 0.9340	
**Structure solution technique**	direct methods	
**Structure solution program**	XT, VERSION 2014/5	
**Refinement method**	Full-matrix least-squares on F^2	
**Refinement program**	SHELXL-2014/7 (Sheldrick, 2014)	
**Function minimized**	$\Sigma w(F_o^2 - F_c^2)^2$	
**Data / restraints / parameters**	3897 / 0 / 207	
**Goodness-of-fit on F^2**	1.063	
**Final R indices**	3445 data; I>2σ(I)	R1 = 0.0431, wR2 = 0.1099

	all data	R1 = 0.0507, wR2 = 0.1145
**Weighting scheme**	$w=1/[\sigma^2(F_o^2)+(0.0622P)^2+0.2438P]$ where $P=(F_o^2+2F_c^2)/3$	
**Absolute structure parameter**	0.040(17)	
**Largest diff. peak and hole**	0.162 and -0.223 eÅ^{-3}	
**R.M.S. deviation from mean**	0.035 eÅ^{-3}	

Table 3. Atomic coordinates and equivalent isotropic atomic displacement parameters (Å^2) for mazza1801.

U(eq) is defined as one third of the trace of the orthogonalized U_{ij} tensor.

	**x/a**	**y/b**	**z/c**	**U(eq)**
Cl01	0.64753(10)	0.50764(5)	0.79235(10)	0.0819(3)
O002	0.3535(2)	0.73230(11)	0.93555(19)	0.0665(6)
O1	0.3935(3)	0.86798(11)	0.2801(3)	0.0822(7)
O004	0.5416(2)	0.78861(13)	0.2065(3)	0.0852(8)
C005	0.2968(3)	0.77291(13)	0.0139(2)	0.0441(5)
C006	0.3219(2)	0.68814(13)	0.2251(2)	0.0412(5)
C007	0.4294(2)	0.63781(12)	0.4611(2)	0.0406(5)
N1	0.3166(3)	0.70093(15)	0.6225(2)	0.0646(7)
C009	0.3412(2)	0.68442(13)	0.3864(2)	0.0427(5)
C00A	0.4293(3)	0.80704(14)	0.2226(3)	0.0504(6)
C00B	0.3062(2)	0.76489(13)	0.1776(2)	0.0409(5)
C00C	0.4101(3)	0.64929(13)	0.6086(3)	0.0469(6)
C00D	0.2752(3)	0.72143(16)	0.4891(3)	0.0593(7)
C00E	0.5897(3)	0.54847(16)	0.5223(3)	0.0598(7)
C00F	0.4769(3)	0.61054(14)	0.7141(3)	0.0542(6)
C00G	0.5233(3)	0.58707(14)	0.4202(3)	0.0529(6)
C00H	0.5649(3)	0.56067(14)	0.6669(3)	0.0538(6)
C00I	0.2168(4)	0.83303(16)	0.9590(3)	0.0658(8)
C00J	0.2044(3)	0.64118(16)	0.1707(4)	0.0673(8)
C00K	0.0712(4)	0.6578(3)	0.2399(7)	0.1142(18)
C00L	0.2412(5)	0.56401(19)	0.1846(7)	0.1121(17)
C00M	0.5047(6)	0.9132(2)	0.3217(6)	0.1169(19)

Table 4. Bond lengths (Å) for mazza1801.

Cl01-C00H	1.745(3)	O002-C005	1.201(3)
O1-C00A	1.325(3)	O1-C00M	1.449(5)
O004-C00A	1.175(4)	C005-C00I	1.481(4)
C005-C00B	1.532(3)	C006-C009	1.513(3)
C006-C00B	1.533(3)	C006-C00J	1.551(4)
C006-H006	0.98	C007-C00G	1.393(3)
C007-C00C	1.401(3)	C007-C009	1.425(3)
N1-C00C	1.356(4)	N1-C00D	1.362(4)
N1-H1N	0.83(4)	C009-C00D	1.354(4)
C00A-C00B	1.518(4)	C00B-H00B	0.98
C00C-C00F	1.394(4)	C00D-H00D	0.93
C00E-C00G	1.368(4)	C00E-C00H	1.385(4)
C00E-H00E	0.93	C00F-C00H	1.360(4)
C00F-H00F	0.93	C00G-H00G	0.93
C00I-H00A	0.96	C00I-H00C	0.96
C00I-H00H	0.96	C00J-C00K	1.501(6)
C00J-C00L	1.518(5)	C00J-H00J	0.98
C00K-H00I	0.96	C00K-H00K	0.96
C00K-H00L	0.96	C00L-H00M	0.96
C00L-H00N	0.96	C00L-H00O	0.96
C00M-H00P	0.96	C00M-H00Q	0.96
C00M-H00R	0.96		

Table 5. Bond angles (°) for mazza1801.

C00A-O1-C00M	115.1(3)	O002-C005-C00I	122.6(2)
O002-C005-C00B	120.6(2)	C00I-C005-C00B	116.8(2)
C009-C006-C00B	110.02(19)	C009-C006-C00J	113.0(2)
C00B-C006-C00J	112.3(2)	C009-C006-H006	107.1
C00B-C006-H006	107.1	C00J-C006-H006	107.1
C00G-C007-C00C	117.8(2)	C00G-C007-C009	135.0(2)
C00C-C007-C009	107.2(2)	C00C-N1-C00D	109.1(2)
C00C-N1-H1N	125.(3)	C00D-N1-H1N	124.(3)
C00D-C009-C007	106.1(2)	C00D-C009-C006	127.8(2)
C007-C009-C006	126.1(2)	O004-C00A-O1	124.4(3)
O004-C00A-C00B	124.6(2)	O1-C00A-C00B	111.0(2)
C00A-C00B-C005	105.6(2)	C00A-C00B-C006	110.0(2)
C005-C00B-C006	112.76(19)	C00A-C00B-H00B	109.4

C005-C00B-H00B	109.4	C006-C00B-H00B	109.4
N1-C00C-C00F	129.8(2)	N1-C00C-C007	107.4(2)
C00F-C00C-C007	122.7(2)	C009-C00D-N1	110.3(2)
C009-C00D-H00D	124.9	N1-C00D-H00D	124.9
C00G-C00E-C00H	119.8(3)	C00G-C00E-H00E	120.1
C00H-C00E-H00E	120.1	C00H-C00F-C00C	116.5(2)
C00H-C00F-H00F	121.8	C00C-C00F-H00F	121.8
C00E-C00G-C007	120.2(2)	C00E-C00G-H00G	119.9
C007-C00G-H00G	119.9	C00F-C00H-C00E	122.9(3)
C00F-C00H-Cl01	119.2(2)	C00E-C00H-Cl01	117.9(2)
C005-C00I-H00A	109.5	C005-C00I-H00C	109.5
H00A-C00I-H00C	109.5	C005-C00I-H00H	109.5
H00A-C00I-H00H	109.5	H00C-C00I-H00H	109.5
C00K-C00J-C00L	112.2(4)	C00K-C00J-C006	113.5(3)
C00L-C00J-C006	110.5(3)	C00K-C00J-H00J	106.8
C00L-C00J-H00J	106.8	C006-C00J-H00J	106.8
C00J-C00K-H00I	109.5	C00J-C00K-H00K	109.5
H00I-C00K-H00K	109.5	C00J-C00K-H00L	109.5
H00I-C00K-H00L	109.5	H00K-C00K-H00L	109.5
C00J-C00L-H00M	109.5	C00J-C00L-H00N	109.5
H00M-C00L-H00N	109.5	C00J-C00L-H00O	109.5
H00M-C00L-H00O	109.5	H00N-C00L-H00O	109.5
O1-C00M-H00P	109.5	O1-C00M-H00Q	109.5
H00P-C00M-H00Q	109.5	O1-C00M-H00R	109.5
H00P-C00M-H00R	109.5	H00Q-C00M-H00R	109.5

Table 6. Torsion angles (°) for mazza1801.

C00G-C007-C009-C00D	-179.6(3)	C00C-C007-C009-C00D	0.5(3)
C00G-C007-C009-C006	3.2(4)	C00C-C007-C009-C006	-176.7(2)
C00B-C006-C009-C00D	42.1(4)	C00J-C006-C009-C00D	-84.3(4)
C00B-C006-C009-C007	-141.4(2)	C00J-C006-C009-C007	92.3(3)
C00M-O1-C00A-O004	1.1(5)	C00M-O1-C00A-C00B	-177.8(3)
O004-C00A-C00B-C005	-76.0(4)	O1-C00A-C00B-C005	102.9(3)
O004-C00A-C00B-C006	46.0(4)	O1-C00A-C00B-C006	-135.1(2)
O002-C005-C00B-C00A	89.6(3)	C00I-C005-C00B-C00A	-89.6(3)
O002-C005-C00B-C006	-30.7(3)	C00I-C005-C00B-C006	150.2(2)
C009-C006-C00B-C00A	58.0(3)	C00J-C006-C00B-C00A	-175.2(2)

C009-C006-C00B-C005	175.7(2)	C00J-C006-C00B-C005	-57.5(3)
C00D-N1-C00C-C00F	-177.3(3)	C00D-N1-C00C-C007	0.9(4)
C00G-C007-C00C-N1	179.2(2)	C009-C007-C00C-N1	-0.9(3)
C00G-C007-C00C-C00F	-2.4(4)	C009-C007-C00C-C00F	177.5(2)
C007-C009-C00D-N1	0.1(3)	C006-C009-C00D-N1	177.2(3)
C00C-N1-C00D-C009	-0.6(4)	N1-C00C-C00F-C00H	178.9(3)
C007-C00C-C00F-C00H	0.8(4)	C00H-C00E-C00G-C007	-1.0(4)
C00C-C007-C00G-C00E	2.4(4)	C009-C007-C00G-C00E	-177.4(3)
C00C-C00F-C00H-C00E	0.7(4)	C00C-C00F-C00H-Cl01	-177.5(2)
C00G-C00E-C00H-C00F	-0.6(5)	C00G-C00E-C00H-Cl01	177.6(2)
C009-C006-C00J-C00K	57.1(4)	C00B-C006-C00J-C00K	-68.1(3)
C009-C006-C00J-C00L	-69.9(4)	C00B-C006-C00J-C00L	164.9(3)

Table 7. Anisotropic atomic displacement parameters (Å^2) for mazza1801.

The anisotropic atomic displacement factor exponent takes the form: -$2\pi^2$[$h^2 a^{*2} U_{11}$ + ... + 2 h k $a^* b^* U_{12}$]

	**U_{11}**	**U_{22}**	**U_{33}**	**U_{23}**	**U_{13}**	**U_{12}**
Cl01	0.0921(6)	0.0655(5)	0.0882(6)	0.0175(4)	-0.0408(5)	0.0026(4)
O002	0.0891(14)	0.0739(13)	0.0364(9)	-0.0041(9)	0.0066(10)	0.0161(12)
O1	0.0986(18)	0.0472(10)	0.1008(18)	-0.0184(11)	-0.0356(15)	0.0052(11)
O004	0.0506(12)	0.0947(17)	0.110(2)	-0.0374(15)	-0.0002(13)	-0.0123(11)
C005	0.0494(13)	0.0480(13)	0.0348(10)	-0.0007(9)	-0.0011(10)	-0.0047(11)
C006	0.0435(12)	0.0459(12)	0.0342(10)	-0.0022(9)	0.0001(9)	0.0006(10)
C007	0.0393(11)	0.0467(12)	0.0356(11)	-0.0015(9)	0.0025(9)	-0.0010(9)
N1	0.0820(18)	0.0777(16)	0.0340(10)	-0.0017(10)	0.0119(11)	0.0309(14)
C009	0.0413(11)	0.0498(12)	0.0369(10)	0.0011(9)	0.0046(9)	0.0042(11)
C00A	0.0615(16)	0.0507(13)	0.0389(11)	-0.0026(10)	-0.0058(11)	-0.0040(12)
C00B	0.0419(12)	0.0480(13)	0.0328(10)	-0.0025(9)	0.0020(9)	0.0016(10)
C00C	0.0529(13)	0.0501(13)	0.0376(11)	-0.0021(10)	0.0009(10)	0.0030(11)
C00D	0.0679(17)	0.0671(17)	0.0429(13)	0.0055(12)	0.0101(12)	0.0269(14)
C00E	0.0531(15)	0.0583(15)	0.0679(17)	-0.0006(13)	0.0000(13)	0.0170(13)
C00F	0.0683(16)	0.0558(14)	0.0384(12)	-0.0005(11)	-0.0075(12)	-0.0038(13)
C00G	0.0529(14)	0.0596(15)	0.0463(13)	-0.0062(11)	0.0060(12)	0.0098(12)
C00H	0.0553(15)	0.0489(13)	0.0571(15)	0.0086(11)	-0.0186(12)	-0.0037(12)
C00I	0.0765(18)	0.0673(18)	0.0537(15)	0.0054(13)	-0.0124(14)	0.0111(15)
C00J	0.0717(19)	0.0605(17)	0.0697(18)	0.0106(14)	-0.0256(16)	-0.0188(15)

C00K	0.0492(18)	0.121(3)	0.173(5)	0.036(3)	-0.025(2)	-0.021(2)
C00L	0.108(3)	0.061(2)	0.168(5)	-0.005(3)	-0.044(3)	-0.024(2)
C00M	0.144(4)	0.063(2)	0.143(4)	-0.019(2)	-0.058(4)	-0.020(3)

Compound **1a**, optimization level B3LYP/6-31G(d)

Conformation #1

C	4.25850200	-0.04613800	-0.39328700
C	3.78830300	-0.35468400	0.87506600
C	2.40878100	-0.24245900	1.07498300
C	1.51198400	0.17071300	0.05065500
C	2.04018300	0.47030600	-1.21779400
C	3.40661400	0.36056200	-1.43773200
C	0.17886900	0.15940900	0.61180100
C	0.32200900	-0.25533000	1.91584700
N	1.65394100	-0.48655700	2.20033200
C	-1.10334400	0.51086700	-0.10952200
C	-1.55686200	1.99800200	0.07856700
C	-1.91096100	2.36485500	1.52692400
C	-0.51485500	2.97494300	-0.48552900
C	-2.24552800	-0.48486500	0.25522900
C	-3.46600300	-0.23893900	-0.62318900
C	-1.82145600	-1.97024200	0.12112600
O	-3.45359600	-0.19714200	-1.83728400
O	-1.98112000	-2.72074600	1.06333400
O	-4.58165700	-0.08272500	0.11648700
C	-5.79470300	0.12704800	-0.62986500
C	-1.22292200	-2.42952200	-1.19092600
Cl	5.98774100	-0.16919600	-0.71190600
H	4.46439500	-0.66921200	1.66300700
H	1.39066500	0.78864000	-2.02814300
H	3.82959800	0.58729800	-2.41012200
H	-0.42940200	-0.43378000	2.67177200
H	2.00503200	-0.84304900	3.07560800
H	-0.92480500	0.38939700	-1.18510700
H	-2.46553300	2.11495700	-0.52915200
H	-1.04689100	2.23858900	2.18928400
H	-2.22134200	3.41448200	1.58378800
H	-2.73636100	1.76133600	1.92011300
H	-0.88838700	4.00409300	-0.43174800
H	-0.28874900	2.75879000	-1.53635100
H	0.42390400	2.92974900	0.07722200
H	-2.54866800	-0.36957200	1.29684100
H	-6.58181000	0.22650600	0.11754800
H	-5.71930100	1.03408300	-1.23516400
H	-5.99080600	-0.72446100	-1.28632300
H	-1.19690100	-3.52095000	-1.21173800
H	-1.78783800	-2.03630200	-2.04141400
H	-0.19666100	-2.05061000	-1.27426800

Compound **1a**, optimization level B3LYP/6-31G(d)

Conformation #4

C	-3.91038700	-0.06183400	-0.01613400
C	-3.30714300	-0.33213700	1.20381400
C	-1.92131700	-0.51957700	1.18845800
C	-1.14510900	-0.44188100	-0.00200500
C	-1.80613100	-0.16543500	-1.21166400
C	-3.18208500	0.02339000	-1.21778600
C	0.23269600	-0.68402800	0.36299000
C	0.23507800	-0.89116100	1.72267800
N	-1.05146900	-0.79937600	2.21775700
C	1.40806100	-0.70475100	-0.59282000
C	2.09090300	-2.10247000	-0.70120200
C	1.07877300	-3.15169900	-1.18834900
C	3.31372800	-2.09058700	-1.63260500
C	2.41255400	0.47300600	-0.34903200
C	1.65595300	1.79445100	-0.21273400
C	3.38351400	0.32170000	0.83622600
O	1.66897700	2.51741800	0.76066000
O	3.23812800	-0.52715500	1.69550900
O	0.95864500	2.06417800	-1.33423600
C	0.14306300	3.25028800	-1.28530600
C	4.56527800	1.27228100	0.86268800
Cl	-5.65655000	0.18687200	-0.06570600
H	-3.89010200	-0.39251000	2.11683200
H	-1.25068200	-0.09444000	-2.14260400
H	-3.70716100	0.23600000	-2.14263100
H	1.06942600	-1.08681100	2.37853700
H	-1.30255800	-0.88282000	3.19057400
H	0.99838000	-0.49240300	-1.58856000
H	2.42757200	-2.38939100	0.30048500
H	0.20413600	-3.20474500	-0.53370300
H	0.72618800	-2.91947600	-2.20228900
H	1.54051300	-4.14541900	-1.21828800
H	3.70820700	-3.10571600	-1.75486100
H	4.13346100	-1.47426100	-1.24670600
H	3.05121300	-1.71840500	-2.63248100
H	3.02891000	0.57304400	-1.25276600
H	0.75992200	4.13002400	-1.08617600
H	-0.32517100	3.32087900	-2.26708000
H	-0.61522200	3.15459200	-0.50485200
H	4.20503000	2.30048500	0.97344200
H	5.21632100	1.01949600	1.70181300
H	5.13268100	1.21780600	-0.07476200

Compound **1a**, optimization level B3LYP/6-31G(d)

Conformation #6

C	4.18236100	-0.10013400	-0.48911500
C	3.77472700	-0.26708100	0.82652800
C	2.40664800	-0.12651700	1.08014500
C	1.46057000	0.17765700	0.06189600
C	1.92633600	0.33568500	-1.25562300
C	3.28033500	0.19584300	-1.52874400
C	0.15651300	0.23434600	0.68492900
C	0.36364800	-0.03626300	2.01813000
N	1.70789800	-0.24289100	2.26056900
C	-1.16036700	0.51175200	-0.00733400
C	-1.61030900	2.01049800	0.04996300
C	-1.94411300	2.50748700	1.46390400
C	-0.57904600	2.92992200	-0.62024000
C	-2.26868900	-0.44621700	0.52588600
C	-3.61590000	-0.20073400	-0.14710200
C	-1.88128100	-1.94565900	0.45062900
O	-4.61888700	0.15310100	0.43110500
O	-2.07149100	-2.64962900	1.42274900
O	-3.56677600	-0.41612900	-1.48409500
C	-4.80372600	-0.20035700	-2.18825600
C	-1.27599700	-2.49277900	-0.82626600
Cl	5.89365600	-0.26592600	-0.87649200
H	4.48860900	-0.49777600	1.61011200
H	1.23827400	0.56914300	-2.06316700
H	3.65543700	0.31458300	-2.53922000
H	-0.34811200	-0.12071200	2.82698900
H	2.10236000	-0.49711900	3.15291800
H	-1.02599600	0.28245100	-1.07178600
H	-2.53124900	2.07297000	-0.54781500
H	-1.07622300	2.42339500	2.12832400
H	-2.23392000	3.56391400	1.43121200
H	-2.77904500	1.95617700	1.90844800
H	-0.95575500	3.95842200	-0.66104000
H	-0.36367800	2.61435900	-1.64822900
H	0.36694000	2.94154900	-0.06817700
H	-2.45335900	-0.26072100	1.58508800
H	-4.58666600	-0.42114200	-3.23354000
H	-5.58156300	-0.86582200	-1.80575800
H	-5.13271200	0.83544700	-2.07275300
H	-1.84863300	-2.17799500	-1.70312200
H	-0.25434300	-2.11134400	-0.94321200
H	-1.24083600	-3.58224800	-0.76294100

Compound **1a**, optimization level B3LYP/6-31G(d)

Conformation #7

C	-3.98626200	-0.34431900	-0.45004500
C	-3.73279900	0.21617300	0.79262200
C	-2.40377300	0.54758700	1.07361200
C	-1.33498600	0.34305100	0.15132100
C	-1.65403700	-0.22890300	-1.09655900
C	-2.96704800	-0.57099700	-1.39251500
C	-0.11940700	0.79617900	0.80198400
C	-0.50111900	1.23041100	2.04779400
N	-1.86421900	1.09710700	2.21375700
C	1.32176000	0.78352300	0.33247800
C	1.69314900	1.89844600	-0.69595300
C	1.03958200	1.75841000	-2.07887300
C	1.42337500	3.29221900	-0.11006900
C	1.72314800	-0.64511900	-0.12719200
C	3.12830800	-0.74979500	-0.71750600
C	1.64898500	-1.65530200	1.04872100
O	3.38729600	-1.27251600	-1.78117200
O	1.87020800	-1.31701500	2.19198800
O	4.05995000	-0.22797400	0.10574000
C	5.41782400	-0.31469700	-0.36121800
C	1.29865500	-3.07805700	0.66671800
Cl	-5.64091500	-0.78884600	-0.86453100
H	-4.53209000	0.38290400	1.50710800
H	-0.88739500	-0.39966900	-1.84467100
H	-3.21748200	-1.01082000	-2.35159500
H	0.11443900	1.61311200	2.85006600
H	-2.37509400	1.32489900	3.05237100
H	1.93233000	0.98108400	1.22045600
H	2.77981900	1.81826300	-0.83728900
H	-0.04848100	1.86807600	-2.02228800
H	1.41399600	2.53993900	-2.75061600
H	1.26761400	0.79430200	-2.54783300
H	1.79658300	4.07116400	-0.78511900
H	1.92097200	3.42269600	0.85877800
H	0.35122500	3.46126100	0.03869400
H	1.06614000	-1.00538200	-0.92135600
H	6.02387000	0.13989400	0.42242600
H	5.70316000	-1.35871000	-0.51342900
H	5.53405600	0.22737400	-1.30335300
H	1.88825500	-3.40649900	-0.19750900
H	1.45829200	-3.74707000	1.51471200
H	0.24310200	-3.11900700	0.36682300

Compound **1a**, optimization level B3LYP/6-31G(d)

Conformation #8

C	-3.86177900	-0.09785900	-0.11811700
C	-3.29119600	-0.32867700	1.12539400
C	-1.90792500	-0.53385400	1.14927000
C	-1.10446000	-0.51634200	-0.02534600
C	-1.73168800	-0.27095600	-1.26000000
C	-3.10427500	-0.06379700	-1.30416500
C	0.26077100	-0.76713600	0.38084100
C	0.23049100	-0.91241400	1.74745800
N	-1.06487600	-0.77787700	2.20998500
C	1.45750200	-0.86215300	-0.54374100
C	2.26847300	-2.18114000	-0.36433100
C	1.37921900	-3.39422000	-0.68320400
C	3.53398400	-2.22556900	-1.23585800
C	2.34111000	0.43266400	-0.53174000
C	1.43975400	1.62302900	-0.86194100
C	3.21283000	0.65854500	0.71237600
O	0.96160100	1.81568800	-1.95831400
O	3.11924900	-0.02280400	1.71608800
O	1.19954100	2.40421200	0.20955500
C	0.26485900	3.47973700	-0.00558900
C	4.27147300	1.74317700	0.59946500
Cl	-5.60309900	0.17125000	-0.21733100
H	-3.89580800	-0.34607700	2.02612300
H	-1.15106700	-0.22139700	-2.17629000
H	-3.60306400	0.12914700	-2.24755900
H	1.04615700	-1.08056500	2.43355700
H	-1.34261800	-0.83922800	3.17709900
H	1.06061600	-0.88712400	-1.56777800
H	2.57852100	-2.24642400	0.68419600
H	1.07283200	-3.39017500	-1.73770600
H	1.92283200	-4.32827600	-0.49976300
H	0.47292100	-3.40358300	-0.07101700
H	4.01331300	-3.20795700	-1.15724900
H	4.28042700	-1.48180200	-0.93556800
H	3.29654100	-2.06045500	-2.29560700
H	3.02584700	0.37200300	-1.38681000
H	0.19749400	4.00029200	0.94967800
H	0.62695100	4.15070500	-0.78851900
H	-0.70768500	3.07640000	-0.29666700
H	3.82712600	2.69903400	0.30447000
H	4.78271900	1.85326900	1.55783700
H	5.00475800	1.47111600	-0.17095000

Compound **1a**, optimization level B3LYP/6-31G(d)

Conformation #9

C	-4.37253400	-0.24282400	-0.35647000
C	-3.91566100	0.64536000	0.60710000
C	-2.53203000	0.69839600	0.80257600
C	-1.62014500	-0.10529800	0.06428700
C	-2.13372500	-0.98902400	-0.90057100
C	-3.50492800	-1.05604000	-1.10977400
C	-0.28760100	0.22195900	0.52010800
C	-0.44390400	1.18232500	1.49217100
N	-1.78573500	1.46688400	1.66791300
C	0.98528100	-0.43354300	0.03101000
C	1.16625100	-1.83459000	0.71697100
C	2.14369700	-2.77022200	-0.01447700
C	1.51846200	-1.74608900	2.20907900
C	2.21367700	0.51392300	0.17213200
C	3.42235500	-0.00814000	-0.59551500
C	1.92868800	1.95494600	-0.33508600
O	3.43117500	-0.28408900	-1.77805500
O	2.15174800	2.89613400	0.40161900
O	4.51026800	-0.09316000	0.19719600
C	5.71774800	-0.52809400	-0.45538700
C	1.39240200	2.13265500	-1.73862300
Cl	-6.10685300	-0.35779000	-0.65097500
H	-4.60541700	1.26239700	1.17312000
H	-1.46981800	-1.61910600	-1.48630500
H	-3.91908400	-1.73086000	-1.85066700
H	0.30109500	1.71595500	2.06462000
H	-2.15000900	2.17150700	2.29011300
H	0.86575300	-0.62926300	-1.04285000
H	0.17266100	-2.29771700	0.64597600
H	3.18769900	-2.47995000	0.14510100
H	2.03557300	-3.79173000	0.36889000
H	1.96123300	-2.79254500	-1.09426600
H	1.46148500	-2.73877200	2.67012100
H	0.82936900	-1.08815000	2.74885900
H	2.53937600	-1.37572200	2.36195700
H	2.49811900	0.63028300	1.21909800
H	6.48499300	-0.51921400	0.31878200
H	5.59091200	-1.53473900	-0.86223500
H	5.97941700	0.15492200	-1.26733100
H	1.96276200	1.53387700	-2.45456800
H	0.35252400	1.78631700	-1.77715800
H	1.42427400	3.19148200	-2.00333600

Compound **1a**, optimization level B3LYP/6-31G(d)

Conformation #16

C	4.18792500	-0.25241700	-0.43389400
C	3.80360400	0.05814000	0.86212600
C	2.43848500	0.28255800	1.07029500
C	1.47742600	0.20854800	0.02549500
C	1.91791700	-0.12007400	-1.26784500
C	3.26786500	-0.34740400	-1.49571600
C	0.18385600	0.49689700	0.60110200
C	0.40891700	0.71821900	1.94071900
N	1.75736500	0.59249300	2.22623700
C	-1.13430200	0.53992500	-0.14398000
C	-1.68960100	1.98171900	-0.37201400
C	-2.12957400	2.70578400	0.90943300
C	-0.68850800	2.83732900	-1.16235100
C	-2.15513100	-0.40986700	0.53646600
C	-3.56901500	-0.35174500	-0.04049500
C	-1.69010300	-1.88767000	0.44442800
O	-4.57597200	-0.22335700	0.62407800
O	-1.01179100	-2.28295000	-0.47902500
O	-3.57056700	-0.50536500	-1.37961500
C	-4.86672300	-0.49594500	-2.00409500
C	-2.15365600	-2.79263000	1.56741700
Cl	5.89530000	-0.54533500	-0.76583200
H	4.53112700	0.11896500	1.66459500
H	1.21063900	-0.20824300	-2.08697500
H	3.62577300	-0.60399400	-2.48660900
H	-0.28621500	0.95771200	2.73334800
H	2.17209600	0.71094400	3.13722000
H	-0.95673600	0.11589500	-1.13729500
H	-2.57956200	1.86249000	-1.00504400
H	-1.28176800	2.87235000	1.58379300
H	-2.54672800	3.68873200	0.66141100
H	-2.90366500	2.15290300	1.45370100
H	-1.12863700	3.80937700	-1.41385200
H	-0.39616400	2.34948700	-2.09972500
H	0.22332900	3.02112800	-0.58347600
H	-2.27977900	-0.15881800	1.59331500
H	-4.67734300	-0.63911800	-3.06784400
H	-5.48604800	-1.30542600	-1.60955900
H	-5.37025200	0.45775200	-1.82578900
H	-3.21651200	-2.63491800	1.78511800
H	-1.96559400	-3.83726900	1.31154800
H	-1.59927000	-2.54151900	2.48156000

Compound **1a**, optimization level B3LYP/6-31G(d)

Conformation #19

C	-3.88836300	-0.58654300	-0.43134600
C	-3.71885800	0.25535000	0.65731500
C	-2.42396800	0.72850100	0.89112100
C	-1.30825300	0.39148000	0.06849600
C	-1.54233200	-0.46638900	-1.02485300
C	-2.82004100	-0.95247400	-1.27017900
C	-0.14760000	1.05632600	0.63054300
C	-0.60413900	1.73507200	1.73391400
N	-1.96328300	1.55472200	1.88975500
C	1.30917800	1.03602700	0.20988300
C	1.64776500	1.93835900	-1.02021400
C	1.00377300	1.50471800	-2.34550200
C	1.31641300	3.40816600	-0.72114000
C	1.82513300	-0.42045100	0.06976600
C	3.33042100	-0.49025600	-0.17242300
C	1.57751900	-1.22277100	1.38020200
O	4.14605300	0.33649200	0.16681800
O	1.84818000	-0.73829200	2.45857400
O	3.65569900	-1.65414800	-0.78589600
C	5.06363200	-1.86365600	-0.99945900
C	1.01598800	-2.61790100	1.22013400
Cl	-5.49583100	-1.22131700	-0.77784500
H	-4.55516200	0.52577000	1.29340500
H	-0.73638700	-0.74857200	-1.69387900
H	-3.00574000	-1.61172700	-2.11097300
H	-0.04524200	2.33102100	2.44217500
H	-2.51961600	1.93242800	2.64079000
H	1.87903300	1.45262800	1.04797700
H	2.73530600	1.87506400	-1.14087100
H	-0.08800500	1.58403700	-2.31024800
H	1.35464800	2.14869000	-3.16057800
H	1.26181700	0.47399300	-2.61814200
H	1.65779400	4.05222200	-1.53997300
H	1.80933800	3.74844900	0.19756500
H	0.23784800	3.56035800	-0.60110500
H	1.33606000	-0.94975000	-0.75137000
H	5.14348100	-2.83591500	-1.48618700
H	5.47342600	-1.07788400	-1.63922300
H	5.59963300	-1.86268500	-0.04690100
H	0.01008300	-2.56008700	0.78533700
H	1.63684000	-3.19446400	0.52417000
H	0.96988000	-3.11801500	2.18962200

Compound **1a**, optimization level B3LYP/6-31G(d)

Conformation #33

C	4.19642300	0.09720600	0.44262800
C	3.71244400	0.62644300	-0.74535200
C	2.35725100	0.41303400	-1.01756000
C	1.50168200	-0.30949900	-0.14020800
C	2.04090900	-0.82447900	1.05143100
C	3.38372000	-0.62109300	1.34037900
C	0.18872000	-0.33779700	-0.74079900
C	0.29543000	0.35628800	-1.92237100
N	1.59287700	0.80925000	-2.09211700
C	-1.05491500	-0.95287200	-0.13533800
C	-1.88161300	-1.76051400	-1.18107300
C	-1.05897100	-2.96770400	-1.66486400
C	-3.24785700	-2.24067100	-0.67123700
C	-1.80943100	0.14712200	0.68216400
C	-2.46285700	1.22287200	-0.16895700
C	-2.73520400	-0.43582000	1.78439600
O	-3.05247500	1.05710800	-1.21731300
O	-2.39205000	-1.43783400	2.37835800
O	-2.32180700	2.44083600	0.40617700
C	-2.92750500	3.53508500	-0.30320500
C	-3.99617000	0.31905400	2.15116000
Cl	5.89724400	0.33338900	0.84591500
H	4.36060600	1.17617000	-1.41968200
H	1.41834200	-1.38033400	1.74745500
H	3.81821300	-1.01144200	2.25401900
H	-0.45794200	0.57325700	-2.66651000
H	1.93083600	1.31345100	-2.89684900
H	-0.73573000	-1.66644100	0.63403600
H	-2.07208600	-1.10722500	-2.03948200
H	-0.88252800	-3.67417600	-0.84328700
H	-1.59569600	-3.50624300	-2.45457100
H	-0.08547700	-2.66579700	-2.06297700
H	-3.74727800	-2.82832000	-1.45062300
H	-3.91037300	-1.40245500	-0.43243500
H	-3.15158200	-2.87438000	0.21690300
H	-1.04255000	0.66632600	1.27350300
H	-2.71767100	4.42224000	0.29460100
H	-2.49139000	3.63127000	-1.30074900
H	-4.00561600	3.38152000	-0.40086300
H	-4.50575500	-0.19886800	2.96557200
H	-3.74710100	1.34210200	2.45647700
H	-4.66417800	0.39617000	1.28478200

Compound **1a**, optimization level B3LYP/6-31G(d)

Conformation #35

C	4.22557900	-0.27908500	-0.36920700
C	3.78407900	-0.12121800	0.93619600
C	2.41689200	0.12196700	1.10618800
C	1.50930400	0.21259400	0.01599400
C	2.00607800	0.03500700	-1.28658700
C	3.35908200	-0.20785700	-1.47703800
C	0.19602000	0.47050900	0.56075200
C	0.35796600	0.51471900	1.92706400
N	1.68601600	0.30702500	2.25853200
C	-1.07967600	0.65195800	-0.23689600
C	-1.58105400	2.12991400	-0.30305700
C	-2.03687400	2.71009600	1.04424600
C	-0.52212200	3.03695500	-0.94771700
C	-2.16042400	-0.34532600	0.25183400
C	-3.44573800	-0.28318200	-0.56872400
C	-1.64725300	-1.80842900	0.12127800
O	-3.52871500	0.03211000	-1.73387300
O	-1.04475400	-2.16579300	-0.86784000
O	-4.50525200	-0.68525200	0.17523400
C	-5.76287200	-0.73281800	-0.52378700
C	-1.96436400	-2.73834600	1.27229700
Cl	5.93877400	-0.58619200	-0.65413900
H	4.47095600	-0.18677400	1.77336700
H	1.33990800	0.07700900	-2.14299900
H	3.76024000	-0.34895300	-2.47450200
H	-0.36957700	0.68114600	2.70927900
H	2.05787600	0.30308400	3.19533500
H	-0.86573700	0.35526700	-1.26874200
H	-2.44833200	2.11950500	-0.97318200
H	-1.20423400	2.77749500	1.75389800
H	-2.42907000	3.72405300	0.90376500
H	-2.83461700	2.11707700	1.50828800
H	-0.91791100	4.04992700	-1.08634500
H	-0.21942300	2.65989500	-1.93153000
H	0.37705800	3.10990600	-0.32561400
H	-2.42742100	-0.16940400	1.29773600
H	-6.49150700	-1.07934200	0.20942700
H	-6.03104700	0.26000100	-0.89388900
H	-5.70547900	-1.42384700	-1.36867100
H	-3.03312100	-2.69580700	1.51201200
H	-1.66982300	-3.75961200	1.02269400
H	-1.41797700	-2.40781100	2.16512800

Compound **1a**, optimization level B3LYP/6-31G(d)

Conformation #46

C	-3.91692500	-0.57768700	-0.40799600
C	-3.75061200	0.41880900	0.54079000
C	-2.44211700	0.86059000	0.76207700
C	-1.31415900	0.34095900	0.06427400
C	-1.54340000	-0.67475000	-0.88408400
C	-2.83367000	-1.12838900	-1.11742500
C	-0.14471100	1.03790600	0.56630200
C	-0.61043100	1.91398100	1.51915300
N	-1.98165100	1.81917500	1.63655000
C	1.31761900	0.92275500	0.18764000
C	1.71293800	1.70769800	-1.10875400
C	1.06926500	1.18283000	-2.40038900
C	1.44495000	3.21094000	-0.94535100
C	1.80951000	-0.55620500	0.14047500
C	3.32303000	-0.60082700	-0.03451100
C	1.43661100	-1.37303600	1.40527600
O	4.12515300	-0.01651400	0.66666800
O	0.85764800	-2.43255700	1.27897300
O	3.67427600	-1.38985900	-1.06885400
C	5.08972800	-1.52096200	-1.29538900
C	1.82323800	-0.81654200	2.75986200
Cl	-5.54049000	-1.17880500	-0.73711200
H	-4.59758900	0.82871700	1.08076800
H	-0.72301100	-1.11587600	-1.43854000
H	-3.01720500	-1.91233900	-1.84360800
H	-0.05610500	2.61565600	2.12806700
H	-2.55127000	2.34829900	2.27816900
H	1.88956100	1.40097700	0.99291700
H	2.80151600	1.58983900	-1.20672400
H	-0.02160600	1.27472700	-2.37048400
H	1.42728100	1.76252200	-3.25925300
H	1.31989400	0.13373200	-2.59237600
H	1.83370000	3.76425400	-1.80798100
H	1.93209200	3.61000300	-0.04709900
H	0.37225900	3.41967100	-0.86854400
H	1.35917500	-1.09906700	-0.69001800
H	5.18593900	-2.18782100	-2.15218500
H	5.53427900	-0.54618200	-1.51222100
H	5.57873300	-1.94854200	-0.41646000
H	2.84344900	-0.42164000	2.74848000
H	1.15022500	0.01150800	3.01313300
H	1.71676900	-1.59995200	3.51303100

Compound **1a**, optimization level B3LYP/6-31G(d)

Conformation #59

C	4.21375100	-0.03164900	-0.51943700
C	3.81708600	-0.44899700	0.74310300
C	2.45737800	-0.32301300	1.04472400
C	1.50985400	0.20388800	0.12285900
C	1.96397900	0.61426800	-1.14316900
C	3.31006600	0.49504100	-1.46192400
C	0.21733100	0.17583400	0.76813100
C	0.42952600	-0.35152100	2.02007000
N	1.76911000	-0.64500900	2.19306100
C	-1.09686400	0.65391800	0.18674000
C	-1.49947900	2.04012400	0.79371400
C	-0.41660100	3.09687900	0.52239500
C	-2.85988600	2.56883400	0.31046400
C	-2.23055400	-0.40823800	0.36599800
C	-3.33368700	-0.23989400	-0.67315300
C	-1.72935500	-1.87694600	0.27395900
O	-3.15656100	0.02780300	-1.84416600
O	-1.88355900	-2.61841300	1.22387900
O	-4.54830100	-0.48677500	-0.14118700
C	-5.65319600	-0.41519100	-1.06082800
C	-1.07335400	-2.33384800	-1.01222700
Cl	5.91473300	-0.16570800	-0.96283600
H	4.53321600	-0.85197400	1.45138700
H	1.27265900	1.02435600	-1.87416800
H	3.67745800	0.80544600	-2.43379500
H	-0.27830900	-0.57061500	2.80717000
H	2.16341300	-1.08953800	3.00753600
H	-0.96379500	0.78816900	-0.89399400
H	-1.56239100	1.89518400	1.88307500
H	-0.31551800	3.28432300	-0.55459100
H	-0.68467300	4.04733900	0.99790200
H	0.56159300	2.79251800	0.90403300
H	-3.04706900	3.55920500	0.74148400
H	-3.69369500	1.92677100	0.61192100
H	-2.88360100	2.67012600	-0.78076200
H	-2.67734100	-0.32939900	1.36049900
H	-6.53795400	-0.65339300	-0.47046300
H	-5.73115300	0.58876000	-1.48600900
H	-5.52368000	-1.13700700	-1.87122400
H	-0.96085700	-3.41971800	-0.99082700
H	-1.64936900	-2.02189600	-1.88855900
H	-0.08179100	-1.87441700	-1.10055500

Compound **1a**, optimization level B3LYP/6-31G(d)

Conformation #99

C	-3.99574200	-0.35055000	-0.41889200
C	-3.73112900	0.24702900	0.80329500
C	-2.39415800	0.55658500	1.07301200
C	-1.33120800	0.29048700	0.16239200
C	-1.66037500	-0.32013200	-1.06390200
C	-2.98052300	-0.63740200	-1.34996100
C	-0.10590700	0.74386000	0.79488800
C	-0.47942800	1.24338500	2.02094400
N	-1.84445800	1.14130700	2.19160900
C	1.32814400	0.72850400	0.30444900
C	1.68725000	1.89038900	-0.68207400
C	0.98972000	1.81557300	-2.04793700
C	1.45513600	3.26009000	-0.02782600
C	1.73231400	-0.66993200	-0.25772000
C	3.16850600	-0.68280200	-0.77458700
C	1.51472400	-1.84409300	0.73202300
O	3.48323600	-0.87568800	-1.92727900
O	1.01266800	-2.87070600	0.32144800
O	4.06928200	-0.44439800	0.21014500
C	5.44765700	-0.44108300	-0.20490900
C	1.94169400	-1.68884100	2.17862700
H	-4.52670600	0.45974900	1.50953400
H	-0.89614200	-0.54779900	-1.79825400
H	-3.23968200	-1.11004100	-2.29072500
H	0.13754100	1.67861100	2.79579600
H	-2.35391400	1.42591000	3.01354800
H	1.95816900	0.90341600	1.18602000
H	2.76870000	1.80443700	-0.86167900
H	1.32295000	2.64515100	-2.68232800
H	-0.09804800	1.89308200	-1.94739600
H	1.22666600	0.88810200	-2.57950200
H	1.82891700	4.06054800	-0.67654600
H	1.97605000	3.34099000	0.93464500
H	0.38995600	3.44263900	0.15073300
H	1.12714700	-0.92614900	-1.12674700
H	5.71878700	-1.40822100	-0.63530500
H	5.62040100	0.34078300	-0.94881600
H	6.02430100	-0.24593400	0.69951600
H	2.94938300	-1.27066100	2.25249400
H	1.89539900	-2.66436300	2.66689800
H	1.25587300	-1.00242200	2.68894900
Cl	-5.65976900	-0.76570800	-0.82220800

Compound **1a**, optimization level PCM-B3LYP/6-31G(d)

Conformation #1

C	4.25111000	-0.10638800	-0.37627700
C	3.79004500	-0.20851600	0.92808100
C	2.41006600	-0.06476200	1.11190400
C	1.50973100	0.17929300	0.03483400
C	2.03250200	0.27171200	-1.26811600
C	3.39924000	0.12800200	-1.47269300
C	0.18057200	0.26410900	0.59614000
C	0.33163600	0.06818400	1.95189000
N	1.66126400	-0.12405700	2.26279100
C	-1.10129900	0.51061300	-0.16878200
C	-1.58488700	1.99950700	-0.14512000
C	-1.98641800	2.50948700	1.24637200
C	-0.54069300	2.92983900	-0.77989500
C	-2.22330900	-0.46834100	0.29234900
C	-3.46498900	-0.32273900	-0.58281200
C	-1.77953000	-1.95074800	0.27082300
O	-3.46538400	-0.36143300	-1.79941700
O	-1.94749600	-2.63908400	1.26216100
O	-4.57077500	-0.15975900	0.16018000
C	-5.81112200	-0.03230000	-0.56934700
C	-1.15958200	-2.49854500	-0.99401300
Cl	5.98688800	-0.27911400	-0.67963600
H	4.46417200	-0.39166500	1.75756300
H	1.38141800	0.45601200	-2.11783100
H	3.81695300	0.19667400	-2.47114100
H	-0.41078600	0.03707900	2.73666000
H	2.01921100	-0.30217100	3.18997000
H	-0.90765200	0.28134000	-1.22353100
H	-2.47715700	2.03742400	-0.78572600
H	-1.13923300	2.47755300	1.94096100
H	-2.32209300	3.55065600	1.17991800
H	-2.80787100	1.93048700	1.68230500
H	-0.93533100	3.94971100	-0.85348800
H	-0.27078200	2.60178500	-1.79062800
H	0.37635800	2.96802700	-0.18129900
H	-2.51283000	-0.26912000	1.32506500
H	-6.58197300	0.08968100	0.19035600
H	-5.77588300	0.84009000	-1.22564500
H	-5.99428500	-0.93045400	-1.16330800
H	-1.12953000	-3.58888500	-0.94317100
H	-1.71354900	-2.16817900	-1.87745000
H	-0.13429600	-2.12095300	-1.09074200

Compound **1a**, optimization level PCM-B3LYP/6-31G(d)

Conformation #4

C	-3.90709500	-0.05252300	-0.02940300
C	-3.32023600	-0.39642300	1.17971200
C	-1.93362500	-0.58759000	1.16494000
C	-1.14675000	-0.44210600	-0.01466700
C	-1.79532100	-0.09028300	-1.21240100
C	-3.17141100	0.10351900	-1.21976900
C	0.22609300	-0.71373100	0.34623100
C	0.21177800	-1.00220600	1.69268900
N	-1.07600000	-0.92986300	2.18269200
C	1.40668100	-0.69337700	-0.60396200
C	2.10136200	-2.08148600	-0.75425900
C	1.09693300	-3.12745400	-1.26466900
C	3.31767800	-2.03357200	-1.69303500
C	2.40535900	0.48347700	-0.32554800
C	1.63632100	1.78961800	-0.14183100
C	3.37721800	0.31424200	0.85414500
O	1.57612800	2.42673200	0.89250400
O	3.22369100	-0.53473000	1.71521200
O	1.01785200	2.14903700	-1.27772000
C	0.21395600	3.34626700	-1.20959100
C	4.57504500	1.24151000	0.86613200
Cl	-5.65945000	0.20453700	-0.08116200
H	-3.90710900	-0.50980100	2.08476600
H	-1.23293600	0.03638100	-2.13304400
H	-3.68485900	0.37525600	-2.13567600
H	1.03456900	-1.24500800	2.34696300
H	-1.33803300	-1.08931700	3.14459700
H	0.99983100	-0.45449100	-1.59475000
H	2.44589900	-2.39421400	0.23760800
H	0.23003600	-3.21429500	-0.60283100
H	0.73207100	-2.86522200	-2.26666900
H	1.57225500	-4.11286400	-1.33143900
H	3.72038900	-3.04199000	-1.84160300
H	4.13337100	-1.41736500	-1.29982200
H	3.04232300	-1.63903700	-2.68028200
H	3.01812300	0.61695100	-1.22585200
H	0.83123400	4.20120100	-0.92507000
H	-0.18969700	3.48109200	-2.21226300
H	-0.59321800	3.21793500	-0.48520900
H	4.25872500	2.28573100	0.77899100
H	5.14308700	1.10131900	1.78794900
H	5.22129900	1.02404000	0.00628900

Compound **1a**, optimization level PCM-B3LYP/6-31G(d)

Conformation #6

C	4.17285600	-0.17656500	-0.46861800
C	3.77938700	-0.10645200	0.85975800
C	2.41127000	0.07394000	1.09345700
C	1.45717600	0.18634100	0.04118700
C	1.91239200	0.10618100	-1.28771200
C	3.26627700	-0.07570400	-1.54134300
C	0.15893300	0.35508900	0.65368200
C	0.37955700	0.33459100	2.01407000
N	1.72291600	0.17070800	2.27889100
C	-1.16093400	0.51193700	-0.07070000
C	-1.63951800	1.99544300	-0.21486100
C	-1.98564700	2.67585500	1.11735500
C	-0.62091200	2.83254300	-1.00218800
C	-2.25191800	-0.38869600	0.58345700
C	-3.61084300	-0.26368200	-0.10369100
C	-1.85257300	-1.88200500	0.67678700
O	-4.63461400	0.05949700	0.46481200
O	-2.05848800	-2.48139900	1.71808400
O	-3.54784100	-0.55864300	-1.41484200
C	-4.79006300	-0.47091500	-2.14638600
C	-1.23275600	-2.56023900	-0.52404100
Cl	5.88991800	-0.40403100	-0.83508100
H	4.49469100	-0.18929600	1.67067100
H	1.21901500	0.18692900	-2.11993300
H	3.63212600	-0.13912000	-2.56028000
H	-0.31998600	0.41597700	2.83385700
H	2.12897300	0.11486300	3.20163500
H	-1.01908500	0.14760800	-1.09494000
H	-2.55744500	1.96011200	-0.81893800
H	-1.11370900	2.71783700	1.78009700
H	-2.31489700	3.70542400	0.93651100
H	-2.79540900	2.16311900	1.64750700
H	-1.01742800	3.83737300	-1.18745900
H	-0.39184800	2.37888200	-1.97372300
H	0.31935400	2.93995400	-0.44990700
H	-2.43009900	-0.08073500	1.61499600
H	-4.54247200	-0.74480500	-3.17099800
H	-5.52409300	-1.16351500	-1.72883000
H	-5.18145800	0.54784000	-2.10400200
H	-1.79818100	-2.34309200	-1.43458200
H	-0.21301100	-2.18616000	-0.67425300
H	-1.19262900	-3.63722600	-0.34898600

Compound **1a**, optimization level PCM-B3LYP/6-31G(d)

Conformation #7

C	-3.98832200	-0.34767700	-0.43242900
C	-3.73724000	0.24841200	0.79370500
C	-2.40441600	0.57655400	1.06697300
C	-1.33503900	0.33047200	0.15220200
C	-1.65604200	-0.27734600	-1.07864200
C	-2.97234400	-0.61542600	-1.36799000
C	-0.11849100	0.79570900	0.79004000
C	-0.50283400	1.27901700	2.01966800
N	-1.86405500	1.15859200	2.18773000
C	1.32229700	0.77210300	0.32010100
C	1.69716500	1.88082700	-0.71452400
C	1.02111600	1.74711600	-2.08704000
C	1.45625500	3.27942800	-0.12756900
C	1.72895500	-0.65912700	-0.13893100
C	3.14263700	-0.72730000	-0.71742200
C	1.64260400	-1.67470000	1.02680800
O	3.40576600	-1.11729300	-1.83838900
O	1.88769600	-1.34846100	2.17265200
O	4.06600500	-0.31864700	0.16815200
C	5.43613300	-0.34584800	-0.28591000
C	1.25187300	-3.08390400	0.64612700
Cl	-5.65311700	-0.79059500	-0.84070500
H	-4.53274900	0.44716000	1.50376000
H	-0.89075500	-0.48154500	-1.81951500
H	-3.22259600	-1.08294200	-2.31400600
H	0.10750000	1.70049600	2.80644000
H	-2.37655900	1.43893200	3.01110600
H	1.93370200	0.97474900	1.20619400
H	2.78099700	1.78760900	-0.87237800
H	-0.06528600	1.86343900	-2.01182200
H	1.39075100	2.52727600	-2.76279000
H	1.23234200	0.78136900	-2.55973600
H	1.83713900	4.04957900	-0.80822100
H	1.96387100	3.40187000	0.83687400
H	0.38827400	3.46706600	0.02972200
H	1.08315000	-1.01261400	-0.94448100
H	6.02818200	0.00144100	0.55978600
H	5.72216500	-1.36235300	-0.56490200
H	5.56394300	0.31896600	-1.14320600
H	1.84861700	-3.43579300	-0.20379300
H	1.37383900	-3.75715300	1.49690900
H	0.20327900	-3.09223700	0.32161400

Compound **1a**, optimization level PCM-B3LYP/6-31G(d)

Conformation #8

C	3.95598600	0.00585100	-0.12410600
C	3.36366900	-0.06107200	1.12858500
C	1.98162900	0.15640300	1.17458700
C	1.20475000	0.43243300	0.01190700
C	1.85823600	0.48603000	-1.23242100
C	3.22986600	0.27408600	-1.30026100
C	-0.16602300	0.59972700	0.43827900
C	-0.16033700	0.42071300	1.80368300
N	1.12011200	0.15747500	2.24550100
C	-1.33409200	0.93625000	-0.46758800
C	-1.97435100	2.32329200	-0.15648500
C	-0.92537000	3.43879500	-0.29336900
C	-3.17842000	2.63499500	-1.06020500
C	-2.38107600	-0.22354000	-0.59298300
C	-1.63596900	-1.51538500	-0.93429300
C	-3.36203600	-0.41283900	0.57305800
O	-1.03348400	-1.68753200	-1.97407900
O	-3.17858700	0.07185500	1.67713900
O	-1.70226600	-2.42432600	0.05275800
C	-0.99635100	-3.66379900	-0.17407300
C	-4.62291000	-1.19355700	0.25823400
Cl	5.70316800	-0.25963600	-0.25344000
H	3.94320200	-0.27060000	2.02110400
H	1.30270800	0.68585800	-2.14423100
H	3.74701600	0.31168000	-2.25288100
H	-0.98539500	0.45009200	2.49809100
H	1.37545400	-0.01687000	3.20675800
H	-0.92306800	1.01287200	-1.48275700
H	-2.32103800	2.30623000	0.88298000
H	-0.55685000	3.50626900	-1.32556900
H	-1.36344400	4.40914900	-0.03276700
H	-0.06492700	3.26951200	0.36082700
H	-3.53864900	3.65274900	-0.87172500
H	-4.02391400	1.95949900	-0.89037600
H	-2.90456800	2.57288600	-2.12192100
H	-2.98943000	-0.02132200	-1.48290200
H	-1.17182200	-4.26401600	0.71766600
H	-1.38939400	-4.16646000	-1.06042000
H	0.07036000	-3.46943500	-0.30418400
H	-4.40025000	-2.11385900	-0.29037700
H	-5.15457800	-1.42881200	1.18249700
H	-5.27456900	-0.58422600	-0.38168400

Compound **1a**, optimization level PCM-B3LYP/6-31G(d)

Conformation #9

C	-4.36580900	-0.22175000	-0.36311300
C	-3.91441600	0.59823400	0.66119900
C	-2.52985500	0.63423400	0.86295200
C	-1.61765800	-0.12056900	0.07091900
C	-2.12972800	-0.93506400	-0.95477500
C	-3.50148800	-0.98485200	-1.17131200
C	-0.28711900	0.16936400	0.55394300
C	-0.44769000	1.06001400	1.59197400
N	-1.78636600	1.33904400	1.77941200
C	0.98799500	-0.45424000	0.02892500
C	1.20968100	-1.86488500	0.68221100
C	2.16966400	-2.77637600	-0.10080300
C	1.61294400	-1.80113300	2.16263600
C	2.19626500	0.51900300	0.17087700
C	3.41767100	0.03667200	-0.60648100
C	1.87889800	1.95506300	-0.32164900
O	3.42664600	-0.22471800	-1.79468200
O	2.13396600	2.90091500	0.40362300
O	4.50417900	-0.02968300	0.18099000
C	5.73191600	-0.43036700	-0.46667300
C	1.28751800	2.13293200	-1.70068200
Cl	-6.10701800	-0.31430300	-0.67095600
H	-4.59995300	1.17792400	1.26965400
H	-1.46669500	-1.52583800	-1.58067600
H	-3.91325100	-1.60729600	-1.95814300
H	0.29169900	1.53967400	2.21697400
H	-2.15220500	1.98097900	2.46735400
H	0.85191600	-0.63006500	-1.04586200
H	0.21985200	-2.33862600	0.63460600
H	3.21603900	-2.47452200	0.01659000
H	2.09135400	-3.80321600	0.27497100
H	1.93702100	-2.79024500	-1.17126100
H	1.58175200	-2.80362700	2.60427800
H	0.93814900	-1.16033500	2.74005600
H	2.63483100	-1.42290000	2.28697000
H	2.48361300	0.62900600	1.21767400
H	6.49002700	-0.41167000	0.31508900
H	5.63121400	-1.43628800	-0.88051800
H	5.98366200	0.27074600	-1.26535900
H	1.85127000	1.55516000	-2.43886000
H	0.25746500	1.75816900	-1.71146100
H	1.28504000	3.19301800	-1.96202900

Compound **1a**, optimization level PCM-B3LYP/6-31G(d)

Conformation #16

C	4.19405800	-0.17629600	-0.46780600
C	3.80081600	-0.13328900	0.86167200
C	2.43320100	0.04921500	1.09864200
C	1.48045400	0.18865600	0.04855400
C	1.93528900	0.13342200	-1.28100100
C	3.28835500	-0.04829800	-1.53861900
C	0.18304300	0.35332300	0.66124700
C	0.40115800	0.30406100	2.02104800
N	1.74427500	0.12405200	2.28506800
C	-1.13211000	0.53604400	-0.06720600
C	-1.64421200	2.01193500	-0.09830200
C	-2.03737900	2.57357100	1.27607300
C	-0.62920900	2.93377200	-0.79023000
C	-2.19071000	-0.45788700	0.48632400
C	-3.58272500	-0.26894200	-0.11834300
C	-1.76792200	-1.92783700	0.24644300
O	-4.59053200	-0.06499500	0.53021900
O	-1.10723700	-2.24870300	-0.72312000
O	-3.56330100	-0.37619500	-1.45696400
C	-4.83333100	-0.21672600	-2.12483600
C	-2.23860600	-2.92579300	1.27879000
Cl	5.91093800	-0.40414500	-0.83889700
H	4.51562800	-0.23734800	1.67063900
H	1.24069600	0.23032300	-2.11044900
H	3.65439400	-0.09304400	-2.55853700
H	-0.29731100	0.37781500	2.84267500
H	2.14945100	0.05273600	3.20697900
H	-0.96752100	0.24369400	-1.10946200
H	-2.54734800	2.00625300	-0.72484000
H	-1.17773300	2.60143100	1.95528700
H	-2.40795800	3.59965500	1.16962800
H	-2.83150200	1.98718600	1.75127800
H	-1.04495600	3.94151200	-0.90462900
H	-0.36621400	2.56384400	-1.78835600
H	0.29519700	3.01661600	-0.20757000
H	-2.33167500	-0.31660100	1.56046700
H	-4.62019500	-0.34158500	-3.18562300
H	-5.53889600	-0.97654600	-1.78156300
H	-5.24164600	0.77737600	-1.92918000
H	-3.30066800	-2.77943100	1.50730900
H	-2.06263900	-3.94604100	0.93218000
H	-1.68743800	-2.76019100	2.21375800

Compound **1a**, optimization level PCM-B3LYP/6-31G(d)

Conformation #19

C	-3.88806300	-0.59674600	-0.42554800
C	-3.73828200	0.34092100	0.58447800
C	-2.44078700	0.81503100	0.80876800
C	-1.30876200	0.38303500	0.05206800
C	-1.52707300	-0.57163700	-0.96221400
C	-2.80680800	-1.05924900	-1.19776800
C	-0.15484700	1.08502500	0.58024200
C	-0.63353200	1.87606100	1.59944700
N	-1.99538900	1.72579800	1.73540300
C	1.31078700	1.01615300	0.19842900
C	1.69194800	1.80494400	-1.09537600
C	1.09758300	1.24777000	-2.39724200
C	1.35388700	3.29559200	-0.94394300
C	1.81598400	-0.45458100	0.18978600
C	3.31405000	-0.54654300	-0.09140500
C	1.59442900	-1.11994300	1.57358100
O	4.15984100	0.18118200	0.38865700
O	1.87896300	-0.52981700	2.59860300
O	3.58753500	-1.57143300	-0.91887500
C	4.98383000	-1.78496800	-1.21969700
C	1.03634400	-2.52219900	1.56088200
Cl	-5.50301600	-1.24011700	-0.75979000
H	-4.58279400	0.68615800	1.17097700
H	-0.71026000	-0.93214000	-1.57798700
H	-2.97842900	-1.79339900	-1.97731100
H	-0.09135700	2.54276600	2.25579300
H	-2.56815500	2.19611100	2.42095800
H	1.86339900	1.49876000	1.01238100
H	2.78448300	1.73359600	-1.17464800
H	0.00462800	1.31887800	-2.40340700
H	1.47082000	1.82161900	-3.25354400
H	1.37322700	0.20015800	-2.56562100
H	1.73452900	3.86198400	-1.80181400
H	1.80306500	3.71757900	-0.03672100
H	0.27142000	3.45638400	-0.88786500
H	1.30241400	-1.05270500	-0.56431700
H	5.00823700	-2.64587400	-1.88649000
H	5.40271900	-0.90478500	-1.71262500
H	5.54167800	-1.99135100	-0.30351700
H	0.02432500	-2.50831900	1.13706900
H	1.64335000	-3.16377900	0.91078900
H	1.00684500	-2.93169700	2.57250700

Compound **1a**, optimization level PCM-B3LYP/6-31G(d)

Conformation #33

C	-4.12972200	-0.07889200	-0.43628400
C	-3.67689100	0.61890200	0.67429900
C	-2.31870700	0.48046400	0.98297500
C	-1.43459900	-0.33081400	0.21460500
C	-1.94672800	-1.01508400	-0.90223200
C	-3.29220800	-0.88943300	-1.22654100
C	-0.12922300	-0.24254200	0.82435500
C	-0.27203000	0.60019100	1.90274800
N	-1.57993900	1.03410900	2.00083500
C	1.13302900	-0.89662300	0.30490800
C	2.06921700	-1.39327600	1.44487300
C	1.34529900	-2.44758600	2.30193700
C	3.40127200	-1.97694300	0.94849500
C	1.78123500	0.04784200	-0.77274700
C	2.44477000	1.27449900	-0.16735900
C	2.65445400	-0.69856800	-1.80605100
O	3.45878300	1.27012600	0.50706000
O	2.36434300	-1.83719900	-2.12911700
O	1.76094000	2.39520100	-0.46000000
C	2.29229000	3.62110500	0.08536800
C	3.78208900	0.05648100	-2.47239700
Cl	-5.83861800	0.05407900	-0.88299200
H	-4.34162900	1.23718700	1.26762600
H	-1.30332400	-1.64067500	-1.51494000
H	-3.70406300	-1.41052000	-2.08388000
H	0.45691800	0.93184100	2.62884100
H	-1.93629300	1.64185800	2.72396500
H	0.83974800	-1.78075600	-0.27441800
H	2.30992800	-0.53620100	2.08731800
H	1.10834400	-3.33670000	1.70289700
H	1.98564300	-2.76817600	3.13173300
H	0.41074200	-2.06657600	2.72280400
H	3.98625700	-2.33775200	1.80286800
H	4.00880100	-1.22813800	0.43332300
H	3.24050600	-2.82308800	0.27126700
H	0.94881200	0.41959700	-1.38459500
H	1.61797700	4.40599900	-0.25493700
H	2.30847900	3.57463200	1.17664400
H	3.30428600	3.79508700	-0.28737200
H	4.20886000	-0.54860600	-3.27424700
H	3.41437700	1.00662700	-2.87963300
H	4.55789600	0.30289000	-1.73949300

Compound **1a**, optimization level PCM-B3LYP/6-31G(d)

Conformation #35

C	4.25927500	-0.17961800	-0.36382000
C	3.79460700	-0.21684500	0.94259800
C	2.41845500	-0.02832800	1.11727300
C	1.52662700	0.19426700	0.02875300
C	2.05249600	0.21849300	-1.27530500
C	3.41524800	0.03231900	-1.47110800
C	0.19967200	0.33906200	0.57988500
C	0.34205200	0.19765600	1.94342600
N	1.66642200	-0.02014300	2.26721700
C	-1.07091000	0.59006300	-0.20510800
C	-1.56788100	2.07067000	-0.15567500
C	-2.02974600	2.53720700	1.23287800
C	-0.50686100	3.02681700	-0.72081300
C	-2.16720500	-0.42870600	0.20627900
C	-3.45140200	-0.25603300	-0.60273600
C	-1.69367900	-1.88378000	-0.04566000
O	-3.49600200	-0.06228000	-1.80121200
O	-1.06216800	-2.16871800	-1.04554700
O	-4.53884700	-0.38339000	0.17865300
C	-5.81438200	-0.28538600	-0.49211900
C	-2.08295400	-2.90415900	0.99662600
Cl	5.99086500	-0.41040300	-0.65625900
H	4.46294500	-0.38502200	1.77995100
H	1.40546000	0.38068900	-2.13253700
H	3.83606200	0.04833800	-2.47052900
H	-0.40005000	0.23017300	2.72869900
H	2.01899300	-0.15832600	3.20310100
H	-0.85378600	0.37264900	-1.25626500
H	-2.43401000	2.11978700	-0.82833700
H	-1.20717600	2.51191000	1.95674400
H	-2.39160200	3.57052500	1.17995300
H	-2.84953400	1.92552000	1.62679500
H	-0.90645200	4.04561000	-0.78408500
H	-0.19208600	2.72703000	-1.72746900
H	0.38461400	3.05559900	-0.08398400
H	-2.42496400	-0.33079800	1.26311900
H	-6.56274300	-0.42304800	0.28720200
H	-5.92108300	0.69657500	-0.95846100
H	-5.90299800	-1.06370100	-1.25332700
H	-3.15447200	-2.83461900	1.21746700
H	-1.83048100	-3.91124900	0.65894300
H	-1.55062700	-2.68687600	1.93150400

Compound **1a**, optimization level PCM-B3LYP/6-31G(d)

Conformation #46

C	-3.91117300	-0.59528500	-0.39061900
C	-3.74742100	0.41820100	0.54053000
C	-2.43957800	0.87430000	0.74182200
C	-1.31261400	0.34949100	0.04031700
C	-1.54407500	-0.68090800	-0.89264700
C	-2.83391200	-1.14997600	-1.10621700
C	-0.14582700	1.06239700	0.52311500
C	-0.61399800	1.95214000	1.46489000
N	-1.98015600	1.84873000	1.59456200
C	1.31722200	0.94168000	0.14813900
C	1.71415400	1.66287600	-1.18331600
C	1.08978700	1.06880900	-2.45410400
C	1.42683700	3.16895500	-1.09473000
C	1.80896600	-0.53995100	0.18068200
C	3.31881000	-0.61128700	-0.02956700
C	1.45380800	-1.25978100	1.50347200
O	4.14169000	0.02812000	0.59935900
O	0.82884100	-2.30498300	1.46490500
O	3.63461700	-1.48713900	-0.99658000
C	5.04412100	-1.65023200	-1.26775700
C	1.89622800	-0.63595300	2.80741400
Cl	-5.53938800	-1.21972000	-0.69421500
H	-4.58829400	0.83388200	1.08479400
H	-0.72872700	-1.11777200	-1.45838300
H	-3.01700300	-1.94262400	-1.82334400
H	-0.06290000	2.66662000	2.06116300
H	-2.54807300	2.39088500	2.22933500
H	1.88283900	1.46255000	0.93034200
H	2.80444100	1.55335400	-1.26763300
H	-0.00151000	1.16114100	-2.44506000
H	1.46038600	1.60427100	-3.33584100
H	1.34333800	0.01129500	-2.58804400
H	1.81975800	3.68347200	-1.97914000
H	1.89644100	3.61561200	-0.20999200
H	0.35070000	3.36750300	-1.04020500
H	1.33457300	-1.12948000	-0.60360900
H	5.10136100	-2.39146000	-2.06371400
H	5.47883000	-0.70224300	-1.59253100
H	5.56268800	-2.00367500	-0.37380400
H	2.91896500	-0.25558300	2.73812700
H	1.24418200	0.21571900	3.03625200
H	1.80947700	-1.37017600	3.61099000

Compound **1a**, optimization level PCM-B3LYP/6-31G(d)

Conformation #59

C	4.20747500	-0.09447200	-0.50688900
C	3.81995200	-0.33575400	0.80328200
C	2.45952600	-0.17197500	1.08861300
C	1.50735200	0.21999500	0.10287700
C	1.95648000	0.45278700	-1.20995400
C	3.30313200	0.29490100	-1.51362600
C	0.21900300	0.28242900	0.75169200
C	0.44074100	-0.06104000	2.06671100
N	1.77771700	-0.33277200	2.27110600
C	-1.09994100	0.68204400	0.12168500
C	-1.53048800	2.10650400	0.60851200
C	-0.46952300	3.15442000	0.23437500
C	-2.90385200	2.56863500	0.09541700
C	-2.20866400	-0.38742400	0.38940200
C	-3.33843600	-0.31934200	-0.63584100
C	-1.68330400	-1.84733500	0.38013000
O	-3.18901400	-0.12211100	-1.82673200
O	-1.84740800	-2.54748800	1.36351600
O	-4.52874600	-0.56836200	-0.06394700
C	-5.66605200	-0.59090900	-0.95393400
C	-1.00758100	-2.36352200	-0.87039300
Cl	5.91500100	-0.28344900	-0.93574700
H	4.53489800	-0.63434400	1.56222900
H	1.26349000	0.75671000	-1.98944200
H	3.66508000	0.47049600	-2.52075200
H	-0.25604200	-0.14325500	2.88922800
H	2.18202300	-0.62185700	3.14993700
H	-0.96142000	0.72681900	-0.96547400
H	-1.58366300	2.05674900	1.70618500
H	-0.38046600	3.24626400	-0.85597100
H	-0.75150600	4.13756800	0.62805100
H	0.51683100	2.90114800	0.63300500
H	-3.10643600	3.58547300	0.45084600
H	-3.72334700	1.93745300	0.45370100
H	-2.93728000	2.58579600	-1.00008600
H	-2.63869900	-0.24808200	1.38448200
H	-6.52289500	-0.82172100	-0.32237400
H	-5.79153400	0.38382300	-1.43072600
H	-5.53303200	-1.35885300	-1.71913500
H	-0.87024300	-3.44369700	-0.78972300
H	-1.59033900	-2.11849800	-1.76329500
H	-0.02803100	-1.88592800	-0.98578600

Compound **1a**, optimization level PCM-B3LYP/6-31G(d)

Conformation #99

C	-3.99138400	-0.32680100	-0.41175000
C	-3.71948000	0.24826100	0.81964100
C	-2.37903100	0.55389300	1.08264400
C	-1.32357300	0.30300700	0.15442500
C	-1.66485700	-0.28308800	-1.08106400
C	-2.98870500	-0.59628800	-1.36150800
C	-0.09374200	0.74217800	0.78556800
C	-0.45921500	1.21994500	2.02483000
N	-1.81942000	1.11581900	2.20465100
C	1.33902100	0.72426600	0.29128400
C	1.70630800	1.88217100	-0.69558500
C	0.99410100	1.82590900	-2.05477500
C	1.49940400	3.25191000	-0.03286500
C	1.73312600	-0.67685100	-0.27375100
C	3.18128800	-0.72877700	-0.75822100
C	1.46287300	-1.83799600	0.71420000
O	3.50886000	-1.06201100	-1.87972700
O	0.84691600	-2.81325900	0.32029500
O	4.05771000	-0.36941900	0.19705100
C	5.45218000	-0.39476100	-0.17836400
C	1.96556100	-1.73567000	2.13661200
H	-4.50449200	0.44901400	1.54066200
H	-0.90952100	-0.49123100	-1.83066000
H	-3.25553100	-1.04729000	-2.31096900
H	0.16271000	1.63619800	2.80561600
H	-2.31936800	1.39329900	3.03674900
H	1.97066900	0.89321200	1.17159900
H	2.78464900	1.78248900	-0.88463000
H	1.34740600	2.64339400	-2.69382300
H	-0.08980300	1.93733400	-1.94398800
H	1.19092700	0.88950600	-2.58842100
H	1.87689300	4.04949200	-0.68296600
H	2.03062000	3.31895600	0.92427500
H	0.43826900	3.44688900	0.15782800
H	1.13787700	-0.91837600	-1.15415200
H	5.74363500	-1.40256200	-0.48179900
H	5.63396100	0.30260000	-0.99908400
H	5.99740100	-0.08861800	0.71335300
H	3.00211000	-1.39014900	2.16889100
H	1.87106600	-2.70837700	2.62349500
H	1.35823800	-1.00483700	2.68366600
Cl	-5.66605400	-0.73953100	-0.80924000

Compound **1a**, optimization level B3LYP/6-311++G(2d,p)

Conformation #1

C	4.24681300	-0.06489400	-0.40071200
C	3.78068400	-0.37681700	0.86166800
C	2.40719200	-0.25653700	1.06869400
C	1.51240500	0.16806800	0.05404300
C	2.03628000	0.47140500	-1.21051800
C	3.39658400	0.35335100	-1.43466500
C	0.18427900	0.16073100	0.61886400
C	0.32802200	-0.26238800	1.91449500
N	1.65758700	-0.50259400	2.19398400
C	-1.09544600	0.52870500	-0.09281500
C	-1.53575800	2.01430600	0.11643300
C	-1.80576400	2.38957100	1.57771800
C	-0.53018900	2.98754900	-0.50892800
C	-2.24670400	-0.45366100	0.25976900
C	-3.45719300	-0.21003900	-0.63098900
C	-1.85093700	-1.94539800	0.13029100
O	-3.42374500	-0.13057200	-1.83579300
O	-2.07343500	-2.69685900	1.05141000
O	-4.58871300	-0.12036800	0.08890700
C	-5.80117100	0.05974000	-0.66951600
C	-1.21086900	-2.41289200	-1.15314300
Cl	5.97143000	-0.19807000	-0.72808700
H	4.45650600	-0.70031300	1.64234800
H	1.39068400	0.79952200	-2.01584900
H	3.81636700	0.58264100	-2.40452600
H	-0.41961500	-0.43627800	2.67114200
H	2.00671500	-0.87341000	3.06106900
H	-0.91903900	0.42013400	-1.16640100
H	-2.47484100	2.13107300	-0.43616200
H	-0.89527800	2.32287900	2.17770300
H	-2.16521300	3.41962900	1.63726200
H	-2.56549600	1.75622500	2.04073600
H	-0.89251800	4.01498700	-0.42466000
H	-0.37698100	2.77731500	-1.57025200
H	0.44037900	2.93594100	-0.01046600
H	-2.56168800	-0.33427900	1.29414400
H	-6.59782300	0.10775300	0.06779200
H	-5.75288400	0.98205500	-1.24735300
H	-5.95150100	-0.78110500	-1.34564400
H	-1.20435900	-3.50101800	-1.17544200
H	-1.73336400	-2.00963500	-2.02210000
H	-0.17969200	-2.05117400	-1.19408600

Compound **1a**, optimization level B3LYP/6-311++G(2d,p)

Conformation #4

C	-3.92276100	-0.04007600	-0.02275700
C	-3.32344800	-0.27951100	1.19877200
C	-1.94232000	-0.47022000	1.19162300
C	-1.16680900	-0.42250000	0.00478600
C	-1.82446800	-0.17972000	-1.20896500
C	-3.19555700	0.01027000	-1.22069600
C	0.20686600	-0.65816800	0.37694000
C	0.20733200	-0.83484200	1.73563800
N	-1.07776700	-0.72961500	2.22699400
C	1.37902600	-0.70820400	-0.57700500
C	2.01977600	-2.12250000	-0.69242500
C	0.97442800	-3.14353900	-1.16008300
C	3.22463500	-2.14826900	-1.64217600
C	2.41977000	0.43557800	-0.34014400
C	1.71643800	1.78197400	-0.20110400
C	3.40281800	0.25770100	0.82838600
O	1.74306200	2.48645100	0.77710400
O	3.23352300	-0.56425100	1.69924100
O	1.05502600	2.09678900	-1.32896400
C	0.29157600	3.31927000	-1.28518000
C	4.63043300	1.14068300	0.80517300
Cl	-5.66585800	0.21133400	-0.08185900
H	-3.90704300	-0.31586200	2.10932900
H	-1.27296800	-0.13704600	-2.14044200
H	-3.71790300	0.19765400	-2.14898400
H	1.03773300	-1.02013700	2.39510600
H	-1.32734400	-0.78125200	3.19971300
H	0.97190800	-0.48432400	-1.56797900
H	2.36211300	-2.41562300	0.30213700
H	0.11060200	-3.17349200	-0.49542500
H	0.61528000	-2.90631000	-2.16686700
H	1.40871800	-4.14560800	-1.19244500
H	3.58484500	-3.17196900	-1.76694200
H	4.06757700	-1.55868400	-1.27542000
H	2.95577600	-1.77333800	-2.63568500
H	3.02843200	0.51567700	-1.24708800
H	0.94440600	4.16593100	-1.07656600
H	-0.15970400	3.41318700	-2.26914800
H	-0.47635300	3.25320600	-0.51584500
H	4.34210700	2.19194600	0.75174200
H	5.22347600	0.96451800	1.70036600
H	5.23298500	0.91666800	-0.08065000

Compound **1a**, optimization level B3LYP/6-311++G(2d,p)

Conformation #6

C	4.16950200	-0.07671000	-0.49670000
C	3.76432500	-0.33127700	0.79912400
C	2.40149900	-0.20513800	1.06487100
C	1.45786800	0.16956800	0.07496900
C	1.92090900	0.41594500	-1.22521400
C	3.26970200	0.29136300	-1.50757600
C	0.15782200	0.18461700	0.70141100
C	0.36447500	-0.17788700	2.00708900
N	1.70678000	-0.40135300	2.23405400
C	-1.15742600	0.51394700	0.03517900
C	-1.59521500	2.00628200	0.19379900
C	-1.83354900	2.43852000	1.64460500
C	-0.60763900	2.95473500	-0.49501200
C	-2.27382900	-0.46187900	0.50403000
C	-3.61449300	-0.18823600	-0.16762300
C	-1.90509000	-1.95783100	0.35044600
O	-4.63052800	0.07119300	0.42415400
O	-2.13135900	-2.71134500	1.26887900
O	-3.54253200	-0.27655300	-1.51379300
C	-4.77690500	-0.04194300	-2.21984300
C	-1.27541900	-2.44065600	-0.93476800
Cl	5.87719400	-0.22024700	-0.89851900
H	4.47747300	-0.61647800	1.56130200
H	1.23721200	0.70640500	-2.01341700
H	3.64269900	0.47757500	-2.50538700
H	-0.34499300	-0.31584900	2.80668900
H	2.09882400	-0.72515900	3.10167700
H	-1.02643600	0.35573100	-1.03878300
H	-2.54946500	2.10039400	-0.33731700
H	-0.91312600	2.38009900	2.23037200
H	-2.17624900	3.47549500	1.67180400
H	-2.59647400	1.83506500	2.13985500
H	-0.97460600	3.98288600	-0.44970300
H	-0.47204700	2.69850100	-1.54894400
H	0.37278900	2.93073100	-0.01429900
H	-2.46994700	-0.33306300	1.56629700
H	-4.53703400	-0.16446800	-3.27280700
H	-5.53276800	-0.76207900	-1.90900800
H	-5.13969100	0.96615600	-2.02221600
H	-1.80068600	-2.04876300	-1.80608600
H	-0.24189700	-2.08741100	-0.98596200
H	-1.27719700	-3.52898600	-0.94425900

Compound **1a**, optimization level B3LYP/6-311++G(2d,p)

Conformation #7

C	-3.97646500	-0.33376800	-0.41748300
C	-3.70958200	0.26659600	0.79643900
C	-2.38026300	0.59671400	1.05616200
C	-1.32362700	0.35040000	0.13756000
C	-1.65614000	-0.26006300	-1.08344500
C	-2.96968200	-0.60035700	-1.35516700
C	-0.10199800	0.81542100	0.76185800
C	-0.46910700	1.29651700	1.98903300
N	-1.83036200	1.18378600	2.16899300
C	1.33387400	0.77909100	0.28509700
C	1.70484200	1.87144600	-0.76424400
C	0.97712100	1.76199900	-2.10892200
C	1.52997200	3.27509200	-0.17300600
C	1.72927500	-0.65282200	-0.15774800
C	3.16004400	-0.79056700	-0.66920300
C	1.57580300	-1.66930300	1.00487300
O	3.46139900	-1.39342600	-1.67127300
O	1.85325700	-1.37046300	2.14014300
O	4.05218400	-0.20538000	0.14538700
C	5.43371900	-0.33621400	-0.23890100
C	1.08150200	-3.04139300	0.61914000
Cl	-5.63483200	-0.77982800	-0.80443700
H	-4.50057800	0.46536200	1.50771000
H	-0.90192500	-0.46377200	-1.83123900
H	-3.23067100	-1.06992400	-2.29376300
H	0.15489000	1.70034600	2.77028100
H	-2.32897900	1.43485000	3.00525800
H	1.94775700	0.98903700	1.16373200
H	2.77463400	1.74385900	-0.96050700
H	-0.09564200	1.93207000	-1.99937900
H	1.35956200	2.51669000	-2.80072100
H	1.12512900	0.78946900	-2.58383300
H	1.89412400	4.03221400	-0.87183200
H	2.08762100	3.38760000	0.76068700
H	0.47953300	3.49104500	0.03550400
H	1.11059600	-0.99640400	-0.98424900
H	5.99810100	0.17926700	0.53343600
H	5.71514600	-1.38764700	-0.28511600
H	5.60183800	0.12338600	-1.21253600
H	1.65444600	-3.43060700	-0.22693800
H	1.14860600	-3.71831000	1.46867200
H	0.04004300	-2.96653600	0.29100000

Compound **1a**, optimization level B3LYP/6-311++G(2d,p)

Conformation #8

C	-3.89555700	-0.04933600	-0.12238300
C	-3.32304700	-0.19199800	1.12675700
C	-1.94578500	-0.40703800	1.16179600
C	-1.14998600	-0.48042800	-0.01009100
C	-1.77994500	-0.32627700	-1.25304700
C	-3.14634800	-0.11315800	-1.30609700
C	0.21215000	-0.70943000	0.40716000
C	0.18731800	-0.76049600	1.77556100
N	-1.10317200	-0.58390800	2.23268400
C	1.39875700	-0.88715600	-0.51349300
C	2.13149900	-2.24502400	-0.30916100
C	1.16774800	-3.40936800	-0.57515200
C	3.37216600	-2.39237300	-1.19984000
C	2.35820800	0.34744100	-0.53903800
C	1.54442300	1.59408300	-0.87847400
C	3.27219900	0.55030000	0.67476700
O	1.08318400	1.80861600	-1.97093100
O	3.13965700	-0.06365500	1.70867400
O	1.37806500	2.40823400	0.17714500
C	0.55028100	3.56741600	-0.04882100
C	4.42368600	1.51633600	0.48120100
Cl	-5.63239400	0.22756400	-0.23559300
H	-3.92299600	-0.13805900	2.02577300
H	-1.20890900	-0.35701100	-2.17279200
H	-3.64702600	0.01060700	-2.25661500
H	1.00234900	-0.89377300	2.46590500
H	-1.37425700	-0.56679200	3.20076200
H	0.99564700	-0.91392500	-1.53161200
H	2.45428200	-2.29811100	0.73303700
H	0.84291100	-3.41732200	-1.62058200
H	1.65838200	-4.36474800	-0.37426800
H	0.27722900	-3.35024600	0.05122800
H	3.79169200	-3.39573300	-1.09721400
H	4.16657500	-1.68914700	-0.94112600
H	3.12300700	-2.24875800	-2.25658500
H	3.02087900	0.22804100	-1.40141800
H	0.53565200	4.10017100	0.89811600
H	0.97520700	4.18846400	-0.83660800
H	-0.45411200	3.25762300	-0.33353900
H	4.11783800	2.41720700	-0.05237200
H	4.84902700	1.77818400	1.44819700
H	5.19566600	1.02558300	-0.12097400

Compound **1a**, optimization level B3LYP/6-311++G(2d,p)

Conformation #9

C	-4.36090500	-0.21730000	-0.36689000
C	-3.90419500	0.62866600	0.62559300
C	-2.52570800	0.66539300	0.83058100
C	-1.61866500	-0.11578200	0.07156300
C	-2.13194700	-0.95789500	-0.92414500
C	-3.49806800	-1.00634100	-1.14096900
C	-0.28926200	0.18591000	0.54349300
C	-0.44213400	1.11041000	1.54264600
N	-1.78109900	1.39733200	1.72436700
C	0.97956000	-0.45744000	0.03775100
C	1.16889300	-1.86358300	0.70467200
C	2.13128200	-2.78898700	-0.05199500
C	1.53808300	-1.79831700	2.19103100
C	2.20174200	0.49127800	0.17905000
C	3.41088600	0.00287500	-0.60645000
C	1.91611200	1.93863500	-0.30532800
O	3.40797800	-0.26772600	-1.78307800
O	2.19319000	2.86524100	0.42157200
O	4.51266300	-0.04217800	0.16392100
C	5.72927800	-0.42517000	-0.50677000
C	1.32594300	2.14106800	-1.67823800
Cl	-6.09198200	-0.30970700	-0.67488500
H	-4.59139600	1.22862800	1.20750500
H	-1.47411000	-1.57135700	-1.52831900
H	-3.91205700	-1.64936600	-1.90547700
H	0.30119000	1.61334400	2.13940100
H	-2.14147800	2.08405700	2.36437800
H	0.84881800	-0.64196800	-1.03313500
H	0.17740700	-2.32387900	0.64101600
H	3.17478900	-2.49337900	0.07456500
H	2.04180900	-3.80808300	0.33279200
H	1.91741700	-2.81288500	-1.12240300
H	1.49067200	-2.79679500	2.63206300
H	0.85415500	-1.15798400	2.75119500
H	2.55563700	-1.42784300	2.34050900
H	2.49548000	0.59447700	1.22145000
H	6.50079900	-0.39860400	0.25792900
H	5.63305200	-1.42713300	-0.92403300
H	5.95559900	0.27745700	-1.30790200
H	1.86699000	1.55452500	-2.42231700
H	0.28846400	1.79696300	-1.68280600
H	1.35213100	3.20029700	-1.92683500

Compound **1a**, optimization level B3LYP/6-311++G(2d,p)

Conformation #16

C	4.17104400	-0.19499400	-0.47562700
C	3.78761800	-0.09070900	0.84685200
C	2.42718400	0.09975700	1.08833900
C	1.46919900	0.18996800	0.04847300
C	1.90889100	0.06837200	-1.27635500
C	3.25413000	-0.12157200	-1.53483800
C	0.17869800	0.38405300	0.66142700
C	0.40243100	0.39435900	2.01408300
N	1.74909100	0.22619100	2.27708500
C	-1.13708700	0.54893200	-0.06543200
C	-1.65549800	2.01978400	-0.10635900
C	-2.00574200	2.61209000	1.26373500
C	-0.66974000	2.92917200	-0.84900600
C	-2.18607200	-0.44404100	0.49120800
C	-3.57668500	-0.32742100	-0.12640700
C	-1.73371700	-1.91451000	0.29065500
O	-4.60066200	-0.32469000	0.51394100
O	-1.11973500	-2.25400500	-0.68974200
O	-3.53481700	-0.27571400	-1.46688300
C	-4.80963000	-0.21678200	-2.13420700
C	-2.13068500	-2.88158900	1.37960600
Cl	5.87532600	-0.43460700	-0.85021200
H	4.51374000	-0.15712300	1.64634800
H	1.20650800	0.11054500	-2.09930600
H	3.61103300	-0.22037600	-2.55079700
H	-0.29065300	0.51189300	2.83150600
H	2.16172600	0.19718500	3.19334800
H	-0.96892400	0.25097500	-1.10174100
H	-2.57593000	1.99750800	-0.69913500
H	-1.11759800	2.71231600	1.89196200
H	-2.43151400	3.61079400	1.14014600
H	-2.74359100	2.01287800	1.80241700
H	-1.08138800	3.93632500	-0.95136600
H	-0.45664800	2.55298500	-1.85262400
H	0.27926600	3.00973200	-0.31387400
H	-2.34659000	-0.28550200	1.55722200
H	-4.57880500	-0.19526700	-3.19578100
H	-5.40623500	-1.09416100	-1.88710500
H	-5.35149100	0.68102200	-1.83800100
H	-3.19035100	-2.77083200	1.62478900
H	-1.91411900	-3.90298700	1.07262600
H	-1.56692400	-2.64686500	2.28796900

Compound **1a**, optimization level B3LYP/6-311++G(2d,p)

Conformation #19

C	-3.88604700	-0.57655200	-0.43224400
C	-3.71335400	0.25552200	0.65553600
C	-2.42107800	0.72233200	0.89184700
C	-1.31037400	0.38575500	0.07069800
C	-1.54732500	-0.46260500	-1.02368800
C	-2.82278300	-0.93988900	-1.26990400
C	-0.14950500	1.04169200	0.63604300
C	-0.60149100	1.71494800	1.73827500
N	-1.95973700	1.54159400	1.89225200
C	1.30490000	1.02592400	0.21628300
C	1.64114500	1.92908700	-1.01093500
C	0.98602700	1.51113700	-2.33240200
C	1.32992500	3.39842900	-0.70302300
C	1.83095800	-0.42381400	0.07358000
C	3.33390000	-0.48602800	-0.16971600
C	1.58354300	-1.23611200	1.37653600
O	4.14230000	0.33041000	0.18524700
O	1.89079000	-0.78061100	2.45028200
O	3.66176700	-1.63182900	-0.80979500
C	5.07090500	-1.83911500	-1.02892200
C	0.96643500	-2.60237600	1.21497700
Cl	-5.49312000	-1.20398100	-0.78236700
H	-4.54688400	0.52510200	1.29086400
H	-0.74734700	-0.74725800	-1.69356000
H	-3.01082000	-1.59217700	-2.11178800
H	-0.04039700	2.30227600	2.44759600
H	-2.51066700	1.90995200	2.64845400
H	1.86785400	1.44669400	1.05270000
H	2.72389800	1.85707000	-1.14014200
H	-0.10020400	1.61463300	-2.29598400
H	1.34851700	2.14765000	-3.14354200
H	1.22117200	0.47978800	-2.60800200
H	1.66942000	4.03911100	-1.52066700
H	1.83456700	3.72873100	0.20863000
H	0.25747500	3.56267300	-0.57329900
H	1.34698900	-0.95045100	-0.74762400
H	5.14820300	-2.79964100	-1.53151300
H	5.47729000	-1.04454000	-1.65376600
H	5.60320400	-1.85601000	-0.07848400
H	-0.04086900	-2.49795900	0.80108200
H	1.54704200	-3.19448800	0.50260700
H	0.91920200	-3.10840100	2.17739000

Compound **1a**, optimization level B3LYP/6-311++G(2d,p)

Conformation #33

C	4.15235800	0.09182500	0.44223800
C	3.69122000	0.50288900	-0.79373400
C	2.34193200	0.27769400	-1.06340000
C	1.46990500	-0.34244900	-0.13353700
C	1.98632300	-0.74206700	1.10677200
C	3.32310100	-0.52412700	1.39097800
C	0.16774700	-0.41933300	-0.74611800
C	0.29704300	0.14638000	-1.98601600
N	1.59970700	0.56810600	-2.18296700
C	-1.08345500	-0.96479900	-0.09832000
C	-1.95316900	-1.79044300	-1.08706200
C	-1.17427600	-3.03388100	-1.54404100
C	-3.30961200	-2.22281200	-0.51956100
C	-1.78524700	0.19542500	0.68130900
C	-2.41938500	1.25108900	-0.20451900
C	-2.70467300	-0.29798600	1.82604000
O	-3.12762500	1.04793300	-1.16069000
O	-2.36101800	-1.24741200	2.48923200
O	-2.10408300	2.49343300	0.22245800
C	-2.67226800	3.57867000	-0.53351000
C	-3.95702700	0.48330400	2.14347000
Cl	5.84769000	0.34934000	0.84454600
H	4.35361600	0.97425300	-1.50787000
H	1.35312200	-1.22142900	1.84404100
H	3.74046400	-0.82630700	2.34170800
H	-0.43856000	0.28308600	-2.76205600
H	1.95167600	0.98545400	-3.02714000
H	-0.77232500	-1.65131200	0.69443800
H	-2.15533500	-1.16917200	-1.96284700
H	-0.99567800	-3.70968500	-0.70193800
H	-1.74471600	-3.58626100	-2.29466500
H	-0.20725700	-2.77729600	-1.97822900
H	-3.84042300	-2.82842500	-1.25839900
H	-3.94970400	-1.36912100	-0.29346200
H	-3.19533700	-2.82463800	0.38474500
H	-0.99100800	0.70951000	1.23290100
H	-2.31688800	4.48539700	-0.05122600
H	-2.33477000	3.53605000	-1.56845700
H	-3.76079200	3.53034200	-0.51042800
H	-4.46101000	0.03328700	2.99619300
H	-3.69804600	1.52114600	2.37183800
H	-4.62698700	0.50168000	1.28040000

Compound **1a**, optimization level B3LYP/6-311++G(2d,p)

Conformation #35

C	4.21568500	-0.26937900	-0.38882000
C	3.77756400	-0.16469900	0.91659400
C	2.41481700	0.06947800	1.09959800
C	1.50802900	0.20163900	0.01932300
C	2.00153700	0.07783600	-1.28630700
C	3.34990300	-0.15497900	-1.48679300
C	0.19832600	0.43479000	0.57574300
C	0.36156700	0.42593900	1.93714600
N	1.68865000	0.20787000	2.25846900
C	-1.07582100	0.65329600	-0.20989900
C	-1.55881100	2.13676900	-0.22838600
C	-1.96935400	2.69442700	1.13968500
C	-0.51008300	3.04390000	-0.88259000
C	-2.17296800	-0.33730400	0.24570200
C	-3.44212800	-0.25232100	-0.59369700
C	-1.67731100	-1.80503000	0.11756400
O	-3.50552200	0.09503700	-1.74315700
O	-1.12459000	-2.18107700	-0.88548600
O	-4.51213800	-0.67418900	0.11892800
C	-5.76141000	-0.70745500	-0.59911400
C	-1.94432700	-2.71496700	1.29144100
Cl	5.92601000	-0.56288900	-0.69002100
H	4.46488800	-0.26310300	1.74651300
H	1.33817500	0.15203700	-2.13884800
H	3.74826400	-0.25582400	-2.48701300
H	-0.36266500	0.56271400	2.72398200
H	2.06027600	0.16511000	3.19157800
H	-0.86485000	0.38966900	-1.24789400
H	-2.44120700	2.15321500	-0.87283800
H	-1.11518400	2.76285800	1.81758900
H	-2.37394500	3.70292300	1.02429200
H	-2.74284200	2.09286600	1.62467700
H	-0.89194200	4.06378600	-0.97424200
H	-0.25239300	2.69497700	-1.88535800
H	0.40980400	3.08371800	-0.29424600
H	-2.45691300	-0.16758500	1.28450800
H	-6.49459600	-1.08037100	0.11120700
H	-6.02898100	0.29257300	-0.93854800
H	-5.68530800	-1.37029900	-1.46035900
H	-3.00296800	-2.68194800	1.56141700
H	-1.64524800	-3.73355500	1.05157700
H	-1.37836800	-2.35954100	2.15766600

Compound **1a**, optimization level B3LYP/6-311++G(2d,p)

Conformation #46

C	-3.91466700	-0.57557000	-0.40451600
C	-3.74645800	0.40431200	0.55207200
C	-2.44197900	0.84349100	0.77604900
C	-1.31937900	0.33518500	0.07108400
C	-1.55018200	-0.66561700	-0.88638800
C	-2.83653000	-1.11456100	-1.12042900
C	-0.15129200	1.02505500	0.57832400
C	-0.61252300	1.88496800	1.54033000
N	-1.98163600	1.78915900	1.66048700
C	1.30796900	0.92398500	0.19324800
C	1.69226400	1.74268300	-1.08246800
C	0.98298300	1.30247300	-2.36786200
C	1.49792800	3.24532200	-0.85093800
C	1.81205900	-0.54348000	0.09957000
C	3.32765700	-0.57807700	-0.04926300
C	1.44585200	-1.42080500	1.32326600
O	4.10670700	0.01416800	0.65917200
O	0.93299400	-2.49974500	1.14283000
O	3.70407200	-1.38130800	-1.05875600
C	5.12547000	-1.52569600	-1.24869000
C	1.76495300	-0.90375000	2.70530500
Cl	-5.53607300	-1.17390000	-0.73755900
H	-4.59049900	0.80519100	1.09778500
H	-0.73566500	-1.10356700	-1.44589700
H	-3.02114500	-1.88855400	-1.85257500
H	-0.05719200	2.57482700	2.15650300
H	-2.54692000	2.30607300	2.31177400
H	1.87628500	1.38344300	1.00698700
H	2.76699000	1.58426500	-1.22600700
H	-0.09479100	1.46449000	-2.30506400
H	1.35529300	1.88367500	-3.21509000
H	1.15555500	0.24974000	-2.60053400
H	1.86833600	3.81343900	-1.70762500
H	2.04163700	3.58705900	0.03392100
H	0.44274900	3.49486400	-0.71749600
H	1.38213100	-1.05801800	-0.75546200
H	5.23627800	-2.20186300	-2.09196600
H	5.57865200	-0.55907200	-1.46593700
H	5.58464000	-1.94585800	-0.35459600
H	2.76203000	-0.46188300	2.73934100
H	1.04778100	-0.12162700	2.96761700
H	1.67761700	-1.71886400	3.42128300

Compound **1a**, optimization level B3LYP/6-311++G(2d,p)

Conformation #59

C	4.19842100	-0.06166000	-0.51994700
C	3.80732000	-0.40472700	0.75995500
C	2.45354000	-0.26004200	1.06011900
C	1.50625900	0.21352300	0.11693600
C	1.95451000	0.55074200	-1.16781000
C	3.29480000	0.41159000	-1.48261400
C	0.21931300	0.22293700	0.76595100
C	0.43375500	-0.23115100	2.04024400
N	1.77205700	-0.51460700	2.22620000
C	-1.09339200	0.66957500	0.16355500
C	-1.50616900	2.07108200	0.71917300
C	-0.42312800	3.11893700	0.43035400
C	-2.85736900	2.58283000	0.20289200
C	-2.21911800	-0.39033400	0.37488100
C	-3.32271000	-0.27322300	-0.66831000
C	-1.71834800	-1.85928600	0.33728600
O	-3.14827300	-0.05214000	-1.84207800
O	-1.90521200	-2.57007200	1.29743600
O	-4.53156000	-0.51167600	-0.12853400
C	-5.64266800	-0.49246500	-1.04540400
C	-1.03345400	-2.36017400	-0.91074600
Cl	5.89463600	-0.22380800	-0.96306900
H	4.52455100	-0.76745000	1.48443400
H	1.26524000	0.91878500	-1.91826700
H	3.65784500	0.66561300	-2.46905800
H	-0.26886000	-0.39934100	2.84058000
H	2.16613200	-0.91678500	3.05937300
H	-0.95297900	0.76792500	-0.91665400
H	-1.58364200	1.96094900	1.80816200
H	-0.31027900	3.27429500	-0.64702500
H	-0.69816400	4.07908100	0.87313300
H	0.54819400	2.83044800	0.83168200
H	-3.04748400	3.58281900	0.60015400
H	-3.69418400	1.95528000	0.51357100
H	-2.86712700	2.65141400	-0.88772300
H	-2.66815800	-0.27800100	1.36162600
H	-6.51828900	-0.71599600	-0.44206700
H	-5.73340000	0.48955500	-1.50877400
H	-5.50670300	-1.24492300	-1.82139300
H	-0.92782000	-3.44175500	-0.85113600
H	-1.58552200	-2.07470000	-1.80755700
H	-0.04157400	-1.90804200	-0.98798800

Compound **1a**, optimization level B3LYP/6-311++G(2d,p)

Conformation #99

C	-3.99574200	-0.35055000	-0.41889200
C	-3.73112900	0.24702900	0.80329500
C	-2.39415800	0.55658500	1.07301200
C	-1.33120800	0.29048700	0.16239200
C	-1.66037500	-0.32013200	-1.06390200
C	-2.98052300	-0.63740200	-1.34996100
C	-0.10590700	0.74386000	0.79488800
C	-0.47942800	1.24338500	2.02094400
N	-1.84445800	1.14130700	2.19160900
C	1.32814400	0.72850400	0.30444900
C	1.68725000	1.89038900	-0.68207400
C	0.98972000	1.81557300	-2.04793700
C	1.45513600	3.26009000	-0.02782600
C	1.73231400	-0.66993200	-0.25772000
C	3.16850600	-0.68280200	-0.77458700
C	1.51472400	-1.84409300	0.73202300
O	3.48323600	-0.87568800	-1.92727900
O	1.01266800	-2.87070600	0.32144800
O	4.06928200	-0.44439800	0.21014500
C	5.44765700	-0.44108300	-0.20490900
C	1.94169400	-1.68884100	2.17862700
H	-4.52670600	0.45974900	1.50953400
H	-0.89614200	-0.54779900	-1.79825400
H	-3.23968200	-1.11004100	-2.29072500
H	0.13754100	1.67861100	2.79579600
H	-2.35391400	1.42591000	3.01354800
H	1.95816900	0.90341600	1.18602000
H	2.76870000	1.80443700	-0.86167900
H	1.32295000	2.64515100	-2.68232800
H	-0.09804800	1.89308200	-1.94739600
H	1.22666600	0.88810200	-2.57950200
H	1.82891700	4.06054800	-0.67654600
H	1.97605000	3.34099000	0.93464500
H	0.38995600	3.44263900	0.15073300
H	1.12714700	-0.92614900	-1.12674700
H	5.71878700	-1.40822100	-0.63530500
H	5.62040100	0.34078300	-0.94881600
H	6.02430100	-0.24593400	0.69951600
H	2.94938300	-1.27066100	2.25249400
H	1.89539900	-2.66436300	2.66689800
H	1.25587300	-1.00242200	2.68894900
Cl	-5.65976900	-0.76570800	-0.82220800

Compound **1a**, optimization level PCM-B3LYP/6-311++G(2d,p)

Conformation #1

C	4.24681300	-0.06489400	-0.40071200
C	3.78068400	-0.37681700	0.86166800
C	2.40719200	-0.25653700	1.06869400
C	1.51240500	0.16806800	0.05404300
C	2.03628000	0.47140500	-1.21051800
C	3.39658400	0.35335100	-1.43466500
C	0.18427900	0.16073100	0.61886400
C	0.32802200	-0.26238800	1.91449500
N	1.65758700	-0.50259400	2.19398400
C	-1.09544600	0.52870500	-0.09281500
C	-1.53575800	2.01430600	0.11643300
C	-1.80576400	2.38957100	1.57771800
C	-0.53018900	2.98754900	-0.50892800
C	-2.24670400	-0.45366100	0.25976900
C	-3.45719300	-0.21003900	-0.63098900
C	-1.85093700	-1.94539800	0.13029100
O	-3.42374500	-0.13057200	-1.83579300
O	-2.07343500	-2.69685900	1.05141000
O	-4.58871300	-0.12036800	0.08890700
C	-5.80117100	0.05974000	-0.66951600
C	-1.21086900	-2.41289200	-1.15314300
Cl	5.97143000	-0.19807000	-0.72808700
H	4.45650600	-0.70031300	1.64234800
H	1.39068400	0.79952200	-2.01584900
H	3.81636700	0.58264100	-2.40452600
H	-0.41961500	-0.43627800	2.67114200
H	2.00671500	-0.87341000	3.06106900
H	-0.91903900	0.42013400	-1.16640100
H	-2.47484100	2.13107300	-0.43616200
H	-0.89527800	2.32287900	2.17770300
H	-2.16521300	3.41962900	1.63726200
H	-2.56549600	1.75622500	2.04073600
H	-0.89251800	4.01498700	-0.42466000
H	-0.37698100	2.77731500	-1.57025200
H	0.44037900	2.93594100	-0.01046600
H	-2.56168800	-0.33427900	1.29414400
H	-6.59782300	0.10775300	0.06779200
H	-5.75288400	0.98205500	-1.24735300
H	-5.95150100	-0.78110500	-1.34564400
H	-1.20435900	-3.50101800	-1.17544200
H	-1.73336400	-2.00963500	-2.02210000
H	-0.17969200	-2.05117400	-1.19408600

Compound **1a**, optimization level PCM-B3LYP/6-311++G(2d,p)

Conformation #4

C	-3.92273100	-0.04043000	-0.02279700
C	-3.32337000	-0.27841600	1.19895800
C	-1.94220600	-0.46899400	1.19193400
C	-1.16676700	-0.42254300	0.00499600
C	-1.82452800	-0.18115200	-1.20897500
C	-3.19564100	0.00868400	-1.22083200
C	0.20696400	-0.65754900	0.37734300
C	0.20755200	-0.83259500	1.73625700
N	-1.07753000	-0.72693400	2.22755100
C	1.37904800	-0.70843800	-0.57663900
C	2.01984400	-2.12281300	-0.69089500
C	0.97454700	-3.14433300	-1.15762600
C	3.22464300	-2.14928800	-1.64071700
C	2.41981500	0.43551900	-0.34086300
C	1.71644300	1.78194900	-0.20208600
C	3.40332700	0.25831500	0.82741300
O	1.74433300	2.48734200	0.77542600
O	3.23396600	-0.56270500	1.69913500
O	1.05339200	2.09559300	-1.32929400
C	0.28963800	3.31782400	-1.28551100
C	4.63137400	1.14055200	0.80277700
Cl	-5.66591800	0.21073000	-0.08209900
H	-3.90687000	-0.31377400	2.10960900
H	-1.27310100	-0.13942500	-2.14054000
H	-3.71806200	0.19500600	-2.14928600
H	1.03804700	-1.01692800	2.39587500
H	-1.32707300	-0.77773800	3.20031800
H	0.97183500	-0.48542100	-1.56776700
H	2.36224500	-2.41503600	0.30390900
H	0.11079400	-3.17383200	-0.49285600
H	0.61526600	-2.90796000	-2.16456300
H	1.40894200	-4.14637900	-1.18921600
H	3.58504000	-3.17303500	-1.76450800
H	4.06750700	-1.55916600	-1.27463400
H	2.95561400	-1.77536700	-2.63456200
H	3.02798900	0.51525500	-1.24815200
H	0.94240400	4.16487400	-1.07825500
H	-0.16283400	3.41092500	-2.26901900
H	-0.47739300	3.25206600	-0.51525100
H	4.34353100	2.19192600	0.74850900
H	5.22480700	0.96502100	1.69782700
H	5.23337300	0.91551000	-0.08316800

Compound **1a**, optimization level PCM-B3LYP/6-311++G(2d,p)

Conformation #6

C	4.16959500	-0.07641600	-0.49665700
C	3.76441900	-0.33150700	0.79905400
C	2.40158200	-0.20550800	1.06483300
C	1.45793800	0.16954100	0.07507300
C	1.92099200	0.41641900	-1.22500800
C	3.26979600	0.29200100	-1.50739900
C	0.15787600	0.18423300	0.70148300
C	0.36454400	-0.17863400	2.00706400
N	1.70683800	-0.40224300	2.23390900
C	-1.15736500	0.51373100	0.03534200
C	-1.59524900	2.00599000	0.19448900
C	-1.83337400	2.43783400	1.64544800
C	-0.60789900	2.95473500	-0.49425000
C	-2.27374900	-0.46235000	0.50378200
C	-3.61452900	-0.18824100	-0.16739500
C	-1.90513500	-1.95825200	0.34923600
O	-4.63047400	0.07072000	0.42474400
O	-2.13111000	-2.71222700	1.26737700
O	-3.54280000	-0.27540500	-1.51366300
C	-4.77730300	-0.04023500	-2.21925100
C	-1.27570500	-2.44041100	-0.93632700
Cl	5.87730300	-0.21975100	-0.89852700
H	4.47755100	-0.61699000	1.56114100
H	1.23729800	0.70712700	-2.01312300
H	3.64278900	0.47858800	-2.50513900
H	-0.34491800	-0.31687700	2.80662100
H	2.09888000	-0.72641200	3.10139600
H	-1.02635600	0.35593400	-1.03867600
H	-2.54960000	2.10015400	-0.33643800
H	-0.91286300	2.37924000	2.23106100
H	-2.17604800	3.47480600	1.67298500
H	-2.59623300	1.83425500	2.14064600
H	-0.97501900	3.98282200	-0.44868800
H	-0.47239000	2.69873300	-1.54824600
H	0.37258900	2.93078400	-0.01366100
H	-2.46961500	-0.33419200	1.56616800
H	-4.53763500	-0.16177600	-3.27238300
H	-5.53305900	-0.76071500	-1.90895200
H	-5.14011900	0.96766000	-2.02064700
H	-1.80110100	-2.04803200	-1.80735000
H	-0.24217400	-2.08719100	-0.98750100
H	-1.27753000	-3.52873400	-0.94640200

Compound **1a**, optimization level PCM-B3LYP/6-311++G(2d,p)

Conformation #7

C	-3.97646500	-0.33376800	-0.41748300
C	-3.70958200	0.26659600	0.79643900
C	-2.38026300	0.59671400	1.05616200
C	-1.32362700	0.35040000	0.13756000
C	-1.65614000	-0.26006300	-1.08344500
C	-2.96968200	-0.60035700	-1.35516700
C	-0.10199800	0.81542100	0.76185800
C	-0.46910700	1.29651700	1.98903300
N	-1.83036200	1.18378600	2.16899300
C	1.33387400	0.77909100	0.28509700
C	1.70484200	1.87144600	-0.76424400
C	0.97712100	1.76199900	-2.10892200
C	1.52997200	3.27509200	-0.17300600
C	1.72927500	-0.65282200	-0.15774800
C	3.16004400	-0.79056700	-0.66920300
C	1.57580300	-1.66930300	1.00487300
O	3.46139900	-1.39342600	-1.67127300
O	1.85325700	-1.37046300	2.14014300
O	4.05218400	-0.20538000	0.14538700
C	5.43371900	-0.33621400	-0.23890100
C	1.08150200	-3.04139300	0.61914000
Cl	-5.63483200	-0.77982800	-0.80443700
H	-4.50057800	0.46536200	1.50771000
H	-0.90192500	-0.46377200	-1.83123900
H	-3.23067100	-1.06992400	-2.29376300
H	0.15489000	1.70034600	2.77028100
H	-2.32897900	1.43485000	3.00525800
H	1.94775700	0.98903700	1.16373200
H	2.77463400	1.74385900	-0.96050700
H	-0.09564200	1.93207000	-1.99937900
H	1.35956200	2.51669000	-2.80072100
H	1.12512900	0.78946900	-2.58383300
H	1.89412400	4.03221400	-0.87183200
H	2.08762100	3.38760000	0.76068700
H	0.47953300	3.49104500	0.03550400
H	1.11059600	-0.99640400	-0.98424900
H	5.99810100	0.17926700	0.53343600
H	5.71514600	-1.38764700	-0.28511600
H	5.60183800	0.12338600	-1.21253600
H	1.65444600	-3.43060700	-0.22693800
H	1.14860600	-3.71831000	1.46867200
H	0.04004300	-2.96653600	0.29100000

Compound **1a**, optimization level PCM-B3LYP/6-311++G(2d,p)

Conformation #8

C	-3.89555700	-0.04933600	-0.12238300
C	-3.32304700	-0.19199800	1.12675700
C	-1.94578500	-0.40703800	1.16179600
C	-1.14998600	-0.48042800	-0.01009100
C	-1.77994500	-0.32627700	-1.25304700
C	-3.14634800	-0.11315800	-1.30609700
C	0.21215000	-0.70943000	0.40716000
C	0.18731800	-0.76049600	1.77556100
N	-1.10317200	-0.58390800	2.23268400
C	1.39875700	-0.88715600	-0.51349300
C	2.13149900	-2.24502400	-0.30916100
C	1.16774800	-3.40936800	-0.57515200
C	3.37216600	-2.39237300	-1.19984000
C	2.35820800	0.34744100	-0.53903800
C	1.54442300	1.59408300	-0.87847400
C	3.27219900	0.55030000	0.67476700
O	1.08318400	1.80861600	-1.97093100
O	3.13965700	-0.06365500	1.70867400
O	1.37806500	2.40823400	0.17714500
C	0.55028100	3.56741600	-0.04882100
C	4.42368600	1.51633600	0.48120100
Cl	-5.63239400	0.22756400	-0.23559300
H	-3.92299600	-0.13805900	2.02577300
H	-1.20890900	-0.35701100	-2.17279200
H	-3.64702600	0.01060700	-2.25661500
H	1.00234900	-0.89377300	2.46590500
H	-1.37425700	-0.56679200	3.20076200
H	0.99564700	-0.91392500	-1.53161200
H	2.45428200	-2.29811100	0.73303700
H	0.84291100	-3.41732200	-1.62058200
H	1.65838200	-4.36474800	-0.37426800
H	0.27722900	-3.35024600	0.05122800
H	3.79169200	-3.39573300	-1.09721400
H	4.16657500	-1.68914700	-0.94112600
H	3.12300700	-2.24875800	-2.25658500
H	3.02087900	0.22804100	-1.40141800
H	0.53565200	4.10017100	0.89811600
H	0.97520700	4.18846400	-0.83660800
H	-0.45411200	3.25762300	-0.33353900
H	4.11783800	2.41720700	-0.05237200
H	4.84902700	1.77818400	1.44819700
H	5.19566600	1.02558300	-0.12097400

Compound **1a**, optimization level PCM-B3LYP/6-311++G(2d,p)

Conformation #9

C	-4.36105800	-0.21737200	-0.36643500
C	-3.90410300	0.62885400	0.62579400
C	-2.52560600	0.66577100	0.83045600
C	-1.61872700	-0.11557200	0.07146800
C	-2.13212200	-0.95797600	-0.92394100
C	-3.49826200	-1.00654600	-1.14050700
C	-0.28923500	0.18622400	0.54310000
C	-0.44192300	1.11071700	1.54225200
N	-1.78089300	1.39787400	1.72406900
C	0.97961100	-0.45719500	0.03732800
C	1.16850000	-1.86349200	0.70408700
C	2.13069800	-2.78915800	-0.05250500
C	1.53763400	-1.79832200	2.19045700
C	2.20174400	0.49131200	0.17900500
C	3.41103200	0.00283300	-0.60634800
C	1.91652600	1.93878700	-0.30545500
O	3.40814000	-0.26746000	-1.78307700
O	2.19384300	2.86533300	0.42136300
O	4.51254200	-0.04287300	0.16417800
C	5.72927500	-0.42623100	-0.50629400
C	1.32641200	2.14131800	-1.67846400
Cl	-6.09208100	-0.30987700	-0.67403100
H	-4.59133400	1.22884000	1.20766000
H	-1.47430200	-1.57149500	-1.52807300
H	-3.91239500	-1.64975700	-1.90479600
H	0.30148700	1.61375400	2.13881000
H	-2.14115400	2.08495000	2.36377900
H	0.84898900	-0.64144300	-1.03363000
H	0.17689500	-2.32348400	0.64035600
H	3.17431300	-2.49406300	0.07453200
H	2.04059100	-3.80828800	0.33202000
H	1.91724100	-2.81269400	-1.12300000
H	1.49022600	-2.79682200	2.63145800
H	0.85368600	-1.15801500	2.75063400
H	2.55517600	-1.42784800	2.34001100
H	2.49532900	0.59452100	1.22146600
H	6.50053500	-0.40007700	0.25864000
H	5.63268300	-1.42804000	-0.92382500
H	5.95599300	0.27645000	-1.30727100
H	1.86760400	1.55493700	-2.42252800
H	0.28898800	1.79705000	-1.68306600
H	1.35249100	3.20060400	-1.92683000

Compound **1a**, optimization level PCM-B3LYP/6-311++G(2d,p)

Conformation #16

C	4.17104400	-0.19499400	-0.47562700
C	3.78761800	-0.09070900	0.84685200
C	2.42718400	0.09975700	1.08833900
C	1.46919900	0.18996800	0.04847300
C	1.90889100	0.06837200	-1.27635500
C	3.25413000	-0.12157200	-1.53483800
C	0.17869800	0.38405300	0.66142700
C	0.40243100	0.39435900	2.01408300
N	1.74909100	0.22619100	2.27708500
C	-1.13708700	0.54893200	-0.06543200
C	-1.65549800	2.01978400	-0.10635900
C	-2.00574200	2.61209000	1.26373500
C	-0.66974000	2.92917200	-0.84900600
C	-2.18607200	-0.44404100	0.49120800
C	-3.57668500	-0.32742100	-0.12640700
C	-1.73371700	-1.91451000	0.29065500
O	-4.60066200	-0.32469000	0.51394100
O	-1.11973500	-2.25400500	-0.68974200
O	-3.53481700	-0.27571400	-1.46688300
C	-4.80963000	-0.21678200	-2.13420700
C	-2.13068500	-2.88158900	1.37960600
Cl	5.87532600	-0.43460700	-0.85021200
H	4.51374000	-0.15712300	1.64634800
H	1.20650800	0.11054500	-2.09930600
H	3.61103300	-0.22037600	-2.55079700
H	-0.29065300	0.51189300	2.83150600
H	2.16172600	0.19718500	3.19334800
H	-0.96892400	0.25097500	-1.10174100
H	-2.57593000	1.99750800	-0.69913500
H	-1.11759800	2.71231600	1.89196200
H	-2.43151400	3.61079400	1.14014600
H	-2.74359100	2.01287800	1.80241700
H	-1.08138800	3.93632500	-0.95136600
H	-0.45664800	2.55298500	-1.85262400
H	0.27926600	3.00973200	-0.31387400
H	-2.34659000	-0.28550200	1.55722200
H	-4.57880500	-0.19526700	-3.19578100
H	-5.40623500	-1.09416100	-1.88710500
H	-5.35149100	0.68102200	-1.83800100
H	-3.19035100	-2.77083200	1.62478900
H	-1.91411900	-3.90298700	1.07262600
H	-1.56692400	-2.64686500	2.28796900

Compound **1a**, optimization level PCM-B3LYP/6-311++G(2d,p)

Conformation #19

C	-3.88604700	-0.57655200	-0.43224400
C	-3.71335400	0.25552200	0.65553600
C	-2.42107800	0.72233200	0.89184700
C	-1.31037400	0.38575500	0.07069800
C	-1.54732500	-0.46260500	-1.02368800
C	-2.82278300	-0.93988900	-1.26990400
C	-0.14950500	1.04169200	0.63604300
C	-0.60149100	1.71494800	1.73827500
N	-1.95973700	1.54159400	1.89225200
C	1.30490000	1.02592400	0.21628300
C	1.64114500	1.92908700	-1.01093500
C	0.98602700	1.51113700	-2.33240200
C	1.32992500	3.39842900	-0.70302300
C	1.83095800	-0.42381400	0.07358000
C	3.33390000	-0.48602800	-0.16971600
C	1.58354300	-1.23611200	1.37653600
O	4.14230000	0.33041000	0.18524700
O	1.89079000	-0.78061100	2.45028200
O	3.66176700	-1.63182900	-0.80979500
C	5.07090500	-1.83911500	-1.02892200
C	0.96643500	-2.60237600	1.21497700
Cl	-5.49312000	-1.20398100	-0.78236700
H	-4.54688400	0.52510200	1.29086400
H	-0.74734700	-0.74725800	-1.69356000
H	-3.01082000	-1.59217700	-2.11178800
H	-0.04039700	2.30227600	2.44759600
H	-2.51066700	1.90995200	2.64845400
H	1.86785400	1.44669400	1.05270000
H	2.72389800	1.85707000	-1.14014200
H	-0.10020400	1.61463300	-2.29598400
H	1.34851700	2.14765000	-3.14354200
H	1.22117200	0.47978800	-2.60800200
H	1.66942000	4.03911100	-1.52066700
H	1.83456700	3.72873100	0.20863000
H	0.25747500	3.56267300	-0.57329900
H	1.34698900	-0.95045100	-0.74762400
H	5.14820300	-2.79964100	-1.53151300
H	5.47729000	-1.04454000	-1.65376600
H	5.60320400	-1.85601000	-0.07848400
H	-0.04086900	-2.49795900	0.80108200
H	1.54704200	-3.19448800	0.50260700
H	0.91920200	-3.10840100	2.17739000

Compound **1a**, optimization level PCM-B3LYP/6-311++G(2d,p)

Conformation #33

C	4.15235800	0.09182500	0.44223800
C	3.69122000	0.50288900	-0.79373400
C	2.34193200	0.27769400	-1.06340000
C	1.46990500	-0.34244900	-0.13353700
C	1.98632300	-0.74206700	1.10677200
C	3.32310100	-0.52412700	1.39097800
C	0.16774700	-0.41933300	-0.74611800
C	0.29704300	0.14638000	-1.98601600
N	1.59970700	0.56810600	-2.18296700
C	-1.08345500	-0.96479900	-0.09832000
C	-1.95316900	-1.79044300	-1.08706200
C	-1.17427600	-3.03388100	-1.54404100
C	-3.30961200	-2.22281200	-0.51956100
C	-1.78524700	0.19542500	0.68130900
C	-2.41938500	1.25108900	-0.20451900
C	-2.70467300	-0.29798600	1.82604000
O	-3.12762500	1.04793300	-1.16069000
O	-2.36101800	-1.24741200	2.48923200
O	-2.10408300	2.49343300	0.22245800
C	-2.67226800	3.57867000	-0.53351000
C	-3.95702700	0.48330400	2.14347000
Cl	5.84769000	0.34934000	0.84454600
H	4.35361600	0.97425300	-1.50787000
H	1.35312200	-1.22142900	1.84404100
H	3.74046400	-0.82630700	2.34170800
H	-0.43856000	0.28308600	-2.76205600
H	1.95167600	0.98545400	-3.02714000
H	-0.77232500	-1.65131200	0.69443800
H	-2.15533500	-1.16917200	-1.96284700
H	-0.99567800	-3.70968500	-0.70193800
H	-1.74471600	-3.58626100	-2.29466500
H	-0.20725700	-2.77729600	-1.97822900
H	-3.84042300	-2.82842500	-1.25839900
H	-3.94970400	-1.36912100	-0.29346200
H	-3.19533700	-2.82463800	0.38474500
H	-0.99100800	0.70951000	1.23290100
H	-2.31688800	4.48539700	-0.05122600
H	-2.33477000	3.53605000	-1.56845700
H	-3.76079200	3.53034200	-0.51042800
H	-4.46101000	0.03328700	2.99619300
H	-3.69804600	1.52114600	2.37183800
H	-4.62698700	0.50168000	1.28040000

Compound **1a**, optimization level PCM-B3LYP/6-311++G(2d,p)

Conformation #35

C	4.21568500	-0.26937900	-0.38882000
C	3.77756400	-0.16469900	0.91659400
C	2.41481700	0.06947800	1.09959800
C	1.50802900	0.20163900	0.01932300
C	2.00153700	0.07783600	-1.28630700
C	3.34990300	-0.15497900	-1.48679300
C	0.19832600	0.43479000	0.57574300
C	0.36156700	0.42593900	1.93714600
N	1.68865000	0.20787000	2.25846900
C	-1.07582100	0.65329600	-0.20989900
C	-1.55881100	2.13676900	-0.22838600
C	-1.96935400	2.69442700	1.13968500
C	-0.51008300	3.04390000	-0.88259000
C	-2.17296800	-0.33730400	0.24570200
C	-3.44212800	-0.25232100	-0.59369700
C	-1.67731100	-1.80503000	0.11756400
O	-3.50552200	0.09503700	-1.74315700
O	-1.12459000	-2.18107700	-0.88548600
O	-4.51213800	-0.67418900	0.11892800
C	-5.76141000	-0.70745500	-0.59911400
C	-1.94432700	-2.71496700	1.29144100
Cl	5.92601000	-0.56288900	-0.69002100
H	4.46488800	-0.26310300	1.74651300
H	1.33817500	0.15203700	-2.13884800
H	3.74826400	-0.25582400	-2.48701300
H	-0.36266500	0.56271400	2.72398200
H	2.06027600	0.16511000	3.19157800
H	-0.86485000	0.38966900	-1.24789400
H	-2.44120700	2.15321500	-0.87283800
H	-1.11518400	2.76285800	1.81758900
H	-2.37394500	3.70292300	1.02429200
H	-2.74284200	2.09286600	1.62467700
H	-0.89194200	4.06378600	-0.97424200
H	-0.25239300	2.69497700	-1.88535800
H	0.40980400	3.08371800	-0.29424600
H	-2.45691300	-0.16758500	1.28450800
H	-6.49459600	-1.08037100	0.11120700
H	-6.02898100	0.29257300	-0.93854800
H	-5.68530800	-1.37029900	-1.46035900
H	-3.00296800	-2.68194800	1.56141700
H	-1.64524800	-3.73355500	1.05157700
H	-1.37836800	-2.35954100	2.15766600

Compound **1a**, optimization level PCM-B3LYP/6-311++G(2d,p)

Conformation #46

C	-3.91466700	-0.57557000	-0.40451600
C	-3.74645800	0.40431200	0.55207200
C	-2.44197900	0.84349100	0.77604900
C	-1.31937900	0.33518500	0.07108400
C	-1.55018200	-0.66561700	-0.88638800
C	-2.83653000	-1.11456100	-1.12042900
C	-0.15129200	1.02505500	0.57832400
C	-0.61252300	1.88496800	1.54033000
N	-1.98163600	1.78915900	1.66048700
C	1.30796900	0.92398500	0.19324800
C	1.69226400	1.74268300	-1.08246800
C	0.98298300	1.30247300	-2.36786200
C	1.49792800	3.24532200	-0.85093800
C	1.81205900	-0.54348000	0.09957000
C	3.32765700	-0.57807700	-0.04926300
C	1.44585200	-1.42080500	1.32326600
O	4.10670700	0.01416800	0.65917200
O	0.93299400	-2.49974500	1.14283000
O	3.70407200	-1.38130800	-1.05875600
C	5.12547000	-1.52569600	-1.24869000
C	1.76495300	-0.90375000	2.70530500
Cl	-5.53607300	-1.17390000	-0.73755900
H	-4.59049900	0.80519100	1.09778500
H	-0.73566500	-1.10356700	-1.44589700
H	-3.02114500	-1.88855400	-1.85257500
H	-0.05719200	2.57482700	2.15650300
H	-2.54692000	2.30607300	2.31177400
H	1.87628500	1.38344300	1.00698700
H	2.76699000	1.58426500	-1.22600700
H	-0.09479100	1.46449000	-2.30506400
H	1.35529300	1.88367500	-3.21509000
H	1.15555500	0.24974000	-2.60053400
H	1.86833600	3.81343900	-1.70762500
H	2.04163700	3.58705900	0.03392100
H	0.44274900	3.49486400	-0.71749600
H	1.38213100	-1.05801800	-0.75546200
H	5.23627800	-2.20186300	-2.09196600
H	5.57865200	-0.55907200	-1.46593700
H	5.58464000	-1.94585800	-0.35459600
H	2.76203000	-0.46188300	2.73934100
H	1.04778100	-0.12162700	2.96761700
H	1.67761700	-1.71886400	3.42128300

Compound **1a**, optimization level PCM-B3LYP/6-311++G(2d,p)

Conformation #59

C	4.19842100	-0.06166000	-0.51994700
C	3.80732000	-0.40472700	0.75995500
C	2.45354000	-0.26004200	1.06011900
C	1.50625900	0.21352300	0.11693600
C	1.95451000	0.55074200	-1.16781000
C	3.29480000	0.41159000	-1.48261400
C	0.21931300	0.22293700	0.76595100
C	0.43375500	-0.23115100	2.04024400
N	1.77205700	-0.51460700	2.22620000
C	-1.09339200	0.66957500	0.16355500
C	-1.50616900	2.07108200	0.71917300
C	-0.42312800	3.11893700	0.43035400
C	-2.85736900	2.58283000	0.20289200
C	-2.21911800	-0.39033400	0.37488100
C	-3.32271000	-0.27322300	-0.66831000
C	-1.71834800	-1.85928600	0.33728600
O	-3.14827300	-0.05214000	-1.84207800
O	-1.90521200	-2.57007200	1.29743600
O	-4.53156000	-0.51167600	-0.12853400
C	-5.64266800	-0.49246500	-1.04540400
C	-1.03345400	-2.36017400	-0.91074600
Cl	5.89463600	-0.22380800	-0.96306900
H	4.52455100	-0.76745000	1.48443400
H	1.26524000	0.91878500	-1.91826700
H	3.65784500	0.66561300	-2.46905800
H	-0.26886000	-0.39934100	2.84058000
H	2.16613200	-0.91678500	3.05937300
H	-0.95297900	0.76792500	-0.91665400
H	-1.58364200	1.96094900	1.80816200
H	-0.31027900	3.27429500	-0.64702500
H	-0.69816400	4.07908100	0.87313300
H	0.54819400	2.83044800	0.83168200
H	-3.04748400	3.58281900	0.60015400
H	-3.69418400	1.95528000	0.51357100
H	-2.86712700	2.65141400	-0.88772300
H	-2.66815800	-0.27800100	1.36162600
H	-6.51828900	-0.71599600	-0.44206700
H	-5.73340000	0.48955500	-1.50877400
H	-5.50670300	-1.24492300	-1.82139300
H	-0.92782000	-3.44175500	-0.85113600
H	-1.58552200	-2.07470000	-1.80755700
H	-0.04157400	-1.90804200	-0.98798800

Compound **1a**, optimization level PCM-B3LYP/6-311++G(2d,p)

Conformation #99

C	-3.99121900	-0.33773400	-0.38527900
C	-3.71202700	0.28443000	0.81418900
C	-2.37347500	0.59396400	1.05936300
C	-1.32639200	0.30030500	0.14145100
C	-1.67464100	-0.33363900	-1.06309600
C	-2.99689800	-0.65066100	-1.32270800
C	-0.09488500	0.76041700	0.74762000
C	-0.45159100	1.28885200	1.96260300
N	-1.80915500	1.19897900	2.15257200
C	1.33443000	0.72740900	0.25116300
C	1.69323100	1.85508000	-0.76930900
C	0.90764300	1.81311500	-2.08460800
C	1.57498000	3.23772800	-0.11815700
C	1.73730900	-0.68093200	-0.27652600
C	3.19591700	-0.75879100	-0.72166000
C	1.46297500	-1.82793000	0.72271300
O	3.54468700	-1.19323000	-1.79606500
O	0.89669800	-2.82601100	0.32551600
O	4.04575600	-0.31627000	0.21200000
C	5.45289800	-0.36932200	-0.11756900
C	1.90836900	-1.68941900	2.15442800
Cl	-5.66698000	-0.75837200	-0.75953200
H	-4.49120500	0.51871800	1.52724900
H	-0.92838700	-0.58004000	-1.80559600
H	-3.26907700	-1.13921800	-2.24843700
H	0.17499900	1.73117400	2.72103700
H	-2.30240000	1.51055400	2.97364900
H	1.96333400	0.92385900	1.12297300
H	2.75160100	1.71379400	-1.01512700
H	1.27578800	2.59031400	-2.75859600
H	-0.15596800	1.99639700	-1.91933700
H	1.01252300	0.85915500	-2.60550600
H	1.93341000	4.01005400	-0.80283100
H	2.16769200	3.30075200	0.79817200
H	0.53803400	3.47059500	0.13521600
H	1.16411700	-0.93913700	-1.16296000
H	5.75104600	-1.39625200	-0.32137600
H	5.65605400	0.25494700	-0.98610700
H	5.96766200	0.01374000	0.75838200
H	2.92432200	-1.29957100	2.21767000
H	1.83866800	-2.65510800	2.65132300
H	1.25365600	-0.97840600	2.66494400

Compound **1a**, optimization level PCM-M06-2X/6-311++G(2d,p)

Conformation #1

C	4.22463600	-0.02880100	-0.36385600
C	3.76274900	-0.29190200	0.90579400
C	2.38328300	-0.19554500	1.09333000
C	1.49703800	0.15575300	0.05014300
C	2.01847900	0.40993600	-1.22503100
C	3.38019600	0.31666400	-1.42948800
C	0.16777400	0.14802100	0.59928800
C	0.30476200	-0.20500000	1.91388800
N	1.63017600	-0.40556300	2.21612500
C	-1.10108000	0.45670500	-0.14568400
C	-1.56173500	1.93055800	-0.01125300
C	-1.94816600	2.32030700	1.41370000
C	-0.49237800	2.87758100	-0.55095200
C	-2.21670000	-0.52746100	0.26408800
C	-3.45371700	-0.27494900	-0.57634500
C	-1.80064600	-1.99394000	0.09247800
O	-3.47460700	-0.32129800	-1.78104000
O	-2.05860000	-2.78868500	0.96557300
O	-4.51524300	0.01528400	0.17009000
C	-5.72396400	0.28855400	-0.55373400
C	-1.08564700	-2.38756000	-1.16851200
H	4.43602900	-0.56107300	1.70834100
H	1.36801000	0.67802500	-2.04905800
H	3.80796300	0.50723500	-2.40415100
H	-0.44685800	-0.33813800	2.67658700
H	1.98349300	-0.68192700	3.11797900
H	-0.91109900	0.29198500	-1.21162000
H	-2.44831000	2.03880900	-0.64718100
H	-2.25717300	3.36663100	1.43892700
H	-1.09707500	2.20748000	2.08932500
H	-2.77696200	1.72403100	1.79902700
H	-0.21806600	2.62507700	-1.57749000
H	0.40987000	2.83250100	0.06375100
H	-0.85715000	3.90595000	-0.54074800
H	-2.48362300	-0.40765400	1.31255100
H	-6.47252400	0.51118500	0.19922100
H	-6.01238600	-0.58283500	-1.13817000
H	-5.57528900	1.14138600	-1.21349700
H	-0.06991000	-1.98334700	-1.13783900
H	-1.58615800	-1.95856000	-2.03720000
H	-1.04141900	-3.47123800	-1.24047600
Cl	5.94680600	-0.13191300	-0.67089800

Compound **1a**, optimization level PCM-M06-2X/6-311++G(2d,p)

Conformation #4

C	-3.80699000	-0.13340000	-0.03003200
C	-3.22668200	-0.53663000	1.15096500
C	-1.84268200	-0.71821700	1.12896500
C	-1.06412200	-0.49810400	-0.03071200
C	-1.70663000	-0.09235500	-1.20836400
C	-3.07474400	0.08841400	-1.20619600
C	0.30485200	-0.77532200	0.31448900
C	0.29533700	-1.14341500	1.63119900
N	-0.98921200	-1.11408300	2.12144300
C	1.48001300	-0.66695300	-0.62213700
C	2.31438700	-1.96329700	-0.69417600
C	1.45874900	-3.08380700	-1.28474900
C	3.58184100	-1.78235600	-1.52742200
C	2.32469300	0.61021700	-0.36460100
C	1.39056200	1.78644800	-0.15577700
C	3.29275200	0.55629800	0.81325300
O	1.29213500	2.41603500	0.86743900
O	3.20201600	-0.28036000	1.67917000
O	0.65726200	2.01992200	-1.24095500
C	-0.36342800	3.01647300	-1.09574400
C	4.38448000	1.59087000	0.80945200
Cl	-5.54198500	0.11600900	-0.07391800
H	-3.81584200	-0.70202400	2.04298900
H	-1.14002200	0.09137200	-2.11346800
H	-3.59308100	0.40358600	-2.10149600
H	1.11798000	-1.42110100	2.26873600
H	-1.25154000	-1.33235300	3.06907000
H	1.06492000	-0.51647100	-1.62489100
H	2.60115700	-2.24107300	0.32238700
H	0.53969100	-3.22764200	-0.71473500
H	1.18566300	-2.84824400	-2.31769200
H	2.00955400	-4.02582800	-1.28918300
H	4.08664900	-2.74073500	-1.65885400
H	4.29602200	-1.10039100	-1.06120500
H	3.33832100	-1.39344100	-2.52099700
H	2.91074000	0.83057500	-1.26264100
H	0.08426800	3.98050900	-0.86236700
H	-0.87726000	3.05184500	-2.05121300
H	-1.05152800	2.72260900	-0.30387500
H	3.97154100	2.58047900	0.61080600
H	4.91309900	1.58239600	1.75925400
H	5.08226600	1.35840300	0.00017200

Compound **1a**, optimization level PCM-M06-2X/6-311++G(2d,p)

Conformation #6

C	4.15297100	-0.07651500	-0.47110700
C	3.75462800	-0.29659500	0.82783600
C	2.38741800	-0.17955200	1.08256700
C	1.45218400	0.15097300	0.07599500
C	1.90968000	0.36061300	-1.23133300
C	3.25805600	0.24596500	-1.50227300
C	0.15262100	0.17428800	0.69192200
C	0.35354200	-0.14198000	2.00781100
N	1.69078500	-0.34845000	2.24784900
C	-1.14931700	0.47690600	0.00248700
C	-1.58313300	1.96112900	0.11077300
C	-1.92467700	2.38914400	1.53620200
C	-0.51791000	2.87949100	-0.48318200
C	-2.24864300	-0.47624300	0.51329900
C	-3.58866600	-0.17477900	-0.13092500
C	-1.91036900	-1.96039000	0.30669900
O	-4.58220700	0.12557900	0.47915500
O	-2.22999700	-2.75616600	1.15826400
O	-3.53887300	-0.27434600	-1.45878600
C	-4.76048800	0.01889700	-2.15124700
C	-1.19872500	-2.37703300	-0.94977300
H	4.46559200	-0.54847100	1.60312900
H	1.22033200	0.61075300	-2.02899300
H	3.63692400	0.40241200	-2.50290300
H	-0.35821200	-0.24341400	2.81236000
H	2.08649000	-0.60384700	3.13819200
H	-1.01303800	0.27628700	-1.06498100
H	-2.48637300	2.06637600	-0.50219600
H	-2.20737800	3.44310000	1.54789700
H	-1.06022500	2.26830300	2.19341000
H	-2.75835700	1.82015400	1.95124300
H	-0.27238600	2.59459800	-1.50854400
H	0.39934300	2.84216500	0.10931800
H	-0.87173100	3.91166700	-0.49384700
H	-2.40467700	-0.35202500	1.58371200
H	-4.54110300	-0.11722300	-3.20499100
H	-5.06161600	1.04544400	-1.95016700
H	-5.54391500	-0.66401600	-1.82901200
H	-0.17157200	-2.00379400	-0.91724600
H	-1.68085900	-1.94029000	-1.82473800
H	-1.18905200	-3.46178000	-1.01772800
Cl	5.85587700	-0.20668800	-0.86308300

Compound **1a**, optimization level PCM-M06-2X/6-311++G(2d,p)

Conformation #7

C	-3.92463200	-0.30222300	-0.41467300
C	-3.66676300	0.31846600	0.78470600
C	-2.33275300	0.63468100	1.04635600
C	-1.27862700	0.34964200	0.14322000
C	-1.60577500	-0.28113900	-1.06902100
C	-2.91837000	-0.60665300	-1.34206700
C	-0.05919000	0.80833000	0.76856700
C	-0.42990000	1.32638900	1.97748400
N	-1.78735600	1.23525500	2.14678500
C	1.36906100	0.74162800	0.29428300
C	1.74881800	1.80985500	-0.75767500
C	1.02341800	1.65680700	-2.09265000
C	1.53263000	3.21222800	-0.19400800
C	1.71420400	-0.69173800	-0.16678200
C	3.14042500	-0.79972300	-0.66905400
C	1.49618300	-1.67900300	0.99182000
O	3.43901900	-1.25071300	-1.74707800
O	1.99937100	-1.48779100	2.07085600
O	4.02622300	-0.33670500	0.20694100
C	5.39629300	-0.38691500	-0.21198300
C	0.61985900	-2.85919100	0.69465200
H	-4.45762100	0.54589800	1.48702000
H	-0.84891300	-0.51213300	-1.80685500
H	-3.18221800	-1.09271700	-2.27130600
H	0.19134400	1.75772500	2.74707400
H	-2.29469000	1.53893700	2.96223400
H	1.99676400	0.93037100	1.16955000
H	2.82317600	1.69287500	-0.94136400
H	1.38123600	2.41057100	-2.79635300
H	-0.05183000	1.80228400	-1.96842400
H	1.19273600	0.67826100	-2.54744600
H	1.92242500	3.96289400	-0.88359500
H	2.03642800	3.33610500	0.76731700
H	0.46826800	3.40768600	-0.04553300
H	1.08964700	-0.99930800	-1.00270100
H	5.68699900	-1.41540900	-0.41678600
H	5.53282900	0.21787000	-1.10698900
H	5.97132600	0.01697400	0.61468600
H	-0.37429700	-2.49475700	0.41932800
H	0.55591800	-3.51713500	1.55738300
H	1.01322100	-3.39748600	-0.17104900
Cl	-5.57690900	-0.73078100	-0.80814600

Compound **1a**, optimization level PCM-M06-2X/6-311++G(2d,p)

Conformation #8

C	-3.72383800	-0.21840200	-0.13461000
C	-3.16723900	-0.55177100	1.07938200
C	-1.78610900	-0.75336000	1.09155100
C	-0.98663800	-0.62186500	-0.06782500
C	-1.60576500	-0.28765900	-1.28012400
C	-2.97127500	-0.08704500	-1.31112000
C	0.37399300	-0.88464100	0.32049200
C	0.33847900	-1.16261700	1.65704700
N	-0.95370900	-1.08925300	2.12310300
C	1.57581300	-0.79925700	-0.58318900
C	2.57279900	-1.95910900	-0.37372900
C	1.92198300	-3.27127400	-0.81192300
C	3.87781000	-1.74676700	-1.13888800
C	2.23200300	0.60876300	-0.54331200
C	1.14397700	1.63551900	-0.79826400
C	3.03004900	0.94433500	0.71304700
O	0.69661300	1.87430100	-1.89089600
O	3.03498900	0.22894700	1.68555000
O	0.69555600	2.19377300	0.32350600
C	-0.45179500	3.04258700	0.17967100
C	3.86274300	2.19721700	0.63944400
Cl	-5.45346800	0.05520900	-0.22129700
H	-3.77183800	-0.64960100	1.97102100
H	-1.02392200	-0.17216100	-2.18632700
H	-3.47140900	0.17413500	-2.23369800
H	1.14730900	-1.39319300	2.33029900
H	-1.23537200	-1.25332200	3.07609700
H	1.20796500	-0.89204200	-1.61300100
H	2.80460700	-2.02185500	0.69134600
H	1.72715700	-3.25641500	-1.88823600
H	2.58103600	-4.11521500	-0.60157800
H	0.97532700	-3.43971200	-0.29682200
H	4.50294100	-2.63828000	-1.06810600
H	4.45964800	-0.90906800	-0.74904000
H	3.67920800	-1.56131900	-2.19902100
H	2.91622000	0.69998800	-1.39126400
H	-0.67577200	3.40540600	1.17748600
H	-0.22526500	3.87039000	-0.48943800
H	-1.28411600	2.46049700	-0.21622000
H	3.28653400	3.02819900	0.23081800
H	4.24551800	2.45046900	1.62483900
H	4.69992300	2.01522700	-0.03998500

Compound **1a**, optimization level PCM-M06-2X/6-311++G(2d,p)

Conformation #9

C	-4.34691600	-0.25555600	-0.30542600
C	-3.89224700	0.63356000	0.64244500
C	-2.50894300	0.69805700	0.81519700
C	-1.61456300	-0.09871400	0.06595900
C	-2.12762200	-0.98698400	-0.88725000
C	-3.49356100	-1.06367800	-1.07133700
C	-0.28377800	0.23500200	0.49611300
C	-0.42612700	1.19225700	1.46131400
N	-1.75893100	1.47219400	1.65800900
C	0.97508900	-0.40166800	-0.02243200
C	1.17597000	-1.80043700	0.62327200
C	2.13945300	-2.70235900	-0.15029700
C	1.58342600	-1.72203800	2.09328900
C	2.17143500	0.55365100	0.15985800
C	3.41484100	0.02401400	-0.52856300
C	1.90145600	1.95160500	-0.42183000
O	3.48780400	-0.21414100	-1.70773700
O	2.22466600	2.92692500	0.21423500
O	4.42835100	-0.13142200	0.32021500
C	5.64328400	-0.63179200	-0.25583500
C	1.25961700	2.04909000	-1.77648600
Cl	-6.07359700	-0.38761800	-0.57366200
H	-4.57468700	1.24563100	1.21653600
H	-1.46723100	-1.61164900	-1.47757800
H	-3.91766800	-1.74059200	-1.80017100
H	0.32369200	1.71462400	2.03504700
H	-2.11755600	2.15320900	2.30750800
H	0.85128000	-0.56245600	-1.09957700
H	0.18865500	-2.27070200	0.57447800
H	3.18090700	-2.40649800	-0.00718100
H	2.04941700	-3.72779900	0.21268400
H	1.92531500	-2.69790000	-1.22052500
H	1.53658200	-2.71337600	2.54680700
H	0.92686800	-1.05890000	2.65971300
H	2.61073800	-1.36290400	2.19589400
H	2.39803300	0.69967600	1.21525300
H	6.35060900	-0.70003900	0.56386200
H	5.46801100	-1.61246400	-0.69603800
H	6.00275000	0.05361700	-1.02070000
H	1.76159400	1.38317200	-2.47883900
H	0.21836300	1.72607900	-1.69900600
H	1.29654000	3.07846500	-2.12363600

Compound **1a**, optimization level PCM-M06-2X/6-311++G(2d,p)

Conformation #16

C	-4.18214500	0.04723900	-0.47932700
C	-3.77998900	0.40292000	0.78793800
C	-2.41218100	0.31003200	1.05054000
C	-1.48010200	-0.12606300	0.08180300
C	-1.94183800	-0.47304000	-1.19404900
C	-3.29079000	-0.38582300	-1.47250600
C	-0.17965900	-0.09392900	0.69347000
C	-0.37571300	0.35418100	1.97069900
N	-1.71265800	0.59485500	2.19074700
C	1.11403300	-0.48644300	0.03328000
C	1.55343000	-1.93820100	0.34566900
C	1.92571100	-2.15695200	1.81061300
C	0.47809300	-2.93193400	-0.08707000
C	2.20804800	0.53834900	0.37475900
C	3.56166300	0.12306300	-0.17506300
C	1.87177100	1.93248800	-0.17488300
O	4.54399800	-0.06206800	0.49730100
O	1.16839800	2.07243700	-1.14382400
O	3.53253800	-0.01862500	-1.49780900
C	4.75827100	-0.45059700	-2.10401400
C	2.47836800	3.08742600	0.56839400
H	-4.48862100	0.73623100	1.53405200
H	-1.25413400	-0.80688600	-1.96177400
H	-3.67283600	-0.64691600	-2.44984500
H	0.33635900	0.52850300	2.76250200
H	-2.10563300	0.94008600	3.05139800
H	0.96051000	-0.42429100	-1.04729900
H	2.44389200	-2.13237100	-0.26412700
H	2.23546600	-3.19209100	1.96467500
H	1.06778100	-1.96600800	2.45951700
H	2.74999000	-1.51530600	2.12776700
H	0.20647400	-2.79290700	-1.13582000
H	-0.42482300	-2.81034800	0.51619200
H	0.83399100	-3.95582900	0.03912000
H	2.34917600	0.62461100	1.45265700
H	4.55587500	-0.50753300	-3.16831800
H	5.04016500	-1.42693200	-1.71330400
H	5.54873300	0.26888700	-1.89994700
H	3.53956700	2.90378600	0.75078100
H	1.99425600	3.15891400	1.54634800
H	2.33817700	4.01396700	0.01799100
Cl	-5.88629800	0.14048200	-0.87909800

Compound **1a**, optimization level PCM-M06-2X/6-311++G(2d,p)

Conformation #19

C	-3.83820400	-0.57980400	-0.41187300
C	-3.68079800	0.29818400	0.63440300
C	-2.38731600	0.77275700	0.85778900
C	-1.27712700	0.39390700	0.06319000
C	-1.50170900	-0.50265900	-0.99471800
C	-2.77239600	-0.98722900	-1.22673000
C	-0.12261600	1.06922400	0.60939400
C	-0.58229100	1.79556400	1.67166800
N	-1.93560700	1.63156900	1.82068100
C	1.32516200	1.02280500	0.19216500
C	1.65274000	1.87029500	-1.06082100
C	0.96614700	1.39511800	-2.33953100
C	1.32583500	3.33998600	-0.80454600
C	1.81154200	-0.43293900	0.07377000
C	3.30898300	-0.52280900	-0.14568100
C	1.53512800	-1.18958400	1.38875300
O	4.11644100	0.27665700	0.24911600
O	1.97960300	-0.77149500	2.42932500
O	3.63262300	-1.64608900	-0.78890500
C	5.03669400	-1.86775800	-0.97937900
C	0.71421000	-2.43929000	1.28953200
H	-4.51630300	0.60022800	1.25166500
H	-0.69588700	-0.82123200	-1.64276800
H	-2.95793600	-1.67899300	-2.03688600
H	-0.02852300	2.42710000	2.34901600
H	-2.49795300	2.05298700	2.54223400
H	1.90055300	1.45581300	1.01539200
H	2.73373700	1.79365400	-1.20722600
H	1.29625100	2.00492900	-3.18259600
H	-0.11838900	1.49638600	-2.26131200
H	1.20090700	0.35561200	-2.57924900
H	1.67081900	3.95621600	-1.63668200
H	1.80521600	3.69998900	0.10866300
H	0.24798900	3.48406800	-0.70019000
H	1.32011200	-0.96531500	-0.73911600
H	5.46301600	-1.06192000	-1.57371600
H	5.53941100	-1.91827400	-0.01538700
H	5.11664500	-2.81408600	-1.50398000
H	-0.26895600	-2.18620200	0.88369000
H	0.61293400	-2.90905600	2.26443200
H	1.18676800	-3.12398200	0.58094300
Cl	-5.43378900	-1.22094700	-0.74601700

Compound **1a**, optimization level PCM-M06-2X/6-311++G(2d,p)

Conformation #33

C	4.01416800	-0.06670700	0.46311300
C	3.60317800	0.21311900	-0.82094100
C	2.25190800	0.00020100	-1.09697000
C	1.34692200	-0.48198300	-0.12456100
C	1.81545000	-0.75073700	1.16757200
C	3.14863900	-0.54216800	1.45930700
C	0.05895300	-0.58726600	-0.75373600
C	0.23219100	-0.16849200	-2.04235700
N	1.54630600	0.18336100	-2.25444600
C	-1.21874300	-0.97466300	-0.05967200
C	-2.26136900	-1.56514200	-1.02996700
C	-1.73972200	-2.88311100	-1.60895800
C	-3.62329400	-1.81137700	-0.38283700
C	-1.68836500	0.25218800	0.77787500
C	-2.12736800	1.38475500	-0.12232800
C	-2.67964400	-0.09256600	1.89221000
O	-3.13042500	1.38413100	-0.79230000
O	-2.52394200	-1.11013800	2.52426500
O	-1.25227900	2.39013200	-0.11866000
C	-1.55203100	3.47294300	-1.00813900
C	-3.75210900	0.90413200	2.22979600
Cl	5.69889700	0.18102200	0.87878400
H	4.29426800	0.57795400	-1.56883800
H	1.14652900	-1.12166700	1.93587900
H	3.53772200	-0.74201300	2.44822600
H	-0.48027900	-0.09483000	-2.84895900
H	1.92657600	0.50175700	-3.13089000
H	-0.98940000	-1.74493800	0.68485300
H	-2.40875200	-0.85561400	-1.84959300
H	-1.61997400	-3.62097800	-0.81051500
H	-2.45101400	-3.28597800	-2.33185700
H	-0.77812500	-2.76293800	-2.10689500
H	-4.29250700	-2.27893300	-1.10746200
H	-4.09583600	-0.88473400	-0.05572700
H	-3.53100900	-2.48072900	0.47528000
H	-0.81024400	0.60791400	1.32728600
H	-0.74721700	4.18956200	-0.88170900
H	-1.58614800	3.11008500	-2.03416200
H	-2.50931600	3.91919500	-0.74543100
H	-4.27142200	0.59717500	3.13366100
H	-3.30493500	1.89209400	2.36800300
H	-4.45373000	0.98495100	1.39776600

Compound **1a**, optimization level PCM-M06-2X/6-311++G(2d,p)

Conformation #35

C	-4.25480700	0.03983400	-0.35006100
C	-3.78303500	0.31106500	0.91412000
C	-2.40181600	0.21525800	1.09136200
C	-1.52381700	-0.14141700	0.04282200
C	-2.05562800	-0.40345800	-1.22610100
C	-3.41899100	-0.31265500	-1.42038400
C	-0.19082900	-0.13692900	0.58040100
C	-0.31629800	0.21792400	1.89513000
N	-1.64001800	0.42773300	2.20719800
C	1.06742300	-0.46284800	-0.17657400
C	1.54185000	-1.92465300	0.01252700
C	1.95357500	-2.25133400	1.44653000
C	0.47162500	-2.90141100	-0.46962000
C	2.16761000	0.55820500	0.16247000
C	3.43763500	0.22470600	-0.60099000
C	1.75630800	1.98144400	-0.23491000
O	3.52278300	0.24238400	-1.80232400
O	1.00247700	2.18024700	-1.15437400
O	4.44019800	-0.10990000	0.20752100
C	5.66128200	-0.48601900	-0.44586200
C	2.35988600	3.08884600	0.57991800
H	-4.45020000	0.58452600	1.72038200
H	-1.41079800	-0.67466600	-2.05333800
H	-3.85468400	-0.50907500	-2.39039700
H	0.43931600	0.34329300	2.65531600
H	-1.98618500	0.70122900	3.11250600
H	0.85429600	-0.33097400	-1.24113300
H	2.41942000	-2.05538800	-0.63186400
H	2.30453400	-3.28314700	1.50547700
H	1.10319300	-2.14967000	2.12458700
H	2.75981100	-1.60795600	1.80340000
H	0.17955800	-2.69359100	-1.50131900
H	-0.42144900	-2.83469600	0.15650200
H	0.84256800	-3.92661900	-0.42114800
H	2.40481700	0.54930100	1.22664600
H	6.35831600	-0.72561800	0.35016700
H	6.03186200	0.34171600	-1.04725100
H	5.48843900	-1.35328400	-1.08068300
H	3.43795600	2.94127300	0.67769900
H	1.93912800	3.04466400	1.58827300
H	2.14542800	4.05520100	0.13122200
Cl	-5.98004100	0.14048000	-0.64392300

Compound **1a**, optimization level PCM-M06-2X/6-311++G(2d,p)

Conformation #46

C	-3.86201600	-0.60063500	-0.36970700
C	-3.70836900	0.46515600	0.48476500
C	-2.40495300	0.92873900	0.67011300
C	-1.28186500	0.35514900	0.02567400
C	-1.50215500	-0.72934100	-0.83893500
C	-2.78306500	-1.20314200	-1.03229800
C	-0.12351200	1.09414400	0.47121200
C	-0.59354500	2.04337700	1.33564100
N	-1.95625300	1.95549600	1.45419100
C	1.33224200	0.92784600	0.12480500
C	1.73792900	1.55587000	-1.23210900
C	1.06252100	0.91212600	-2.44105000
C	1.47986400	3.06066500	-1.22143400
C	1.76887100	-0.55247900	0.22018500
C	3.26891800	-0.65340400	0.01748800
C	1.39421000	-1.18009200	1.56792900
O	4.08750600	-0.04472200	0.65987600
O	0.78123800	-2.22129600	1.58561200
O	3.57991300	-1.49260000	-0.96672000
C	4.98200400	-1.63816200	-1.23528800
C	1.79437700	-0.46264800	2.82596700
H	-4.55376000	0.91737700	0.98555600
H	-0.68604800	-1.20119800	-1.36918900
H	-2.96663000	-2.03760400	-1.69510800
H	-0.04215000	2.78990000	1.88675600
H	-2.52927500	2.53961800	2.04167500
H	1.90400000	1.46717600	0.88718300
H	2.82017000	1.41006100	-1.32937000
H	1.42048700	1.38419100	-3.35770600
H	-0.02029300	1.04803100	-2.39716500
H	1.27551200	-0.15583300	-2.51935500
H	1.88330200	3.52042300	-2.12511000
H	1.94681300	3.53835600	-0.35716400
H	0.40801300	3.26755100	-1.18517800
H	1.28521200	-1.16155300	-0.53894100
H	5.40591700	-0.67718800	-1.52071200
H	5.49231800	-2.01726000	-0.35214000
H	5.05169200	-2.34700000	-2.05355500
H	2.81835300	-0.09715800	2.75207400
H	1.67641800	-1.12776300	3.67769100
H	1.14094900	0.40418800	2.95236200
Cl	-5.47030600	-1.23663800	-0.64881300

Compound **1a**, optimization level PCM-M06-2X/6-311++G(2d,p)

Conformation #59

C	4.20559600	0.00577800	-0.46540000
C	3.79935600	-0.51429600	0.74266200
C	2.43651700	-0.42112700	1.02836100
C	1.51349900	0.17215300	0.13730500
C	1.97853700	0.68692800	-1.07952900
C	3.32329900	0.60205100	-1.37881600
C	0.21651900	0.08855800	0.74967300
C	0.40435500	-0.53384600	1.95203100
N	1.73420700	-0.83964500	2.12533900
C	-1.07878500	0.60669800	0.18204500
C	-1.45418400	1.95988900	0.83850100
C	-0.35472300	2.99218600	0.58791400
C	-2.79581100	2.52343700	0.36983500
C	-2.20218000	-0.44260800	0.33938200
C	-3.36479500	-0.18668300	-0.60439900
C	-1.72819900	-1.87486400	0.04011700
O	-3.25014200	0.16352600	-1.75107600
O	-1.96630800	-2.75987900	0.82680900
O	-4.53500600	-0.44943200	-0.02610300
C	-5.68952700	-0.25508700	-0.85470100
C	-1.00195300	-2.11894800	-1.25351200
Cl	5.90396700	-0.07917700	-0.88899700
H	4.50189000	-0.96929400	1.42778100
H	1.29741500	1.14997700	-1.78387200
H	3.70886000	0.99095300	-2.31125400
H	-0.31389800	-0.79725800	2.71323800
H	2.12048100	-1.31621000	2.92425500
H	-0.94074100	0.77718000	-0.89178700
H	-1.51708900	1.77629600	1.91753000
H	-0.24692300	3.17566700	-0.48546400
H	-0.60955000	3.93958000	1.06534500
H	0.61001100	2.66386600	0.97485900
H	-2.95563700	3.50495200	0.81910400
H	-3.64129900	1.89817400	0.66282600
H	-2.81114800	2.64184000	-0.71619800
H	-2.58400700	-0.45280600	1.36124000
H	-6.54232500	-0.51248900	-0.23546400
H	-5.74430500	0.78516600	-1.17199700
H	-5.63682600	-0.90297300	-1.72737800
H	-0.95223100	-3.18759600	-1.44690100
H	-1.49430400	-1.59820700	-2.07520900
H	0.01125300	-1.71833000	-1.17095400

Compound **1a**, optimization level PCM-M06-2X/6-311++G(2d,p)

Conformation #99

C	-3.94716200	-0.34346300	-0.39927400
C	-3.68580100	0.29889500	0.78758600
C	-2.35004000	0.61487200	1.04100200
C	-1.29806900	0.30602100	0.14387500
C	-1.62846300	-0.34715400	-1.05527300
C	-2.94280400	-0.67030100	-1.32130100
C	-0.07760200	0.78057100	0.75503500
C	-0.44630400	1.33132900	1.95077300
N	-1.80250400	1.24213600	2.12581900
C	1.34714100	0.72081900	0.27026000
C	1.71045400	1.80788200	-0.77181400
C	0.96423900	1.67028900	-2.09703700
C	1.49985600	3.20196500	-0.18635300
C	1.69912500	-0.69716300	-0.23391000
C	3.13310200	-0.77088900	-0.72557800
C	1.44240000	-1.78230800	0.82072700
O	3.44590500	-1.13675900	-1.82958900
O	0.85204000	-2.78562900	0.49606300
O	4.01035000	-0.37532100	0.19526800
C	5.38667300	-0.39052200	-0.20922100
C	1.91702200	-1.55879800	2.22946100
H	-4.47487500	0.54498400	1.48554400
H	-0.87395000	-0.59542300	-1.78939800
H	-3.20980000	-1.17279900	-2.24084700
H	0.17518900	1.78787500	2.70601200
H	-2.30840800	1.56776000	2.93369300
H	1.98352100	0.91705900	1.13889100
H	2.78256400	1.69670000	-0.97315100
H	1.29786000	2.44459000	-2.79013400
H	-0.11078900	1.79558500	-1.95036700
H	1.14034900	0.70484500	-2.57584800
H	1.88163300	3.96138300	-0.87067900
H	2.01485800	3.31414800	0.77050300
H	0.43731800	3.39530200	-0.02351100
H	1.08945500	-0.97369400	-1.08996000
H	5.68077500	-1.39923100	-0.49190700
H	5.53134400	0.28431800	-1.05107000
H	5.95027400	-0.05303100	0.65421400
H	2.92980500	-1.15809800	2.24286800
H	1.86244300	-2.49359100	2.78155300
H	1.26213100	-0.82246800	2.70191700
Cl	-5.60194900	-0.77010300	-0.78451400

Compound **1a**, optimization level PCM-M11/6-311++G(2d,p)

Conformation #1

C	4.21528000	-0.00962900	-0.36466000
C	3.75842100	-0.26961200	0.90439900
C	2.37862000	-0.18230400	1.09364600
C	1.49369200	0.15750500	0.04986400
C	2.00960000	0.40858100	-1.22669800
C	3.36913900	0.32449500	-1.43176400
C	0.16300000	0.14335600	0.59784200
C	0.29973700	-0.20343900	1.91229400
N	1.62578800	-0.39335800	2.21691400
C	-1.10828200	0.43683300	-0.15060800
C	-1.58368300	1.90684300	-0.01928100
C	-2.00172400	2.27527100	1.40417900
C	-0.50059600	2.86152600	-0.52150300
C	-2.21517300	-0.55430300	0.27153800
C	-3.45083000	-0.29855900	-0.57041800
C	-1.77138600	-2.01197400	0.09835700
O	-3.47787800	-0.37958600	-1.77575700
O	-1.98439900	-2.80782700	0.98510700
O	-4.50085300	0.05680500	0.16425400
C	-5.70316600	0.35422300	-0.57272500
C	-1.07005800	-2.38878600	-1.17536200
H	4.43897300	-0.53146300	1.70873500
H	1.35140500	0.66794300	-2.05347100
H	3.79813400	0.51362300	-2.41094300
H	-0.45892900	-0.34014800	2.67405800
H	1.98065200	-0.66161200	3.12242400
H	-0.91898400	0.26512400	-1.22018000
H	-2.45909800	2.01534500	-0.67724500
H	-2.29036600	3.33112900	1.44694200
H	-1.16617500	2.12918300	2.09988800
H	-2.85567100	1.68516600	1.75471200
H	-0.18925100	2.61250200	-1.54295400
H	0.38350200	2.81329200	0.12620400
H	-0.86815300	3.89330200	-0.51853700
H	-2.48002300	-0.43061800	1.32404000
H	-6.44826200	0.62992200	0.17213600
H	-6.01935700	-0.52889100	-1.13276700
H	-5.51523500	1.18161400	-1.26140800
H	-0.05339600	-1.97585600	-1.14661300
H	-1.58476500	-1.94863500	-2.03475000
H	-1.01708800	-3.47481200	-1.26121700
Cl	5.94123900	-0.10281700	-0.67475800

Compound **1a**, optimization level PCM-M11/6-311++G(2d,p)

Conformation #4

```
C                  -3.80687400   -0.13512300   -0.02948900
 C                  -3.22631300   -0.51903200    1.15428600
 C                  -1.84165100   -0.69632400    1.13427100
 C                  -1.06871000   -0.48831800   -0.02750100
 C                  -1.71211700   -0.10360300   -1.21046400
 C                  -3.07808000    0.07222500   -1.20987500
 C                   0.30303500   -0.75852400    0.31808400
 C                   0.29752500   -1.11198500    1.63722700
 N                  -0.98527000   -1.07818100    2.13017400
 C                   1.47746000   -0.66836900   -0.62323900
 C                   2.30639000   -1.97053700   -0.66960200
 C                   1.44461200   -3.09224100   -1.25322100
 C                   3.58053500   -1.80203000   -1.49846200
 C                   2.33250600    0.60491500   -0.38437700
 C                   1.40321200    1.78503200   -0.17300600
 C                   3.30476900    0.55494100    0.79106700
 O                   1.33012000    2.43391400    0.84338400
 O                   3.20597500   -0.27182900    1.66775600
 O                   0.63559300    1.99883500   -1.23803500
 C                  -0.38704500    2.99960000   -1.07672600
 C                   4.40503000    1.57999800    0.76833800
 Cl                 -5.54641400    0.10877300   -0.07633100
 H                  -3.81847500   -0.67457900    2.05098700
 H                  -1.14251900    0.06816500   -2.12148700
 H                  -3.60213500    0.37290800   -2.11204600
 H                   1.12876300   -1.38353500    2.27384000
 H                  -1.24550500   -1.28735000    3.08213900
 H                   1.06074100   -0.53115700   -1.63186200
 H                   2.58299100   -2.23425800    0.35747900
 H                   0.51635300   -3.21832800   -0.68554800
 H                   1.18011100   -2.86446100   -2.29478200
 H                   1.98669200   -4.04416700   -1.24368500
 H                   4.08225400   -2.76712300   -1.62671700
 H                   4.30006400   -1.11979300   -1.03001200
 H                   3.34141800   -1.41282900   -2.49789800
 H                   2.91607700    0.81738100   -1.29018000
 H                   0.07173100    3.97185800   -0.88191900
 H                  -0.94289300    3.01144800   -2.01393600
 H                  -1.03967400    2.71859000   -0.24518200
 H                   3.99095100    2.57335700    0.56904000
 H                   4.94953700    1.57369000    1.71338900
 H                   5.09022900    1.33719900   -0.05389500
```

Compound **1a**, optimization level PCM-M11/6-311++G(2d,p)

Conformation #6

C	4.14331300	-0.07959600	-0.47101700
C	3.74928400	-0.26754900	0.83128300
C	2.38244800	-0.14723100	1.08606200
C	1.44956300	0.15542900	0.07306500
C	1.90217500	0.33262800	-1.23938700
C	3.24783200	0.21464700	-1.50924200
C	0.14877000	0.19125600	0.68802000
C	0.34862900	-0.09197800	2.00976000
N	1.68591000	-0.28925400	2.25536300
C	-1.15462600	0.47124100	-0.01007500
C	-1.59680200	1.95536700	0.07015800
C	-1.96631000	2.38937900	1.48864400
C	-0.51318300	2.86714500	-0.50462900
C	-2.24878300	-0.47361200	0.52864900
C	-3.58377700	-0.18376700	-0.13264100
C	-1.88869300	-1.95645400	0.35129600
O	-4.58071900	0.14877300	0.45966300
O	-2.17275200	-2.73919300	1.22979400
O	-3.52837400	-0.31849600	-1.45764300
C	-4.74919500	-0.03241400	-2.16706400
C	-1.18613200	-2.38240300	-0.90722800
H	4.46655500	-0.49943700	1.61262500
H	1.20606200	0.56107700	-2.04399400
H	3.62794700	0.34701200	-2.51753800
H	-0.36989400	-0.17550100	2.81665600
H	2.08255900	-0.52111600	3.15353800
H	-1.01928000	0.24421000	-1.07688700
H	-2.48994900	2.05275400	-0.56552800
H	-2.22598600	3.45351800	1.49849500
H	-1.11645400	2.24680400	2.16777500
H	-2.82581000	1.83530900	1.88216300
H	-0.23316000	2.56577100	-1.52103900
H	0.38744400	2.83446300	0.12082500
H	-0.86474400	3.90386800	-0.54053600
H	-2.40583100	-0.32319900	1.59928000
H	-4.52797900	-0.19741600	-3.22066400
H	-5.04171000	1.00523800	-1.98807500
H	-5.53989100	-0.70399000	-1.82457100
H	-0.15814300	-1.99930900	-0.88223800
H	-1.67940500	-1.95305300	-1.78426400
H	-1.16764600	-3.47130600	-0.96579000
Cl	5.84950800	-0.21659500	-0.86330600

Compound **1a**, optimization level PCM-M11/6-311++G(2d,p)

Conformation #7

C	-3.91695300	-0.29752400	-0.41894000
C	-3.66308100	0.32227900	0.77828700
C	-2.32961200	0.63650100	1.04456900
C	-1.27677500	0.34791100	0.14576000
C	-1.60000600	-0.28251200	-1.06685900
C	-2.90983600	-0.60456300	-1.34348800
C	-0.05642500	0.80395600	0.77477100
C	-0.42805400	1.32373400	1.98093200
N	-1.78562900	1.23612600	2.14643200
C	1.37341400	0.73153600	0.30292100
C	1.75881000	1.80246400	-0.74489000
C	1.04456500	1.63532600	-2.08590100
C	1.51251600	3.20230600	-0.18255900
C	1.70745700	-0.70260800	-0.16769800
C	3.13223400	-0.79135200	-0.67914800
C	1.49227300	-1.68279200	0.99761200
O	3.42949500	-1.18503600	-1.78249500
O	2.00779600	-1.48666400	2.07201900
O	4.02347100	-0.36626800	0.21075700
C	5.39638800	-0.39250000	-0.22109300
C	0.59349100	-2.85083000	0.71806500
H	-4.46033500	0.55116500	1.47904000
H	-0.83795500	-0.51499400	-1.80543300
H	-3.17447300	-1.09206200	-2.27668500
H	0.19759000	1.75619000	2.75265600
H	-2.29521300	1.54453200	2.96072200
H	2.00531200	0.90827100	1.18285000
H	2.84035400	1.69670500	-0.91592000
H	1.39381700	2.39578300	-2.79308900
H	-0.03725800	1.76585500	-1.96445400
H	1.23259000	0.65395200	-2.53847500
H	1.90930800	3.96492700	-0.86151300
H	1.99083500	3.32925800	0.79630100
H	0.43747400	3.38077000	-0.05969300
H	1.07421100	-1.00847500	-1.00258000
H	5.68418300	-1.41500100	-0.47702000
H	5.51979100	0.25439600	-1.09359200
H	5.98122100	-0.02379100	0.62057500
H	-0.39911500	-2.46685600	0.44899800
H	0.52503600	-3.50385400	1.58884400
H	0.96969900	-3.40324800	-0.15084800
Cl	-5.57213000	-0.72533200	-0.81835300

Compound **1a**, optimization level PCM-M11/6-311++G(2d,p)

Conformation #8

C	-3.70486400	-0.24023300	-0.13304100
C	-3.14573300	-0.54505000	1.08397400
C	-1.76495500	-0.74966100	1.09557100
C	-0.97426000	-0.64387300	-0.06853700
C	-1.59690800	-0.34384500	-1.28665200
C	-2.95948100	-0.14224200	-1.31672600
C	0.39042200	-0.89240000	0.31959100
C	0.36251000	-1.14255700	1.66005200
N	-0.92727900	-1.06315200	2.13036500
C	1.59133200	-0.80064500	-0.58601500
C	2.61431000	-1.93269600	-0.34684200
C	1.99404000	-3.26662200	-0.76888900
C	3.91862400	-1.69702600	-1.10981900
C	2.21691800	0.62212600	-0.56457900
C	1.09656600	1.61741000	-0.80858400
C	3.00840700	0.98682600	0.68871100
O	0.61421400	1.82788700	-1.89472700
O	3.03248100	0.27504700	1.66553400
O	0.65405200	2.17944700	0.31443800
C	-0.52766800	2.99280500	0.18333500
C	3.80392300	2.26323600	0.60532200
Cl	-5.43753400	0.03851800	-0.21940800
H	-3.75095700	-0.61982700	1.98230200
H	-1.01464400	-0.25365700	-2.20058200
H	-3.46719800	0.09543600	-2.24656500
H	1.18235800	-1.35369500	2.33354700
H	-1.20404900	-1.20608600	3.08987600
H	1.22698300	-0.92018000	-1.61885300
H	2.83822000	-1.96895200	0.72496000
H	1.80206800	-3.27021200	-1.85028100
H	2.67155300	-4.09774600	-0.54452600
H	1.04497400	-3.44632100	-0.25275600
H	4.56731700	-2.57612500	-1.03214400
H	4.48134700	-0.83927400	-0.72288900
H	3.71691400	-1.52276100	-2.17587000
H	2.89579500	0.72552100	-1.41973000
H	-0.73564200	3.37961900	1.18017700
H	-0.34247300	3.80785900	-0.52024300
H	-1.35274200	2.36888600	-0.17439600
H	3.19029700	3.07854400	0.20947500
H	4.19931300	2.52637100	1.58720000
H	4.63452200	2.10749000	-0.09450300

Compound **1a**, optimization level PCM-M11/6-311++G(2d,p)

Conformation #9

C	-4.34344300	-0.27326900	-0.28865400
C	-3.89090300	0.64338800	0.62934800
C	-2.50725700	0.72252400	0.79220500
C	-1.61650600	-0.08776600	0.05937500
C	-2.12715900	-1.00613100	-0.86432700
C	-3.49099800	-1.09752000	-1.03707400
C	-0.28371100	0.26503500	0.47044300
C	-0.42325300	1.24538200	1.41026100
N	-1.75580900	1.52364800	1.60857500
C	0.97637400	-0.38002100	-0.03712400
C	1.16873200	-1.77356500	0.62418800
C	2.12319200	-2.69264900	-0.14470500
C	1.59215700	-1.66656200	2.08976300
C	2.17323400	0.57306500	0.15838900
C	3.41875500	0.01675100	-0.50521900
C	1.90629300	1.95932000	-0.45184800
O	3.50427000	-0.23419600	-1.68348000
O	2.19698900	2.95037100	0.17923900
O	4.41663600	-0.16428300	0.35731800
C	5.62640600	-0.71049100	-0.20281200
C	1.29232500	2.02151800	-1.82108200
Cl	-6.07448800	-0.42390500	-0.54351900
H	-4.57862200	1.26788400	1.19112900
H	-1.46082100	-1.64400400	-1.44210000
H	-3.91830200	-1.79989200	-1.74610700
H	0.33455900	1.78720100	1.96400800
H	-2.11445600	2.21885500	2.24544600
H	0.86663000	-0.54591100	-1.11947500
H	0.17360300	-2.23743400	0.59144400
H	3.17227800	-2.41434700	0.00935700
H	2.01309000	-3.72162300	0.21494400
H	1.91865800	-2.68019100	-1.22115700
H	1.52788800	-2.64584300	2.57594300
H	0.95427400	-0.96768800	2.64346400
H	2.63282100	-1.32658200	2.16989500
H	2.38410100	0.73126900	1.21923800
H	6.32508900	-0.80194200	0.62755700
H	5.41606400	-1.68828600	-0.64541900
H	6.01536900	-0.03606400	-0.96919000
H	1.79459500	1.31725500	-2.49080800
H	0.24074400	1.71855200	-1.74398500
H	1.35081100	3.04043900	-2.20575500

Compound **1a**, optimization level PCM-M11/6-311++G(2d,p)

Conformation #16

C	-4.17370800	0.02478500	-0.48143600
C	-3.77308500	0.43784800	0.76561200
C	-2.40544300	0.35744900	1.03290000
C	-1.47810300	-0.12325300	0.08563900
C	-1.93783500	-0.52766800	-1.17263500
C	-3.28412400	-0.45309000	-1.45458000
C	-0.17580900	-0.06488600	0.69379900
C	-0.36821700	0.44236600	1.94741200
N	-1.70422000	0.69501000	2.15790700
C	1.11812800	-0.48482500	0.04920900
C	1.55511000	-1.92373400	0.42137000
C	1.94011300	-2.06652800	1.89419500
C	0.46122600	-2.92616800	0.05414200
C	2.21290900	0.54738800	0.36212100
C	3.55929600	0.11358800	-0.19268300
C	1.87060700	1.92292900	-0.23055700
O	4.55773200	-0.03752600	0.46894600
O	1.18236000	2.02264700	-1.21739600
O	3.50951800	-0.09101500	-1.50681200
C	4.73487300	-0.53924300	-2.11617200
C	2.44751300	3.10462200	0.49384900
H	-4.48633500	0.80606100	1.49658700
H	-1.24545100	-0.89724600	-1.92601900
H	-3.66922800	-0.75903300	-2.42246900
H	0.35189400	0.65234700	2.72972100
H	-2.09656000	1.07962800	3.00396300
H	0.96915800	-0.45764400	-1.03766200
H	2.43993200	-2.15347400	-0.19116200
H	2.22146800	-3.10299300	2.11053600
H	1.09090800	-1.80991600	2.53957500
H	2.78988200	-1.42934600	2.16501400
H	0.16860000	-2.83093300	-0.99839100
H	-0.43091600	-2.76254100	0.67132600
H	0.80760500	-3.95174700	0.22191500
H	2.36153800	0.66420200	1.43995300
H	4.51744200	-0.65072900	-3.17750900
H	5.03426900	-1.49461000	-1.67754800
H	5.51990500	0.20336900	-1.95484000
H	3.50858100	2.93262300	0.70881500
H	1.93532200	3.19610300	1.46015700
H	2.31250500	4.01855400	-0.08552900
Cl	-5.88168100	0.09881900	-0.88545900

Compound **1a**, optimization level PCM-M11/6-311++G(2d,p)

Conformation #19

C	-3.82364200	-0.59006800	-0.39586900
C	-3.67144200	0.30109800	0.63603100
C	-2.38073300	0.78590100	0.85282300
C	-1.27224500	0.39951500	0.06472000
C	-1.49221700	-0.50911900	-0.98276300
C	-2.75799900	-1.00217700	-1.20740200
C	-0.11969900	1.09187200	0.59708100
C	-0.58100700	1.83434700	1.64502300
N	-1.93279100	1.66446700	1.79951500
C	1.32923400	1.03553200	0.18052300
C	1.65978300	1.89290600	-1.06538400
C	0.92942300	1.44819000	-2.33275200
C	1.36167400	3.36529800	-0.77978200
C	1.79721800	-0.42209000	0.03984800
C	3.30051900	-0.52627300	-0.13992700
C	1.49438300	-1.20621000	1.33336700
O	4.10336700	0.28361600	0.25000200
O	1.85478000	-0.76612100	2.39908000
O	3.63906500	-1.67306300	-0.73267600
C	5.05314800	-1.90521300	-0.87825900
C	0.76764800	-2.50705800	1.17599500
H	-4.51311200	0.60766900	1.24965000
H	-0.68260600	-0.82861100	-1.63304400
H	-2.94338200	-1.70609400	-2.01282800
H	-0.02463800	2.48236800	2.31177400
H	-2.49774900	2.09849400	2.51379100
H	1.91382200	1.45053300	1.01162200
H	2.73964100	1.79367100	-1.23316000
H	1.23884800	2.07115700	-3.17939200
H	-0.15483400	1.56091100	-2.21445600
H	1.14423900	0.40636200	-2.59914200
H	1.70326200	3.99507600	-1.60865800
H	1.86015900	3.70292200	0.13701800
H	0.28301700	3.52114700	-0.65631900
H	1.32389600	-0.93983300	-0.79830700
H	5.49427300	-1.11592600	-1.49162100
H	5.52743100	-1.91663900	0.10605400
H	5.14812500	-2.87413800	-1.36653900
H	-0.22029800	-2.30599800	0.74324100
H	0.66646200	-3.01396300	2.13629300
H	1.31210700	-3.13514300	0.46053700
Cl	-5.41947600	-1.24584700	-0.72141100

Compound **1a**, optimization level PCM-M11/6-311++G(2d,p)

Conformation #33

C	3.98775100	-0.01225000	0.47559200
C	3.58811600	0.02004700	-0.83865800
C	2.23823300	-0.23769000	-1.07998400
C	1.32835600	-0.52211200	-0.04102100
C	1.78584900	-0.54437300	1.28133200
C	3.11529800	-0.28866600	1.53778200
C	0.04202700	-0.73964700	-0.64714800
C	0.22361500	-0.57389600	-1.98913900
N	1.54048900	-0.27590500	-2.25622300
C	-1.24473300	-0.97573500	0.09875100
C	-2.31413300	-1.65542900	-0.78109600
C	-1.84652800	-3.06466600	-1.16251100
C	-3.68156600	-1.75172700	-0.10307400
C	-1.66854500	0.37916200	0.74220800
C	-2.06237000	1.37533500	-0.32616900
C	-2.67609500	0.23665900	1.88445200
O	-3.08078900	1.32898300	-0.97544000
O	-2.55352100	-0.68144900	2.66325700
O	-1.12753300	2.30837200	-0.50821700
C	-1.37765600	3.23838400	-1.57715400
C	-3.72127300	1.30269900	2.05569000
Cl	5.67365000	0.30704500	0.85091900
H	4.28964000	0.23662200	-1.63847100
H	1.10641500	-0.76456800	2.10303200
H	3.50025000	-0.29946900	2.55282300
H	-0.49034400	-0.65358400	-2.80000700
H	1.92799100	-0.12993700	-3.17598700
H	-1.04493500	-1.63875400	0.95291800
H	-2.43597400	-1.05994700	-1.69570200
H	-1.75286300	-3.68355400	-0.26024900
H	-2.57836700	-3.54372700	-1.82206700
H	-0.87838300	-3.05679200	-1.67112200
H	-4.37574800	-2.29908300	-0.75051900
H	-4.11794700	-0.76521900	0.08274700
H	-3.60639300	-2.29093300	0.84867800
H	-0.77672700	0.78665000	1.23766300
H	-0.52633500	3.91777400	-1.58810900
H	-1.45462300	2.69573700	-2.52317200
H	-2.30671400	3.78130600	-1.38716100
H	-4.25473500	1.15412600	2.99499400
H	-3.24535500	2.29085800	2.04059500
H	-4.41766100	1.27024300	1.21115400

Compound **1a**, optimization level PCM-M11/6-311++G(2d,p)

Conformation #35

C	4.24481500	-0.11510700	-0.36803400
C	3.77218700	-0.37985100	0.89403600
C	2.39558100	-0.24074500	1.07907200
C	1.52965700	0.15127300	0.03753000
C	2.06156400	0.40432600	-1.23178800
C	3.41768600	0.27105700	-1.43275500
C	0.19764300	0.18868000	0.57898000
C	0.31379800	-0.17995100	1.88948800
N	1.63054300	-0.43560800	2.19618200
C	-1.05146400	0.56338200	-0.17423900
C	-1.48215800	2.03581600	0.04095500
C	-1.98699900	2.30991400	1.45807000
C	-0.33358500	2.98239700	-0.30866400
C	-2.17302300	-0.42706600	0.16482900
C	-3.44043100	-0.12033100	-0.61362300
C	-1.76548500	-1.86015300	-0.22235000
O	-3.46130600	0.18553300	-1.78020600
O	-1.12409700	-2.06463600	-1.22450200
O	-4.53562800	-0.25973100	0.13245800
C	-5.78503100	-0.02805500	-0.54718200
C	-2.22218900	-2.94721700	0.70456000
H	4.43819700	-0.68083200	1.69690600
H	1.41718400	0.70220200	-2.05619800
H	3.85885300	0.46029700	-2.40648900
H	-0.44910800	-0.28382000	2.65244100
H	1.97050700	-0.72792200	3.09979500
H	-0.84563500	0.43662800	-1.24472900
H	-2.30349800	2.22653600	-0.66260100
H	-2.24335900	3.36942800	1.56716400
H	-1.21023600	2.07782700	2.19746600
H	-2.88371000	1.72782400	1.70223200
H	0.05519900	2.78461800	-1.31484100
H	0.49251600	2.86378400	0.40352300
H	-0.66921900	4.02447000	-0.26999800
H	-2.42213400	-0.41586600	1.23021900
H	-6.56374800	-0.18902500	0.19700700
H	-5.88762900	-0.73074300	-1.37754600
H	-5.81283800	0.99690100	-0.92457300
H	-3.29100300	-2.82592100	0.91635700
H	-1.69024700	-2.83112100	1.65743100
H	-2.01896800	-3.93106400	0.28032500
Cl	5.96811200	-0.26732200	-0.67249800

Compound **1a**, optimization level PCM-M11/6-311++G(2d,p)

Conformation #46

C	-3.85532600	-0.59956700	-0.36002400
C	-3.70331800	0.50514800	0.43875000
C	-2.39860700	0.96869100	0.61431300
C	-1.27703400	0.35406700	0.01273600
C	-1.49575700	-0.77102600	-0.79740700
C	-2.77578500	-1.24358900	-0.97973100
C	-0.11582400	1.10638700	0.43228200
C	-0.58519200	2.10173200	1.24084000
N	-1.94903100	2.03079600	1.34968000
C	1.34297900	0.90834400	0.11207600
C	1.77101300	1.47029000	-1.26728500
C	1.12023700	0.75060100	-2.44839300
C	1.48525700	2.97022300	-1.33778700
C	1.75595100	-0.57492800	0.27580700
C	3.25342800	-0.68789500	0.06073800
C	1.37141900	-1.11387200	1.65766600
O	4.08556900	-0.11228500	0.72088200
O	0.72791400	-2.13601200	1.73733300
O	3.55294200	-1.47225000	-0.97156300
C	4.95696200	-1.59574700	-1.27169100
C	1.78557000	-0.32537700	2.86732300
H	-4.55491200	0.98864400	0.90759800
H	-0.67504700	-1.27574500	-1.29844400
H	-2.96152400	-2.11247100	-1.60355000
H	-0.02742400	2.87333500	1.75903100
H	-2.52373300	2.65257200	1.89849400
H	1.91674200	1.47307900	0.86095300
H	2.86079000	1.33447400	-1.33734400
H	1.47563600	1.18295100	-3.39020300
H	0.03059300	0.86904500	-2.41696400
H	1.35714300	-0.31969000	-2.46800000
H	1.90972600	3.39868200	-2.25212000
H	1.91323300	3.49988500	-0.47796600
H	0.40418800	3.15357800	-1.34539200
H	1.26045800	-1.21505400	-0.45428300
H	5.36985900	-0.61096600	-1.50398900
H	5.48068000	-2.02311100	-0.41344100
H	5.01980000	-2.25714700	-2.13461900
H	2.80649500	0.04832000	2.75179500
H	1.68712000	-0.94263600	3.76131900
H	1.11827800	0.54114600	2.95239200
Cl	-5.46894800	-1.23829300	-0.62573600

Compound **1a**, optimization level PCM-M11/6-311++G(2d,p)

Conformation #59

C	4.20264600	0.02276400	-0.45966500
C	3.79782800	-0.53272800	0.72969700
C	2.43426800	-0.45298700	1.01609600
C	1.51583400	0.16306500	0.14098700
C	1.97893500	0.71469100	-1.05881900
C	3.32173900	0.64270400	-1.35776900
C	0.21600600	0.05912300	0.74615400
C	0.39933100	-0.59829500	1.92837100
N	1.72886100	-0.90664200	2.09746700
C	-1.07991400	0.59121200	0.19064500
C	-1.44644500	1.93314500	0.87565900
C	-0.34395500	2.96507300	0.62681000
C	-2.79325100	2.50322600	0.42527600
C	-2.20645000	-0.45600500	0.34010000
C	-3.37030700	-0.17612900	-0.59725200
C	-1.72542400	-1.87680800	0.00082600
O	-3.25323100	0.19454800	-1.73995300
O	-1.94759600	-2.78628700	0.76666500
O	-4.54550400	-0.43311800	-0.02641500
C	-5.70052000	-0.20247500	-0.85544800
C	-1.00084100	-2.06950600	-1.30252500
Cl	5.90562000	-0.04374400	-0.88378400
H	4.50529100	-1.00611400	1.40355200
H	1.29238000	1.19815100	-1.75117800
H	3.71114700	1.06082700	-2.28090700
H	-0.32881300	-0.88759900	2.67738600
H	2.11465500	-1.40285800	2.88663800
H	-0.94949600	0.77841800	-0.88607900
H	-1.49833000	1.72897000	1.95567800
H	-0.24012200	3.15379200	-0.45067200
H	-0.59287100	3.91534800	1.11121300
H	0.62474400	2.62809000	1.00817400
H	-2.94722800	3.48734500	0.88088000
H	-3.63943800	1.87532300	0.72686200
H	-2.81980700	2.62588100	-0.66440100
H	-2.58632600	-0.48550000	1.36638000
H	-6.56376400	-0.45839600	-0.24268100
H	-5.73149800	0.84935100	-1.15240800
H	-5.65087300	-0.83603700	-1.74409700
H	-0.98711500	-3.12852900	-1.56416400
H	-1.46565700	-1.47428100	-2.09400600
H	0.02932300	-1.71362300	-1.18134000

Compound **1a**, optimization level PCM-M11/6-311++G(2d,p)

Conformation #99

C	-3.93676200	-0.34450000	-0.39307500
C	-3.67789900	0.32603200	0.77517900
C	-2.34236600	0.64622100	1.02375600
C	-1.29279900	0.31165000	0.13775800
C	-1.62075300	-0.36999600	-1.04505800
C	-2.93270200	-0.69631000	-1.30511500
C	-0.07061100	0.79740200	0.73978100
C	-0.43856200	1.37885800	1.91930900
N	-1.79479300	1.29887600	2.09370500
C	1.35642700	0.71671500	0.26093900
C	1.73233000	1.78750700	-0.79411000
C	0.99740400	1.61625600	-2.12352500
C	1.49719400	3.18979300	-0.23366700
C	1.69225600	-0.71031400	-0.23176100
C	3.13126000	-0.78655900	-0.70965300
C	1.40705000	-1.77019300	0.84069300
O	3.45552300	-1.13047200	-1.82008600
O	0.77604600	-2.75757500	0.53713000
O	4.00444000	-0.40531100	0.22161600
C	5.38885600	-0.41631000	-0.17706900
C	1.88949400	-1.52946300	2.24372400
H	-4.47240000	0.59088300	1.46621600
H	-0.86232200	-0.63747200	-1.77488600
H	-3.20160800	-1.22290000	-2.21558100
H	0.18901100	1.85352100	2.66493800
H	-2.30172600	1.64786700	2.89319500
H	1.99622900	0.91250800	1.13263700
H	2.81152900	1.67901700	-0.97962100
H	1.31760700	2.39032200	-2.82953500
H	-0.08458900	1.72230500	-1.98031200
H	1.19689400	0.64374000	-2.58920900
H	1.89169400	3.94751700	-0.91924400
H	1.98534600	3.31876200	0.74012500
H	0.42432600	3.37430600	-0.10229500
H	1.08570200	-0.98877900	-1.09336300
H	5.67919100	-1.42697100	-0.47318800
H	5.53340500	0.27044600	-1.01491400
H	5.95307000	-0.08898300	0.69514200
H	2.90663800	-1.12984900	2.24622800
H	1.83035500	-2.45676000	2.81499100
H	1.23440200	-0.77967600	2.70376900
Cl	-5.59507000	-0.77916600	-0.77170300

Compound **1a**, optimization level PCM-ωB97XD/6-311++G(2d,p)

Conformation #1

C	4.21578600	-0.07598000	-0.37048000
C	3.75775200	-0.25909200	0.91414000
C	2.38252400	-0.13405500	1.10420700
C	1.49536100	0.16731100	0.04978100
C	2.01061900	0.34248600	-1.23868000
C	3.36847000	0.22000100	-1.44688700
C	0.17118400	0.20878300	0.60560800
C	0.31392000	-0.06616600	1.93578900
N	1.63610200	-0.26729400	2.24135700
C	-1.09614300	0.49117900	-0.15174200
C	-1.55400300	1.97070000	-0.06892100
C	-1.92312100	2.42381200	1.34171100
C	-0.49716600	2.89483800	-0.67028900
C	-2.21377400	-0.48372200	0.27441400
C	-3.44846200	-0.29035200	-0.58816700
C	-1.79117900	-1.95807200	0.18033800
O	-3.44829300	-0.32067200	-1.79469000
O	-2.05948800	-2.71071000	1.08842300
O	-4.53550200	-0.09078100	0.14798900
C	-5.76130300	0.09798900	-0.57273700
C	-1.06316600	-2.41368500	-1.05058300
H	4.43032900	-0.48985700	1.72970300
H	1.35770800	0.57430900	-2.07199300
H	3.78766600	0.35145100	-2.43565700
H	-0.43488200	-0.14419700	2.70856600
H	1.99008400	-0.49724200	3.15361500
H	-0.89777700	0.29731200	-1.21038800
H	-2.44995500	2.05402500	-0.69479200
H	-2.25598600	3.46387000	1.32246400
H	-1.06033000	2.36218100	2.01003500
H	-2.73214700	1.83002200	1.77255300
H	-0.24485400	2.59979200	-1.69194400
H	0.42105600	2.87855700	-0.07709400
H	-0.86213200	3.92395800	-0.69673100
H	-2.49845500	-0.32209200	1.31255300
H	-6.52387800	0.24955300	0.18544000
H	-5.98576100	-0.78477300	-1.17011300
H	-5.68554500	0.97159300	-1.21903200
H	-0.03748600	-2.03564800	-1.01359100
H	-1.53126600	-2.00425100	-1.94703100
H	-1.04224500	-3.50078100	-1.08697200
Cl	5.93797700	-0.22031000	-0.68164100

Compound **1a**, optimization level PCM-ωB97XD /6-311++G(2d,p)

Conformation #4

C	-3.82449600	-0.11424400	-0.03474100
C	-3.24618400	-0.51175000	1.14888300
C	-1.86467000	-0.69876700	1.13077500
C	-1.08489500	-0.49233400	-0.02748200
C	-1.72275700	-0.09244700	-1.20669200
C	-3.08898800	0.09586600	-1.20904800
C	0.28154900	-0.76725200	0.32326200
C	0.26713500	-1.12070700	1.64126000
N	-1.01536500	-1.08533200	2.12890600
C	1.45926800	-0.66973400	-0.61026100
C	2.26082300	-1.98593700	-0.71375400
C	1.35902400	-3.09421500	-1.25716500
C	3.49938200	-1.84067200	-1.59691300
C	2.33656500	0.58482200	-0.34115400
C	1.43940200	1.79037000	-0.12906000
C	3.32687200	0.50431700	0.81979200
O	1.33771300	2.40393800	0.90482900
O	3.22982400	-0.31777100	1.69999800
O	0.75075200	2.07303600	-1.22911500
C	-0.24216500	3.09894100	-1.10884000
C	4.46169200	1.49019500	0.77331200
Cl	-5.56168700	0.14413500	-0.08190100
H	-3.83449200	-0.66854100	2.04353900
H	-1.15509500	0.08035400	-2.11333800
H	-3.59981700	0.40832700	-2.11032000
H	1.09074400	-1.38961400	2.28141300
H	-1.27817200	-1.29241500	3.07672700
H	1.04636400	-0.49274400	-1.60916300
H	2.58494900	-2.26601200	0.29154700
H	0.47015900	-3.22765300	-0.63782700
H	1.03029100	-2.85848900	-2.27476700
H	1.89638800	-4.04464400	-1.29024100
H	3.97982500	-2.81144800	-1.73625900
H	4.24742000	-1.17081900	-1.16650900
H	3.23047200	-1.45658300	-2.58658100
H	2.92044800	0.79231800	-1.24320800
H	0.22002100	4.04463800	-0.82892600
H	-0.70298400	3.17444900	-2.08957100
H	-0.98358200	2.81015600	-0.36382200
H	4.09741000	2.49410200	0.54897900
H	5.00487300	1.48700000	1.71601400
H	5.13936800	1.20231200	-0.03645300

Compound **1a**, optimization level PCM-ωB97XD/6-311++G(2d,p)

Conformation #6

C	4.13309400	-0.11047600	-0.48013500
C	3.74376200	-0.24455700	0.83285200
C	2.38113000	-0.10368100	1.09150100
C	1.44030000	0.16635100	0.07582900
C	1.88660000	0.29157200	-1.24381900
C	3.23066300	0.15231000	-1.51976500
C	0.14784500	0.23626500	0.69948500
C	0.36007100	0.00669400	2.02902700
N	1.69581300	-0.19235400	2.27059300
C	-1.15717400	0.50004100	0.00074700
C	-1.59772000	1.98631000	0.04582500
C	-1.89050300	2.49824500	1.45419000
C	-0.56696700	2.87532500	-0.64648400
C	-2.25267700	-0.44540000	0.53665600
C	-3.59178700	-0.22338100	-0.14600000
C	-1.88033700	-1.93408400	0.43331700
O	-4.61723800	0.01416400	0.44154900
O	-2.17266600	-2.67576900	1.34316100
O	-3.50293500	-0.32726200	-1.46860500
C	-4.71377200	-0.12207200	-2.20749300
C	-1.17159600	-2.42304900	-0.79697700
H	4.45833900	-0.45022700	1.61898600
H	1.19130800	0.49775600	-2.04910900
H	3.59677200	0.24526700	-2.53367600
H	-0.34579200	-0.03807600	2.84376300
H	2.09668600	-0.39272300	3.17028300
H	-1.01519800	0.26224600	-1.05747400
H	-2.52447900	2.05396700	-0.53605700
H	-2.21498900	3.54019800	1.41098400
H	-0.99520200	2.45327300	2.07973500
H	-2.68161400	1.92963400	1.94774400
H	-0.36972300	2.53646200	-1.66669600
H	0.38048700	2.87499200	-0.10105200
H	-0.92576700	3.90570400	-0.69672200
H	-2.43531900	-0.26310100	1.59433800
H	-4.44349900	-0.25153500	-3.25135300
H	-5.09222900	0.88450100	-2.03280400
H	-5.46369100	-0.85481500	-1.91243200
H	-0.13933500	-2.06241500	-0.77738200
H	-1.64019500	-2.02596600	-1.69818400
H	-1.17099000	-3.51077800	-0.81263500
Cl	5.83534500	-0.27737800	-0.87739900

Compound **1a**, optimization level PCM-ωB97XD/6-311++G(2d,p)

Conformation #7

C	-3.92289500	-0.30294600	-0.40448400
C	-3.65934600	0.32503900	0.78965100
C	-2.32660400	0.64549600	1.04356800
C	-1.27899900	0.35755700	0.13849800
C	-1.60872600	-0.28062700	-1.06577300
C	-2.92053400	-0.61009500	-1.33285900
C	-0.05715900	0.81673800	0.75459500
C	-0.42235400	1.33969000	1.96065800
N	-1.77682600	1.25034800	2.13791300
C	1.36838200	0.75106900	0.27166000
C	1.73779000	1.81839300	-0.78795200
C	0.99470600	1.68159000	-2.11494900
C	1.55489400	3.22228700	-0.21554000
C	1.72697400	-0.68410600	-0.17460700
C	3.16302300	-0.82794800	-0.64376500
C	1.47279200	-1.67737100	0.97499200
O	3.47499900	-1.38256300	-1.67018200
O	1.98172900	-1.51863200	2.05798000
O	4.03429500	-0.29020200	0.19927600
C	5.41512800	-0.38247700	-0.17065400
C	0.56139600	-2.82511500	0.66231200
H	-4.44560600	0.55472900	1.49691000
H	-0.85230400	-0.51820200	-1.80197700
H	-3.18236500	-1.10378800	-2.25931800
H	0.20259200	1.77540700	2.72494200
H	-2.27760200	1.55711400	2.95366200
H	1.99674500	0.95749800	1.14202100
H	2.80697700	1.69151200	-0.99130000
H	1.36416000	2.42470100	-2.82523600
H	-0.07678900	1.85076200	-1.98537200
H	1.13533400	0.69863600	-2.57089600
H	1.92549900	3.97175700	-0.91857500
H	2.09785700	3.34170900	0.72574100
H	0.49915500	3.43140500	-0.02388400
H	1.12072900	-0.98958600	-1.02479400
H	5.71417100	-1.42649300	-0.25632800
H	5.58498300	0.12795500	-1.11824800
H	5.96413600	0.10665900	0.62870900
H	-0.42154100	-2.43063100	0.38787500
H	0.47261300	-3.49287400	1.51655000
H	0.93962000	-3.36752300	-0.20836600
Cl	-5.57938700	-0.74029700	-0.78624800

Compound **1a**, optimization level PCM-ωB97XD/6-311++G(2d,p)

Conformation #8

C	-3.74248300	-0.18867800	-0.13807900
C	-3.19125000	-0.53013000	1.07582000
C	-1.81297800	-0.73813100	1.09340900
C	-1.00963100	-0.60778400	-0.06010900
C	-1.62056100	-0.26420800	-1.27098900
C	-2.98365500	-0.05505600	-1.30823100
C	0.34715100	-0.87428400	0.33262400
C	0.30386800	-1.15242400	1.66657000
N	-0.98710900	-1.07659200	2.12784200
C	1.55087200	-0.80617100	-0.56957500
C	2.50975200	-2.00497600	-0.39358000
C	1.79186000	-3.29634900	-0.78760200
C	3.79214000	-1.85416100	-1.21077900
C	2.25411100	0.58018900	-0.51852000
C	1.21378100	1.65176000	-0.79362400
C	3.07236600	0.89787300	0.73201000
O	0.81263200	1.92379600	-1.89785000
O	3.06164000	0.19677700	1.71585600
O	0.75753700	2.21238800	0.32053900
C	-0.34666900	3.11390600	0.17274200
C	3.95912400	2.11164900	0.63500300
Cl	-5.47385900	0.09527600	-0.23021200
H	-3.79747200	-0.62845700	1.96680100
H	-1.03426700	-0.14772600	-2.17448800
H	-3.47354400	0.21470500	-2.23455200
H	1.11034400	-1.38784800	2.34089900
H	-1.27110200	-1.23843300	3.07832900
H	1.17663000	-0.87136300	-1.59882600
H	2.78426300	-2.07008400	0.66167200
H	1.53664400	-3.28401200	-1.85233200
H	2.43309200	-4.16203500	-0.60703500
H	0.86947600	-3.43243600	-0.22032300
H	4.38314000	-2.77079100	-1.15435000
H	4.42609400	-1.03996600	-0.85281600
H	3.56450100	-1.66947600	-2.26595200
H	2.94574400	0.64210800	-1.36290800
H	-0.57034900	3.46749000	1.17498500
H	-0.07473500	3.94594800	-0.47538800
H	-1.20083200	2.57938700	-0.24418800
H	3.44070300	2.95428900	0.17488900
H	4.32616900	2.38890100	1.62103300
H	4.80960700	1.86441600	-0.00763800

Compound **1a**, optimization level PCM-ωB97XD/6-311++G(2d,p)

Conformation #9

C	-4.33232900	-0.23326200	-0.33667200
C	-3.88448300	0.57908400	0.68022300
C	-2.50484900	0.62213000	0.87522200
C	-1.60546600	-0.11953600	0.08134400
C	-2.10963700	-0.93017200	-0.94042300
C	-3.47233300	-0.98572700	-1.14792100
C	-0.28043900	0.17281900	0.55251000
C	-0.43315100	1.05207400	1.58496000
N	-1.76439400	1.32480500	1.78415900
C	0.98188900	-0.43172500	0.00416100
C	1.21633000	-1.83631200	0.62890900
C	2.16409400	-2.72255600	-0.18020700
C	1.64732900	-1.77917800	2.09293800
C	2.16522600	0.54723400	0.15975400
C	3.40205700	0.06945000	-0.57935100
C	1.84802000	1.95849400	-0.36791000
O	3.44925100	-0.14095100	-1.76636300
O	2.18265400	2.92000300	0.28547200
O	4.44059400	-0.06790700	0.23870600
C	5.66156000	-0.51797100	-0.36358700
C	1.15339400	2.08956200	-1.69185400
Cl	-6.05964400	-0.33489300	-0.63631700
H	-4.56881100	1.15200500	1.29207200
H	-1.44468600	-1.51347500	-1.56706300
H	-3.88518100	-1.60616100	-1.93230100
H	0.31082200	1.52317800	2.20807900
H	-2.12678500	1.95629400	2.47695500
H	0.83272000	-0.58683600	-1.06970900
H	0.23194400	-2.31462700	0.59424600
H	3.20673200	-2.41291000	-0.07856500
H	2.10326400	-3.75171300	0.18120700
H	1.90922200	-2.71999900	-1.24220500
H	1.62608300	-2.78025500	2.52889800
H	0.98579600	-1.14173400	2.68374000
H	2.66859200	-1.40092600	2.19249400
H	2.42649600	0.67189700	1.20950400
H	6.38156900	-0.57736600	0.44718400
H	5.51595900	-1.49783200	-0.81811700
H	5.99300900	0.19237100	-1.11981700
H	1.62645000	1.44283600	-2.43220200
H	0.11616800	1.76183200	-1.58351200
H	1.17209200	3.12735000	-2.01769700

Compound **1a**, optimization level PCM-ωB97XD/6-311++G(2d,p)

Conformation #16

C	-4.15962400	0.00642600	-0.49700000
C	-3.76229600	0.44986400	0.74343800
C	-2.39765400	0.37408000	1.01971100
C	-1.46275000	-0.12910900	0.09099400
C	-1.91732600	-0.56480000	-1.15771000
C	-3.26336700	-0.49669100	-1.45007700
C	-0.16719000	-0.05785300	0.70656300
C	-0.37131200	0.47508100	1.94739100
N	-1.70589800	0.73456800	2.14164000
C	1.12938100	-0.49886900	0.08346000
C	1.56881800	-1.92182600	0.51114400
C	1.89798600	-2.04405700	1.99750900
C	0.51989700	-2.95525700	0.10577000
C	2.22089900	0.55707200	0.33326200
C	3.57692800	0.15539600	-0.22354800
C	1.83332100	1.91735400	-0.27386300
O	4.59595500	0.14927600	0.42283900
O	1.25204900	1.99102100	-1.32872400
O	3.51084000	-0.17816500	-1.50645700
C	4.73817500	-0.58940000	-2.12068600
C	2.22901700	3.12310200	0.52499400
H	-4.47233900	0.83591400	1.46294300
H	-1.22572100	-0.95571000	-1.89469600
H	-3.63585600	-0.82881700	-2.41012400
H	0.33670500	0.69737000	2.73072700
H	-2.10152300	1.13945700	2.97220500
H	0.97547900	-0.52853100	-0.99799900
H	2.47989600	-2.14933400	-0.05406600
H	2.22951300	-3.05990700	2.22381600
H	1.01751000	-1.83676900	2.61125400
H	2.69595100	-1.36369200	2.30377700
H	0.29350200	-2.89453500	-0.96178400
H	-0.41203500	-2.80490500	0.65734000
H	0.87757100	-3.96520700	0.31861600
H	2.38533700	0.70147800	1.40069600
H	4.48573000	-0.81775200	-3.15200500
H	5.13052900	-1.47347400	-1.61899900
H	5.47069900	0.21560200	-2.07558700
H	3.28210000	3.05599200	0.81011300
H	1.64904500	3.13054600	1.45271900
H	2.04256900	4.03727300	-0.03445100
Cl	-5.86538400	0.07193500	-0.91090300

Compound **1a**, optimization level PCM-ωB97XD/6-311++G(2d,p)

Conformation #19

C	-3.83535900	-0.58006000	-0.41591100
C	-3.68333600	0.32574900	0.60700100
C	-2.39185800	0.80018700	0.83136400
C	-1.27787400	0.39497800	0.06067000
C	-1.49410100	-0.52901200	-0.97146200
C	-2.76317900	-1.01417100	-1.20539300
C	-0.12633000	1.07837500	0.59871200
C	-0.59319200	1.83506400	1.63350000
N	-1.94657200	1.68230200	1.77421700
C	1.32394900	1.01874800	0.19405800
C	1.66512300	1.82981500	-1.08140200
C	1.00528500	1.31861600	-2.36033200
C	1.34432800	3.30832900	-0.87258200
C	1.81799300	-0.44003700	0.12637300
C	3.31478600	-0.53524400	-0.10414200
C	1.52614000	-1.16813600	1.45457000
O	4.13555400	0.23040200	0.33255700
O	1.95859400	-0.73805500	2.49645500
O	3.61619400	-1.61326400	-0.82623300
C	5.01069000	-1.84671600	-1.06204500
C	0.69799600	-2.41323200	1.36572900
H	-4.52235700	0.65035900	1.20848300
H	-0.68143600	-0.87010600	-1.59912600
H	-2.93797500	-1.72837200	-1.99911500
H	-0.04328400	2.48392200	2.29767900
H	-2.51178600	2.12646100	2.47671400
H	1.89006200	1.48265900	1.00650400
H	2.74872100	1.74995700	-1.21087100
H	1.36205100	1.89549400	-3.21668300
H	-0.08070700	1.42873600	-2.31642900
H	1.23603900	0.26900000	-2.55884700
H	1.69127200	3.89739100	-1.72471900
H	1.82797200	3.69665500	0.02764600
H	0.26719400	3.46474600	-0.77005800
H	1.32749400	-0.98902500	-0.67545600
H	5.44458200	-1.01066900	-1.60931800
H	5.53548500	-1.97978100	-0.11661100
H	5.05812400	-2.75530400	-1.65520800
H	-0.28073800	-2.16134500	0.94769400
H	0.58153500	-2.87030700	2.34603900
H	1.16909600	-3.11463400	0.67185300
Cl	-5.43437800	-1.22200800	-0.75083400

Compound **1a**, optimization level PCM-ωB97XD/6-311++G(2d,p)

Conformation #33

C	4.03774100	-0.06777400	0.45157900
C	3.61277200	0.33462700	-0.79445000
C	2.26237000	0.13988700	-1.08022900
C	1.36975800	-0.44255100	-0.15582800
C	1.85009500	-0.83407000	1.09790000
C	3.18317800	-0.64588600	1.39984500
C	0.07866700	-0.49276500	-0.78239500
C	0.24052900	0.05347800	-2.02167800
N	1.54805000	0.43310300	-2.20771900
C	-1.19102600	-0.96368500	-0.12625500
C	-2.18852900	-1.57118500	-1.13560100
C	-1.58411800	-2.83033700	-1.76279300
C	-3.54756300	-1.91355200	-0.52832200
C	-1.72215100	0.19714200	0.76666900
C	-2.20787900	1.36760400	-0.06094200
C	-2.68565100	-0.24766900	1.87233000
O	-3.17586300	1.35571500	-0.78191400
O	-2.46676200	-1.27875900	2.46479200
O	-1.41628400	2.42840000	0.08350500
C	-1.75234500	3.57766600	-0.70250500
C	-3.81201200	0.66830200	2.25424700
Cl	5.72749000	0.15127600	0.87785400
H	4.29245700	0.77856600	-1.50995000
H	1.18951200	-1.28305600	1.83104700
H	3.57683300	-0.94173800	2.36330000
H	-0.48044000	0.20403600	-2.80988100
H	1.91787700	0.83959400	-3.04918500
H	-0.93199100	-1.75433500	0.58588000
H	-2.36275900	-0.83920000	-1.92983100
H	-1.43249200	-3.59993300	-0.99915500
H	-2.25800700	-3.23908900	-2.51910000
H	-0.62249600	-2.63157100	-2.23742200
H	-4.18033700	-2.38325000	-1.28501400
H	-4.07360600	-1.02515000	-0.17629000
H	-3.44336500	-2.61277500	0.30501100
H	-0.85981300	0.55898100	1.33628400
H	-1.00669500	4.32809900	-0.45645000
H	-1.71033500	3.33151800	-1.76316900
H	-2.75030100	3.93212100	-0.44676400
H	-4.32092900	0.28792600	3.13696500
H	-3.42312900	1.67165100	2.45016200
H	-4.51541300	0.75596100	1.42358200

Compound **1a**, optimization level PCM-ωB97XD/6-311++G(2d,p)

Conformation #35

C	4.22808900	-0.12475300	-0.38706100
C	3.75994700	-0.36749800	0.88381100
C	2.38725200	-0.21686300	1.07639000
C	1.51264500	0.16362500	0.03707200
C	2.03809600	0.39658000	-1.23783300
C	3.39343400	0.25235300	-1.44817200
C	0.18829900	0.21607800	0.58997000
C	0.31816800	-0.12903000	1.90518200
N	1.63342300	-0.38788100	2.20317700
C	-1.06560700	0.58788400	-0.15456600
C	-1.50207800	2.05768100	0.06886800
C	-1.91548100	2.36841800	1.50595300
C	-0.40763400	3.01782300	-0.39332200
C	-2.18422600	-0.41963900	0.15868500
C	-3.44502500	-0.14901600	-0.64304600
C	-1.73496200	-1.85366500	-0.18352700
O	-3.46549900	0.16318900	-1.80649400
O	-1.19025400	-2.09666700	-1.23266300
O	-4.53657100	-0.33201900	0.09635600
C	-5.79138600	-0.14655000	-0.57225800
C	-2.02382700	-2.89997600	0.84972000
H	4.42299000	-0.65955100	1.68748800
H	1.39419300	0.68885400	-2.05896700
H	3.82052000	0.42759300	-2.42675000
H	-0.43288600	-0.21387800	2.67533600
H	1.97782400	-0.66992500	3.10431200
H	-0.85630800	0.48202700	-1.22224100
H	-2.37208500	2.22292700	-0.57478600
H	-2.24099200	3.40821400	1.58326000
H	-1.07721800	2.23080300	2.19399600
H	-2.74297200	1.74169700	1.84695200
H	-0.11849700	2.82240900	-1.42910000
H	0.48586400	2.92322000	0.22980300
H	-0.75486900	4.05155000	-0.32880300
H	-2.45282400	-0.39331100	1.21431500
H	-6.55268900	-0.34266300	0.17706600
H	-5.88257900	-0.84605000	-1.40237800
H	-5.87285800	0.87533400	-0.94082000
H	-3.08145400	-2.86678400	1.12394400
H	-1.45293300	-2.66600100	1.75296100
H	-1.75545500	-3.88939000	0.48573800
Cl	5.94797900	-0.29462600	-0.70037300

Compound **1a**, optimization level PCM-ωB97XD/6-311++G(2d,p)

Conformation #46

C	-3.86320600	-0.60224200	-0.37646900
C	-3.70861100	0.43135500	0.51627100
C	-2.40840400	0.89563000	0.71036400
C	-1.28944900	0.35447700	0.03762000
C	-1.50769500	-0.69754000	-0.86250900
C	-2.78555700	-1.17222000	-1.06636700
C	-0.13256800	1.08156900	0.50033900
C	-0.60093900	1.99352300	1.40152400
N	-1.96019900	1.89372400	1.52857500
C	1.32063600	0.94054600	0.13348500
C	1.70607200	1.62336200	-1.20469700
C	1.01415500	1.03853300	-2.43408600
C	1.46783900	3.12948800	-1.12453800
C	1.78305000	-0.53378100	0.18000600
C	3.28778500	-0.62315300	-0.00812000
C	1.41351500	-1.23509900	1.49552200
O	4.09717200	0.00693500	0.62741300
O	0.85313000	-2.30622600	1.45933400
O	3.60865300	-1.49375900	-0.95861400
C	5.01009300	-1.66390700	-1.21256800
C	1.76258500	-0.55689600	2.78834500
H	-4.55161000	0.86092100	1.04128500
H	-0.69026500	-1.14520100	-1.41165700
H	-2.96355300	-1.98392300	-1.75926200
H	-0.04921900	2.72291300	1.97480000
H	-2.52973900	2.45254700	2.13994000
H	1.89054900	1.46578000	0.90609400
H	2.78519900	1.47504600	-1.32647400
H	1.38108700	1.53341100	-3.33604400
H	-0.06652300	1.19197000	-2.38645900
H	1.20346600	-0.03088700	-2.55080200
H	1.84477600	3.62255600	-2.02339200
H	1.97381500	3.56700400	-0.25993200
H	0.40105900	3.35227900	-1.03950700
H	1.31645300	-1.12028900	-0.60748700
H	5.45053700	-0.71988700	-1.53125600
H	5.51391000	-2.02183900	-0.31558100
H	5.07426100	-2.40188700	-2.00668100
H	2.75817800	-0.11448400	2.73897400
H	1.69306400	-1.26746200	3.60930600
H	1.04949900	0.25483700	2.95551600
Cl	-5.47358700	-1.23687900	-0.66948200

Compound **1a**, optimization level PCM-ωB97XD/6-311++G(2d,p)

Conformation #59

C	4.17502500	-0.06660800	-0.48570900
C	3.78639900	-0.36693600	0.79986000
C	2.43022300	-0.21840000	1.08683900
C	1.49541400	0.21540900	0.12314600
C	1.94053600	0.50861600	-1.16977600
C	3.27867800	0.36684700	-1.47227500
C	0.20988300	0.24312600	0.76012300
C	0.41740000	-0.16035200	2.04707100
N	1.74658000	-0.43736500	2.24993900
C	-1.09397300	0.65892100	0.13308900
C	-1.51584300	2.06085700	0.64514800
C	-0.43534800	3.09125900	0.31533300
C	-2.86336500	2.54262000	0.10846400
C	-2.18539700	-0.41027100	0.37638600
C	-3.33439600	-0.30726700	-0.61392500
C	-1.65801200	-1.85169600	0.25991700
O	-3.20486700	-0.09715200	-1.79421000
O	-1.89465300	-2.64690900	1.13949600
O	-4.50528700	-0.53323400	-0.02611700
C	-5.65635300	-0.49599800	-0.87994000
C	-0.87884800	-2.23013400	-0.96884100
Cl	5.86942600	-0.23345900	-0.91596300
H	4.49699900	-0.70081800	1.54456400
H	1.24801500	0.84385800	-1.93315600
H	3.64484700	0.58751100	-2.46622500
H	-0.28937400	-0.28112800	2.85338900
H	2.14305800	-0.76866900	3.11210400
H	-0.94472200	0.72883000	-0.94936100
H	-1.59396100	1.98582700	1.73654000
H	-0.31168000	3.18213100	-0.76866200
H	-0.71537800	4.07295900	0.70335800
H	0.53081400	2.82066900	0.74343800
H	-3.05799000	3.55754700	0.46187700
H	-3.69668200	1.92425100	0.44767100
H	-2.86891600	2.56051600	-0.98457200
H	-2.59131100	-0.31968700	1.38449500
H	-6.50449800	-0.70589900	-0.23494100
H	-5.75488000	0.49076100	-1.33162400
H	-5.57216200	-1.25165700	-1.65997400
H	-0.96446500	-3.30174500	-1.14021900
H	-1.21010700	-1.67092400	-1.84362000
H	0.17240100	-1.98558500	-0.79608500

Compound **1a**, optimization level PCM-ωB97XD/6-311++G(2d,p)

Conformation #99

C	-3.94688900	-0.34488800	-0.38453400
C	-3.67835400	0.30694500	0.79535900
C	-2.34332100	0.62680800	1.03891200
C	-1.29928600	0.31276500	0.13901000
C	-1.63362200	-0.35008900	-1.05026500
C	-2.94762800	-0.67704200	-1.30805800
C	-0.07588500	0.79051900	0.73689300
C	-0.43672600	1.34884900	1.92896700
N	-1.78971500	1.26118300	2.11483000
C	1.34495300	0.72998800	0.24163000
C	1.69914700	1.81678900	-0.80642900
C	0.92472600	1.70450800	-2.11781800
C	1.53706400	3.21179500	-0.20729800
C	1.70949300	-0.68911400	-0.25186700
C	3.15731600	-0.78894300	-0.70361000
C	1.41933800	-1.78546100	0.78543700
O	3.49441500	-1.22537200	-1.77591200
O	0.83724300	-2.78593100	0.43429400
O	4.00969800	-0.34725100	0.21562800
C	5.40025800	-0.39504000	-0.12849300
C	1.85992300	-1.57907400	2.20619400
H	-4.46210700	0.55755000	1.49821900
H	-0.88004600	-0.60614400	-1.78288000
H	-3.21415700	-1.18875200	-2.22333900
H	0.18989400	1.81157600	2.67613500
H	-2.28795300	1.59299800	2.92231400
H	1.98279600	0.93805100	1.10562900
H	2.76340500	1.68842500	-1.03594300
H	1.27049000	2.46755400	-2.81869400
H	-0.14457300	1.86065900	-1.95696100
H	1.06117900	0.73418500	-2.60062100
H	1.89858300	3.96979400	-0.90574800
H	2.09984400	3.31295600	0.72444700
H	0.48690700	3.42480600	0.00852400
H	1.12350800	-0.96042000	-1.12623500
H	5.70162800	-1.42057100	-0.33774500
H	5.59129600	0.23104800	-0.99942700
H	5.92824300	-0.01124700	0.73950200
H	2.86428800	-1.15786300	2.25027100
H	1.81315200	-2.52251500	2.74595200
H	1.18143200	-0.86279700	2.67727700
Cl	-5.60668800	-0.77970800	-0.75533800

Compound **1a**, optimization level SMD-B3LYP/6-311++G(2d,p)

Conformation #1

C	4.24167800	-0.14625100	-0.37714300
C	3.78645200	-0.22005500	0.92477300
C	2.41260900	-0.05791700	1.11054000
C	1.51414300	0.17538000	0.03436000
C	2.03217800	0.23849100	-1.26761600
C	3.39311800	0.07744400	-1.47188900
C	0.19084800	0.28368300	0.59663800
C	0.34387100	0.11170500	1.95090000
N	1.66990700	-0.08969300	2.26155000
C	-1.08836700	0.53793200	-0.16676600
C	-1.55652800	2.02942300	-0.14058900
C	-1.86057200	2.57627600	1.25789400
C	-0.55571900	2.93706800	-0.86346200
C	-2.22232800	-0.41969400	0.29616200
C	-3.46353700	-0.28460600	-0.58060900
C	-1.81427500	-1.91036500	0.29002600
O	-3.45733100	-0.24431700	-1.79050100
O	-2.02575500	-2.58940800	1.27472000
O	-4.58055200	-0.25301200	0.15721500
C	-5.82907200	-0.17765300	-0.57038200
C	-1.18498500	-2.48255100	-0.94987700
Cl	5.97552100	-0.34427500	-0.68365900
H	4.45722800	-0.39644300	1.75559900
H	1.38184700	0.41226600	-2.11650600
H	3.80616600	0.12367600	-2.47095900
H	-0.39526500	0.10780700	2.73612000
H	2.03057700	-0.24690400	3.19110200
H	-0.89138100	0.30779800	-1.21646900
H	-2.48737500	2.06419000	-0.71651800
H	-0.95799600	2.62934100	1.87195800
H	-2.26401700	3.58945800	1.17809100
H	-2.59809700	1.97364700	1.79300800
H	-0.94527200	3.95708000	-0.92379800
H	-0.36559500	2.59192600	-1.88321000
H	0.40107400	2.98035000	-0.33641400
H	-2.51195300	-0.20320500	1.32207400
H	-6.60510900	-0.16524700	0.19076800
H	-5.86540400	0.73446000	-1.16503600
H	-5.94267800	-1.04802900	-1.21587900
H	-1.15901200	-3.56922700	-0.88135900
H	-1.72670500	-2.17082400	-1.84471500
H	-0.16222900	-2.10754900	-1.04572600

Compound **1a**, optimization level SMD-B3LYP/6-311++G(2d,p)

Conformation #16

C	4.18527100	-0.07378700	-0.49992900
C	3.79020400	-0.34308900	0.79573300
C	2.42564200	-0.22307100	1.06449200
C	1.47813100	0.15749300	0.07567100
C	1.93637500	0.41567300	-1.22465300
C	3.28700100	0.29983000	-1.51108100
C	0.18194000	0.17144400	0.70599100
C	0.39668700	-0.19576500	2.01188200
N	1.73693000	-0.42975400	2.23032200
C	-1.13204400	0.52268600	0.04499200
C	-1.60791100	1.98414300	0.31997700
C	-1.85544000	2.30569300	1.79781400
C	-0.64631100	3.00638400	-0.29527300
C	-2.22049900	-0.52504600	0.40104600
C	-3.58933300	-0.19909900	-0.19309000
C	-1.83041500	-1.94867500	-0.06046700
O	-4.60703600	-0.10508500	0.45699300
O	-1.21366400	-2.13576800	-1.08666100
O	-3.54087400	-0.04633400	-1.52068100
C	-4.78881400	0.26638900	-2.17984600
C	-2.27433200	-3.07445400	0.83113200
Cl	5.90426500	-0.20698500	-0.90895400
H	4.49870800	-0.63313100	1.56072100
H	1.24703700	0.70372400	-2.00929100
H	3.65394300	0.49495500	-2.51019500
H	-0.30526600	-0.31778700	2.82138200
H	2.14068600	-0.72050600	3.10851900
H	-0.97782100	0.44871300	-1.03285700
H	-2.56338500	2.09779900	-0.20443500
H	-0.92554700	2.28497300	2.37168200
H	-2.27818900	3.30966700	1.89298800
H	-2.55604700	1.61086900	2.26664300
H	-1.04437700	4.01843900	-0.18035300
H	-0.49871800	2.82505900	-1.36324100
H	0.33251400	2.97996100	0.19087200
H	-2.38186100	-0.55885800	1.47721000
H	-4.54376500	0.35097200	-3.23555000
H	-5.51194000	-0.53298400	-2.02044100
H	-5.18814100	1.20880500	-1.80593400
H	-3.34139900	-2.98589000	1.05502100
H	-2.06694300	-4.03772100	0.36706800
H	-1.74482500	-3.00303800	1.78660400

Compound **1a**, optimization level SMD-B3LYP/6-311++G(2d,p)

Conformation #35

C	4.26178300	-0.12510600	-0.38258100
C	3.79575900	-0.32199500	0.90249700
C	2.42085900	-0.17257200	1.09246600
C	1.53200800	0.16720500	0.03667000
C	2.06087100	0.35128900	-1.24935700
C	3.42282800	0.20514100	-1.45770700
C	0.20517300	0.23135800	0.59594900
C	0.34590000	-0.06894400	1.92882300
N	1.66902200	-0.30992600	2.22936000
C	-1.06587500	0.56800400	-0.15084800
C	-1.53954900	2.04359700	0.04195600
C	-1.86737100	2.42505500	1.48951200
C	-0.53110400	3.03133800	-0.55472600
C	-2.18666900	-0.45236200	0.17534600
C	-3.45035700	-0.18978200	-0.63906400
C	-1.75622000	-1.90390700	-0.14569000
O	-3.47270700	0.01694500	-1.83021200
O	-1.12526800	-2.16264700	-1.14732300
O	-4.55003700	-0.24625800	0.12654700
C	-5.81347100	-0.05207900	-0.55077600
C	-2.18355300	-2.95792400	0.83616200
Cl	5.99792700	-0.29867900	-0.69263600
H	4.45906000	-0.57948300	1.71799000
H	1.41761100	0.60565800	-2.08323100
H	3.84406100	0.34375600	-2.44476700
H	-0.39834800	-0.13748100	2.70609500
H	2.02236200	-0.54972300	3.14392100
H	-0.85548700	0.45196900	-1.21573600
H	-2.46140200	2.14439900	-0.54028000
H	-0.97379100	2.41433300	2.11871400
H	-2.27901400	3.43767900	1.52109900
H	-2.60625200	1.75908500	1.94157700
H	-0.92183600	4.05133500	-0.50063000
H	-0.32555100	2.80811200	-1.60504600
H	0.41830300	3.01314200	-0.01282600
H	-2.45710600	-0.40930100	1.22884800
H	-6.57210100	-0.14550500	0.22225200
H	-5.85137400	0.93875800	-1.00234700
H	-5.95386100	-0.81507300	-1.31574900
H	-3.26011800	-2.89185800	1.01856800
H	-1.92547100	-3.95111000	0.47115800
H	-1.68957000	-2.77587200	1.79579400

Compound **1a**, optimization level SMD-M06-2x/6-311++G(2d,p)

Conformation #1

C	4.23199600	-0.06605900	-0.36091200
C	3.76358900	-0.35692500	0.90026200
C	2.38454400	-0.24418300	1.08606900
C	1.50700400	0.14944700	0.04928500
C	2.03624900	0.43068900	-1.21733600
C	3.39770600	0.32196500	-1.42050600
C	0.17626900	0.14864300	0.59438200
C	0.30435800	-0.24160800	1.90026900
N	1.62534500	-0.47240400	2.19992800
C	-1.08727200	0.49894100	-0.14297200
C	-1.51184300	1.98080700	0.02332300
C	-1.85555500	2.36306600	1.46080800
C	-0.43721600	2.91370800	-0.52917100
C	-2.22192600	-0.46489600	0.25617600
C	-3.46569400	-0.19882100	-0.57166000
C	-1.84168200	-1.93910700	0.05507500
O	-3.47198100	-0.08615400	-1.77200300
O	-2.10305900	-2.74508100	0.91687900
O	-4.56005600	-0.12499900	0.18141300
C	-5.78654500	0.10234000	-0.52908000
C	-1.16083600	-2.32418500	-1.22611700
Cl	5.95627500	-0.18720300	-0.66604400
H	4.42711100	-0.65800700	1.70056000
H	1.38928200	0.73147900	-2.03342300
H	3.83080900	0.53322200	-2.38927200
H	-0.45344200	-0.38107400	2.65661500
H	1.97418900	-0.77972800	3.09624400
H	-0.89968200	0.35166000	-1.21150100
H	-2.41048500	2.11639000	-0.58882000
H	-0.98575500	2.25094700	2.11298100
H	-2.16681300	3.40927900	1.49926700
H	-2.67051600	1.76327600	1.87119100
H	-0.78730400	3.94803900	-0.50986100
H	-0.18195000	2.66049500	-1.56092400
H	0.47473500	2.85808200	0.07118100
H	-2.47779500	-0.35903800	1.30898200
H	-6.56266400	0.14117100	0.22970200
H	-5.73563300	1.04579300	-1.07077600
H	-5.97384000	-0.71424300	-1.22485800
H	-1.14775100	-3.40757500	-1.32353500
H	-1.66134900	-1.86486800	-2.07997300
H	-0.13336100	-1.94909000	-1.20821900

Compound **1a**, optimization level SMD-M06-2x/6-311++G(2d,p)

Conformation #4

C	-3.81171300	-0.12709200	-0.03364700
C	-3.23813000	-0.57988300	1.13242300
C	-1.85402500	-0.76344900	1.10712900
C	-1.06988000	-0.49843300	-0.04053600
C	-1.70747600	-0.04256800	-1.20325400
C	-3.07571500	0.14124200	-1.19837000
C	0.29734700	-0.79320100	0.29896700
C	0.27903200	-1.21482500	1.60056500
N	-1.00710900	-1.20114700	2.08551300
C	1.47690300	-0.65723800	-0.62979300
C	2.31823600	-1.94831400	-0.72706300
C	1.47454200	-3.06278000	-1.34447100
C	3.58559200	-1.74325200	-1.55450900
C	2.31993200	0.61323400	-0.34065100
C	1.39602800	1.79421100	-0.11800700
C	3.28441900	0.53875600	0.83783100
O	1.30086100	2.41391600	0.91190000
O	3.18889500	-0.30533000	1.69686400
O	0.67103500	2.05331500	-1.20376700
C	-0.32715400	3.07173100	-1.05561500
C	4.38941800	1.55708300	0.83517300
Cl	-5.54834900	0.12829100	-0.07345500
H	-3.82727600	-0.78255500	2.01747300
H	-1.13696900	0.17368100	-2.09900700
H	-3.58909100	0.49369200	-2.08333400
H	1.09521600	-1.52428500	2.23248700
H	-1.27611300	-1.45602300	3.02475500
H	1.06541500	-0.48706700	-1.63080100
H	2.60521700	-2.24593800	0.28420000
H	0.55914000	-3.24011100	-0.77678800
H	1.19329700	-2.80326300	-2.36990700
H	2.03822300	-3.99753000	-1.37711500
H	4.09892600	-2.69627000	-1.69874100
H	4.29338000	-1.06022900	-1.07909200
H	3.33967000	-1.34214100	-2.54305800
H	2.91546200	0.84741400	-1.22906000
H	0.14295500	4.03151200	-0.84447200
H	-0.85456100	3.10953500	-2.00481100
H	-1.01280400	2.81038500	-0.24987900
H	4.02011200	2.53801900	0.53365600
H	4.86121600	1.60836900	1.81430300
H	5.12982800	1.24335300	0.09231000

Compound **1a**, optimization level SMD-M06-2x/6-311++G(2d,p)

Conformation #6

C	4.15744200	-0.06717200	-0.46865400
C	3.76006100	-0.31446900	0.82574700
C	2.39196400	-0.20468200	1.08123800
C	1.45688700	0.14481600	0.07962000
C	1.91464500	0.38195700	-1.22328700
C	3.26414800	0.27508000	-1.49544100
C	0.15688900	0.15315400	0.69451200
C	0.35777900	-0.18971400	2.00481400
N	1.69515200	-0.39885500	2.24112300
C	-1.14506400	0.46923400	0.00994000
C	-1.57717200	1.95159000	0.14832000
C	-1.88670700	2.36463700	1.58485100
C	-0.52812300	2.87981600	-0.45770700
C	-2.24518500	-0.49226000	0.50332900
C	-3.59164700	-0.17006100	-0.11721400
C	-1.92351800	-1.97226800	0.24578900
O	-4.58390500	0.09237300	0.51254100
O	-2.25140900	-2.79377600	1.07012700
O	-3.55344800	-0.21266800	-1.44909500
C	-4.78220800	0.10511400	-2.11826900
C	-1.21852600	-2.35471800	-1.02362700
Cl	5.86367900	-0.18747500	-0.86189800
H	4.46836700	-0.58131000	1.59930400
H	1.22196900	0.64675900	-2.01392400
H	3.64265500	0.45207100	-2.49349000
H	-0.35666100	-0.30812700	2.80567400
H	2.09435600	-0.67038300	3.12788500
H	-1.00400000	0.29125300	-1.06070700
H	-2.49409200	2.06447700	-0.44275600
H	-1.00026700	2.26762600	2.21685600
H	-2.19930300	3.41085100	1.60910900
H	-2.68951500	1.77080100	2.02636000
H	-0.89068200	3.90997600	-0.46018600
H	-0.29451900	2.59904500	-1.48747700
H	0.39853400	2.85258700	0.12161000
H	-2.38620400	-0.39916700	1.57895800
H	-4.57156200	0.01355100	-3.17988300
H	-5.56311700	-0.59391700	-1.82280700
H	-5.08557800	1.12319400	-1.87693400
H	-1.68813100	-1.87985500	-1.88592200
H	-0.18451600	-2.00102600	-0.97763800
H	-1.22622300	-3.43708700	-1.13197100

Compound **1a**, optimization level SMD-M06-2x/6-311++G(2d,p)

Conformation #16

C	4.19180600	-0.03474100	-0.47345800
C	3.78489800	-0.44126900	0.77702800
C	2.41552900	-0.35736300	1.03642400
C	1.48765800	0.11846200	0.08066000
C	1.95587800	0.51732400	-1.17825900
C	3.30670200	0.43974300	-1.45376400
C	0.18434600	0.06108300	0.68485300
C	0.37576600	-0.43873300	1.94439700
N	1.71154800	-0.68722000	2.16006700
C	-1.10928400	0.47589900	0.03721800
C	-1.53691600	1.92372000	0.38473600
C	-1.87081200	2.12090200	1.86140200
C	-0.47444500	2.92540200	-0.05962900
C	-2.21035800	-0.54823100	0.36249800
C	-3.56391100	-0.09467400	-0.15596500
C	-1.90087100	-1.93059800	-0.23031400
O	-4.52383800	0.13037200	0.53649000
O	-1.17636600	-2.06009000	-1.18585400
O	-3.56266000	0.03771800	-1.48097800
C	-4.78382900	0.51761600	-2.05977100
C	-2.55564600	-3.09196500	0.45750800
Cl	5.90034800	-0.11409000	-0.86916900
H	4.48701800	-0.80621500	1.51546200
H	1.26977200	0.88343200	-1.93330500
H	3.69224700	0.74089100	-2.41883700
H	-0.34240000	-0.64619900	2.72339500
H	2.10382000	-1.06991400	3.00810300
H	-0.95690700	0.43995300	-1.04473600
H	-2.44269600	2.13086600	-0.19811100
H	-0.99188100	1.94172500	2.48578200
H	-2.19731100	3.14878000	2.03463700
H	-2.67149600	1.45867000	2.19725600
H	-0.83212000	3.94716200	0.08528600
H	-0.22550200	2.79903700	-1.11605300
H	0.44285900	2.80598200	0.52291100
H	-2.33388600	-0.66581400	1.43973800
H	-4.60353800	0.56658100	-3.12973200
H	-5.59962500	-0.17025900	-1.84241400
H	-5.01992100	1.50638400	-1.66786800
H	-3.61913500	-2.89215100	0.61122500
H	-2.41831600	-4.00604100	-0.11588700
H	-2.10389300	-3.20225500	1.44791500

Compound **1a**, optimization level SMD-M06-2x/6-311++G(2d,p)

Conformation #35

C	4.26586900	-0.01427200	-0.33654800
C	3.78630500	-0.36230600	0.90568700
C	2.40247200	-0.28583500	1.07519100
C	1.53082100	0.12709800	0.04056300
C	2.07218500	0.46802300	-1.20605200
C	3.43837400	0.39651700	-1.39301500
C	0.19324900	0.08166200	0.56550400
C	0.31123700	-0.35036200	1.85870200
N	1.63356700	-0.56835500	2.16899100
C	-1.06431400	0.44114600	-0.17868200
C	-1.52192600	1.90264300	0.05482700
C	-1.88864900	2.20372900	1.50604200
C	-0.46358000	2.88592100	-0.43864500
C	-2.17462200	-0.57696400	0.14076200
C	-3.45327600	-0.19480000	-0.58393100
C	-1.79798700	-1.99216900	-0.31387500
O	-3.58347300	-0.22649100	-1.78122000
O	-1.03479600	-2.17709200	-1.22927100
O	-4.41019600	0.20408300	0.25165200
C	-5.63026700	0.63774900	-0.36699800
C	-2.44590300	-3.11263500	0.44464900
Cl	5.99686100	-0.08780100	-0.62022300
H	4.44508300	-0.67859100	1.70403300
H	1.43051000	0.78535500	-2.01995500
H	3.88037100	0.65355400	-2.34657900
H	-0.45205700	-0.52745900	2.60146800
H	1.97655500	-0.89989700	3.05875900
H	-0.85373700	0.34031700	-1.24725400
H	-2.41727000	2.05377600	-0.56025200
H	-1.01291100	2.11526500	2.15375100
H	-2.26116000	3.22719900	1.58974400
H	-2.66517200	1.53714100	1.88628500
H	-0.83391200	3.91085400	-0.36517700
H	-0.19771200	2.69414000	-1.48091700
H	0.44595500	2.81514200	0.16367200
H	-2.38763000	-0.60487700	1.21000700
H	-6.28631500	0.92824600	0.44845100
H	-5.43651000	1.48722300	-1.02094300
H	-6.07183300	-0.17645600	-0.93982100
H	-3.52275100	-2.94094500	0.52295700
H	-2.24652900	-4.06837300	-0.03502500
H	-2.04677800	-3.11538600	1.46327200

Compound **1a**, optimization level SMD-M11/6-311++G(2d,p)

Conformation #1

C	4.22180400	-0.01175600	-0.35647900
C	3.76268600	-0.28138900	0.90983600
C	2.38143200	-0.19712400	1.09459800
C	1.49954300	0.14953800	0.04915800
C	2.01891400	0.40990200	-1.22458400
C	3.37995600	0.32858200	-1.42578800
C	0.16733800	0.13238300	0.59277400
C	0.30036100	-0.22331400	1.90622100
N	1.62541900	-0.41659600	2.21289000
C	-1.10175100	0.43340300	-0.15739900
C	-1.56945000	1.90576000	-0.02217000
C	-1.97526400	2.27934800	1.40302700
C	-0.48884300	2.85758600	-0.53373800
C	-2.21429700	-0.55137500	0.26542800
C	-3.45815600	-0.27896300	-0.55951700
C	-1.79048800	-2.01226800	0.06913400
O	-3.50010000	-0.33848800	-1.76571300
O	-2.00573700	-2.81825900	0.94668900
O	-4.50284400	0.05734300	0.19285000
C	-5.71405100	0.36443300	-0.52654300
C	-1.10716700	-2.38158600	-1.21509100
Cl	5.95185400	-0.09962600	-0.66157900
H	4.43785000	-0.54741600	1.71826600
H	1.35930500	0.67393200	-2.04934800
H	3.81108000	0.52499900	-2.40335000
H	-0.46254000	-0.36316100	2.66406500
H	1.98038600	-0.68878600	3.11995300
H	-0.90768600	0.26446800	-1.22624200
H	-2.44947400	2.01770700	-0.67346300
H	-1.13200800	2.14747300	2.09300200
H	-2.27509600	3.33322000	1.44162900
H	-2.81835500	1.68232000	1.76934000
H	-0.86221600	3.88815100	-0.54893600
H	-0.17056400	2.59298400	-1.54959900
H	0.39313500	2.82889800	0.11898400
H	-2.46250400	-0.43532100	1.32285500
H	-6.45349500	0.62415800	0.23136600
H	-5.53986000	1.20747700	-1.20092000
H	-6.03811700	-0.50921400	-1.09836100
H	-1.07118800	-3.46782300	-1.31738300
H	-1.62054500	-1.92321300	-2.06615900
H	-0.08350900	-1.98477200	-1.19290300

Compound **1a**, optimization level SMD-M11/6-311++G(2d,p)

Conformation #4

C	-3.81252700	-0.12864400	-0.03181000
C	-3.23778500	-0.55919000	1.13857500
C	-1.85303400	-0.73877200	1.11491300
C	-1.07513100	-0.48866000	-0.03648500
C	-1.71454500	-0.05684300	-1.20622800
C	-3.08074800	0.12198700	-1.20267000
C	0.29517400	-0.77547700	0.30368100
C	0.28151100	-1.17933100	1.60951100
N	-1.00268400	-1.16035600	2.09778100
C	1.47393500	-0.65876800	-0.63088000
C	2.30780600	-1.95755300	-0.70368500
C	1.45640800	-3.07168300	-1.31528500
C	3.58234600	-1.76659700	-1.52691400
C	2.32919700	0.60670800	-0.35971600
C	1.41249600	1.79408700	-0.13818000
C	3.29559000	0.53510700	0.81822100
O	1.34494800	2.43711700	0.88262600
O	3.19275800	-0.30248400	1.68505700
O	0.65294900	2.03116100	-1.20508500
C	-0.34654800	3.05492400	-1.04465200
C	4.40690500	1.54607300	0.80143500
Cl	-5.55363400	0.12159800	-0.07407400
H	-3.82908000	-0.74981600	2.02976600
H	-1.14146800	0.14500700	-2.10915600
H	-3.60058000	0.45733300	-2.09578800
H	1.10673400	-1.48056600	2.24134100
H	-1.26914500	-1.40318100	3.04271000
H	1.06075800	-0.50057600	-1.63772300
H	2.58408200	-2.24251300	0.31820400
H	0.53111200	-3.23096100	-0.74979000
H	1.18431300	-2.81814200	-2.34935600
H	2.01037200	-4.01754500	-1.33511800
H	4.08997000	-2.72761100	-1.67005900
H	4.29745900	-1.08615200	-1.04816500
H	3.34168000	-1.36250900	-2.52048000
H	2.92491400	0.83153000	-1.25475400
H	0.13352500	4.02069300	-0.86323700
H	-0.90790200	3.07599800	-1.97967500
H	-1.00402300	2.80244700	-0.20723500
H	4.03689900	2.52763600	0.48874800
H	4.88675200	1.60606900	1.78028700
H	5.14267300	1.21762100	0.05451200

Compound **1a**, optimization level SMD-M11/6-311++G(2d,p)

Conformation #6

C	4.14911900	-0.07361600	-0.46771200
C	3.75597700	-0.27881900	0.83238400
C	2.38801600	-0.16486800	1.08684300
C	1.45516300	0.14769600	0.07543700
C	1.90797000	0.34164900	-1.23503200
C	3.25500200	0.23066600	-1.50499500
C	0.15386200	0.17414100	0.68949800
C	0.35375800	-0.12407500	2.00903900
N	1.69118300	-0.32248400	2.25261500
C	-1.14919500	0.46310800	-0.00694700
C	-1.58950500	1.94683900	0.09236000
C	-1.94747300	2.37035600	1.51636600
C	-0.51221000	2.86495800	-0.48304500
C	-2.24462000	-0.48666100	0.52061500
C	-3.58663700	-0.17885500	-0.11832600
C	-1.90225100	-1.96819500	0.30089200
O	-4.58201300	0.11995300	0.49467700
O	-2.19357000	-2.77254700	1.15781000
O	-3.54396700	-0.26467600	-1.44848800
C	-4.77220100	0.04403300	-2.13581500
C	-1.20860600	-2.36765000	-0.96976600
Cl	5.85883300	-0.20086600	-0.86020700
H	4.47054100	-0.51865300	1.61467400
H	1.20802400	0.57760100	-2.03470800
H	3.63488500	0.37588500	-2.51233300
H	-0.36731800	-0.21712700	2.81357500
H	2.09151000	-0.55906600	3.15067100
H	-1.00780500	0.25160400	-1.07578700
H	-2.48767400	2.05086900	-0.53517500
H	-1.08959200	2.23577900	2.18768500
H	-2.22027400	3.43210900	1.53348200
H	-2.79492500	1.80406800	1.91980000
H	-0.87394700	3.89882800	-0.52722700
H	-0.22417700	2.55867700	-1.49630200
H	0.38674000	2.84951200	0.14656300
H	-2.38542400	-0.36084000	1.59670300
H	-4.56007900	-0.08125100	-3.19804400
H	-5.56047500	-0.64293600	-1.81635800
H	-5.06673400	1.07515200	-1.92086600
H	-1.68950600	-1.90312300	-1.83614000
H	-0.17273700	-2.00589400	-0.93428100
H	-1.21010000	-3.45516900	-1.06347900

Compound **1a**, optimization level SMD-M11/6-311++G(2d,p)

Conformation #16

C	4.18419900	-0.02377000	-0.47447700
C	3.78003900	-0.45193800	0.76651200
C	2.41084300	-0.37425800	1.02876700
C	1.48644700	0.11729100	0.08227100
C	1.95143900	0.53804100	-1.16946500
C	3.29964000	0.46659300	-1.44672400
C	0.18168300	0.04997000	0.68476100
C	0.37085100	-0.47162400	1.93396100
N	1.70612700	-0.72444300	2.14613400
C	-1.11291600	0.47368400	0.04288300
C	-1.54296800	1.91421200	0.41934100
C	-1.89481600	2.06464900	1.89915600
C	-0.46232100	2.91934300	0.02323300
C	-2.21216700	-0.55413400	0.35988000
C	-3.55986000	-0.09308600	-0.16904400
C	-1.89758600	-1.92770600	-0.25167600
O	-4.53465300	0.11388300	0.51242700
O	-1.18303600	-2.03488000	-1.21961200
O	-3.53932100	0.07523000	-1.49022600
C	-4.75929000	0.56609900	-2.07721000
C	-2.53215100	-3.10219800	0.43270800
Cl	5.89628700	-0.09370800	-0.87328400
H	4.48732300	-0.82998200	1.49934500
H	1.25945600	0.91748000	-1.91907400
H	3.68740900	0.78532600	-2.41013700
H	-0.35457400	-0.69150900	2.70935700
H	2.09874200	-1.11955000	2.99028300
H	-0.96358000	0.45101600	-1.04403000
H	-2.44213500	2.14156300	-0.17348700
H	-1.02425900	1.83942800	2.52824900
H	-2.19956200	3.09655100	2.11041400
H	-2.71859600	1.40672300	2.20022900
H	-0.81474800	3.94513700	0.18304100
H	-0.18479400	2.81281900	-1.03278400
H	0.44130100	2.77558900	0.62953600
H	-2.34111900	-0.68332400	1.43886800
H	-4.56502000	0.64818600	-3.14702100
H	-5.57437700	-0.13738900	-1.88683100
H	-5.00627400	1.54424500	-1.65442300
H	-3.59617500	-2.90546700	0.61210800
H	-2.40234900	-4.01210300	-0.15598800
H	-2.05674800	-3.22147200	1.41525900

Compound **1a**, optimization level SMD-M11/6-311++G(2d,p)

Conformation #1

C	4.25105700	-0.10775300	-0.37901100
C	3.76877800	-0.49539600	0.84723600
C	2.39199500	-0.36118200	1.03777000
C	1.53607600	0.14610900	0.03662300
C	2.07945600	0.52367800	-1.19719300
C	3.43605400	0.39573300	-1.40394500
C	0.20082900	0.13896100	0.57249100
C	0.30660500	-0.36622300	1.83841300
N	1.61897800	-0.66331800	2.12375600
C	-1.04704900	0.59263800	-0.13980400
C	-1.43991000	2.05643600	0.18704900
C	-1.88680800	2.24905100	1.63623600
C	-0.28939600	3.00691700	-0.14349600
C	-2.18923300	-0.38952300	0.14871700
C	-3.45107900	-0.02948200	-0.61556400
C	-1.81650000	-1.81568200	-0.29951200
O	-3.46575100	0.42643200	-1.73215400
O	-1.19089000	-1.99682500	-1.31674600
O	-4.55523600	-0.31893900	0.07470000
C	-5.80032600	-0.06322700	-0.60441000
C	-2.28536800	-2.92518900	0.59130500
Cl	5.97706300	-0.24885600	-0.68840100
H	4.42313600	-0.88503900	1.62195500
H	1.44148500	0.91291700	-1.98843500
H	3.88421800	0.67948200	-2.35194800
H	-0.46637000	-0.54522500	2.57766700
H	1.95246700	-1.06088300	2.99176000
H	-0.85292400	0.54060300	-1.21871100
H	-2.28314600	2.30607300	-0.46987300
H	-1.09009400	1.95737700	2.33278300
H	-2.11792800	3.30504000	1.82021100
H	-2.78547800	1.66893500	1.87863200
H	-0.61047600	4.04966700	-0.03534900
H	0.06579500	2.86323300	-1.17149800
H	0.55777500	2.84382700	0.53529300
H	-2.43469700	-0.42657400	1.21445200
H	-6.58624600	-0.36252900	0.08967400
H	-5.88187800	1.00096500	-0.84208400
H	-5.85140500	-0.65444900	-1.52284600
H	-3.35544000	-2.80293200	0.79902200
H	-2.08775900	-3.89941200	0.14049100
H	-1.76001600	-2.83783600	1.55151000

Compound **1a**, optimization level SMD-ωB97X-D/6-311++G(2d,p)

Conformation #1

C	4.21839400	-0.08764600	-0.36567900
C	3.76109000	-0.24892500	0.92218200
C	2.38527200	-0.11987300	1.10910900
C	1.49867100	0.16444700	0.04816800
C	2.01429500	0.31687600	-1.24343000
C	3.37289800	0.19021200	-1.44897100
C	0.17438900	0.21707100	0.60229400
C	0.31682900	-0.03558100	1.93778900
N	1.63837400	-0.23460900	2.24677700
C	-1.09225300	0.49237400	-0.15977800
C	-1.54867200	1.97319300	-0.09117400
C	-1.89671000	2.45109700	1.31617700
C	-0.50380300	2.88946800	-0.72332300
C	-2.21183600	-0.47578100	0.27764300
C	-3.45355300	-0.28409900	-0.57579000
C	-1.79707200	-1.95257100	0.18316700
O	-3.46266300	-0.29556600	-1.78262400
O	-2.05381800	-2.70188900	1.09788200
O	-4.54078300	-0.11546500	0.16930800
C	-5.77371500	0.06242100	-0.54281200
C	-1.08918200	-2.41533000	-1.05535100
Cl	5.94361000	-0.23834600	-0.67335700
H	4.43047800	-0.46645400	1.74473700
H	1.35858700	0.53389000	-2.07907300
H	3.79164600	0.30441600	-2.44072800
H	-0.43369300	-0.09818800	2.71097400
H	1.99478700	-0.44096100	3.16612800
H	-0.88920800	0.28867400	-1.21536700
H	-2.45465200	2.04687200	-0.70360300
H	-1.01864900	2.43042800	1.96767000
H	-2.25769900	3.48224200	1.27897000
H	-2.67885700	1.84715300	1.78226400
H	-0.87568000	3.91641800	-0.76927400
H	-0.25945000	2.57275400	-1.74091700
H	0.42120400	2.89601400	-0.13949600
H	-2.48435300	-0.30833300	1.31814000
H	-6.53573700	0.19110600	0.22187900
H	-5.72053700	0.94774400	-1.17676300
H	-5.99135500	-0.81644100	-1.15002400
H	-1.07449800	-3.50351300	-1.09023800
H	-1.56410800	-2.00700800	-1.94916200
H	-0.06035100	-2.04341800	-1.03435200

Compound **1a**, optimization level SMD-ωB97X-D/6-311++G(2d,p)

Conformation #6

C	4.13616400	-0.08484600	-0.48384400
C	3.74769900	-0.29383200	0.81964800
C	2.38407600	-0.17009100	1.08412600
C	1.44319900	0.15470000	0.08326700
C	1.88976300	0.35399400	-1.22755500
C	3.23510700	0.23370300	-1.50963100
C	0.15051500	0.18879700	0.70902400
C	0.36310200	-0.11363000	2.02475700
N	1.69868800	-0.32562800	2.25496600
C	-1.15469900	0.49290700	0.02609000
C	-1.59002500	1.97624700	0.15369700
C	-1.84736300	2.42376500	1.59013900
C	-0.57611700	2.89738000	-0.52024200
C	-2.25317900	-0.47541600	0.51310000
C	-3.59758400	-0.21712100	-0.14663800
C	-1.88976600	-1.95744900	0.31983500
O	-4.62786800	-0.06453500	0.46097300
O	-2.16714300	-2.74938000	1.19178400
O	-3.51268900	-0.19407900	-1.47390200
C	-4.73188400	0.04955900	-2.18744800
C	-1.20479300	-2.37841500	-0.94714400
Cl	5.84207100	-0.22440500	-0.88842200
H	4.45938100	-0.54238500	1.59666700
H	1.19100400	0.60247100	-2.01830100
H	3.60109400	0.38383100	-2.51734500
H	-0.34525900	-0.20253400	2.83442500
H	2.10331200	-0.56858900	3.14488900
H	-1.00994700	0.31313100	-1.04279100
H	-2.53065400	2.07312500	-0.40105700
H	-0.93207100	2.37545900	2.18666700
H	-2.19582300	3.45981100	1.59963900
H	-2.60828500	1.81740500	2.08714700
H	-0.94112100	3.92779800	-0.52322500
H	-0.39456100	2.59978700	-1.55659300
H	0.38156300	2.88477300	0.00807900
H	-2.42601700	-0.35294900	1.58099800
H	-4.46461700	0.01794400	-3.24086300
H	-5.46682800	-0.72267800	-1.95990800
H	-5.13101000	1.03056800	-1.92828500
H	-1.67570700	-1.92363800	-1.81986000
H	-0.16714100	-2.03259900	-0.92011100
H	-1.21703200	-3.46398900	-1.02968500

Compound **1a**, optimization level SMD-ωB97X-D/6-311++G(2d,p)

Conformation #35

C	4.24166100	-0.09754500	-0.39289600
C	3.76467400	-0.48649300	0.83785100
C	2.39027500	-0.35658200	1.03617100
C	1.52366400	0.14558600	0.04106200
C	2.05957200	0.52556700	-1.19416500
C	3.41696400	0.40327900	-1.41008800
C	0.19515500	0.13533800	0.58731000
C	0.31590900	-0.36292400	1.85392500
N	1.62850200	-0.65666500	2.12878300
C	-1.05594100	0.59150400	-0.11463100
C	-1.45563700	2.04995600	0.22820400
C	-1.81894100	2.26405900	1.69563800
C	-0.36285200	3.02666700	-0.20010900
C	-2.20033000	-0.40375100	0.13619600
C	-3.45321800	-0.04941000	-0.64499800
C	-1.80241400	-1.82939800	-0.29459200
O	-3.46585100	0.37736800	-1.77163600
O	-1.25574700	-2.02827800	-1.35262200
O	-4.55290200	-0.30672100	0.06126400
C	-5.80153000	-0.06190700	-0.60006900
C	-2.14712000	-2.92592000	0.66484600
Cl	5.96641200	-0.23378200	-0.71083100
H	4.41836600	-0.87211400	1.60991100
H	1.42008000	0.91494100	-1.97815200
H	3.85073400	0.69164500	-2.35915800
H	-0.44394100	-0.53945400	2.59990100
H	1.96815800	-1.04894900	2.99224300
H	-0.85515800	0.56610700	-1.18876900
H	-2.34334300	2.27470100	-0.37166900
H	-0.96225900	2.07267500	2.34805400
H	-2.13125600	3.29966700	1.85447500
H	-2.64172200	1.62139300	2.01873700
H	-0.69783600	4.05807900	-0.06109300
H	-0.10259100	2.89523600	-1.25400700
H	0.54667300	2.88934900	0.39201800
H	-2.46441400	-0.43680300	1.19272000
H	-6.57090700	-0.33792300	0.11699800
H	-5.89138100	0.99236200	-0.86277000
H	-5.88333200	-0.67566100	-1.49741400
H	-3.21231900	-2.87966300	0.90933900
H	-1.89589100	-3.90064900	0.24964300
H	-1.59953400	-2.76108500	1.59766300

Compound **1b**, optimization level PCM-B3LYP/6-311++G(2d,p)

Conformation #1

C	-3.83532100	-0.73060800	-0.26632600
C	-2.49506300	-1.08387400	-0.10391800
C	-1.49344500	-0.14225900	0.25441800
C	-1.86862600	1.19442900	0.45148900
C	-3.19459100	1.56274900	0.29386300
C	-4.15004700	0.59889200	-0.06093400
C	-0.24476900	-0.85505700	0.33855500
C	-0.53287800	-2.15994700	0.03174800
N	-1.87908800	-2.30217400	-0.23299800
C	1.10403700	-0.24958100	0.65968000
C	1.92254200	-1.12371600	1.65133900
C	1.18857000	-1.19780500	2.99917100
C	3.35614700	-0.63271700	1.87983700
C	1.78671200	0.16868600	-0.68839100
C	2.21751400	-0.98525900	-1.60865400
C	2.94069900	1.16012000	-0.57764000
O	2.73001200	-1.99377600	-1.17158900
O	3.99820900	1.05567300	-1.15940400
O	2.61864200	2.20542600	0.19135000
C	3.62196300	3.23728700	0.32718400
C	1.95439400	-0.79495600	-3.07883400
H	-4.59125900	-1.45538700	-0.53763600
H	-1.13621500	1.94426900	0.72586000
H	-3.50086000	2.58936600	0.44237700
H	0.11642900	-3.01868200	-0.01734100
H	-2.33255900	-3.16962400	-0.47025800
H	0.91328200	0.69897500	1.17000600
H	1.98596100	-2.13277600	1.23984800
H	1.13774000	-0.20957200	3.46762600
H	0.16944600	-1.57141800	2.89086800
H	1.71955000	-1.86248300	3.68484900
H	3.37286400	0.39430000	2.25565800
H	3.84620500	-1.26120800	2.62760700
H	3.95887100	-0.68190100	0.97291100
H	1.02042400	0.73483600	-1.22908600
H	4.52612200	2.82489700	0.77201300
H	3.85164700	3.66721400	-0.64629900
H	3.17970900	3.98313100	0.98088800
H	2.41596200	-1.59382400	-3.65608600
H	0.87287000	-0.79778100	-3.24863400
H	2.32661300	0.17628300	-3.41284400
Cl	-5.83183200	1.11292300	-0.25174800

Compound **1b**, optimization level PCM-B3LYP/6-311++G(2d,p)

Conformation #2

C	-3.50957700	-0.00470200	0.99758200
C	-2.13888300	0.21652900	1.13936600
C	-1.26244200	0.33921700	0.02854800
C	-1.79938900	0.22738300	-1.26188600
C	-3.15722000	0.00633700	-1.42202500
C	-3.98547800	-0.10406000	-0.29520200
C	0.06446900	0.55480100	0.54897200
C	-0.06355900	0.54896700	1.91569000
N	-1.37942600	0.34987600	2.27337100
C	1.32259900	0.74290600	-0.26650800
C	1.77819600	2.23203600	-0.39404800
C	2.10966000	2.91641100	0.93719000
C	0.74476600	3.05456700	-1.17266300
C	2.47634600	-0.16219800	0.23788400
C	3.67797200	-0.20203100	-0.73531100
C	2.04456500	-1.60953900	0.47145800
O	3.51977000	-0.12447200	-1.93427200
O	2.29151000	-2.23368100	1.47940200
O	1.39767100	-2.11788300	-0.58009000
C	0.95592700	-3.48880900	-0.46412100
C	5.03618000	-0.35656900	-0.10660800
H	-4.16675200	-0.09619800	1.85199300
H	-1.16765100	0.31164500	-2.13765600
H	-3.58607900	-0.08225400	-2.41099800
H	0.68422100	0.66558500	2.68323000
H	-1.71923100	0.29785800	3.22010300
H	1.10482700	0.40733100	-1.28196100
H	2.69256800	2.21887700	-0.99520200
H	1.22295800	3.01070500	1.56814700
H	2.87525600	2.38509300	1.50721800
H	2.48950400	3.92358200	0.74942700
H	-0.20182100	3.11990900	-0.63034300
H	1.10918300	4.07271700	-1.32966600
H	0.54254400	2.61705400	-2.15344400
H	2.84307300	0.17451300	1.20658900
H	1.81090000	-4.14700200	-0.31816100
H	0.26436500	-3.58972000	0.37065400
H	0.45735600	-3.71059900	-1.40290700
H	5.79329800	-0.52846500	-0.86906800
H	5.27634300	0.55463600	0.45024000
H	5.03314200	-1.17518700	0.61752600
Cl	-5.71402500	-0.38461200	-0.54585500

Compound **1b**, optimization level PCM-B3LYP/6-311++G(2d,p)

Conformation #3

C	-3.89512800	0.41627700	0.66859000
C	-2.55994700	0.82179100	0.65449900
C	-1.58396500	0.21356400	-0.17960800
C	-1.97994900	-0.83685600	-1.02006200
C	-3.30120400	-1.25275200	-1.01823700
C	-4.23111600	-0.62248400	-0.17801900
C	-0.33388900	0.88699700	0.06139300
C	-0.59612000	1.84376400	1.00783700
N	-1.92793000	1.80913600	1.36561100
C	0.99392200	0.55327300	-0.58092100
C	1.77900700	1.82450700	-1.01001600
C	0.99086500	2.57634900	-2.09382100
C	3.19768600	1.54371600	-1.51761800
C	1.73449800	-0.48966300	0.32291000
C	2.24337400	0.03607900	1.67377000
C	2.79366000	-1.29118200	-0.43282000
O	2.79665300	1.11147600	1.77636800
O	2.61600500	-1.76052800	-1.53221700
O	3.91233200	-1.47074600	0.28005700
C	4.94469400	-2.28032700	-0.32885600
C	1.98978100	-0.84500100	2.86949600
H	-4.63112900	0.88766200	1.30614200
H	-1.26746800	-1.32807800	-1.67199600
H	-3.62346600	-2.06104600	-1.66057800
H	0.06198000	2.56585900	1.46285300
H	-2.36270900	2.42392300	2.03442800
H	0.77816000	-0.00357500	-1.49818900
H	1.86900600	2.47598900	-0.13849300
H	0.90829400	1.97142200	-3.00250800
H	-0.01807800	2.82940600	-1.76534200
H	1.50120400	3.50531700	-2.35936500
H	3.18926800	0.87355200	-2.38107500
H	3.66781700	2.47925500	-1.83106100
H	3.83325000	1.10852200	-0.74548900
H	0.98358600	-1.25394700	0.55042800
H	5.28136800	-1.81998200	-1.25608800
H	5.75055500	-2.31164300	0.39820400
H	4.56624400	-3.28147700	-0.52769000
H	0.91275600	-0.87381300	3.06461800
H	2.30372700	-1.87159600	2.66826600
H	2.50700600	-0.45746600	3.74520400
Cl	-5.90781100	-1.18542300	-0.20801800

Compound **1b**, optimization level PCM-B3LYP/6-311++G(2d,p)

Conformation #5

C	3.54330700	0.34709200	0.90119800
C	2.17485800	0.14627300	1.08689000
C	1.31742700	-0.30393600	0.04829800
C	1.87116000	-0.54984900	-1.21621400
C	3.22691700	-0.35401500	-1.41968600
C	4.03652700	0.08721300	-0.36238000
C	-0.01231700	-0.39114600	0.59745400
C	0.09484200	0.00470900	1.90749000
N	1.40139500	0.32343700	2.20539900
C	-1.25572600	-0.82915300	-0.14134900
C	-1.68416600	-2.30145300	0.14896000
C	-2.04872300	-2.57947400	1.61160300
C	-0.61742400	-3.28963000	-0.33645900
C	-2.42466600	0.17153500	0.09665300
C	-3.71087200	-0.19006800	-0.67571000
C	-2.00480800	1.58692000	-0.28483900
O	-4.74704900	-0.34535800	-0.06122500
O	-1.45654800	1.89183700	-1.31947100
O	-2.35055400	2.46762100	0.66213400
C	-2.02879800	3.85253300	0.39738200
C	-3.64616100	-0.33336800	-2.17378000
H	4.18579000	0.68903100	1.70150100
H	1.25441300	-0.89074500	-2.03879200
H	3.66859300	-0.53879600	-2.38951100
H	-0.66225000	0.09503200	2.66932200
H	1.72637800	0.65191600	3.10039700
H	-1.02035500	-0.78047900	-1.20727100
H	-2.58353200	-2.48672700	-0.44938200
H	-2.40404600	-3.60745000	1.71414600
H	-1.18149000	-2.46320000	2.26537200
H	-2.84132500	-1.92378400	1.97792400
H	0.31156000	-3.17894400	0.22800000
H	-0.96558500	-4.31711800	-0.20738100
H	-0.38875000	-3.14460200	-1.39519000
H	-2.71029600	0.18289600	1.14624100
H	-0.95298900	3.97051700	0.27937200
H	-2.53914400	4.19008600	-0.50316800
H	-2.38133700	4.40036400	1.26609200
H	-3.15155100	0.52938600	-2.62255300
H	-3.05175500	-1.21113800	-2.44057400
H	-4.65134600	-0.44910400	-2.57382400
Cl	5.76318800	0.32123000	-0.66606300

Compound **1b**, optimization level PCM-B3LYP/6-311++G(2d,p)

Conformation #7

C	-3.46499200	-0.19170700	0.87940200
C	-2.12593800	-0.44576500	1.17892400
C	-1.09451500	-0.43813000	0.19817800
C	-1.45917000	-0.15294700	-1.12835100
C	-2.78218000	0.10613600	-1.44370800
C	-3.76083700	0.08143800	-0.44046600
C	0.14282900	-0.73543100	0.88831800
C	-0.19417900	-0.89516900	2.20820100
N	-1.54650200	-0.73240500	2.38800500
C	1.56407900	-0.85238800	0.38297700
C	1.89767300	-2.18972200	-0.34676700
C	1.11808500	-2.43709700	-1.64273600
C	1.74688700	-3.38091300	0.60641500
C	1.97926600	0.40040700	-0.45025800
C	3.42923200	0.32672000	-0.97269700
C	1.80438400	1.66695700	0.37899500
O	3.62485300	0.35360800	-2.17135100
O	2.24964900	1.83209000	1.49191100
O	1.11076700	2.59534600	-0.29069000
C	0.89475000	3.85133000	0.39270300
C	4.56500400	0.23745200	0.01239100
H	-4.23237000	-0.20623900	1.64189100
H	-0.72517700	-0.13489400	-1.92201800
H	-3.06678400	0.32650100	-2.46362600
H	0.44433100	-1.11376100	3.04954400
H	-2.02647500	-0.79190300	3.27152000
H	2.19325700	-0.83838600	1.27672500
H	2.95917000	-2.13484500	-0.61474900
H	0.05131600	-2.56204100	-1.44744400
H	1.24201200	-1.62791600	-2.36507600
H	1.47569900	-3.35335600	-2.11859700
H	0.70433900	-3.52453800	0.89975200
H	2.08807800	-4.29879500	0.12192300
H	2.33620200	-3.24268100	1.51661900
H	1.36049400	0.49540800	-1.33842000
H	0.33478000	3.68575100	1.31155500
H	1.84935100	4.32265500	0.62078300
H	0.32218200	4.46044600	-0.30023400
H	4.42171200	-0.59243600	0.70684900
H	5.50353400	0.11365700	-0.52377800
H	4.59900600	1.14635500	0.61713700
Cl	-5.43844500	0.41426400	-0.88849800

Compound **1b**, optimization level PCM-B3LYP/6-311++G(2d,p)

Conformation #8

C	3.48901900	-0.08626300	0.85549000
C	2.15465400	0.15560400	1.18513400
C	1.13485600	0.34277500	0.21027500
C	1.50522300	0.27056500	-1.14275100
C	2.82351000	0.02822300	-1.48929100
C	3.79134600	-0.14376300	-0.48971800
C	-0.10223300	0.55771300	0.93010600
C	0.22349000	0.48572600	2.26041600
N	1.56904700	0.25390400	2.42076400
C	-1.50979200	0.81429800	0.43759300
C	-1.77765800	2.28154100	-0.02753400
C	-0.92390400	2.75913600	-1.20761500
C	-1.65009200	3.25447600	1.15048000
C	-1.96954000	-0.21929400	-0.62563900
C	-3.48816900	-0.14534500	-0.91047400
C	-1.65794600	-1.66213800	-0.22757100
O	-4.28143300	0.13627100	-0.03894300
O	-1.06315800	-2.44503100	-0.93385700
O	-2.14715300	-1.96841400	0.97664500
C	-1.91161800	-3.31517100	1.44368100
C	-3.91239100	-0.45625700	-2.32032300
H	4.24792200	-0.22352000	1.61425200
H	0.77785900	0.40350700	-1.93165700
H	3.11292500	-0.02869800	-2.52988000
H	-0.41832200	0.58116400	3.12200300
H	2.04017400	0.15353800	3.30530300
H	-2.16578300	0.65842300	1.29634100
H	-2.82385300	2.30922500	-0.34715100
H	-1.23844000	3.76163300	-1.50805500
H	0.13369400	2.81116900	-0.94138300
H	-1.02219200	2.11609800	-2.08504700
H	-0.62273500	3.29900100	1.52023400
H	-1.93878300	4.26266900	0.84344100
H	-2.29447500	2.95980800	1.98284100
H	-1.44801400	-0.07462400	-1.56983600
H	-0.84189800	-3.50308800	1.51996600
H	-2.37979900	-3.36641500	2.42214600
H	-2.36559600	-4.03320000	0.76257700
H	-3.55891000	0.34101900	-2.98180000
H	-3.44578400	-1.38160100	-2.66754300
H	-4.99597600	-0.53123600	-2.38612700
Cl	5.46388700	-0.44739800	-0.97707700

Compound **1b**, optimization level PCM-B3LYP/6-311++G(2d,p)

Conformation #9

C	3.52654600	-0.12614400	0.98561900
C	2.15444200	-0.34023800	1.12413500
C	1.26209600	-0.33923500	0.01934700
C	1.78436200	-0.10770800	-1.26120400
C	3.14319500	0.10898000	-1.41764500
C	3.98752900	0.09474400	-0.29742700
C	-0.06064800	-0.58684800	0.53618200
C	0.08488800	-0.71789000	1.89497400
N	1.40781600	-0.57521200	2.25026000
C	-1.33353700	-0.67479700	-0.27330700
C	-1.81435700	-2.13264400	-0.55518300
C	-2.18103600	-2.93358900	0.69898400
C	-0.78766700	-2.89586700	-1.39979500
C	-2.45801800	0.19136700	0.37139200
C	-3.78644300	0.14669800	-0.41229800
C	-2.03216200	1.63896200	0.59377400
O	-4.78015900	-0.28417500	0.13746300
O	-2.23834100	2.24375900	1.62220900
O	-1.43698800	2.17447700	-0.47642000
C	-1.02125000	3.55412400	-0.36097800
C	-3.83052000	0.65629600	-1.82979900
H	4.19585400	-0.13083300	1.83541800
H	1.14125800	-0.09591100	-2.13267000
H	3.56071700	0.28920600	-2.39896900
H	-0.65452000	-0.89820200	2.65825600
H	1.75999300	-0.61646500	3.19304000
H	-1.11608000	-0.23350400	-1.24811600
H	-2.72225000	-2.04517900	-1.16366700
H	-1.30762600	-3.09416400	1.33505500
H	-2.95286500	-2.44508000	1.29694900
H	-2.56484200	-3.91514800	0.41129500
H	0.14700800	-3.04062500	-0.85272000
H	-1.17442900	-3.88239600	-1.66590600
H	-0.55762400	-2.36587700	-2.32735000
H	-2.70187500	-0.18820400	1.36130800
H	-0.29505400	3.65899600	0.44321100
H	-0.57084000	3.79975100	-1.31799000
H	-1.88322300	4.19026500	-0.16672600
H	-4.76598400	0.35355500	-2.29592000
H	-3.77251800	1.74821100	-1.81903400
H	-2.98352800	0.30233800	-2.41848400
Cl	5.71665200	0.37329400	-0.54385000

Compound **1b**, optimization level PCM-B3LYP/6-311++G(2d,p)

Conformation #14

C	-3.52135200	-0.33541300	1.15791100
C	-2.14966700	-0.07965900	1.18562500
C	-1.41377000	0.28835500	0.02755200
C	-2.09928000	0.40305600	-1.19059000
C	-3.46111200	0.15530600	-1.23662900
C	-4.14567400	-0.20903900	-0.06763600
C	-0.04137600	0.47115400	0.43013700
C	-0.00722900	0.21649100	1.77796500
N	-1.26539500	-0.11346800	2.23310500
C	1.08893800	0.90951500	-0.47607300
C	1.73048100	2.25889300	-0.03842000
C	0.67047900	3.36844800	-0.01057100
C	2.89391100	2.68450600	-0.94418000
C	2.14006200	-0.20930900	-0.77726300
C	1.41494700	-1.48050600	-1.27187300
C	3.14367600	-0.49714400	0.32689000
O	0.92738200	-1.47024100	-2.38319000
O	3.02335200	-0.24119700	1.50249300
O	4.22971100	-1.10053000	-0.18670000
C	5.26753600	-1.46516700	0.75001100
C	1.31981500	-2.68050800	-0.37041300
H	-4.07026800	-0.61647900	2.04672900
H	-1.57842000	0.67956400	-2.09918700
H	-4.00283600	0.23879300	-2.16905600
H	0.82851800	0.23806600	2.45571600
H	-1.49002000	-0.35114100	3.18588500
H	0.64015300	1.09390900	-1.45709800
H	2.11600900	2.13206100	0.97671100
H	0.26906700	3.54836100	-1.01293200
H	-0.16346400	3.11869600	0.64647200
H	1.11016200	4.30370100	0.34376500
H	2.57731000	2.74361700	-1.99056100
H	3.25188300	3.67439300	-0.65218400
H	3.74767600	2.00648200	-0.88913700
H	2.72368700	0.11228600	-1.64085400
H	4.87845600	-2.16451500	1.48858600
H	6.04397000	-1.93293300	0.15204500
H	5.65085900	-0.57680400	1.24927000
H	0.65596500	-3.42336100	-0.80764300
H	2.31436700	-3.11717100	-0.24047600
H	0.96256400	-2.38745400	0.61883900
Cl	-5.88433900	-0.51905300	-0.17247700

Compound **1b**, optimization level PCM-B3LYP/6-311++G(2d,p)

Conformation #15

C	-3.46056000	-0.35742600	0.80230300
C	-2.13068700	-0.67518100	1.08165100
C	-1.07390800	-0.48769300	0.14649200
C	-1.40239300	0.04843700	-1.10975900
C	-2.71586200	0.37285500	-1.40381700
C	-3.72043900	0.16485500	-0.44837600
C	0.14516600	-0.91903800	0.79585300
C	-0.22558900	-1.32732600	2.05108100
N	-1.58266500	-1.19299200	2.22640800
C	1.57666600	-0.94816200	0.30750900
C	1.92163400	-2.15738400	-0.61864200
C	1.11155100	-2.23350400	-1.91786500
C	1.81662700	-3.47845900	0.15223300
C	2.02425100	0.40075000	-0.31539500
C	3.55198700	0.45899800	-0.55382600
C	1.69978700	1.57625000	0.60461200
O	4.32685700	-0.02027100	0.24492100
O	1.95224400	1.61469500	1.78551000
O	1.12975300	2.58317400	-0.07135600
C	0.81052300	3.76823400	0.69335600
C	4.00759000	1.15377200	-1.80786700
H	-4.24826500	-0.50974400	1.52816900
H	-0.64610800	0.21315800	-1.86482800
H	-2.97326800	0.78616600	-2.36960300
H	0.39118200	-1.70552000	2.85095300
H	-2.08440500	-1.41827400	3.07015400
H	2.19871300	-1.07203100	1.19665000
H	2.97402700	-2.03705400	-0.89489800
H	1.19599800	-1.32612000	-2.51986600
H	1.47506600	-3.06122900	-2.53174000
H	0.05272700	-2.41063000	-1.71832200
H	0.78577300	-3.68302400	0.45161900
H	2.15488100	-4.30990500	-0.47094700
H	2.43248000	-3.46350900	1.05517800
H	1.52918300	0.58756800	-1.26613500
H	1.71509300	4.18618900	1.13215700
H	0.37412200	4.46140000	-0.01951500
H	0.09647400	3.52544700	1.47848400
H	5.09044400	1.26095900	-1.81358000
H	3.69139100	0.56762300	-2.67623400
H	3.52862300	2.13202900	-1.89842400
Cl	-5.38496300	0.59098800	-0.86670500

Compound **1b**, optimization level PCM-B3LYP/6-311++G(2d,p)

Conformation #17

C	3.20311100	0.13784300	1.18413200
C	1.84855800	0.43387200	1.33794100
C	0.94273000	0.51428700	0.24320800
C	1.45250100	0.28870300	-1.04576500
C	2.79576300	-0.00404300	-1.21793200
C	3.64563200	-0.07596800	-0.10612600
C	-0.35403200	0.84414200	0.79203300
C	-0.17214400	0.94671100	2.14623600
N	1.13977600	0.70009500	2.48086300
C	-1.67946600	1.07897600	0.10006900
C	-1.58892500	2.05122400	-1.11327700
C	-0.88562300	3.35450200	-0.70784800
C	-2.96714600	2.38861200	-1.70175100
C	-2.45253100	-0.24264600	-0.22392400
C	-3.16319700	-0.85010000	0.99745800
C	-1.62659700	-1.25148300	-1.00558800
O	-3.19425200	-0.27604600	2.06551000
O	-1.50190100	-1.23231900	-2.21049700
O	-1.03301400	-2.15074200	-0.21297100
C	-0.20989800	-3.14592100	-0.86201200
C	-3.90390000	-2.15236200	0.79387900
H	3.87412700	0.08088500	2.03077200
H	0.81217400	0.33185300	-1.91624700
H	3.19405200	-0.17915200	-2.20819600
H	-0.89641200	1.17631100	2.91048800
H	1.51447900	0.71569300	3.41555100
H	-2.31733800	1.57447700	0.83709200
H	-0.99854600	1.57939500	-1.90372900
H	-0.82256800	4.03164200	-1.56275000
H	-1.44581300	3.86611500	0.08141700
H	0.12590100	3.18054800	-0.34091000
H	-3.63757500	2.78163600	-0.93084100
H	-2.86487800	3.15573500	-2.47259400
H	-3.45512700	1.53279300	-2.17094000
H	-3.27666500	0.00994200	-0.89642300
H	0.62327200	-2.66489200	-1.37110000
H	0.14913500	-3.78744100	-0.06276100
H	-0.80371600	-3.71538700	-1.57511300
H	-4.77356500	-2.17779000	1.44930600
H	-4.21144800	-2.29767300	-0.24253200
H	-3.24310700	-2.97896000	1.06512900
Cl	5.35291100	-0.45539000	-0.37073000

Compound **1b**, optimization level PCM-B3LYP/6-311++G(2d,p)

Conformation #20

C	3.63523900	0.27849900	0.87974400
C	2.25850100	0.16648600	1.07817400
C	1.37710400	-0.31315400	0.07345000
C	1.91329600	-0.68920100	-1.16606700
C	3.27795800	-0.58556900	-1.38047400
C	4.11112300	-0.10620300	-0.35885900
C	0.04430800	-0.28313800	0.62038800
C	0.17282300	0.19665000	1.89882500
N	1.49676000	0.46791800	2.17752400
C	-1.19997200	-0.75063500	-0.09789100
C	-1.42031000	-2.28277500	0.14676700
C	-2.37377500	-2.94699200	-0.85497100
C	-1.82102100	-2.64093000	1.58296200
C	-2.43735500	0.13075400	0.22429400
C	-3.57949100	-0.00156200	-0.81244500
C	-2.11270600	1.62481800	0.28901800
O	-3.34482600	-0.08892300	-1.99740200
O	-2.46037900	2.34772900	1.19672500
O	-1.44824900	2.04803900	-0.78781300
C	-1.10785100	3.45154400	-0.82342000
C	-4.98081100	0.04256300	-0.26454200
H	4.29747900	0.64724200	1.65155900
H	1.27594800	-1.06007100	-1.95981300
H	3.70799600	-0.87119400	-2.33088300
H	-0.57721900	0.37895900	2.65115300
H	1.83951300	0.84156600	3.04761000
H	-1.01705500	-0.63096000	-1.16816800
H	-0.43172900	-2.72054600	-0.02771500
H	-2.11900100	-2.69424000	-1.88587100
H	-3.41579700	-2.66441200	-0.68709700
H	-2.31881100	-4.03335900	-0.74861200
H	-1.14550900	-2.20043400	2.31805100
H	-2.83884400	-2.31718000	1.81725000
H	-1.79213900	-3.72508600	1.71539900
H	-2.84074300	-0.10662100	1.20733600
H	-2.01153500	4.05807700	-0.79207300
H	-0.46496500	3.70187500	0.01898900
H	-0.58164300	3.59613300	-1.76222500
H	-5.70741500	0.05371500	-1.07442700
H	-5.15332600	-0.82725000	0.37571700
H	-5.10780400	0.92560200	0.36784000
Cl	5.84768000	0.01006300	-0.67520500

Compound **1b**, optimization level PCM-B3LYP/6-311++G(2d,p)

Conformation #21

C	3.54463900	0.44365300	0.89307700
C	2.17755900	0.22620700	1.07081400
C	1.34302000	-0.29057200	0.04441700
C	1.91845400	-0.58613400	-1.19955200
C	3.27315600	-0.37485200	-1.39502200
C	4.05982400	0.13204200	-0.34992600
C	0.00722400	-0.37706600	0.57857900
C	0.08888100	0.08361700	1.86897400
N	1.38525300	0.44318600	2.16878700
C	-1.21691800	-0.87599700	-0.15353800
C	-1.60694600	-2.34716700	0.19642400
C	-1.93079400	-2.58607100	1.67587200
C	-0.52730200	-3.32738500	-0.27712100
C	-2.41597100	0.09104100	0.01653200
C	-3.60741500	-0.26410400	-0.90294700
C	-2.01981000	1.52334900	-0.33787200
O	-3.42514400	-0.68298800	-2.02544300
O	-1.40981800	1.84113200	-1.33132400
O	-2.46802900	2.39600400	0.57511700
C	-2.18431700	3.79109100	0.32015900
C	-4.98271100	-0.04075200	-0.33533400
H	4.16993700	0.83641000	1.68365900
H	1.31859800	-0.97764800	-2.01198200
H	3.73143500	-0.59759300	-2.34905600
H	-0.68032500	0.19470200	2.61593600
H	1.69195700	0.81721700	3.05220700
H	-0.98400500	-0.86792100	-1.22017500
H	-2.51082600	-2.56812600	-0.37967200
H	-1.04769100	-2.44682000	2.30344600
H	-2.71634700	-1.92491100	2.04870000
H	-2.27823600	-3.61231900	1.81713500
H	0.41226600	-3.17390200	0.25953700
H	-0.84595900	-4.35783700	-0.10183300
H	-0.32799300	-3.21632700	-1.34584100
H	-2.77340000	0.10126700	1.04518800
H	-2.62645900	4.33101300	1.15210900
H	-1.10835600	3.95346100	0.28558300
H	-2.63542200	4.09932500	-0.62161900
H	-5.74040500	-0.18195200	-1.10351700
H	-5.15238700	-0.74849000	0.48206200
H	-5.06060500	0.96076800	0.09513000
Cl	5.78635000	0.38345800	-0.64214800

Compound **1b**, optimization level PCM-B3LYP/6-311++G(2d,p)

Conformation #22

C	3.26016900	-0.84107200	-0.91254900
C	1.91141600	-1.18924500	-0.83596400
C	0.99653900	-0.55759000	0.05237200
C	1.49199000	0.45336500	0.89133500
C	2.82903700	0.81080300	0.83149700
C	3.68772500	0.16283100	-0.06661400
C	-0.29046700	-1.18405100	-0.15111200
C	-0.09453200	-2.13611300	-1.11751100
N	1.21678100	-2.14274400	-1.53439000
C	-1.62263300	-0.92722400	0.51967000
C	-1.54328400	-0.83414000	2.07142500
C	-0.82219800	-2.06189300	2.64623800
C	-2.92893800	-0.71669400	2.72382000
C	-2.42284900	0.24538400	-0.14130800
C	-3.14290500	-0.17391200	-1.43704200
C	-1.60946400	1.51109200	-0.34885800
O	-3.20233300	-1.33647700	-1.77784400
O	-1.03601600	1.80951900	-1.37159700
O	-1.59214300	2.27006400	0.75776500
C	-0.84521400	3.50533500	0.67613400
C	-3.84833000	0.90543700	-2.22171300
H	3.93813600	-1.33227800	-1.59783900
H	0.84646200	0.96521100	1.59210800
H	3.21608500	1.58903300	1.47523200
H	-0.80859500	-2.81750200	-1.55036000
H	1.60130000	-2.75837200	-2.23250900
H	-2.23836400	-1.80453300	0.30486800
H	-0.97172100	0.05549100	2.34919200
H	-1.36575400	-2.97935200	2.39876100
H	0.19337200	-2.16120900	2.26285500
H	-0.76631300	-1.99240900	3.73500100
H	-2.83443000	-0.76938700	3.81075500
H	-3.43232400	0.22427200	2.49586300
H	-3.58192400	-1.53656200	2.40824100
H	-3.24294600	0.51361600	0.53086100
H	-1.27863700	4.15264300	-0.08462500
H	-0.93137500	3.95911400	1.65897800
H	0.19690300	3.30154900	0.43630800
H	-4.63469500	0.45908100	-2.82792400
H	-4.26592100	1.67403800	-1.56868300
H	-3.12522800	1.39341700	-2.87910300
Cl	5.38682700	0.65203700	-0.11310400

Compound **1b**, optimization level PCM-B3LYP/6-311++G(2d,p)

Conformation #23

C	-3.51567400	0.06772400	0.90398200
C	-2.15459500	0.27727300	1.13033800
C	-1.21507800	0.42072700	0.07452800
C	-1.67749100	0.35129700	-1.24725100
C	-3.02525400	0.14521300	-1.49075500
C	-3.91684700	0.00701800	-0.41635200
C	0.07761900	0.62054000	0.67545500
C	-0.12728000	0.59217500	2.03148300
N	-1.46188400	0.38613000	2.30902300
C	1.38578800	0.85087800	-0.04729700
C	1.93299000	2.28257100	0.24679500
C	0.92700300	3.34950800	-0.20618900
C	3.30182000	2.58231700	-0.37627800
C	2.43989000	-0.25059600	0.28111500
C	3.47249400	-0.47671400	-0.84685300
C	1.81229500	-1.61317400	0.57692400
O	3.20334600	-0.25587400	-2.00702500
O	1.94795300	-2.21117300	1.62045700
O	1.12560600	-2.08334000	-0.46794300
C	0.47601800	-3.36146700	-0.29094200
C	4.81113400	-1.02267500	-0.42093700
H	-4.22155500	-0.04131200	1.71650800
H	-0.99487800	0.45485500	-2.08202500
H	-3.39778500	0.08879400	-2.50461300
H	0.57712700	0.70245300	2.84052300
H	-1.85730500	0.31816500	3.23279300
H	1.19349300	0.77979100	-1.12027000
H	2.04057900	2.36142900	1.33537800
H	1.28899300	4.34701500	0.05364200
H	0.79269900	3.31735400	-1.29184700
H	-0.05010800	3.21436300	0.25856400
H	3.27354500	2.49414900	-1.46475300
H	3.59946600	3.60461500	-0.13113800
H	4.09119700	1.92772500	-0.00219100
H	2.98056600	0.00544900	1.19350600
H	-0.25070000	-3.30242100	0.51763500
H	-0.01908500	-3.56604400	-1.23545700
H	1.21446300	-4.13035400	-0.06963600
H	5.40892100	-1.28315900	-1.29200800
H	5.33969800	-0.27255300	0.17418900
H	4.68197300	-1.89734900	0.22173800
Cl	-5.62955000	-0.25638100	-0.77318800

Compound **1b**, optimization level PCM-B3LYP/6-311++G(2d,p)

Conformation #37

C	3.56151700	-0.61208400	-1.08025400
C	2.17866300	-0.42784300	-1.10164000
C	1.47072200	0.20004400	-0.04203600
C	2.19828900	0.65805500	1.06618800
C	3.57209600	0.48366300	1.10492700
C	4.22732200	-0.14601700	0.03683600
C	0.07446500	0.21099500	-0.40514100
C	0.00162200	-0.38952600	-1.63695800
N	1.25679500	-0.77167500	-2.05638600
C	-1.04097800	0.80883900	0.42450400
C	-1.55664500	2.17271100	-0.12921200
C	-0.40429300	3.17286200	-0.28727600
C	-2.65042500	2.78784500	0.75500900
C	-2.20635800	-0.18538800	0.75043500
C	-1.69613200	-1.56565800	1.19294400
C	-3.25035600	-0.37460600	-0.34062300
O	-2.00028600	-2.57444600	0.58916700
O	-3.06303600	-0.31786100	-1.53345100
O	-4.44917500	-0.63057500	0.20491100
C	-5.53075900	-0.89195900	-0.71577600
C	-0.82746300	-1.61081600	2.42403300
H	4.08826500	-1.09290500	-1.89370600
H	1.70425600	1.15073100	1.89507000
H	4.14541900	0.83141200	1.95356200
H	-0.86274000	-0.57921200	-2.24938200
H	1.45518000	-1.24964000	-2.92050700
H	-0.60568100	1.03556400	1.40312900
H	-1.97755400	1.98621800	-1.12094600
H	0.05139100	3.40295600	0.68082900
H	0.37755900	2.79463400	-0.94653100
H	-0.77584900	4.10961900	-0.70910900
H	-2.29367700	2.92504800	1.78096600
H	-2.93343400	3.77038800	0.37069400
H	-3.55944400	2.18498200	0.79354800
H	-2.73674900	0.21302400	1.61882100
H	-5.70243400	-0.02379900	-1.35023000
H	-5.29775900	-1.75898900	-1.33195600
H	-6.39878100	-1.08735400	-0.09305900
H	-0.74369900	-2.63871800	2.77202900
H	0.17133900	-1.23905400	2.18210600
H	-1.23039600	-0.97570700	3.21519100
Cl	5.98232200	-0.34830100	0.12725900

Compound **1b**, optimization level PCM-B3LYP/6-311++G(2d,p)

Conformation #44

C	-3.46783500	-0.17480400	0.85791000
C	-2.12652100	-0.40341000	1.16793100
C	-1.10755500	-0.48244600	0.17870500
C	-1.47923200	-0.32014200	-1.16529900
C	-2.80596600	-0.09377400	-1.49176600
C	-3.77425400	-0.02520100	-0.47995000
C	0.13669000	-0.72019900	0.87303800
C	-0.17874600	-0.77242900	2.20473000
N	-1.53012100	-0.58549600	2.38947400
C	1.52469000	-0.91954000	0.31111400
C	1.57538300	-2.15663400	-0.63853100
C	1.14158200	-3.42672600	0.10621700
C	2.94036400	-2.39748400	-1.29514200
C	2.08520400	0.36941600	-0.37158900
C	3.62712900	0.45745300	-0.34462200
C	1.60020800	1.63796500	0.33006600
O	4.27387900	-0.05169100	0.54363600
O	1.89860100	1.95362000	1.45862800
O	0.82189200	2.37757300	-0.46935900
C	0.30517900	3.60359200	0.09681200
C	4.26740700	1.25513300	-1.45188300
H	-4.22944800	-0.11693800	1.62414100
H	-0.74478600	-0.36270000	-1.95920200
H	-3.10163900	0.03294700	-2.52441000
H	0.46938400	-0.93326400	3.05172800
H	-1.99859800	-0.57482000	3.28093000
H	2.18764600	-1.12142400	1.15659400
H	0.85388200	-1.97950700	-1.44329600
H	1.83069500	-3.64724200	0.92762700
H	0.13932400	-3.33430200	0.52516400
H	1.14723600	-4.28348900	-0.57157700
H	2.88975300	-3.28669800	-1.92788200
H	3.25349100	-1.57304200	-1.93843800
H	3.72041400	-2.56180100	-0.54833900
H	1.75343500	0.42790800	-1.40868200
H	1.12584500	4.26143800	0.37840500
H	-0.29397700	4.05289200	-0.68950800
H	-0.30854700	3.38359700	0.96864900
H	5.33750900	1.35101500	-1.27981300
H	4.09188100	0.76334100	-2.41252500
H	3.81077200	2.24636400	-1.52024500
Cl	-5.45651500	0.26668100	-0.94149000

Compound **1b**, optimization level PCM-M06-2X/6-311++G(2d,p)

Conformation #1

C	-3.76833300	-0.69207900	-0.16125400
C	-2.43659500	-1.09584700	-0.05484900
C	-1.40980400	-0.21805200	0.36015500
C	-1.73327700	1.10910700	0.67112100
C	-3.04495500	1.52611100	0.56822600
C	-4.03426400	0.62103200	0.15567200
C	-0.18229200	-0.96643900	0.36205000
C	-0.50827700	-2.22863800	-0.04711500
N	-1.85932800	-2.31246200	-0.29662000
C	1.18395600	-0.39538400	0.64123800
C	2.18208600	-1.46607500	1.12557900
C	1.73403000	-2.00601800	2.48648000
C	3.61681200	-0.95214300	1.23947000
C	1.59649900	0.41558300	-0.62568100
C	1.76325700	-0.49272100	-1.84236400
C	2.79131300	1.33298500	-0.46742900
O	2.64868700	-1.31309700	-1.88639200
O	3.66351600	1.48193900	-1.28512400
O	2.72293400	2.02742100	0.66761600
C	3.80577500	2.93611200	0.90182000
C	0.75766400	-0.32391000	-2.94337400
H	-4.55260800	-1.36715600	-0.47602800
H	-0.96958000	1.80716300	0.99483500
H	-3.32286100	2.54436500	0.80317200
H	0.11794700	-3.09636000	-0.18227600
H	-2.34337500	-3.14532300	-0.59092200
H	1.08554100	0.34487200	1.44276900
H	2.18232300	-2.28713500	0.40367600
H	1.77093200	-1.21001100	3.23583200
H	0.71850300	-2.40027200	2.46024500
H	2.40196600	-2.80419500	2.81444900
H	3.66845900	-0.08625000	1.90467000
H	4.25233100	-1.73344300	1.66030600
H	4.03237800	-0.68076000	0.26889100
H	0.76497700	1.10051700	-0.82493100
H	4.74530000	2.38681000	0.94516000
H	3.85185000	3.67862300	0.10762000
H	3.59384300	3.40712100	1.85613800
H	0.92403600	-1.05366600	-3.73175900
H	-0.24898400	-0.42850900	-2.52840400
H	0.83513100	0.69052900	-3.34405800
Cl	-5.68561600	1.19579300	0.03939300

Compound **1b**, optimization level PCM-M06-2X/6-311++G(2d,p)

Conformation #2

C	-3.47981700	-0.15223200	1.00039100
C	-2.10633500	0.05146300	1.14308500
C	-1.25837100	0.31162200	0.04269400
C	-1.81224900	0.36055300	-1.24273900
C	-3.16823200	0.15868100	-1.40338900
C	-3.97437400	-0.09098200	-0.28266000
C	0.07567400	0.46374900	0.55693200
C	-0.02017700	0.28606500	1.90967700
N	-1.32625200	0.04336700	2.26692600
C	1.31144000	0.74623100	-0.25359400
C	1.69081800	2.24804700	-0.29729800
C	2.03426600	2.83712000	1.06951800
C	0.57413800	3.05779000	-0.95339700
C	2.48484900	-0.12319000	0.22350900
C	3.68840700	-0.07771600	-0.73017200
C	2.08996100	-1.58599900	0.35778900
O	3.54064900	0.12776600	-1.90954700
O	2.36788400	-2.27817300	1.30434100
O	1.42414300	-2.02037900	-0.70707400
C	0.97874500	-3.38144600	-0.64860700
C	5.02968400	-0.32365200	-0.10354900
H	-4.12444800	-0.34921600	1.84639700
H	-1.19001600	0.55508100	-2.10810800
H	-3.62074700	0.19056600	-2.38501700
H	0.74940500	0.30744200	2.66598600
H	-1.64820500	-0.13008900	3.20532100
H	1.10601500	0.44554300	-1.28416400
H	2.57606100	2.32537500	-0.93556200
H	1.17334200	2.78962300	1.74011900
H	2.87168200	2.32775400	1.55025600
H	2.31282600	3.88661300	0.95891400
H	-0.33312500	3.02949700	-0.34470300
H	0.87421700	4.10135100	-1.06330700
H	0.33072500	2.66827000	-1.94438600
H	2.82604800	0.16725500	1.21762000
H	1.83156400	-4.05053400	-0.55169200
H	0.30889400	-3.51407600	0.19941800
H	0.45476200	-3.55966100	-1.58183500
H	5.79112100	-0.45475000	-0.86788000
H	5.27908700	0.53466600	0.52637200
H	4.98257900	-1.19654700	0.55105400
Cl	-5.69176400	-0.33694500	-0.53495300

Compound **1b**, optimization level PCM-M06-2X/6-311++G(2d,p)

Conformation #3

C	-3.81552500	0.50555500	0.50178600
C	-2.49376600	0.95301200	0.48103800
C	-1.49029800	0.31752700	-0.28482800
C	-1.82735300	-0.80753500	-1.04839600
C	-3.12933400	-1.26574200	-1.03685800
C	-4.09535600	-0.60323400	-0.26496200
C	-0.26728100	1.04375600	-0.07417000
C	-0.57295000	2.05544700	0.79175500
N	-1.90719500	2.00703900	1.12645000
C	1.08187500	0.65536700	-0.62004500
C	2.04867600	1.85316100	-0.70862600
C	1.53081800	2.85440500	-1.74483300
C	3.48044400	1.45733400	-1.06742000
C	1.57354000	-0.56573100	0.21194300
C	1.83968200	-0.18870900	1.66850100
C	2.70204100	-1.32559200	-0.45695200
O	2.71016300	0.59445900	1.96606500
O	2.71330000	-1.58715700	-1.63263800
O	3.63640700	-1.73672400	0.39702700
C	4.70645500	-2.49886200	-0.17739000
C	0.93080700	-0.81320300	2.68689300
H	-4.58175400	0.99651100	1.08657800
H	-1.08190800	-1.31665000	-1.64873500
H	-3.41744500	-2.13130100	-1.61739800
H	0.05694800	2.82714300	1.20521900
H	-2.37579900	2.66035000	1.73301700
H	0.94493900	0.26815700	-1.63647700
H	2.07799700	2.34257900	0.26846200
H	1.53372100	2.39970300	-2.73958700
H	0.51587100	3.18567700	-1.52627900
H	2.17714500	3.73307900	-1.77741400
H	3.51032100	0.92284300	-2.01969300
H	4.09273300	2.35569900	-1.16570900
H	3.93713700	0.83384800	-0.29752100
H	0.74882700	-1.28644000	0.20281600
H	5.22765400	-1.89933300	-0.92194200
H	5.36716200	-2.74524700	0.64718300
H	4.31539000	-3.40207900	-0.64162400
H	-0.10609200	-0.57880000	2.42797400
H	1.03396200	-1.90054800	2.64142000
H	1.16521900	-0.45266100	3.68520800
Cl	-5.73444000	-1.22283400	-0.28111100

Compound **1b**, optimization level PCM-M06-2X/6-311++G(2d,p)

Conformation #5

C	3.53559400	0.39646500	0.90354500
C	2.16317500	0.21094200	1.07711900
C	1.32464000	-0.27145900	0.04693900
C	1.88807300	-0.57124200	-1.19994400
C	3.24326700	-0.39293800	-1.39029100
C	4.03971800	0.08423700	-0.33837300
C	-0.01111700	-0.33002400	0.57625000
C	0.07482000	0.11449200	1.86716200
N	1.37602400	0.43525900	2.17331900
C	-1.23587100	-0.78887100	-0.16755800
C	-1.60215200	-2.27124500	0.08309700
C	-2.03347500	-2.55195700	1.52074300
C	-0.44715600	-3.18531500	-0.31913600
C	-2.41871500	0.15923500	0.13364400
C	-3.70835900	-0.30472800	-0.55112800
C	-2.05976000	1.56193300	-0.31731300
O	-4.65140100	-0.64190700	0.12524700
O	-1.54821200	1.82788100	-1.37542400
O	-2.39419400	2.47464900	0.59141300
C	-2.09725600	3.83356900	0.24074200
C	-3.74879800	-0.33704600	-2.05335500
H	4.17187200	0.76565700	1.69653100
H	1.27460900	-0.93900800	-2.01370800
H	3.70277200	-0.61650900	-2.34329500
H	-0.69658200	0.23555100	2.61173700
H	1.69094600	0.79402200	3.06022200
H	-1.01477200	-0.68671400	-1.23432400
H	-2.44909300	-2.50308400	-0.57399100
H	-2.28008100	-3.60908500	1.63332800
H	-1.22432000	-2.32234400	2.21787700
H	-2.91392600	-1.97424700	1.80711000
H	0.41712600	-3.02302200	0.32933800
H	-0.74399800	-4.23174400	-0.23198100
H	-0.13645500	-3.00490500	-1.35056600
H	-2.63480400	0.18500600	1.20035200
H	-1.02631700	3.95035900	0.08573600
H	-2.63182000	4.10942600	-0.66618900
H	-2.43107700	4.43344800	1.08081500
H	-3.67206300	0.68132400	-2.43942500
H	-2.89675700	-0.89071500	-2.45109800
H	-4.67961900	-0.79160600	-2.38207400
Cl	5.75605100	0.29236000	-0.62660900

Compound **1b**, optimization level PCM-M06-2X/6-311++G(2d,p)

Conformation #7

C	-3.41482300	-0.13825800	0.87438300
C	-2.07354800	-0.34627000	1.19951100
C	-1.04617500	-0.42964800	0.22793600
C	-1.40656600	-0.29173500	-1.12255200
C	-2.72681300	-0.08098600	-1.46280500
C	-3.70670300	-0.00867100	-0.46278100
C	0.19002900	-0.63575100	0.94723500
C	-0.14520000	-0.65870600	2.27214700
N	-1.49695200	-0.49636400	2.43065300
C	1.60471900	-0.76600000	0.44765700
C	1.95942700	-2.14468400	-0.15385800
C	1.20296800	-2.47575900	-1.43779600
C	1.75650500	-3.24498400	0.88487800
C	1.94615000	0.40551600	-0.51247200
C	3.36291300	0.27224600	-1.07373100
C	1.76797700	1.70477100	0.24563800
O	3.50947900	0.07629400	-2.25794800
O	2.34728100	1.98228900	1.26566200
O	0.87904800	2.50300500	-0.33913700
C	0.61178600	3.73508300	0.34424100
C	4.53153600	0.36907500	-0.13363400
H	-4.18513500	-0.07953500	1.63164400
H	-0.66976900	-0.35764300	-1.91177800
H	-3.01698600	0.02652200	-2.49898000
H	0.49760700	-0.77919100	3.13048100
H	-1.98130800	-0.46908700	3.31339600
H	2.24510700	-0.63660100	1.32550300
H	3.02922400	-2.11474000	-0.39344700
H	0.13209300	-2.56308600	-1.24260800
H	1.35816500	-1.72289300	-2.21323000
H	1.54787300	-3.43272100	-1.83346400
H	0.69670000	-3.35799800	1.12348300
H	2.12228900	-4.19932000	0.50247000
H	2.28994000	-3.02038500	1.81138000
H	1.27830300	0.41227100	-1.37011500
H	0.22938400	3.52905200	1.34244100
H	1.52166300	4.32799500	0.41463800
H	-0.13453600	4.24699800	-0.25413500
H	4.36848100	-0.23286700	0.76087900
H	5.43522500	0.05203900	-0.64784200
H	4.63504700	1.40425100	0.19744400
Cl	-5.37013300	0.25631400	-0.94443100

Compound **1b**, optimization level PCM-M06-2X/6-311++G(2d,p)

Conformation #8

C	3.42637100	0.04187700	0.84951600
C	2.09289500	0.31185100	1.16205500
C	1.07258000	0.39250300	0.18349100
C	1.42756100	0.18395700	-1.15847700
C	2.73958100	-0.08779300	-1.48648000
C	3.71459400	-0.15302300	-0.48058800
C	-0.15855000	0.67355700	0.88378500
C	0.17136400	0.73920400	2.20834400
N	1.51657700	0.53351600	2.38230400
C	-1.56024700	0.84960200	0.35986000
C	-1.84186500	2.25175200	-0.23457700
C	-0.95995800	2.61437600	-1.42790000
C	-1.71949200	3.31961700	0.85023100
C	-1.95282900	-0.27044200	-0.62045900
C	-3.46748900	-0.33552100	-0.86739600
C	-1.52156200	-1.63488300	-0.10681000
O	-4.25473000	0.11709000	-0.07392400
O	-0.82762900	-2.40675900	-0.71748300
O	-2.01696800	-1.88053500	1.10312600
C	-1.60760000	-3.11662200	1.70155900
C	-3.89029900	-1.00441700	-2.14381300
H	4.19356700	-0.01402100	1.61014000
H	0.69012000	0.23559600	-1.94840600
H	3.02748300	-0.25156500	-2.51590200
H	-0.47010000	0.91845800	3.05741900
H	1.99528600	0.52404200	3.26841700
H	-2.22958300	0.74133000	1.21705500
H	-2.88286300	2.23990400	-0.57109500
H	-1.24246900	3.59749100	-1.80902100
H	0.09152800	2.65911800	-1.13590500
H	-1.05842100	1.90696300	-2.25370600
H	-0.68663200	3.40055300	1.19700800
H	-2.02292400	4.29344200	0.46197400
H	-2.34862800	3.08418300	1.71157400
H	-1.46155000	-0.16217000	-1.58718800
H	-0.52462800	-3.12917900	1.81520300
H	-2.09469600	-3.15062400	2.67048900
H	-1.92220300	-3.95493500	1.08304700
H	-3.61847700	-0.35412800	-2.97968700
H	-3.34739700	-1.94213200	-2.27972900
H	-4.96316400	-1.17779600	-2.14606700
Cl	5.36847100	-0.49947500	-0.94526800

Compound **1b**, optimization level PCM-M06-2X/6-311++G(2d,p)

Conformation #9

C	3.50699100	-0.06090300	0.99814300
C	2.13228200	-0.25781400	1.13818700
C	1.25622900	-0.31742200	0.03082500
C	1.78299200	-0.16828000	-1.25837600
C	3.13987100	0.02897700	-1.41593800
C	3.97424200	0.07709100	-0.28907100
C	-0.07003200	-0.52415500	0.54656900
C	0.05760000	-0.57187200	1.90769800
N	1.37554100	-0.41783300	2.26673600
C	-1.32572600	-0.64838000	-0.27227700
C	-1.75928400	-2.10922200	-0.54272300
C	-2.18885600	-2.85898300	0.71602500
C	-0.65073300	-2.86902000	-1.26759700
C	-2.45938400	0.17967900	0.37728600
C	-3.77072900	0.05661100	-0.40442800
C	-2.06070900	1.63474100	0.54038500
O	-4.71574000	-0.49733300	0.10592600
O	-2.28618000	2.28248100	1.53110400
O	-1.44976200	2.12374100	-0.53606500
C	-1.04568600	3.49668100	-0.45264000
C	-3.83598900	0.62565100	-1.79456300
H	4.17262600	-0.01678900	1.84955100
H	1.14028800	-0.20554400	-2.12985100
H	3.57173100	0.14735100	-2.40022000
H	-0.69491100	-0.70044700	2.67010000
H	1.72034000	-0.40737300	3.21308300
H	-1.11277200	-0.19895600	-1.24581500
H	-2.62176700	-2.06063800	-1.21871800
H	-1.36483200	-2.91462100	1.43123600
H	-3.04363900	-2.38925000	1.20526000
H	-2.47517000	-3.88015100	0.45859400
H	0.22521800	-2.97978000	-0.62390900
H	-0.99415600	-3.86650200	-1.54644600
H	-0.34012500	-2.34971200	-2.17694900
H	-2.67364100	-0.18545600	1.38050800
H	-0.34491800	3.62641400	0.37005100
H	-0.56894600	3.72090600	-1.40118300
H	-1.91530100	4.13283100	-0.29842700
H	-4.74758900	0.29027400	-2.28221200
H	-3.83391100	1.71668500	-1.73190500
H	-2.96127400	0.33972200	-2.37973000
Cl	5.69111400	0.32673400	-0.53899500

Compound **1b**, optimization level PCM-M06-2X/6-311++G(2d,p)

Conformation #14

C	-3.41207600	-0.02174100	1.18825700
C	-2.05092100	0.28825000	1.18737000
C	-1.30350300	0.43396300	-0.00415600
C	-1.95212500	0.25934100	-1.23431400
C	-3.29785500	-0.04581100	-1.25231600
C	-4.00060200	-0.18119100	-0.04542000
C	0.04735300	0.75175800	0.37571700
C	0.05816100	0.78487800	1.74183100
N	-1.19626600	0.50765400	2.23249400
C	1.19742000	0.97367900	-0.57199400
C	2.05324600	2.20169700	-0.19327400
C	1.21557900	3.47175800	-0.34211300
C	3.31720000	2.31163400	-1.04465700
C	2.02859100	-0.31417400	-0.80884900
C	1.07530700	-1.45647700	-1.18400200
C	2.93925400	-0.70161700	0.33504200
O	0.62036000	-1.48743900	-2.30264100
O	2.89785800	-0.27598100	1.46021300
O	3.82322100	-1.61992500	-0.06768100
C	4.71322000	-2.11050600	0.94304500
C	0.70814400	-2.46547300	-0.13365000
H	-3.97758400	-0.13208700	2.10376700
H	-1.40700200	0.35089700	-2.16598100
H	-3.82103000	-0.18583200	-2.18829700
H	0.87653200	0.97441100	2.41627200
H	-1.44335300	0.48329500	3.20870000
H	0.76235900	1.18732500	-1.55598400
H	2.35027000	2.09828000	0.85316100
H	0.94446800	3.62447000	-1.39080100
H	0.29658100	3.41715700	0.24276700
H	1.78069700	4.34446600	-0.01088500
H	3.06715000	2.30014500	-2.10997200
H	3.83083100	3.25055000	-0.83237900
H	4.02473600	1.50238300	-0.85290600
H	2.66116500	-0.16882600	-1.68624700
H	4.14362900	-2.58020400	1.74349100
H	5.34926000	-2.83713800	0.44842500
H	5.30645800	-1.29240300	1.34693900
H	-0.10942900	-3.08895700	-0.48642200
H	1.57969500	-3.08954800	0.08223300
H	0.43183800	-1.95289200	0.79067500
Cl	-5.70725100	-0.57482900	-0.12013200

Compound **1b**, optimization level PCM-M06-2X/6-311++G(2d,p)

Conformation #15

C	-3.36365900	-0.48478400	0.77896000
C	-2.02684900	-0.79064700	1.04142500
C	-0.98042100	-0.52398500	0.12531400
C	-1.31277700	0.07633500	-1.09931200
C	-2.62754100	0.38860400	-1.37551800
C	-3.62830500	0.10352900	-0.43507000
C	0.24525300	-0.96470200	0.74871900
C	-0.11370900	-1.45615700	1.97214600
N	-1.47071100	-1.36414500	2.15126500
C	1.66734500	-0.91498000	0.25291500
C	2.03535300	-2.06028400	-0.72223300
C	1.18809900	-2.09025400	-1.99308800
C	1.95863000	-3.40853400	-0.00932300
C	2.03392000	0.46125900	-0.33200900
C	3.54967500	0.65583500	-0.47211900
C	1.54755400	1.56608500	0.59168500
O	4.32950500	0.01823600	0.19062300
O	1.88322100	1.67219900	1.74341400
O	0.71157200	2.40131600	-0.01815700
C	0.15191800	3.43008900	0.80911800
C	3.98367800	1.69684800	-1.46405000
H	-4.15085500	-0.69592400	1.49040400
H	-0.55441500	0.30040400	-1.83754300
H	-2.89834100	0.85214800	-2.31426100
H	0.51439300	-1.86916000	2.74627800
H	-1.97018100	-1.65153400	2.97728700
H	2.30742900	-1.03851800	1.13059700
H	3.07816900	-1.89725000	-1.01061300
H	1.25362300	-1.16153600	-2.56342200
H	1.53003400	-2.89589300	-2.64543800
H	0.13791300	-2.27492600	-1.75631800
H	0.92820400	-3.63811900	0.27187500
H	2.31602400	-4.20602100	-0.66313000
H	2.56670800	-3.41258100	0.89805300
H	1.58104000	0.62717700	-1.30930500
H	0.94357400	4.05750600	1.21406600
H	-0.50133400	4.00530800	0.16131000
H	-0.41366700	2.97931700	1.62311900
H	5.04662900	1.89979800	-1.36398500
H	3.76996200	1.32603000	-2.47001000
H	3.40335300	2.61277400	-1.33211400
Cl	-5.28450900	0.51708500	-0.83165200

Compound **1b**, optimization level PCM-M06-2X/6-311++G(2d,p)

Conformation #17

C	3.14032100	0.25579300	1.17238300
C	1.78602200	0.55317200	1.32466500
C	0.86752800	0.54254600	0.24587400
C	1.35383900	0.21959100	-1.03047600
C	2.69258400	-0.07466800	-1.20059100
C	3.56036100	-0.05212200	-0.10113600
C	-0.42202400	0.89424600	0.79171400
C	-0.22308000	1.09543300	2.12685100
N	1.09511400	0.89298400	2.45489500
C	-1.74370100	1.06127400	0.08774600
C	-1.64067900	1.94077900	-1.17905100
C	-1.03425800	3.29736700	-0.81868000
C	-3.00045900	2.14516500	-1.84888500
C	-2.44972200	-0.29501000	-0.16226800
C	-2.97978500	-0.93940400	1.11852200
C	-1.59442100	-1.22756600	-0.99145100
O	-3.00436400	-0.33098600	2.16115200
O	-1.51986900	-1.17774800	-2.19464000
O	-0.90131100	-2.08437700	-0.24578600
C	-0.04349500	-2.98644500	-0.95731600
C	-3.55091000	-2.33155400	1.01178400
H	3.82668800	0.26612100	2.00836800
H	0.69594800	0.17941500	-1.88887900
H	3.08025800	-0.32732300	-2.17815100
H	-0.94283400	1.36751800	2.88207600
H	1.48653000	0.97654100	3.37904500
H	-2.40476300	1.58981300	0.78185600
H	-0.98326700	1.45124700	-1.90159000
H	-0.96750700	3.93100700	-1.70435900
H	-1.66373600	3.80907500	-0.08417000
H	-0.03483800	3.19573400	-0.39477100
H	-3.74309200	2.48399000	-1.12007400
H	-2.92331900	2.90779300	-2.62537100
H	-3.37214300	1.23674800	-2.32385200
H	-3.34691700	-0.11714300	-0.76299800
H	0.71015000	-2.42459200	-1.50616900
H	0.42062500	-3.60907600	-0.19929800
H	-0.63107000	-3.59100900	-1.64597700
H	-4.41143800	-2.41492000	1.67281000
H	-3.83296500	-2.58166700	-0.01078500
H	-2.78942700	-3.04190600	1.33762600
Cl	5.25040900	-0.43440800	-0.36491400

Compound **1b**, optimization level PCM-M06-2X/6-311++G(2d,p)

Conformation #20

C	3.63238500	0.50306800	0.80366700
C	2.24966200	0.43093100	0.97974100
C	1.40368300	-0.23825000	0.06671000
C	1.96769400	-0.85458500	-1.05723100
C	3.33372100	-0.79428200	-1.24650200
C	4.13737300	-0.11945800	-0.31557100
C	0.05783800	-0.10457100	0.55388400
C	0.14504900	0.61630400	1.71156500
N	1.45813200	0.94107100	1.97163800
C	-1.15608400	-0.70897500	-0.09630800
C	-1.24437400	-2.22149800	0.24655300
C	-2.24642300	-2.98820600	-0.61583600
C	-1.50936000	-2.49226700	1.72690400
C	-2.43426800	0.07458700	0.25029100
C	-3.61111800	-0.23634500	-0.69130200
C	-2.23339300	1.58045500	0.15011400
O	-3.43515400	-0.38125700	-1.87506200
O	-2.61119200	2.36483700	0.98443900
O	-1.62731100	1.93280800	-0.97700600
C	-1.36850600	3.33402700	-1.12885800
C	-4.96817800	-0.28137200	-0.05146300
H	4.27680900	1.01731400	1.50389000
H	1.34562800	-1.37559600	-1.77571200
H	3.79574200	-1.26141300	-2.10532800
H	-0.63225500	0.93506900	2.38892500
H	1.77672700	1.48099700	2.75983300
H	-1.02859800	-0.62812100	-1.18020100
H	-0.24933400	-2.61461100	0.01293300
H	-2.11913600	-2.76845500	-1.67673000
H	-3.27873300	-2.75357300	-0.34350000
H	-2.11393000	-4.06108100	-0.46447800
H	-0.81555300	-1.94917500	2.36999400
H	-2.52853600	-2.21363200	2.00711100
H	-1.39921800	-3.55812700	1.93360400
H	-2.74821100	-0.10488700	1.27868000
H	-2.30324300	3.89125900	-1.11904000
H	-0.72590300	3.67927500	-0.32028100
H	-0.86902000	3.43894300	-2.08628800
H	-5.74290200	-0.40128300	-0.80413500
H	-5.00392100	-1.10863000	0.66198500
H	-5.12772400	0.63616200	0.52053600
Cl	5.86702600	-0.07037600	-0.59649200

Compound **1b**, optimization level PCM-M06-2X/6-311++G(2d,p)

Conformation #21

C	3.51953100	0.52108600	0.87865500
C	2.14983600	0.30996000	1.04552200
C	1.34210300	-0.26923500	0.04061200
C	1.93374100	-0.64105100	-1.17321300
C	3.28655200	-0.43829900	-1.35671800
C	4.05206200	0.13514700	-0.33034700
C	-0.00041400	-0.32844800	0.55163800
C	0.05146700	0.20958900	1.80805700
N	1.33839600	0.59033800	2.11054700
C	-1.20248100	-0.87255800	-0.17237900
C	-1.52350800	-2.34402800	0.19004700
C	-1.91682100	-2.54343300	1.65252800
C	-0.34620000	-3.24931800	-0.16691400
C	-2.41218800	0.05093000	0.03276900
C	-3.62180200	-0.34556000	-0.82590500
C	-2.05542800	1.47096200	-0.38094100
O	-3.46987300	-0.85834900	-1.90704800
O	-1.47770100	1.75277400	-1.39810100
O	-2.48401900	2.37051800	0.50272100
C	-2.20456100	3.73904600	0.17444100
C	-4.97186000	-0.03175500	-0.25065700
H	4.13301600	0.96379800	1.65173500
H	1.34292300	-1.08346100	-1.96629000
H	3.76762500	-0.71587100	-2.28455700
H	-0.73647700	0.35981500	2.52999100
H	1.62871400	1.01701700	2.97540900
H	-0.98231200	-0.85023800	-1.24324400
H	-2.37032600	-2.64008900	-0.43562400
H	-1.09740900	-2.26100400	2.31746600
H	-2.80010900	-1.96656300	1.93382200
H	-2.14457500	-3.59538700	1.83346700
H	0.52414700	-3.01535800	0.45111800
H	-0.60719700	-4.29582800	-0.00013300
H	-0.05948200	-3.13382800	-1.21448400
H	-2.72449900	0.08619400	1.07675300
H	-2.61718100	4.32655900	0.98783500
H	-1.12919700	3.88881200	0.09832300
H	-2.67960900	4.00010300	-0.76924700
H	-5.75015100	-0.19044700	-0.99243500
H	-5.14211900	-0.68157300	0.61201600
H	-4.99091500	0.99667600	0.11658400
Cl	5.76699700	0.37062100	-0.60800500

Compound **1b**, optimization level PCM-M06-2X/6-311++G(2d,p)

Conformation #22

C	3.18697500	-1.03389100	-0.69946600
C	1.83762100	-1.36207000	-0.56596100
C	0.91905300	-0.56515900	0.16043000
C	1.39968700	0.60077800	0.77538200
C	2.73193900	0.94246300	0.65357700
C	3.60018200	0.12305600	-0.08000100
C	-0.36452200	-1.21792000	0.07000500
C	-0.16331200	-2.34127100	-0.67948200
N	1.15173800	-2.43544800	-1.06364300
C	-1.68725500	-0.80543100	0.66151300
C	-1.59238000	-0.40580100	2.15125600
C	-0.95760700	-1.54455700	2.95019000
C	-2.96301400	-0.06646200	2.73927000
C	-2.40784000	0.23940600	-0.22832100
C	-2.99137100	-0.37561000	-1.50310100
C	-1.52954000	1.42574700	-0.55993000
O	-3.03480200	-1.57261400	-1.65567200
O	-0.92931100	1.57173200	-1.59416200
O	-1.47514700	2.29765500	0.44935400
C	-0.66053500	3.45633200	0.22053300
C	-3.57771900	0.56604600	-2.52254900
H	3.87469100	-1.65327500	-1.25953400
H	0.74321900	1.24452000	1.34532500
H	3.11534200	1.83946300	1.12069500
H	-0.87959100	-3.08972500	-0.97818900
H	1.54313900	-3.17139300	-1.62880100
H	-2.33544700	-1.68622300	0.62268200
H	-0.95954300	0.47835200	2.25082900
H	-1.56351500	-2.45151300	2.86181100
H	0.04859300	-1.77483700	2.59879800
H	-0.89895500	-1.28048700	4.00719300
H	-2.88893100	0.03885300	3.82272900
H	-3.36544300	0.86993900	2.35163300
H	-3.68145100	-0.86454200	2.52904300
H	-3.27781600	0.62702300	0.31095700
H	-1.07274700	4.04119400	-0.59976200
H	-0.68757400	4.02211600	1.14617400
H	0.35839700	3.15733600	-0.02077400
H	-4.37651900	0.06038700	-3.06048000
H	-3.94725000	1.48156900	-2.06041000
H	-2.79109300	0.84293200	-3.22617700
Cl	5.28307200	0.59578500	-0.20849800

Compound **1b**, optimization level PCM-M06-2X/6-311++G(2d,p)

Conformation #23

C	-3.40175900	0.58520400	0.79199100
C	-2.04180500	0.85361000	0.96048700
C	-1.08070400	0.56173500	-0.03388300
C	-1.50497000	-0.01709900	-1.23740500
C	-2.84492900	-0.28743700	-1.42203300
C	-3.76568700	0.01617300	-0.40666600
C	0.20076400	0.95730000	0.48237700
C	-0.03359700	1.45868400	1.73251600
N	-1.37649200	1.40066100	2.02296000
C	1.53429600	0.79493400	-0.20076700
C	2.35377800	2.10283200	-0.14351300
C	1.62576500	3.20797800	-0.90858800
C	3.77546600	1.96026300	-0.68436400
C	2.31425600	-0.40061900	0.40214100
C	3.32114600	-1.03395500	-0.56695700
C	1.35364900	-1.49833400	0.83073200
O	3.17441600	-0.96961300	-1.76205800
O	1.19569500	-1.86970900	1.96520800
O	0.69045300	-1.99167200	-0.21170900
C	-0.36480700	-2.90942900	0.09860400
C	4.46604500	-1.76739900	0.07382200
H	-4.13247200	0.80779000	1.55775100
H	-0.79087500	-0.26494200	-2.01347500
H	-3.19831400	-0.73649500	-2.34022400
H	0.65708700	1.85858500	2.45836200
H	-1.79799700	1.71170300	2.88326300
H	1.35302500	0.55030100	-1.25180200
H	2.42406700	2.39617900	0.91045100
H	2.16809800	4.15142900	-0.82776800
H	1.56045800	2.94759400	-1.96892000
H	0.61391000	3.36078600	-0.53234400
H	3.76447600	1.63126400	-1.72555000
H	4.28438100	2.92443700	-0.63674300
H	4.37560700	1.25280000	-0.10795200
H	2.83678900	-0.09616100	1.31173400
H	-1.11231500	-2.40884200	0.71444500
H	-0.79371900	-3.19839000	-0.85566700
H	0.03165500	-3.77621300	0.62367500
H	5.04258500	-2.30285900	-0.67580400
H	5.10519000	-1.04795900	0.59172400
H	4.08799400	-2.45841700	0.83100400
Cl	-5.45706700	-0.35093700	-0.68525700

Compound **1b**, optimization level PCM-M06-2X/6-311++G(2d,p)

Conformation #37

C	3.53934300	-0.75042900	-0.99894100
C	2.15337400	-0.58987400	-1.01517900
C	1.46494700	0.17771000	-0.04842500
C	2.20047300	0.80381200	0.96639900
C	3.57267700	0.65447800	0.99941000
C	4.21474100	-0.11668200	0.01959400
C	0.06355500	0.11827300	-0.37183400
C	-0.03134300	-0.66203100	-1.49168900
N	1.21761900	-1.08677600	-1.87978800
C	-1.02776100	0.81014600	0.40370000
C	-1.49130600	2.12799500	-0.26215700
C	-0.31773100	3.09950200	-0.37830400
C	-2.63362800	2.78483700	0.51283000
C	-2.21203200	-0.11608200	0.78408000
C	-1.72941500	-1.48789100	1.25429500
C	-3.24965900	-0.31892100	-0.29818500
O	-2.12034200	-2.50300300	0.72756700
O	-3.04505900	-0.30626800	-1.48493900
O	-4.45285200	-0.53486800	0.23385200
C	-5.50323800	-0.80667700	-0.70195500
C	-0.75927100	-1.49935800	2.40292700
H	4.06012700	-1.33908900	-1.74214900
H	1.70842300	1.40425800	1.72280500
H	4.16246600	1.12727800	1.77272600
H	-0.90662300	-0.95287000	-2.04760100
H	1.40538000	-1.68077000	-2.67115800
H	-0.58678600	1.09983600	1.36531700
H	-1.84036000	1.88353100	-1.26866700
H	0.05952200	3.36252900	0.61472500
H	0.50564400	2.67273100	-0.95203000
H	-0.63525400	4.02012600	-0.87055100
H	-2.34844500	2.94108600	1.55785800
H	-2.86812900	3.75925900	0.08173100
H	-3.55107700	2.19289900	0.49744800
H	-2.72756600	0.32647600	1.64120500
H	-5.63948200	0.04397200	-1.36728700
H	-5.26131500	-1.69321100	-1.28534200
H	-6.39430300	-0.97185200	-0.10524700
H	-0.67653000	-2.50800000	2.80006100
H	0.22002400	-1.17172600	2.04456200
H	-1.07612500	-0.80523100	3.18243900
Cl	5.95827900	-0.27785500	0.10096700

Compound **1b**, optimization level PCM-M06-2X/6-311++G(2d,p)

Conformation #44

C	-3.39244600	-0.23787100	0.85040200
C	-2.04796300	-0.44588200	1.16383400
C	-1.03310300	-0.50368100	0.17994700
C	-1.39478600	-0.33841000	-1.16461600
C	-2.71891700	-0.13350400	-1.49380300
C	-3.69148200	-0.08798500	-0.48392600
C	0.21368900	-0.70545400	0.87357800
C	-0.09687600	-0.75855100	2.20163500
N	-1.45053500	-0.60838800	2.38425000
C	1.59478300	-0.85744300	0.29762200
C	1.68508000	-2.08057500	-0.64417400
C	1.28946000	-3.35506000	0.09992000
C	3.07273500	-2.25343500	-1.26030000
C	2.08842100	0.43423300	-0.40999800
C	3.60837700	0.61766400	-0.34147200
C	1.47985200	1.64945800	0.26680000
O	4.26689300	0.12154500	0.53805800
O	1.83901700	2.08047000	1.33304000
O	0.47693400	2.15914300	-0.44103100
C	-0.30093000	3.16322800	0.22170500
C	4.20774600	1.48743500	-1.41112600
H	-4.15982800	-0.19222000	1.61154500
H	-0.64849700	-0.35327000	-1.94910900
H	-3.01843300	-0.00195600	-2.52453900
H	0.55812800	-0.89778400	3.04761600
H	-1.92140800	-0.60367600	3.27446500
H	2.27995300	-1.02399400	1.13473300
H	0.96941800	-1.92437000	-1.45887300
H	1.95844600	-3.51901000	0.95002500
H	0.26840700	-3.30155900	0.47820900
H	1.36604700	-4.22016600	-0.56067800
H	3.09128700	-3.14727700	-1.88580500
H	3.35847000	-1.41229600	-1.89568600
H	3.83037400	-2.36501300	-0.48127800
H	1.77486200	0.44515700	-1.45652400
H	0.31994400	4.02170700	0.47000000
H	-1.08059100	3.44224200	-0.47987800
H	-0.73451300	2.74440400	1.12973700
H	5.25812700	1.67461600	-1.20451100
H	4.10147200	0.98903900	-2.37789900
H	3.65969600	2.43034900	-1.47695400
Cl	-5.36012900	0.17762000	-0.95006200

Compound **1b**, optimization level PCM-M11/6-311++G(2d,p)

Conformation #1

C	-3.81841500	-0.69553000	-0.24858400
C	-2.48749200	-1.10062900	-0.14046800
C	-1.46768700	-0.22686200	0.29035100
C	-1.79219100	1.09413200	0.61847800
C	-3.10137800	1.51130300	0.51470800
C	-4.08583900	0.61058400	0.08444500
C	-0.23664000	-0.97128400	0.28621400
C	-0.55477000	-2.22605100	-0.14389100
N	-1.90521200	-2.31214100	-0.39653600
C	1.12063600	-0.41170200	0.62454800
C	2.05423500	-1.47921600	1.23900200
C	1.54354400	-1.83529200	2.63832500
C	3.51346000	-1.03149000	1.31690400
C	1.63736200	0.35541300	-0.62116900
C	1.96642100	-0.51430300	-1.83532600
C	2.81456600	1.27860800	-0.36215700
O	2.35561400	-1.65201800	-1.71050700
O	3.80379600	1.34256700	-1.05373800
O	2.61769500	2.04953000	0.70415800
C	3.70126000	2.93775600	1.03577400
C	1.75405200	0.12703200	-3.17697200
H	-4.60519500	-1.36708300	-0.57834400
H	-1.02483600	1.78973800	0.95384800
H	-3.38410800	2.52993100	0.76193000
H	0.08485500	-3.08621900	-0.29768700
H	-2.38353300	-3.13990500	-0.71847900
H	0.97629800	0.36162700	1.39331200
H	2.01545600	-2.37287300	0.60872400
H	1.62815400	-0.96596700	3.30430500
H	0.49417300	-2.14788800	2.61693900
H	2.13628800	-2.64887600	3.07088200
H	3.60777600	-0.08995300	1.87509300
H	4.10674600	-1.78667400	1.84441900
H	3.95254600	-0.90145400	0.32238700
H	0.82733400	1.03636000	-0.92485500
H	4.60196800	2.35226600	1.24079200
H	3.88749200	3.62259400	0.20528700
H	3.38123600	3.48082600	1.92402900
H	2.19532100	-0.48191000	-3.96712300
H	0.67295600	0.22040600	-3.34243700
H	2.17690700	1.13668000	-3.18208300
Cl	-5.74087900	1.18752500	-0.03385800

Compound **1b**, optimization level PCM-M11/6-311++G(2d,p)

Conformation #2

C	-3.48046200	-0.17789000	1.00189500
C	-2.10677200	0.02594100	1.14208800
C	-1.26841100	0.30284100	0.04227400
C	-1.82635200	0.36980700	-1.23924400
C	-3.18001900	0.16826700	-1.39658600
C	-3.97902300	-0.09872500	-0.27549900
C	0.06924200	0.45071800	0.55101300
C	-0.01717800	0.25350500	1.89990400
N	-1.32022900	0.00283600	2.26150100
C	1.30278700	0.74699300	-0.25949900
C	1.66373100	2.25443400	-0.29737100
C	2.00560800	2.83109600	1.07701800
C	0.52393100	3.05242000	-0.93184600
C	2.48416900	-0.10352500	0.22898400
C	3.69310700	-0.04039500	-0.71714100
C	2.11284000	-1.57529700	0.34402700
O	3.54526600	0.17412200	-1.89677300
O	2.41309200	-2.28082500	1.27715000
O	1.44509200	-2.00758900	-0.72044600
C	1.04582000	-3.39025400	-0.68815100
C	5.03164200	-0.28664400	-0.08453400
H	-4.12543000	-0.38879400	1.84948500
H	-1.20389500	0.57828900	-2.10679100
H	-3.64044300	0.21335600	-2.37877000
H	0.76360500	0.26698200	2.65178300
H	-1.63697300	-0.18380900	3.20086300
H	1.10671900	0.43977500	-1.29442100
H	2.54514300	2.34911200	-0.94486400
H	1.14866700	2.74405200	1.75593200
H	2.86619700	2.33753200	1.54306300
H	2.25118200	3.89477600	0.98426600
H	-0.37509600	3.00327900	-0.30469900
H	0.80495100	4.10583900	-1.03951300
H	0.26837500	2.66371200	-1.92490500
H	2.81417800	0.18657400	1.23122200
H	1.92980900	-4.02843800	-0.61527600
H	0.39319500	-3.56292800	0.17132100
H	0.51324500	-3.56918100	-1.62125000
H	5.79966900	-0.42560200	-0.84619400
H	5.28103700	0.57865600	0.54236200
H	4.97509900	-1.15732400	0.57879300
Cl	-5.70077700	-0.34418400	-0.52507900

Compound **1b**, optimization level PCM-M11/6-311++G(2d,p)

Conformation #3

C	-3.86997500	0.46103100	0.58055400
C	-2.54505400	0.89880900	0.58208000
C	-1.55155700	0.29488000	-0.21628700
C	-1.89686600	-0.78457100	-1.03702400
C	-3.20030600	-1.23162400	-1.04815500
C	-4.15842400	-0.60154800	-0.24155800
C	-0.32008900	0.99710600	0.02977800
C	-0.61228400	1.96230700	0.94848200
N	-1.94721300	1.91232500	1.28024000
C	1.01860200	0.65043900	-0.56740900
C	1.92489300	1.89240200	-0.72372600
C	1.34787100	2.79168600	-1.82126400
C	3.37778900	1.54634300	-1.04917000
C	1.59421300	-0.55055200	0.22746200
C	1.99132800	-0.24481700	1.67221000
C	2.70627900	-1.26139400	-0.52614700
O	2.43361700	0.83387600	1.99363100
O	2.63312900	-1.56129300	-1.69177000
O	3.75983900	-1.54621600	0.23929300
C	4.85438700	-2.20409100	-0.42661100
C	1.76305400	-1.35167600	2.66306100
H	-4.63638500	0.92680800	1.19251800
H	-1.15043600	-1.26954800	-1.66361000
H	-3.49878900	-2.06682600	-1.67426700
H	0.03587000	2.69953900	1.40567900
H	-2.40604200	2.52870100	1.93371500
H	0.84480600	0.25277600	-1.57899100
H	1.92021500	2.44117300	0.22307600
H	1.38997400	2.27868500	-2.79144200
H	0.30430800	3.05539900	-1.61971200
H	1.92665900	3.71814000	-1.90574500
H	3.44455600	0.94580000	-1.96526300
H	3.95064100	2.46637300	-1.21077200
H	3.85806300	1.00021000	-0.22951100
H	0.79469200	-1.30516900	0.27216500
H	5.22659400	-1.56366500	-1.23078500
H	5.61862200	-2.35648800	0.33425400
H	4.51722800	-3.15798400	-0.83860400
H	0.68027400	-1.45685800	2.81064900
H	2.13257600	-2.30120200	2.26249400
H	2.24285500	-1.11975600	3.61480700
Cl	-5.80715500	-1.20598200	-0.28877800

Compound **1b**, optimization level PCM-M11/6-311++G(2d,p)

Conformation #5

C	3.51435000	0.45810500	0.86758800
C	2.14018200	0.28887300	1.04433700
C	1.31359900	-0.27047600	0.04821700
C	1.88449100	-0.66590900	-1.16673000
C	3.23875800	-0.50472600	-1.35905700
C	4.02599500	0.05059300	-0.33999100
C	-0.02815000	-0.28741900	0.56760700
C	0.04459600	0.25605100	1.81896400
N	1.34271600	0.59915800	2.11222800
C	-1.25075600	-0.79672600	-0.14825500
C	-1.60800500	-2.26309900	0.20212000
C	-2.05419700	-2.43424600	1.65433200
C	-0.43180500	-3.18755000	-0.11160000
C	-2.44153700	0.15458300	0.10816400
C	-3.70068300	-0.33533500	-0.61287000
C	-2.06566800	1.55061600	-0.35550300
O	-4.68502800	-0.61902800	0.03199600
O	-1.55059200	1.80261400	-1.41827800
O	-2.36810600	2.47758100	0.54996900
C	-2.03266600	3.83121000	0.18891200
C	-3.64377500	-0.48986800	-2.10733800
H	4.14955700	0.88793800	1.63605400
H	1.27233600	-1.09517300	-1.95689100
H	3.70885700	-0.80160700	-2.29153100
H	-0.74170100	0.43673900	2.54249400
H	1.65024900	1.02268700	2.97462800
H	-1.03553800	-0.75457400	-1.22546100
H	-2.44726000	-2.55577300	-0.44621900
H	-2.25358300	-3.49116400	1.86190900
H	-1.26600500	-2.10189000	2.34104800
H	-2.97027200	-1.87477300	1.87434500
H	0.41752800	-2.96363200	0.54546800
H	-0.71542700	-4.23405900	0.04336300
H	-0.09837600	-3.07353500	-1.14998300
H	-2.68692200	0.20186500	1.17166000
H	-0.95602900	3.90890700	0.01934800
H	-2.57104100	4.11475600	-0.71859700
H	-2.33910400	4.44940500	1.03161100
H	-3.17384600	0.38280900	-2.56902800
H	-3.01941800	-1.35871400	-2.34972600
H	-4.64879000	-0.65122600	-2.49898600
Cl	5.74865900	0.23173600	-0.63082300

Compound **1b**, optimization level PCM-M11/6-311++G(2d,p)

Conformation #7

C	-3.40931800	-0.16693500	0.86851900
C	-2.06871800	-0.37764100	1.19416400
C	-1.04099100	-0.43267900	0.22520500
C	-1.39606100	-0.26652100	-1.12259700
C	-2.71337000	-0.05330100	-1.46190900
C	-3.69547300	-0.00722200	-0.46359000
C	0.19533700	-0.65071000	0.94361500
C	-0.14281200	-0.70828800	2.26506900
N	-1.49515100	-0.55541300	2.42319600
C	1.61264400	-0.76174000	0.44293700
C	1.97859700	-2.13459400	-0.16633000
C	1.21919100	-2.45122300	-1.45401300
C	1.76562500	-3.24161300	0.86582500
C	1.93554700	0.41572400	-0.51712000
C	3.35237700	0.28493900	-1.07793600
C	1.75234100	1.70757800	0.25388100
O	3.49935300	0.06118800	-2.25914200
O	2.33620000	1.97720900	1.27642500
O	0.85267800	2.50864500	-0.31047600
C	0.58761300	3.73753000	0.39199100
C	4.51817700	0.41049800	-0.13808000
H	-4.18715600	-0.12857000	1.62518600
H	-0.65402100	-0.31593500	-1.91413500
H	-3.00318100	0.07674700	-2.50008300
H	0.50326200	-0.84863900	3.12362200
H	-1.98303300	-0.55336200	3.30628600
H	2.25542900	-0.62476100	1.32312200
H	3.05319500	-2.09983700	-0.40213900
H	0.14413100	-2.53079100	-1.25392500
H	1.38026500	-1.69008500	-2.22644600
H	1.55464600	-3.41198100	-1.85985300
H	0.69770500	-3.35751000	1.08612300
H	2.14143200	-4.19787200	0.48616300
H	2.28320900	-3.01631000	1.80627100
H	1.26125200	0.42242200	-1.37394200
H	0.22571400	3.51389300	1.39856600
H	1.50178200	4.33303100	0.45202000
H	-0.17499600	4.25460900	-0.18881900
H	4.33622500	-0.13624900	0.79200400
H	5.42274400	0.04994300	-0.62959600
H	4.63813100	1.46519400	0.13355300
Cl	-5.36122100	0.26381200	-0.94697600

Compound **1b**, optimization level PCM-M11/6-311++G(2d,p)

Conformation #8

C	3.42309300	0.14175400	0.83498300
C	2.09061100	0.43271500	1.13276800
C	1.06357300	0.39918400	0.16274700
C	1.40523500	0.05345600	-1.15308700
C	2.71372800	-0.23963400	-1.46459800
C	3.69795600	-0.18936300	-0.46769800
C	-0.16413900	0.74942500	0.83955900
C	0.17615900	0.96306100	2.14400800
N	1.52356700	0.78340100	2.32696400
C	-1.56934000	0.87071700	0.30682700
C	-1.85811900	2.22894100	-0.38192800
C	-0.94394600	2.51784900	-1.57342100
C	-1.76193400	3.36509000	0.63660700
C	-1.95233200	-0.30660400	-0.60561200
C	-3.47201000	-0.41262500	-0.81413800
C	-1.50175100	-1.63952700	-0.02697900
O	-4.24788600	0.09255900	-0.03889900
O	-0.84749400	-2.46069200	-0.62195700
O	-1.93812000	-1.80630400	1.21868900
C	-1.53012200	-3.02618500	1.86429400
C	-3.90914000	-1.18923000	-2.02331500
H	4.20373400	0.17337200	1.58909700
H	0.65499100	0.01357400	-1.93755500
H	2.99559500	-0.51044200	-2.47748300
H	-0.46399400	1.23499800	2.97500900
H	2.01060000	0.87695300	3.20547500
H	-2.24301600	0.80930700	1.17030600
H	-2.89511200	2.18305900	-0.74024800
H	-1.21577100	3.47697900	-2.02811400
H	0.10189500	2.58412800	-1.25041800
H	-1.01515100	1.75478700	-2.35726500
H	-0.72999100	3.47372900	0.99216500
H	-2.06609700	4.31542500	0.18418900
H	-2.40329500	3.17708500	1.50612300
H	-1.48546100	-0.24343900	-1.59248800
H	-0.43901800	-3.06368500	1.91808200
H	-1.96669200	-2.99761900	2.86176700
H	-1.90197800	-3.88529700	1.30070000
H	-3.70041900	-0.58277600	-2.91342100
H	-3.32180900	-2.10950900	-2.11701500
H	-4.97549200	-1.41188400	-1.97241300
Cl	5.35378700	-0.56889400	-0.91376500

Compound **1b**, optimization level PCM-M11/6-311++G(2d,p)

Conformation #9

C	3.50367700	-0.04645700	0.99741600
C	2.12865600	-0.24014000	1.13862200
C	1.25857400	-0.31378200	0.03137600
C	1.78571200	-0.18358100	-1.25838800
C	3.14037300	0.00960600	-1.41632900
C	3.97130900	0.07249100	-0.28844900
C	-0.07056800	-0.51182000	0.54595500
C	0.05177000	-0.54040500	1.90639300
N	1.36839000	-0.38328100	2.26743400
C	-1.32591300	-0.64318500	-0.27403400
C	-1.75272000	-2.10818800	-0.53685500
C	-2.18852400	-2.83888000	0.73295800
C	-0.62647500	-2.87023300	-1.23517800
C	-2.46415300	0.17547800	0.38133400
C	-3.76816000	0.03501800	-0.40962200
C	-2.06599800	1.63257200	0.53600400
O	-4.70437700	-0.54942600	0.08724400
O	-2.26953800	2.28431300	1.53182800
O	-1.47266600	2.12321800	-0.55006800
C	-1.06863200	3.50293900	-0.47246500
C	-3.83106000	0.61568400	-1.79493900
H	4.17231700	0.00961800	1.85094900
H	1.14002600	-0.23241400	-2.13264500
H	3.57729800	0.11448700	-2.40462900
H	-0.70980800	-0.65814800	2.66820000
H	1.71097100	-0.36140500	3.21612100
H	-1.11657100	-0.18942400	-1.25110000
H	-2.61118300	-2.07085100	-1.22510400
H	-1.37059600	-2.85670500	1.46389200
H	-3.06516400	-2.37464400	1.19757700
H	-2.44714500	-3.87701300	0.49740800
H	0.24057200	-2.96608600	-0.56995800
H	-0.95767000	-3.87752100	-1.50972100
H	-0.30027800	-2.35656100	-2.14748300
H	-2.67450300	-0.19274200	1.38821100
H	-0.35610000	3.63038000	0.34610300
H	-0.60403800	3.73235700	-1.43053500
H	-1.94375700	4.13484000	-0.30210700
H	-4.71562500	0.23985500	-2.31047900
H	-3.88874900	1.70804900	-1.71721800
H	-2.92400400	0.38413900	-2.36171300
Cl	5.69239400	0.31736600	-0.54074300

Compound **1b**, optimization level PCM-M11/6-311++G(2d,p)

Conformation #14

C	-3.38765600	-0.02205900	1.19168400
C	-2.02775500	0.29347400	1.18898900
C	-1.28933600	0.45109300	-0.00283700
C	-1.94052200	0.28605900	-1.23175700
C	-3.28242900	-0.02392900	-1.24737800
C	-3.97817800	-0.17317800	-0.03875400
C	0.06325000	0.77266100	0.37332100
C	0.08046400	0.79772000	1.73788200
N	-1.16959800	0.51016900	2.23232700
C	1.21437800	0.99394400	-0.57467300
C	2.09066600	2.20084100	-0.17179000
C	1.27272300	3.48662700	-0.31236000
C	3.36228000	2.29465800	-1.01656200
C	2.02417200	-0.30502600	-0.82521300
C	1.04472300	-1.41869700	-1.21691400
C	2.91243100	-0.72997400	0.32449200
O	0.59506400	-1.42639700	-2.34026000
O	2.90548000	-0.27938300	1.44298600
O	3.73511000	-1.71237200	-0.05815400
C	4.60423300	-2.23287800	0.96417600
C	0.64076700	-2.42222000	-0.17383200
H	-3.95347100	-0.14306700	2.11038300
H	-1.39569800	0.39128100	-2.16725200
H	-3.81259100	-0.15757600	-2.18536800
H	0.90771300	0.98713900	2.40875200
H	-1.41268700	0.47944500	3.21101900
H	0.78126900	1.23037100	-1.55915900
H	2.37778400	2.07594100	0.87865300
H	1.01066700	3.65492100	-1.36552900
H	0.34484300	3.43608400	0.26693700
H	1.84858100	4.35191300	0.03357400
H	3.11580300	2.28724000	-2.08733200
H	3.89085400	3.22948800	-0.80152100
H	4.05924400	1.47145300	-0.82066800
H	2.67188600	-0.16199000	-1.69637200
H	4.00586300	-2.64426000	1.78130500
H	5.19541100	-3.01287600	0.48614200
H	5.24706400	-1.43508300	1.34379900
H	-0.23761100	-2.97611100	-0.50823500
H	1.47376100	-3.11734500	-0.01119100
H	0.44032900	-1.91175000	0.77566700
Cl	-5.68719500	-0.57387500	-0.11101800

Compound **1b**, optimization level PCM-M11/6-311++G(2d,p)

Conformation #15

C	-3.36594800	-0.53309900	0.75039200
C	-2.03277300	-0.85972900	1.00484500
C	-0.98074800	-0.53537900	0.11825000
C	-1.29988000	0.14161500	-1.06856200
C	-2.60903800	0.47404200	-1.33496900
C	-3.61704200	0.13211700	-0.42292000
C	0.23930000	-1.02441100	0.71885300
C	-0.12973300	-1.59757200	1.90081500
N	-1.48747300	-1.51067100	2.07654400
C	1.66461500	-0.93794700	0.23471400
C	2.04638000	-2.04902500	-0.77525300
C	1.17808100	-2.04956900	-2.03435400
C	1.98615700	-3.41868400	-0.09870800
C	2.00887700	0.45571400	-0.31540400
C	3.52503700	0.67692300	-0.43563400
C	1.50884800	1.53801300	0.62972100
O	4.30471000	0.03232100	0.22364400
O	1.74062400	1.55336100	1.81349400
O	0.80161500	2.47547300	0.00357200
C	0.26992700	3.51711100	0.84400000
C	3.95267600	1.74720800	-1.39868100
H	-4.16452700	-0.78842300	1.44038400
H	-0.53243100	0.40771500	-1.78962900
H	-2.87346000	0.99785500	-2.24839200
H	0.49633900	-2.06681800	2.65047800
H	-1.99544500	-1.85601900	2.87677600
H	2.30763200	-1.07427900	1.11305900
H	3.08798300	-1.86234400	-1.06866100
H	1.22930900	-1.10247500	-2.58407300
H	1.50912500	-2.84033600	-2.71673400
H	0.12936100	-2.24359300	-1.77890300
H	0.95322200	-3.66414500	0.17675500
H	2.35097300	-4.19950600	-0.77513800
H	2.59585800	-3.44070500	0.81262200
H	1.56579100	0.64479600	-1.29713900
H	1.08667400	4.03803600	1.34932600
H	-0.26946800	4.19079600	0.17954300
H	-0.40397100	3.07861500	1.58445400
H	5.00706900	1.98946100	-1.26162700
H	3.78697300	1.37739000	-2.41809900
H	3.32801400	2.63929200	-1.27597600
Cl	-5.27216000	0.57624600	-0.80783200

Compound **1b**, optimization level PCM-M11/6-311++G(2d,p)

Conformation #17

C	3.13810400	0.25704300	1.17246200
C	1.78411000	0.55441900	1.32585300
C	0.86735500	0.53804400	0.24934400
C	1.35021300	0.20674200	-1.02518700
C	2.68640800	-0.08679300	-1.19549000
C	3.55478700	-0.05612200	-0.09767400
C	-0.42322200	0.89335800	0.79499200
C	-0.22376400	1.09988100	2.12769400
N	1.09392200	0.89854500	2.45550300
C	-1.74394100	1.06654200	0.08789800
C	-1.62480000	1.92841200	-1.19035500
C	-1.02595600	3.29109400	-0.83387800
C	-2.97904800	2.11449500	-1.87981000
C	-2.46048200	-0.28519800	-0.15321700
C	-2.96948200	-0.92751900	1.13706600
C	-1.61003700	-1.21602000	-0.98934400
O	-2.99251500	-0.30537400	2.17395300
O	-1.53062200	-1.15309100	-2.19440600
O	-0.92000900	-2.08574000	-0.25572900
C	-0.06617900	-2.98839600	-0.98340800
C	-3.52175200	-2.32863800	1.04958500
H	3.83000900	0.27102400	2.00903800
H	0.68771900	0.15836500	-1.88522100
H	3.07582200	-0.34619500	-2.17534400
H	-0.94933200	1.37658900	2.88207500
H	1.48665500	0.98782400	3.38034400
H	-2.40397600	1.61018600	0.77731400
H	-0.95388300	1.42830400	-1.89826500
H	-0.94894300	3.92244000	-1.72562600
H	-1.66824400	3.80782000	-0.10755700
H	-0.02824400	3.19221700	-0.39419200
H	-3.73979600	2.44153100	-1.15762400
H	-2.90458000	2.88186100	-2.65774500
H	-3.32859800	1.19510700	-2.36095200
H	-3.36718800	-0.10411300	-0.74501700
H	0.68930700	-2.41807500	-1.52922700
H	0.39836600	-3.62830500	-0.23410800
H	-0.66564000	-3.57775900	-1.68173700
H	-4.39940400	-2.40766800	1.69486400
H	-3.77667600	-2.60565100	0.02267200
H	-2.75706700	-3.02311000	1.41204900
Cl	5.24809000	-0.43921400	-0.36376400

Compound **1b**, optimization level PCM-M11/6-311++G(2d,p)

Conformation #20

C	3.64160700	0.51874700	0.81113400
C	2.25685800	0.45850700	0.97485300
C	1.42083400	-0.22076400	0.06512200
C	1.99037900	-0.86066100	-1.04109600
C	3.35600300	-0.81227300	-1.21707400
C	4.15233000	-0.12519900	-0.28969900
C	0.06902900	-0.07023300	0.53335600
C	0.14533200	0.66912300	1.67810900
N	1.45663300	0.99069000	1.94853400
C	-1.14417700	-0.67668700	-0.11774600
C	-1.21314900	-2.19516400	0.20571700
C	-2.16332000	-2.97621000	-0.70527900
C	-1.54405800	-2.46944800	1.67381200
C	-2.41786100	0.08917000	0.27812900
C	-3.62760400	-0.28223700	-0.59660800
C	-2.25194200	1.59449400	0.11956800
O	-3.51421200	-0.36034500	-1.79619600
O	-2.63890500	2.40172100	0.93192500
O	-1.66700700	1.92535100	-1.02452100
C	-1.47002200	3.33473400	-1.23818700
C	-4.92622500	-0.48481100	0.12723700
H	4.28651500	1.04311400	1.50953800
H	1.36726100	-1.39167100	-1.75826000
H	3.82742900	-1.29874900	-2.06544300
H	-0.64484600	1.00475000	2.33986000
H	1.76932700	1.54226900	2.73308200
H	-1.04014600	-0.56995100	-1.20613600
H	-0.19718300	-2.56747600	0.01564700
H	-2.01722300	-2.71960800	-1.76011500
H	-3.21458800	-2.78907900	-0.45354900
H	-1.99386600	-4.05155900	-0.58089400
H	-0.92173700	-1.87121400	2.34876700
H	-2.59815500	-2.24904700	1.88911800
H	-1.38258400	-3.52725700	1.90724400
H	-2.67143900	-0.06909700	1.33071300
H	-2.43499700	3.84742100	-1.23931500
H	-0.83716300	3.73993300	-0.44430500
H	-0.98122500	3.42469400	-2.20736600
H	-5.73785000	-0.67543800	-0.57572900
H	-4.82082000	-1.32519700	0.82475500
H	-5.14018100	0.40463300	0.73252600
Cl	5.88846700	-0.08921500	-0.55515500

Compound **1b**, optimization level PCM-M11/6-311++G(2d,p)

Conformation #21

C	3.53203400	0.50184500	0.90631700
C	2.16284800	0.28064300	1.06439100
C	1.36234400	-0.26229100	0.03806600
C	1.95422800	-0.58613800	-1.18778700
C	3.30400800	-0.37323100	-1.36194000
C	4.06508200	0.16314100	-0.31331900
C	0.01774300	-0.34583800	0.54281500
C	0.06317300	0.14456600	1.81694700
N	1.34784000	0.51849100	2.13724700
C	-1.18143100	-0.86429400	-0.20609100
C	-1.49848600	-2.35025500	0.09970800
C	-1.95077100	-2.58389700	1.54161500
C	-0.28807300	-3.22730200	-0.22231000
C	-2.39188200	0.04398700	0.04684600
C	-3.61436500	-0.34949500	-0.79676500
C	-2.06189600	1.47560000	-0.35083100
O	-3.47056400	-0.78229100	-1.91556700
O	-1.41390800	1.77883600	-1.32072100
O	-2.60602800	2.36372300	0.48068600
C	-2.37590300	3.74682200	0.14877100
C	-4.95566600	-0.13430600	-0.16002200
H	4.14772200	0.91895700	1.69738200
H	1.36084800	-0.99999200	-2.00026200
H	3.79012800	-0.61425900	-2.30229000
H	-0.73316300	0.26467500	2.54231800
H	1.63496200	0.91342800	3.01996400
H	-0.96301700	-0.79347200	-1.27940800
H	-2.31533200	-2.64156400	-0.57301800
H	-1.17353300	-2.26544600	2.24723400
H	-2.87721300	-2.05086300	1.78609900
H	-2.13505600	-3.65087400	1.70821100
H	0.54403700	-2.99883900	0.45528100
H	-0.53989600	-4.28712500	-0.10615500
H	0.05794300	-3.06882700	-1.25069400
H	-2.68397900	0.05828600	1.10140900
H	-2.88748400	4.32700700	0.91552500
H	-1.30272900	3.95185200	0.15615500
H	-2.78606900	3.96105100	-0.84105700
H	-5.75703400	-0.29018200	-0.88301000
H	-5.06227900	-0.83813900	0.67495100
H	-5.00249200	0.87524100	0.26453700
Cl	5.78322500	0.41412600	-0.58162000

Compound **1b**, optimization level PCM-M11/6-311++G(2d,p)

Conformation #22

C	3.18371600	-1.07877600	-0.62423000
C	1.83466200	-1.39745800	-0.46855500
C	0.91923500	-0.55304500	0.20025600
C	1.39835300	0.65316700	0.73099900
C	2.72800400	0.98516300	0.58586000
C	3.59518900	0.11611600	-0.08782100
C	-0.36606800	-1.21108400	0.15584700
C	-0.16597400	-2.38174500	-0.51463800
N	1.14823900	-2.50218500	-0.89190700
C	-1.68858400	-0.75751200	0.72055000
C	-1.58180800	-0.23523600	2.17164200
C	-0.96670700	-1.32120600	3.05774700
C	-2.94705900	0.18200800	2.72498900
C	-2.41596700	0.21189900	-0.24478100
C	-2.98014700	-0.50266100	-1.47570500
C	-1.54103300	1.37703800	-0.65276200
O	-3.03461200	-1.71005800	-1.52162300
O	-0.92951100	1.45307500	-1.69053500
O	-1.49489500	2.32054700	0.29161000
C	-0.67972500	3.46738600	-0.01940700
C	-3.53064200	0.35337900	-2.58626900
H	3.87592700	-1.73660000	-1.14106100
H	0.73947300	1.33837400	1.25610500
H	3.11440600	1.91577300	0.99051400
H	-0.88863600	-3.14958100	-0.76010800
H	1.53986100	-3.27742300	-1.40492000
H	-2.33735600	-1.64274600	0.75613800
H	-0.92880700	0.64352000	2.19567800
H	-1.59374800	-2.22314600	3.04035400
H	0.03666500	-1.59914000	2.71912400
H	-0.89777800	-0.97782900	4.09557200
H	-2.88028400	0.35500100	3.80441000
H	-3.31558300	1.10743200	2.26979900
H	-3.69115000	-0.60870600	2.55712300
H	-3.29518200	0.63080400	0.26210700
H	-1.09236500	3.98415600	-0.88945000
H	-0.71340100	4.10581000	0.86270100
H	0.34380300	3.14860800	-0.23403900
H	-4.33743100	-0.18216600	-3.08994700
H	-3.88179100	1.32023600	-2.21357700
H	-2.72414300	0.54467000	-3.30148300
Cl	5.28172500	0.58030200	-0.24762600

Compound **1b**, optimization level PCM-M11/6-311++G(2d,p)

Conformation #23

C	-3.39592800	0.58583500	0.79148400
C	-2.03637600	0.85718000	0.95995400
C	-1.08028400	0.56778100	-0.03516100
C	-1.50291300	-0.00604100	-1.24042300
C	-2.83963000	-0.27820100	-1.42428600
C	-3.75813900	0.02001700	-0.40581100
C	0.20393100	0.95812000	0.48089100
C	-0.02656200	1.45650300	1.73121600
N	-1.36893900	1.40123900	2.02305800
C	1.53831700	0.79050400	-0.20148100
C	2.36167200	2.09671600	-0.14284200
C	1.63343200	3.20348000	-0.90833100
C	3.78132300	1.94145900	-0.68981500
C	2.31882000	-0.40094500	0.40807600
C	3.31506800	-1.04365500	-0.56692900
C	1.35039200	-1.49419100	0.83353300
O	3.15065800	-0.98911000	-1.76195900
O	1.18703800	-1.87180500	1.96780100
O	0.67809400	-1.97532100	-0.20913500
C	-0.39109000	-2.88630700	0.10191000
C	4.46704700	-1.76983400	0.06952900
H	-4.13090600	0.80456900	1.56002700
H	-0.78359500	-0.25080400	-2.01906500
H	-3.19649400	-0.72628100	-2.34669500
H	0.67237800	1.85291700	2.45788600
H	-1.78900800	1.70951400	2.88694400
H	1.36047400	0.53804300	-1.25579200
H	2.43402900	2.38878400	0.91566200
H	2.17969700	4.15008000	-0.83367200
H	1.56245700	2.93835500	-1.97160000
H	0.61951700	3.35829600	-0.52606300
H	3.76042100	1.59811000	-1.73075300
H	4.29909100	2.90623900	-0.65821100
H	4.38272300	1.23423600	-0.10591300
H	2.84876400	-0.09542900	1.31757300
H	-1.12981400	-2.37345900	0.72632000
H	-0.83048800	-3.16869500	-0.85437700
H	0.00311700	-3.75995300	0.62623000
H	5.03640700	-2.32081800	-0.67968700
H	5.11451800	-1.03887500	0.56897300
H	4.09393700	-2.44788500	0.84620700
Cl	-5.45208600	-0.35331000	-0.68400900

Compound **1b**, optimization level PCM-M11/6-311++G(2d,p)

Conformation #37

C	3.53440700	-0.77789400	-0.98127600
C	2.14808500	-0.62091100	-0.99763800
C	1.46657900	0.17329500	-0.05258000
C	2.20199600	0.83001100	0.94085800
C	3.57194500	0.68438900	0.97337100
C	4.21001000	-0.11394800	0.01382200
C	0.06220800	0.10303200	-0.36569800
C	-0.03912400	-0.71107300	-1.45856700
N	1.20777800	-1.14587900	-1.84103300
C	-1.02446200	0.81373200	0.40120100
C	-1.48279200	2.12246100	-0.28719000
C	-0.30183300	3.08752200	-0.40507500
C	-2.62773500	2.78864900	0.47939100
C	-2.21463200	-0.09929700	0.79213100
C	-1.73238200	-1.46757300	1.27176100
C	-3.25035100	-0.31032400	-0.29174800
O	-2.12729000	-2.48730500	0.75256700
O	-3.04371600	-0.30200400	-1.48051300
O	-4.45596200	-0.52868700	0.23467700
C	-5.50631000	-0.80559800	-0.70893700
C	-0.75241900	-1.46432000	2.41201200
H	4.05767500	-1.38958300	-1.70992900
H	1.70681700	1.45391300	1.68277300
H	4.16763300	1.18184100	1.73260500
H	-0.92370700	-1.01902200	-1.99896200
H	1.39194100	-1.76338700	-2.61717600
H	-0.57967000	1.11750900	1.36193900
H	-1.82761400	1.86264900	-1.29523700
H	0.07344600	3.35419800	0.59283000
H	0.52458300	2.64807400	-0.97296300
H	-0.61001100	4.01173600	-0.90578400
H	-2.34572400	2.94494000	1.52997000
H	-2.85583000	3.76785900	0.04526600
H	-3.55195800	2.19905200	0.46021500
H	-2.73329300	0.35477900	1.64594500
H	-5.63638200	0.04861300	-1.37803300
H	-5.25290700	-1.69486800	-1.29130000
H	-6.40473700	-0.97314100	-0.11622700
H	-0.66408900	-2.46941700	2.82658000
H	0.22528700	-1.14011600	2.03498800
H	-1.06360700	-0.75365300	3.18448300
Cl	5.95790000	-0.26880900	0.09449300

Compound **1b**, optimization level PCM-M11/6-311++G(2d,p)

Conformation #44

C	-3.37976800	-0.15715100	0.84540900
C	-2.03589400	-0.34023800	1.17664300
C	-1.02728900	-0.49935000	0.20239600
C	-1.38732600	-0.45552500	-1.15077100
C	-2.70815400	-0.27651200	-1.49652000
C	-3.67828000	-0.13379300	-0.49423800
C	0.22359600	-0.62842300	0.90856600
C	-0.08096100	-0.54262600	2.23492500
N	-1.43391100	-0.37425500	2.40573500
C	1.60387700	-0.81925300	0.33717400
C	1.68858900	-2.10165900	-0.52307900
C	1.30806300	-3.32260700	0.31598700
C	3.07440700	-2.30354600	-1.13796800
C	2.09184900	0.42519700	-0.45831300
C	3.61234400	0.60924600	-0.41242000
C	1.48382500	1.66521100	0.17297000
O	4.27229200	0.17660900	0.50141400
O	1.88231400	2.16914100	1.19524700
O	0.41391100	2.08872100	-0.49183700
C	-0.42085300	3.02651600	0.20989600
C	4.20749800	1.39595800	-1.54671700
H	-4.15052200	-0.03313100	1.60011100
H	-0.63694700	-0.54099400	-1.93311400
H	-3.01030900	-0.23706400	-2.53856800
H	0.58216400	-0.59169600	3.09021100
H	-1.90262500	-0.28515500	3.29449400
H	2.30024500	-0.92703200	1.17992800
H	0.96100800	-2.00441400	-1.34120000
H	1.99078300	-3.42028800	1.17091700
H	0.28747500	-3.24313700	0.70345900
H	1.38152500	-4.23796400	-0.28117700
H	3.10288200	-3.24552600	-1.69607000
H	3.34321400	-1.50512400	-1.84032700
H	3.84165900	-2.34464800	-0.35549400
H	1.75936100	0.37768400	-1.50224900
H	0.11980100	3.96010300	0.38270000
H	-1.28828700	3.19057900	-0.42940000
H	-0.72207300	2.58576800	1.16628000
H	5.26411700	1.59379900	-1.36359800
H	4.08889700	0.82623000	-2.47639800
H	3.65874500	2.33632100	-1.67550100
Cl	-5.34911800	0.09653200	-0.98455200

Compound **1b**, optimization level PCM-ωB97-XD/6-311++G(2d,p)

Conformation #1

C	-3.76386700	-0.69444500	-0.17224600
C	-2.43410900	-1.09763000	-0.06071400
C	-1.40938400	-0.21933500	0.34959700
C	-1.73266200	1.10777200	0.65133300
C	-3.04292400	1.52540600	0.54354800
C	-4.03175100	0.62016700	0.13524900
C	-0.18276300	-0.96668700	0.35478800
C	-0.50979600	-2.22858200	-0.04716600
N	-1.85829700	-2.31466800	-0.29575200
C	1.18135500	-0.39393600	0.64012000
C	2.16371300	-1.44478300	1.19914100
C	1.66795000	-1.93846300	2.56104600
C	3.59628700	-0.93222400	1.33869000
C	1.62420400	0.37859100	-0.64115300
C	1.82486500	-0.55267300	-1.83789900
C	2.79559500	1.32782600	-0.48509400
O	2.69630300	-1.39026300	-1.84259900
O	3.68656700	1.46726700	-1.28615000
O	2.67642600	2.06146600	0.61779100
C	3.71564600	3.01682500	0.85712900
C	0.86089400	-0.38298100	-2.97439500
H	-4.54558000	-1.37406600	-0.48499300
H	-0.96894100	1.80804400	0.97065300
H	-3.31601400	2.54701900	0.77239200
H	0.11768700	-3.09594500	-0.17883900
H	-2.34051800	-3.14818000	-0.58402700
H	1.06314600	0.37475200	1.41095700
H	2.18823300	-2.29349800	0.50988600
H	1.67269800	-1.11905200	3.28711700
H	0.65447900	-2.33826700	2.51035000
H	2.32400900	-2.72457500	2.94150300
H	3.63733600	-0.04272000	1.97351000
H	4.21823400	-1.70055400	1.80369900
H	4.04174600	-0.69650200	0.37159100
H	0.78900100	1.04611800	-0.87898000
H	4.67502800	2.50908300	0.95535000
H	3.76333500	3.73842200	0.04243900
H	3.45016600	3.50992600	1.78785800
H	1.05358800	-1.11095800	-3.75976700
H	-0.16007200	-0.49248600	-2.59648700
H	0.94581600	0.63256600	-3.37208400
Cl	-5.68642300	1.19510000	0.01199800

Compound **1b**, optimization level PCM-ωB97-XD /6-311++G(2d,p)

Conformation #2

C	-3.47214300	-0.08286800	0.99938500
C	-2.10116600	0.12596900	1.14345300
C	-1.24541700	0.33049200	0.04071100
C	-1.78822900	0.31991300	-1.24790800
C	-3.14219000	0.11324100	-1.41089000
C	-3.95789100	-0.08276500	-0.28813200
C	0.08343300	0.50415500	0.55688000
C	-0.02506700	0.39558400	1.91377700
N	-1.33161100	0.17209400	2.27201300
C	1.32391900	0.74163000	-0.25980600
C	1.73895100	2.23363600	-0.33448000
C	2.09112700	2.85235700	1.01682400
C	0.65201800	3.05319500	-1.02784700
C	2.47920600	-0.15492100	0.21778000
C	3.67710100	-0.15667000	-0.74653400
C	2.04619900	-1.60303400	0.39940200
O	3.52442200	-0.01693800	-1.93615600
O	2.31180400	-2.26990400	1.36924200
O	1.36352200	-2.05164400	-0.64538900
C	0.86783400	-3.39194100	-0.55363500
C	5.02313700	-0.35550200	-0.11565800
H	-4.12124700	-0.23895600	1.85102100
H	-1.15866700	0.47367100	-2.11648300
H	-3.58245600	0.10121600	-2.39922000
H	0.73874100	0.45244400	2.67368200
H	-1.66011500	0.04887400	3.21387600
H	1.10456800	0.43283200	-1.28465300
H	2.63168500	2.27551500	-0.96650000
H	1.22442200	2.86040500	1.68267300
H	2.90329400	2.32507400	1.52274300
H	2.41353700	3.88680100	0.87819200
H	-0.26777700	3.06356300	-0.43649600
H	0.97802600	4.08741300	-1.16011700
H	0.41465900	2.64486900	-2.01356800
H	2.83899800	0.15200000	1.19999900
H	1.69319600	-4.09462000	-0.44487500
H	0.19168400	-3.48206900	0.29607000
H	0.33520400	-3.56999700	-1.48327000
H	5.78721900	-0.50727300	-0.87497800
H	5.26343000	0.53079800	0.47915200
H	4.99456000	-1.20183600	0.57507700
Cl	-5.67674100	-0.34072500	-0.54223100

Compound **1b**, optimization level PCM-ωB97-XD /6-311++G(2d,p)

Conformation #3

C	-3.81497700	0.48130800	0.53157000
C	-2.49460800	0.92810400	0.51615100
C	-1.49641500	0.31226700	-0.26784000
C	-1.83705000	-0.79227200	-1.05561600
C	-3.13821900	-1.25059000	-1.05038400
C	-4.10063200	-0.60783900	-0.26009700
C	-0.27284000	1.03040300	-0.04289400
C	-0.57537700	2.01788600	0.84836900
N	-1.90613600	1.96401700	1.18564300
C	1.07282600	0.65841700	-0.60871400
C	2.01252300	1.87421800	-0.75374000
C	1.43486400	2.84990800	-1.78259900
C	3.44064700	1.50621100	-1.15253200
C	1.60223000	-0.54569000	0.22549400
C	1.90336200	-0.16537400	1.67635700
C	2.70684900	-1.32409600	-0.46426800
O	2.75461300	0.64609300	1.95713300
O	2.67908500	-1.61280100	-1.63455800
O	3.66069200	-1.72109200	0.37109400
C	4.71068600	-2.51405100	-0.19656500
C	1.04571700	-0.82535000	2.71556100
H	-4.57628400	0.96070300	1.13288200
H	-1.09374300	-1.28840000	-1.66952300
H	-3.42439800	-2.10289000	-1.65234400
H	0.05834000	2.77634800	1.28016900
H	-2.36973400	2.60068400	1.81004900
H	0.91534100	0.25149000	-1.61399100
H	2.06761100	2.38198300	0.21316300
H	1.40532700	2.38463600	-2.77313300
H	0.42216700	3.16463500	-1.52741900
H	2.06014300	3.74288200	-1.85222200
H	3.45826000	0.95666400	-2.09717100
H	4.03082100	2.41631400	-1.28328000
H	3.93635300	0.90625300	-0.38786800
H	0.77963300	-1.26844800	0.24613800
H	5.22867000	-1.94916300	-0.97093200
H	5.38382600	-2.73743700	0.62600200
H	4.30475500	-3.43232200	-0.61926400
H	-0.00353800	-0.59540800	2.50544700
H	1.15391100	-1.91119100	2.64345900
H	1.31308900	-0.48536600	3.71391300
Cl	-5.74472000	-1.22461500	-0.28485000

Compound **1b**, optimization level PCM-ωB97-XD /6-311++G(2d,p)

Conformation #5

```
C                  3.51070500    0.43440300    0.86771900
 C                  2.14079700    0.25584200    1.05521700
 C                  1.30199500   -0.28135700    0.05638300
 C                  1.86178500   -0.64724000   -1.17188700
 C                  3.21504400   -0.47742300   -1.37657100
 C                  4.01354600    0.05742600   -0.35635900
 C                 -0.03054200   -0.31511600    0.59131200
 C                  0.05944200    0.19378400    1.85545100
 N                  1.35778300    0.53489100    2.14017000
 C                 -1.25636500   -0.81428800   -0.12333300
 C                 -1.63150800   -2.27450100    0.23440000
 C                 -2.01427400   -2.47061100    1.69952800
 C                 -0.50777000   -3.22944400   -0.16264400
 C                 -2.43754900    0.15935700    0.08863100
 C                 -3.70379700   -0.27134500   -0.66220300
 C                 -2.03323300    1.55670600   -0.35002600
 O                 -4.73447700   -0.42793500   -0.04820100
 O                 -1.51639600    1.82263500   -1.40700000
 O                 -2.33116000    2.46150000    0.57601700
 C                 -1.98118100    3.81737200    0.26782700
 C                 -3.61352700   -0.49783600   -2.14451300
 H                  4.14611200    0.84730100    1.64014400
 H                  1.24648700   -1.06246800   -1.96143400
 H                  3.66814100   -0.75391900   -2.31943200
 H                 -0.71162000    0.35125400    2.59334400
 H                  1.67311200    0.93690700    3.00573700
 H                 -1.02674600   -0.79962400   -1.19309700
 H                 -2.50670900   -2.53635800   -0.37155300
 H                 -2.29353700   -3.51193900    1.87400600
 H                 -1.17417400   -2.23576900    2.35790300
 H                 -2.86379700   -1.85019600    1.99318900
 H                  0.39208900   -3.04185300    0.42913700
 H                 -0.81094500   -4.26529900    0.00461900
 H                 -0.24665000   -3.11976800   -1.21831300
 H                 -2.71443700    0.20723900    1.14030600
 H                 -0.90575900    3.90052300    0.11492400
 H                 -2.50741500    4.14873700   -0.62654900
 H                 -2.28978500    4.40113000    1.13008900
 H                 -3.08560700    0.32491900   -2.62827500
 H                 -3.03395100   -1.40474100   -2.33724000
 H                 -4.61109900   -0.61749700   -2.56136400
 Cl                 5.73215600    0.25371900   -0.66043800
```

Compound **1b**, optimization level PCM-ωB97-XD /6-311++G(2d,p)

Conformation #7

C	-3.38065100	-0.34181400	0.86583000
C	-2.03702400	-0.58309100	1.14970400
C	-1.01444400	-0.47996400	0.17941000
C	-1.37853700	-0.11351200	-1.12347200
C	-2.70112200	0.13289100	-1.42336000
C	-3.67913900	0.01462400	-0.42764300
C	0.22681200	-0.78844300	0.84754300
C	-0.10282400	-1.04881400	2.14576100
N	-1.45398800	-0.93734500	2.33361300
C	1.63983200	-0.81628900	0.32718200
C	2.00369800	-2.09089400	-0.47437400
C	1.19155900	-2.28707300	-1.75241500
C	1.90635400	-3.32619500	0.41783700
C	1.97978400	0.47809600	-0.45289200
C	3.45229900	0.54348400	-0.87573100
C	1.63171800	1.68919000	0.39488500
O	3.72544400	0.72636500	-2.04038900
O	2.06942000	1.90552400	1.49822000
O	0.77389500	2.48705500	-0.23016500
C	0.33079700	3.63654200	0.50224800
C	4.51148900	0.36377000	0.17398000
H	-4.14820800	-0.42851900	1.62380300
H	-0.63947400	-0.02324700	-1.90811800
H	-2.99084000	0.41678300	-2.42644600
H	0.54521300	-1.30825800	2.96882200
H	-1.93211400	-1.07021600	3.20770000
H	2.28169400	-0.81724300	1.21347800
H	3.05472000	-1.98876900	-0.76913400
H	0.13406400	-2.44102000	-1.52594100
H	1.27724200	-1.43789600	-2.43409800
H	1.54954400	-3.17002100	-2.28647400
H	0.87175000	-3.50707500	0.72068800
H	2.26201500	-4.21064600	-0.11528300
H	2.50749800	-3.21086400	1.32362900
H	1.40435800	0.54130200	-1.37315400
H	-0.16861100	3.32530400	1.41921700
H	1.17566600	4.28088300	0.74244500
H	-0.36548700	4.14945700	-0.15474600
H	4.54433600	-0.68718800	0.47475100
H	5.48157300	0.64710200	-0.22867700
H	4.27206100	0.94525300	1.06487200
Cl	-5.35133900	0.33586500	-0.85534800

Compound **1b**, optimization level PCM-ωB97-XD /6-311++G(2d,p)

Conformation #8

C	3.43864100	-0.02519100	0.84608700
C	2.10778500	0.22657400	1.17700300
C	1.08860500	0.37215900	0.20839200
C	1.44133300	0.24982200	-1.14232200
C	2.75121300	-0.00255900	-1.48985700
C	3.72698100	-0.13422800	-0.49358800
C	-0.14042600	0.60909600	0.92569300
C	0.19152000	0.58936000	2.24880200
N	1.53413000	0.36999000	2.40858800
C	-1.54158600	0.82444900	0.41697300
C	-1.81601800	2.25998500	-0.10108700
C	-0.97175300	2.67609000	-1.30393600
C	-1.66006700	3.27248800	1.03159400
C	-1.94999200	-0.24519400	-0.61408800
C	-3.45983900	-0.24737100	-0.90590100
C	-1.57110700	-1.65015400	-0.16758600
O	-4.26528300	0.08113800	-0.06880200
O	-0.93597700	-2.42494900	-0.83918200
O	-2.04078100	-1.92816300	1.04172600
C	-1.70182400	-3.21357900	1.57395600
C	-3.85298500	-0.69083500	-2.28413200
H	4.20439200	-0.13157400	1.60333000
H	0.70279800	0.35384800	-1.92602900
H	3.03272100	-0.09917900	-2.53011400
H	-0.44870400	0.71525900	3.10841700
H	2.01154400	0.30402900	3.29062800
H	-2.20699500	0.68335500	1.27223600
H	-2.86634200	2.27874300	-0.40869700
H	-1.26878500	3.67308200	-1.63741100
H	0.08963900	2.71384000	-1.04769900
H	-1.09421500	2.00197300	-2.15492800
H	-0.62096200	3.32677400	1.36714700
H	-1.95887200	4.26865800	0.69716000
H	-2.27824000	3.00386600	1.89227200
H	-1.43176100	-0.10579100	-1.56177100
H	-0.61958600	-3.30970800	1.65706100
H	-2.16348900	-3.25390200	2.55625300
H	-2.09418300	-4.00363500	0.93465400
H	-3.52397900	0.06852000	-2.99986900
H	-3.33595000	-1.61721200	-2.54643900
H	-4.93054500	-0.82167100	-2.35567600
Cl	5.38329600	-0.45228700	-0.98215200

Compound **1b**, optimization level PCM-ωB97-XD /6-311++G(2d,p)

Conformation #9

C	3.49051500	-0.07092200	0.99274300
C	2.11824900	-0.27043800	1.13741900
C	1.24042900	-0.33159300	0.03482700
C	1.76212000	-0.18357100	-1.25419400
C	3.11697200	0.01610300	-1.41743000
C	3.95493300	0.06776400	-0.29508900
C	-0.08234300	-0.54009200	0.55394800
C	0.05081900	-0.58985100	1.91218600
N	1.36687700	-0.43266400	2.26767800
C	-1.33984700	-0.66302800	-0.26138500
C	-1.78948000	-2.12562200	-0.50192300
C	-2.18667400	-2.86689900	0.77237000
C	-0.71285600	-2.89688100	-1.26254100
C	-2.46452400	0.19933300	0.35650700
C	-3.77438300	0.12611000	-0.43984800
C	-2.03287300	1.64526500	0.53930600
O	-4.77611300	-0.27737100	0.10534200
O	-2.26533500	2.28890900	1.53263600
O	-1.38686300	2.12406300	-0.51748100
C	-0.92315900	3.47602700	-0.42331200
C	-3.77087900	0.55618400	-1.87946100
H	4.15637500	-0.02550000	1.84443700
H	1.11637200	-0.22531100	-2.12352500
H	3.54113400	0.13317000	-2.40592200
H	-0.70023600	-0.72082500	2.67547800
H	1.71315500	-0.42311000	3.21126600
H	-1.11766400	-0.24177400	-1.24527200
H	-2.67372500	-2.08150500	-1.14925700
H	-1.33810700	-2.94917400	1.45633400
H	-3.00641300	-2.37481000	1.30010100
H	-2.51548100	-3.87897800	0.52657400
H	0.19177100	-3.00322900	-0.65788900
H	-1.06802100	-3.89801000	-1.51646000
H	-0.44005000	-2.38888600	-2.19116600
H	-2.71161200	-0.15626800	1.35550400
H	-0.22666000	3.57448800	0.40858300
H	-0.42147800	3.67802000	-1.36519400
H	-1.76346500	4.15514700	-0.28375200
H	-4.75172800	0.38456100	-2.31686600
H	-3.51221100	1.61504300	-1.94780900
H	-3.01166600	0.00970000	-2.44283100
Cl	5.67387800	0.32198600	-0.55028200

Compound **1b**, optimization level PCM-ωB97-XD /6-311++G(2d,p)

Conformation #14

C	-3.43440600	-0.08959800	1.18311300
C	-2.07392500	0.21519800	1.19258900
C	-1.32902200	0.40221800	0.00851700
C	-1.97855900	0.27667900	-1.22438100
C	-3.32436500	-0.02317400	-1.25374700
C	-4.02655700	-0.20109300	-0.05385200
C	0.02271600	0.69694700	0.39694300
C	0.03452600	0.67762200	1.76117100
N	-1.21799200	0.38992900	2.24350000
C	1.16768200	0.96047200	-0.54535400
C	1.96337200	2.23421200	-0.18018300
C	1.04803800	3.45553200	-0.27029900
C	3.18718600	2.43500100	-1.07328600
C	2.05698600	-0.28936900	-0.78472000
C	1.16424300	-1.47112400	-1.19250700
C	2.99715900	-0.65074500	0.34700600
O	0.74674000	-1.51910900	-2.32642100
O	2.95373900	-0.24520100	1.48054200
O	3.91733300	-1.51521600	-0.08630700
C	4.85634100	-1.98354300	0.88865400
C	0.81059100	-2.50329100	-0.16209900
H	-3.99664300	-0.23200200	2.09666200
H	-1.43477300	0.40315700	-2.15308700
H	-3.84471400	-0.12583500	-2.19688100
H	0.85678400	0.83833100	2.43826000
H	-1.46098700	0.32448400	3.21671100
H	0.72548900	1.15147600	-1.53020600
H	2.30652900	2.13504300	0.85290700
H	0.70990600	3.60604900	-1.30075200
H	0.16573400	3.34478000	0.36236300
H	1.57991900	4.35675300	0.04269100
H	2.90343200	2.43012800	-2.13094000
H	3.65425700	3.39858400	-0.85885300
H	3.94928200	1.66695000	-0.92404300
H	2.68274300	-0.10081200	-1.65861300
H	4.33552700	-2.49741300	1.69632200
H	5.50915400	-2.67131300	0.35904000
H	5.42850500	-1.14925800	1.29278800
H	0.03039300	-3.15956000	-0.54116100
H	1.70005900	-3.09450100	0.07393500
H	0.48613100	-2.01580000	0.75986200
Cl	-5.73854900	-0.58554100	-0.13937600

Compound **1b**, optimization level PCM-ωB97-XD/6-311++G(2d,p)

Conformation #15

C	-3.37935200	-0.44388100	0.78298400
C	-2.04617200	-0.74376000	1.06093900
C	-0.99823200	-0.51599800	0.14017400
C	-1.32444100	0.03923500	-1.10445300
C	-2.63610000	0.34481800	-1.39767200
C	-3.64039900	0.09903300	-0.45252100
C	0.22348600	-0.93331100	0.78430400
C	-0.14130900	-1.37512200	2.02239700
N	-1.49634100	-1.27373800	2.19374100
C	1.64666100	-0.90951500	0.29190100
C	2.00974800	-2.08050700	-0.65658200
C	1.20097500	-2.11920900	-1.95175300
C	1.89943200	-3.41478200	0.07809400
C	2.02885500	0.45522800	-0.31491300
C	3.54352700	0.60595700	-0.51943700
C	1.59121000	1.58526900	0.60457600
O	4.33408600	0.08807900	0.23168300
O	1.93755400	1.69888800	1.75311500
O	0.78105600	2.43645700	-0.01568700
C	0.26478200	3.51070200	0.77986700
C	3.96262000	1.44054000	-1.69356400
H	-4.16731600	-0.62598100	1.50213400
H	-0.56316700	0.23318800	-1.84805200
H	-2.89719700	0.77443500	-2.35580700
H	0.48334300	-1.75965800	2.81393000
H	-1.99699100	-1.52685100	3.02768900
H	2.28169900	-1.02541300	1.17416600
H	3.06271200	-1.94477300	-0.92425600
H	1.29144800	-1.19596800	-2.52899400
H	1.55638000	-2.93369500	-2.58706300
H	0.14128000	-2.29231200	-1.74940400
H	0.86200500	-3.62586600	0.35101700
H	2.25375800	-4.23057500	-0.55626600
H	2.49542700	-3.41390400	0.99446100
H	1.54279100	0.61470000	-1.27608500
H	1.07912100	4.12944000	1.15502200
H	-0.37525900	4.08387200	0.11545800
H	-0.31126900	3.11271400	1.61472000
H	5.03525800	1.62092000	-1.67696900
H	3.68990600	0.91130800	-2.61162200
H	3.41589100	2.38693100	-1.69993100
Cl	-5.29757400	0.50400500	-0.86878100

Compound **1b**, optimization level PCM-ωB97-XD/6-311++G(2d,p)

Conformation #17

C	3.12387000	0.07031600	1.20215300
C	1.76892000	0.34727500	1.37346300
C	0.87936800	0.52824100	0.28994600
C	1.39330100	0.43307400	-1.00952500
C	2.73262500	0.16100700	-1.19956300
C	3.57337200	-0.01738000	-0.09502100
C	-0.42135400	0.79098700	0.85466000
C	-0.25399100	0.76192500	2.20678700
N	1.05236800	0.49449400	2.52746500
C	-1.73619400	1.04007700	0.16188700
C	-1.64576400	2.07202700	-0.98739500
C	-0.94083800	3.34183700	-0.50894600
C	-3.02498700	2.43439000	-1.54206600
C	-2.43632200	-0.28064100	-0.25920300
C	-3.10254800	-1.01453200	0.90780700
C	-1.52987500	-1.16719900	-1.08793900
O	-3.19863500	-0.50780900	2.00068800
O	-1.39067300	-1.05647200	-2.28229200
O	-0.87379000	-2.05282000	-0.34696200
C	0.07012300	-2.88779700	-1.02754900
C	-3.70987800	-2.36164600	0.61309300
H	3.78883800	-0.06822600	2.04458400
H	0.75412800	0.55488600	-1.87388700
H	3.13841900	0.08170300	-2.19951500
H	-0.99174400	0.90433100	2.98029500
H	1.41977700	0.42642700	3.46042700
H	-2.40294900	1.47326100	0.91350400
H	-1.06180500	1.64351000	-1.80678000
H	-0.90028600	4.07949500	-1.31339200
H	-1.48547500	3.78844900	0.32946100
H	0.07978300	3.14419300	-0.17916000
H	-3.68856400	2.77371100	-0.74019100
H	-2.93556200	3.24635200	-2.26686000
H	-3.50975400	1.60229300	-2.05560200
H	-3.27185400	-0.03550500	-0.92068800
H	0.86520300	-2.27590000	-1.45303600
H	0.46925500	-3.55419200	-0.26832000
H	-0.42561800	-3.45596500	-1.81374300
H	-4.55657700	-2.53287300	1.27565500
H	-4.02190200	-2.44785400	-0.42888500
H	-2.95616400	-3.12963800	0.79983900
Cl	5.27012300	-0.36793400	-0.38252700

Compound **1b**, optimization level PCM-ωB97-XD/6-311++G(2d,p)

Conformation #20

C	3.61362300	0.35639200	0.85142900
C	2.23579200	0.26589500	1.04391800
C	1.37269500	-0.29423100	0.07880600
C	1.91323300	-0.77894500	-1.11618500
C	3.27472400	-0.69843100	-1.32408600
C	4.09767400	-0.13427000	-0.33995300
C	0.03793800	-0.21624700	0.60236700
C	0.14984800	0.36762700	1.83045300
N	1.46546500	0.66060700	2.10115200
C	-1.19045800	-0.73218400	-0.09475100
C	-1.37799500	-2.24636500	0.20260200
C	-2.37057000	-2.93846200	-0.73071900
C	-1.71971700	-2.54809500	1.66086800
C	-2.43208200	0.12636400	0.21820200
C	-3.58240000	-0.06978100	-0.78646900
C	-2.12788000	1.61910500	0.21370700
O	-3.36896100	-0.16139100	-1.97072200
O	-2.49330200	2.37806100	1.07848600
O	-1.44814500	1.99118200	-0.86108500
C	-1.07851600	3.37251400	-0.93065600
C	-4.96372700	-0.08244600	-0.20188400
H	4.26982100	0.78797500	1.59574300
H	1.27633000	-1.21596900	-1.87673700
H	3.71492000	-1.06706700	-2.24118900
H	-0.61444600	0.61031600	2.55225100
H	1.79817000	1.10794000	2.93736900
H	-1.01672700	-0.63832000	-1.17048500
H	-0.39398400	-2.68389900	0.00178200
H	-2.16762800	-2.70862100	-1.77837100
H	-3.40367400	-2.65110000	-0.51623300
H	-2.30864600	-4.02115200	-0.59815400
H	-1.01975100	-2.07075900	2.34900100
H	-2.73036800	-2.21671600	1.91636900
H	-1.68090300	-3.62540600	1.83653700
H	-2.81009300	-0.07956800	1.21921400
H	-1.96676900	4.00316400	-0.93735700
H	-0.44764000	3.63267400	-0.08093300
H	-0.52733300	3.48057500	-1.86024500
H	-5.71535000	-0.14151100	-0.98598200
H	-5.06270500	-0.93306700	0.47799200
H	-5.11206700	0.81871300	0.39963300
Cl	5.82533900	-0.05211100	-0.64632900

Compound **1b**, optimization level PCM-ωB97-XD/6-311++G(2d,p)

Conformation #21

C	3.51677500	0.50291700	0.87434500
C	2.14833700	0.29748200	1.04555600
C	1.33607300	-0.27384800	0.04354500
C	1.92130300	-0.64647800	-1.17066600
C	3.27344600	-0.45041200	-1.35919700
C	4.04513900	0.11784800	-0.33644900
C	-0.00349400	-0.32887400	0.55854200
C	0.05691500	0.20110200	1.81563900
N	1.34364700	0.57607700	2.11453200
C	-1.20807600	-0.86693300	-0.16411100
C	-1.55068700	-2.33020600	0.21635800
C	-1.92621000	-2.51666800	1.68522000
C	-0.40334100	-3.26458500	-0.16303300
C	-2.41275200	0.07316700	0.00865800
C	-3.61208700	-0.32472900	-0.86610600
C	-2.03817900	1.49382900	-0.38776100
O	-3.45153800	-0.77218200	-1.97604000
O	-1.46092700	1.78870800	-1.40282800
O	-2.45073100	2.37809000	0.51639400
C	-2.15887400	3.75264500	0.23139900
C	-4.96743800	-0.10839700	-0.26202500
H	4.13222200	0.94169100	1.64869900
H	1.32575900	-1.08738100	-1.96143100
H	3.74602100	-0.73150900	-2.29108400
H	-0.72832900	0.35081900	2.54027200
H	1.63706300	0.99836200	2.97809800
H	-0.97801300	-0.86790000	-1.23277700
H	-2.41536500	-2.61522800	-0.39119300
H	-1.08606300	-2.26797200	2.33868700
H	-2.77973100	-1.90271800	1.98267400
H	-2.19557500	-3.55883400	1.87074200
H	0.49170100	-3.04610400	0.42581400
H	-0.68114200	-4.30491500	0.02107300
H	-0.14397800	-3.16611300	-1.22019500
H	-2.74329600	0.10623900	1.04661300
H	-2.56607900	4.31626100	1.06572700
H	-1.08171100	3.89958900	0.16051000
H	-2.63398200	4.05339300	-0.70164200
H	-5.75020800	-0.26997200	-0.99997900
H	-5.09376800	-0.80745800	0.57021500
H	-5.03638700	0.89855800	0.15694300
Cl	5.76351600	0.34732700	-0.61936400

Compound **1b**, optimization level PCM-ωB97-XD/6-311++G(2d,p)

Conformation #22

C	3.16649500	-0.77640900	-1.02127000
C	1.81482600	-1.11154200	-0.96910600
C	0.93219100	-0.59135700	0.00473500
C	1.45088200	0.28751900	0.96378600
C	2.78608400	0.63241100	0.92847600
C	3.61952900	0.09892200	-0.06162800
C	-0.36573700	-1.16094900	-0.25840900
C	-0.20417700	-1.97374100	-1.34103900
N	1.09648200	-1.94942500	-1.77422200
C	-1.67668000	-0.93144200	0.44814900
C	-1.57717400	-0.97693300	1.99103600
C	-0.84137000	-2.24022100	2.43945300
C	-2.95641300	-0.93194400	2.65190700
C	-2.40687100	0.32887900	-0.09166600
C	-3.14802700	0.07433600	-1.40851700
C	-1.49451000	1.53209500	-0.20826700
O	-3.25283500	-1.03841800	-1.86833400
O	-0.95666100	1.89726600	-1.22392000
O	-1.33389100	2.13690600	0.96750200
C	-0.45283600	3.26770200	0.97867400
C	-3.80573500	1.25753200	-2.06696100
H	3.82680800	-1.18293300	-1.77615700
H	0.81948900	0.70553000	1.73640900
H	3.19542700	1.31310400	1.66330000
H	-0.94288900	-2.57177900	-1.85063300
H	1.45827700	-2.46612200	-2.55660300
H	-2.32788800	-1.76228700	0.16161100
H	-1.01413600	-0.10800800	2.34157700
H	-1.36575500	-3.13373000	2.08504000
H	0.17980700	-2.27701300	2.05826200
H	-0.79790700	-2.28745900	3.52978900
H	-2.85997700	-1.07343100	3.73045500
H	-3.47126600	0.01858700	2.50115100
H	-3.59722700	-1.73099600	2.26560300
H	-3.20000700	0.60494900	0.60952100
H	-0.84825600	4.05733400	0.34078100
H	-0.41440500	3.59667700	2.01319900
H	0.53857300	2.97595900	0.63269800
H	-4.65452500	0.92001300	-2.65913900
H	-4.12458200	2.00270200	-1.33658000
H	-3.07749600	1.73292600	-2.72743200
Cl	5.31216400	0.56686900	-0.07184700

Compound **1b**, optimization level PCM-ωB97-XD/6-311++G(2d,p)

Conformation #23

C	-3.41501500	0.48862900	0.82219900
C	-2.05713900	0.74619300	1.00812900
C	-1.10090000	0.54443200	-0.00920400
C	-1.52692900	0.06986500	-1.25394300
C	-2.86568900	-0.18902300	-1.45736400
C	-3.78338100	0.02266100	-0.41871600
C	0.18063100	0.89323700	0.53494600
C	-0.05093800	1.28310700	1.82234400
N	-1.39025500	1.19967300	2.11157300
C	1.50955200	0.80735800	-0.16924900
C	2.27785200	2.14597800	-0.07006000
C	1.47950400	3.25797800	-0.75101000
C	3.68925600	2.09750600	-0.65306100
C	2.34345800	-0.38736700	0.36109900
C	3.34232300	-0.95023600	-0.66124200
C	1.44268500	-1.53461000	0.79638400
O	3.14938800	-0.86619200	-1.84979100
O	1.36376600	-1.95058400	1.92530800
O	0.74761700	-2.01850900	-0.22550800
C	-0.25952700	-2.98571100	0.08790400
C	4.54749200	-1.63531800	-0.08279200
H	-4.14108000	0.64224600	1.60963000
H	-0.81565300	-0.10592100	-2.05211200
H	-3.21627800	-0.55940200	-2.41169200
H	0.64384100	1.61863300	2.57652900
H	-1.80713700	1.43105300	2.99641400
H	1.31984400	0.60621000	-1.22757500
H	2.36802100	2.39050400	0.99528000
H	1.98540800	4.21928000	-0.63693900
H	1.38340900	3.05452700	-1.82231000
H	0.47595200	3.34952800	-0.33259500
H	3.67022500	1.81472600	-1.70834000
H	4.15450700	3.08261200	-0.57501700
H	4.33963700	1.39755700	-0.12333800
H	2.89029800	-0.09640300	1.26005700
H	-1.00721200	-2.53758100	0.74309200
H	-0.70826500	-3.25835900	-0.86293100
H	0.18329500	-3.85731800	0.56807900
H	5.12323600	-2.12518300	-0.86499200
H	5.17035200	-0.89382800	0.42523200
H	4.24131800	-2.36158500	0.67453200
Cl	-5.47808900	-0.32792900	-0.72060500

Compound **1b**, optimization level PCM-ωB97-XD/6-311++G(2d,p)

Conformation #37

C	3.54177700	-0.66981800	-1.04264400
C	2.15842000	-0.50118400	-1.06505300
C	1.46081900	0.18788200	-0.05034800
C	2.18397800	0.72672500	1.01898500
C	3.55408700	0.56940100	1.05911500
C	4.20703900	-0.12368800	0.03140200
C	0.06540500	0.16077400	-0.39644100
C	-0.01484000	-0.52206100	-1.57598300
N	1.23478300	-0.91971800	-1.98077700
C	-1.03603700	0.79862200	0.40916900
C	-1.52471900	2.13644700	-0.19721300
C	-0.35671400	3.11057000	-0.34630200
C	-2.63193300	2.77659000	0.64082300
C	-2.19997300	-0.16596200	0.76396700
C	-1.69100400	-1.53568900	1.21322000
C	-3.25133100	-0.35887500	-0.31100300
O	-2.06652900	-2.55346700	0.67780500
O	-3.06384500	-0.34989800	-1.50159500
O	-4.44858000	-0.55212100	0.23926500
C	-5.52779800	-0.80328700	-0.66792800
C	-0.71730500	-1.55023900	2.35853800
H	4.06762900	-1.20047800	-1.82552500
H	1.68372800	1.26624100	1.81511000
H	4.13106600	0.97820100	1.87809200
H	-0.88686800	-0.75956600	-2.16196900
H	1.42957900	-1.45128500	-2.81130100
H	-0.59578700	1.05715300	1.37922300
H	-1.91938300	1.92254300	-1.19465400
H	0.07888300	3.34421600	0.63073600
H	0.43324600	2.70179300	-0.97821000
H	-0.69825500	4.04690200	-0.79295400
H	-2.30209300	2.91739100	1.67550200
H	-2.88983700	3.75798100	0.23732100
H	-3.54885100	2.18353100	0.65749500
H	-2.71705000	0.24612300	1.63525900
H	-5.66993300	0.05003300	-1.33033600
H	-5.32401300	-1.69643900	-1.25767100
H	-6.40525700	-0.95067900	-0.04487700
H	-0.69114900	-2.54274200	2.80411600
H	0.27844400	-1.30931000	1.97634000
H	-0.97345300	-0.80100500	3.10895800
Cl	5.95225200	-0.29976200	0.12277400

Compound **1b**, optimization level PCM-ωB97-XD/6-311++G(2d,p)

Conformation #44

C	-3.39197600	-0.23915600	0.85715900
C	-2.05000100	-0.45637400	1.16720400
C	-1.03883100	-0.51212500	0.18323000
C	-1.40122200	-0.33877600	-1.15761600
C	-2.72340700	-0.12432100	-1.48461000
C	-3.69384200	-0.07837300	-0.47499000
C	0.20646900	-0.72837000	0.87237900
C	-0.10375000	-0.79349200	2.19768700
N	-1.45338500	-0.63490700	2.38418900
C	1.58676600	-0.87627800	0.29501400
C	1.67096200	-2.08539500	-0.66853100
C	1.25169500	-3.36897500	0.04697400
C	3.05483200	-2.26881800	-1.28999900
C	2.08741000	0.42900400	-0.38291300
C	3.60966100	0.60843000	-0.31003900
C	1.47886800	1.64811400	0.29214300
O	4.26416100	0.12280600	0.57997200
O	1.79956800	2.05530400	1.38086100
O	0.53641400	2.20308200	-0.46079600
C	-0.21813100	3.25782000	0.14660700
C	4.21784100	1.45484900	-1.39214100
H	-4.15603100	-0.19570700	1.62232900
H	-0.65635300	-0.35657700	-1.94341800
H	-3.01876800	0.01391900	-2.51632400
H	0.55162400	-0.94474700	3.04154000
H	-1.92101900	-0.63627000	3.27378600
H	2.26849300	-1.06426800	1.13003200
H	0.96171100	-1.90765300	-1.48468100
H	1.91864900	-3.57159400	0.89126400
H	0.23277600	-3.30390000	0.43122600
H	1.30525000	-4.22036100	-0.63514000
H	3.05967000	-3.15331300	-1.93059700
H	3.35066300	-1.42359900	-1.91571500
H	3.81688100	-2.40449100	-0.51839200
H	1.78248700	0.45262100	-1.43106700
H	0.43416300	4.08635000	0.41962800
H	-0.93898600	3.57006900	-0.60349400
H	-0.73017100	2.88157500	1.03240900
H	5.27056500	1.63876200	-1.18925500
H	4.11042700	0.94182100	-2.35188400
H	3.67917800	2.40218800	-1.47845200
Cl	-5.36521900	0.20137700	-0.93639800
